# Supplementary material for: Effect of ethyl methane sulfonate mutagenesis on phenological, yield-related and yield traits in cowpea (Vigna unguiculata (L.) Walp)
Source: PLoS One. 2026 Jul 20;21(7):e0354037. doi: 10.1371/journal.pone.0354037 (PMC13384270; doi:10.1371/journal.pone.0354037)
Supplement: S1 Table — (PDF) [file pone.0354037.s001.pdf]

S1 Table: Trait dataset and cluster grouping of individual M_1 cowpea plants across EMS treatments and control

Trt	ID	*dtg*	*dff*	*d50f*	*dfh*	*d50mp*	*pdl*	*ln*	*sdl*	*sdw*	*sdt*	*swg*	yld	Cluster group
0mM	A01	4	40	60	67	89	12.94	13	8.94	5.6	4.3	23.2	55.68	1
	A02	4	39	60	53	88	17	18	6.62	4.53	3.86	20.34	42.71	2
	A03	3	40	56	49	87	13.24	21	8.98	5.4	4.13	17.94	43.05	2
	A04	3	35	52	50	81	13.2	13	7.03	4.35	3.29	23.96	52.71	2
	A05	3	43	66	58	93	14.24	18	7.22	4.7	4.02	20.08	43.38	2
	A06	3	30	60	60	78	13.58	21	6.72	4.42	3.7	20.97	41.53	2
	A07	4	40	55	47	75	13.8	12	9.26	5.71	4.19	24.87	34.82	3
	A08	4	41	62	53	83	11.55	19	7.91	5.11	4.17	19.93	36.27	2
	A09	4	37	52	41	67	15.4	14	6.16	4.44	4.41	20.92	72.81	2
	A10	4	37	54	58	89	13.09	16	6.23	4.15	3.64	22.13	44.92	2
	A11	4	40	62	45	74	15.29	18	7.37	4.84	4.29	21.1	31.66	2
	A12	4	44	59	46	76	15.95	17	8.98	5.4	4.13	21.01	43.71	3
	A13	3	43	60	49	87	17.58	16	6.99	4.46	3.81	17.82	42.78	2
	A14	5	30	52	53	80	16.64	18	9.65	5.67	4.28	23.13	73.78	3
	A15	4	39	54	47	80	15.57	17	8.24	5.25	4.13	19.75	40.09	3
	A16	3	45	66	40	70	14.08	24	6.77	4.57	4.36	20.79	39.49	2
	A17	4	31	54	43	66	17.21	12	6.74	4.34	3.42	20.36	61.08	2
	A18	3	32	49	56	94	14.25	20	7.57	5.03	4.92	22.64	67.93	4
	A19	2	35	59	42	72	15.3	13	6.21	4.46	4.43	20.61	54.41	2
	A20	3	33	48	47	84	16.62	20	6.38	4.3	4.1	22.57	72.66	4
	A21	4	40	57	44	71	13.33	17	6.43	4.47	4.19	18.36	50.5	2
	A24	4	39	58	49	72	15.42	17	5.68	3.94	3.63	19.63	61.83	2
	A25	3	39	59	50	70	12.83	21	6.24	4.19	3.71	24.67	51.81	2
	A26	3	37	59	54	86	15.3	23	6.76	4.54	4.53	22.04	52.89	4
	A27	4	32	53	53	84	16	16	11.59	6.08	4.82	19.21	22.86	3
	A28	4	40	65	43	79	11.88	12	6.07	4.03	3.85	21.97	85.68	2
	A29	2	39	57	44	81	14.92	17	8.49	5.13	3.92	23.24	23.24	3
	A30	4	43	68	51	88	13.53	18	8.35	5.15	4.48	18.63	50.31	2
	A31	3	40	64	40	69	14.9	13	6.61	4.53	4.52	18.39	74.66	2
20mM	B01	5	50	70	68	92	12.13	18	6.33	4.24	4.06	22.28	55.69	5
	B02	4	46	66	59	98	13.23	21	6.65	4.38	3.66	26.08	28.69	5
	B03	5	42	60	53	79	14.98	13	6.94	4.58	4.23	25.41	37.36	5
	B04	6	43	60	60	100	16.28	18	9.26	5.71	4.19	25.92	54.44	1
	B05	5	47	66	62	82	13.8	21	10.1	5.78	4.92	25.17	49.83	1
	B06	5	51	75	54	76	15.75	12	10.35	5.76	4.91	25.9	62.15	6
	B07	7	38	60	63	89	11.45	19	11.65	6.44	5.19	29.99	67.18	6
	B08	5	49	74	68	102	15.25	14	9.85	5.67	5.12	22.68	41.27	1
	B09	7	52	74	71	98	13	21	11.59	6.08	4.82	27.48	74.2	6
	B10	6	47	63	56	81	14.31	23	7.03	4.48	3.79	20.06	46.14	2
	B11	7	52	70	60	95	13.3	16	9.75	5.66	4.99	26.1	26.1	3
	B12	3	41	59	49	87	17.7	13	10.4	5.9	5.16	26.32	48.7	6
	B13	4	47	62	66	100	14.25	18	9.9	5.72	4.87	23.49	59.2	1
	B14	7	53	69	58	87	14.5	21	7.18	4.88	4.76	24.86	45.75	5
	B15	3	54	71	55	76	15.25	16	9.75	5.88	4.83	27.77	79.15	1
	B16	5	53	75	64	102	14.7	18	9.75	5.68	4.85	26.24	75.56	1
	B17	3	41	65	71	93	13.7	17	10.4	5.82	4.84	27.34	71.37	6
	B18	6	54	71	62	98	15.5	12	10	5.62	4.89	28.33	45.32	3
	B19	5	36	51	48	68	19.88	19	6.23	4.17	4	24.82	93.59	4
	B20	5	34	57	52	72	14.7	14	6.77	4.57	4.36	26.63	42.6	5

Values highlighted red indicate trait means significantly (P<0.05) below the control, while green indicate values significantly (P<0.05) above the control.Key: Trt= Treatment ID=Genotype Identifier, dtg days to germination, dff = days to first flower, d50f = days to 50% flowering, dfh = days to first harvest, d50mp = days to 50% mature pods, pdl = pod length, pdw = pod width, ln = number of locules, nsp = number of seeds per pod, psa = percent seed abortion, sdl = seed length, sdw = seed width, sdt = seed thickness, swgt = seed weight, yld = yield per plant

S1 Table: Trait dataset and cluster grouping of individual M_1 cowpea plants across EMS treatments and control (*cont'd*)

Trt	ID	*dtg*	*dff*	*d50f*	*dfh*	*d50mp*	*pdl*	*ln*	*sdl*	*sdw*	*sdt*	*swg*	*yld*	Cluster group
20mM	B21	5	47	63	70	91	13.6	13	9.85	5.64	5.09	22.6	81.37	1
	B22	6	36	56	70	97	12.1	20	7.04	4.98	4.85	21.91	88.96	1
	B23	7	48	73	55	79	13.04	17	8.26	5.04	3.93	22.04	46.28	5
	B24	6	50	74	52	75	13.35	13	8.7	5.13	4.91	17.81	18.7	3
	B25	7	39	61	58	89	17.75	18	9.65	5.31	4.52	25.92	76.99	1
	B26	4	47	72	64	100	14	21	10.1	5.54	4.87	24.26	91.45	1
	B27	7	39	55	56	85	16.1	12	7.04	4.68	4.28	25.59	67.56	4
	B28	4	39	60	67	87	12.13	19	7.3	4.75	4.04	23	103.5	1
	B29	6	43	66	61	94	13.27	14	6.62	4.53	3.86	24.2	45.73	5
	B30	7	40	61	63	95	14.1	13	6.46	4.29	4.1	23.82	28.58	5
	B31	3	39	60	58	98	14.85	20	9.9	5.46	5	24.65	107.22	1
	B32	6	47	71	49	85	14.22	17	6.14	4.06	3.87	22.04	66.11	4
	B33	6	37	61	56	85	16.33	19	6.1	4.05	3.31	27.88	125.44	4
	B34	7	41	57	53	85	12.44	17	7.04	4.98	4.85	22.2	38.86	5
	B35	7	51	68	56	91	14.75	13	10.05	5.24	3.56	26.06	63.85	5
	B36	4	33	54	64	100	14.1	19	10.15	5.18	3.91	23.62	64.48	1
	B37	7	53	73	62	82	14.35	17	11.25	5.77	3.71	26.15	82.38	1
	B38	5	43	61	61	81	14	13	10.3	5.01	3.84	27.88	42.94	5
	B39	6	54	71	49	85	13.25	19	10.4	5.67	4.46	26.71	86.53	1
	B40	7	37	58	61	90	16.6	15	10.2	5.5	4.4	26.06	68.8	6
	B41	7	33	50	60	99	17.52	18	6.43	4.47	4.19	25.04	66.61	4
	B42	5	49	74	69	107	12.7	20	6.74	4.34	3.42	26.21	46.13	5
	B43	3	37	56	58	96	12.25	18	9.3	4.73	3.72	23.01	68.33	5
	B44	6	48	70	69	107	14.43	19	6.65	4.66	4.27	21.38	29.94	5
	B45	3	48	67	62	96	13.75	20	10.1	5.57	4.08	23.17	76.45	1
	B46	6	46	65	55	80	10.5	18	9.5	4.7	3.72	27.56	46.86	5
	B47	4	52	70	59	89	14.5	19	10.15	5.15	3.97	24.42	72.54	1
	B48	4	39	59	72	105	14.5	14	9.65	5.36	3.86	25.18	30.22	5
40mM	C01	4	49	68	67	103	13.95	21	10	4.95	3.8	23.44	50.63	1
	C02	3	43	59	38	68	14.05	21	9.25	4.69	3.71	17.7	77.01	2
	C03	4	52	67	61	85	11.75	12	9.6	4.67	3.43	25.95	61.76	1
	C04	4	40	55	50	76	11.5	17	10	5.5	4.18	25.96	68.52	1
	C05	7	47	67	62	91	14.2	11	9.55	5.61	4.29	26.03	105.43	1
	C06	7	45	70	66	92	11.95	12	9.85	4.84	3.68	25.34	44.6	5
	C07	3	35	60	60	89	11.75	17	9.95	5.49	4.2	28.39	78.35	1
	C08	4	46	62	67	106	14	11	9.95	5.48	4.09	23.9	55.22	1
	C09	3	46	71	61	98	13.15	11	9.85	5.27	3.95	27.53	60.57	5
	C10	4	55	76	68	97	16.75	12	9.75	5.09	4	25.64	57.43	1
	C11	5	38	54	58	96	11	14	9.2	5.13	4.05	23.3	39.61	5
	C12	6	44	59	67	98	13.6	18	9.85	5.56	3.97	26.7	82.24	1
	C13	6	45	67	65	93	13.6	18	8.1	4.72	3.82	26.98	87.43	1
	C14	3	43	68	39	74	14.95	17	9.4	4.63	3.49	17.77	52.79	2
	C15	3	50	71	66	88	15.6	19	10	5.18	4.29	27.88	57.98	1
	C16	7	52	69	55	80	12.5	17	9.6	5.14	3.98	27.98	76.93	1
	C17	7	45	64	55	95	16.65	13	10.45	5.01	3.51	20.9	68.96	4
	C18	5	53	78	66	91	12.6	19	9.65	4.87	3.99	26.66	72.78	1
	C19	4	52	72	65	88	14.8	17	9.85	5.25	3.83	26.46	47.62	5
	C20	4	46	71	47	83	13.7	13	9.3	4.79	3.51	21.5	54.19	5
	C21	3	53	73	58	90	10.5	19	9	5.02	4.06	25.8	59.33	1

Values highlighted red indicate trait means significantly (P<0.05) below the control, while green indicate values significantly (P<0.05) above the control.Key: Trt= Treatment ID=Genotype Identifier, dtg days to germination, dff = days to first flower, d50f = days to 50% flowering, dfh = days to first harvest, d50mp = days to 50% mature pods, pdl = pod length, pdw = pod width, ln = number of locules, nsp = number of seeds per pod, psa = percent seed abortion, sdl = seed length, sdw = seed width, sdt = seed thickness, swgt = seed weight, yld = yield per plant

S1 Table: Trait dataset and cluster grouping of individual M_1 cowpea plants across EMS treatments and control *(cont'd)*

Trt	ID	*dtg*	*dff*	*d50f*	*dfh*	*d50mp*	*pdl*	*ln*	*sdl*	*sdw*	*sdt*	*swg*	yld	Cluster group
40mM	C22	5	55	73	51	78	14.9	15	10	5.5	4.32	25.76	27.04	3
	C23	5	43	64	55	81	12.05	18	9.6	4.79	3.84	21.7	42.96	1
	C24	4	43	65	56	76	13.5	11	9.8	5.31	3.85	26.02	94.7	1
	C25	4	40	59	62	102	12.5	12	9.4	4.81	3.63	23.51	55.96	5
	C26	7	51	70	54	88	11.95	14	9.85	5.25	3.77	23.6	69.4	1
	C27	5	48	69	67	95	6.95	19	9	4.58	5.59	23.01	48.33	1
	C28	7	49	72	67	91	15.95	17	10	5.33	3.86	26.46	47.62	5
	C29	5	39	62	56	92	15.4	13	10	4.98	3.81	25.45	42.76	5
	C30	3	37	57	62	93	14.9	20	10	5.58	3.98	21.34	59.76	1
	C31	5	42	59	50	83	12.75	18	10	4.88	3.57	27.56	48.24	5
	C32	7	34	49	59	84	14.95	19	9.85	4.91	3.87	27.16	62.74	4
	C33	3	52	77	50	76	12.25	19	9.35	5.33	4.21	24.58	63.91	1
	C34	3	44	68	57	93	15.75	15	8.65	5.03	4.35	24.24	56.71	4
	C35	5	48	70	58	94	15.45	18	9.85	4.88	4.01	23.82	60.04	1
	C36	3	54	74	54	83	13.95	12	10.05	4.78	3.63	24.73	61.83	1
	C37	4	49	67	51	76	13.9	17	9.8	5.28	4.08	23.83	31.45	1
	C38	6	34	59	62	89	8.77	11	8.95	5.06	3.81	19.95	39.5	5
	C39	6	37	57	52	92	15.5	19	10.1	4.98	3.91	22.71	27.02	5
	C40	6	35	58	72	98	13.5	17	10	5.4	3.68	24.03	28.83	5
	C41	5	55	80	64	89	14.5	13	10	4.83	3.71	26.36	90.96	1
	C42	7	36	59	58	95	13.35	19	10	5.59	4.49	25.19	25.19	5
	C43	5	35	53	71	95	14	15	10	5.55	4.45	27.28	90.01	1
	C44	5	41	57	55	95	15.5	18	10	4.97	4.14	25.65	76.17	1
	C45	3	40	59	54	84	15.75	12	11.05	5.07	3.8	27.77	83.32	1
	C46	5	52	71	64	103	16.15	17	10.9	5.69	4.25	24.74	59.87	1
	C47	4	40	57	63	87	13.95	11	10	5.58	4.09	23.3	37.51	3
	C48	4	50	73	62	92	16.75	13	10	5.19	3.99	19.97	19.97	3
	C49	7	39	55	59	80	14.75	8	10.4	5.29	3.92	22.82	60.26	3
	C50	3	51	69	62	84	14.5	9	10.1	5.39	3.93	23.53	54.59	5
	C51	4	53	70	62	87	12.5	13	9.9	5.3	3.96	19.66	21.63	3
	C52	4	39	60	64	86	15.9	8	9.9	5.17	4.02	23.13	60.13	4
	C53	6	39	63	62	85	14.75	9	9.75	5.22	3.9	19.28	75.19	4
	C54	5	41	59	54	80	16.55	12	10.15	5.58	3.55	25.23	55.51	3
	C55	7	53	70	51	89	14.5	11	9.85	4.76	3.69	25.06	52.62	5
80mM	D01	7	49	69	49	80	13.5	17	10	4.94	3.95	22.58	31.17	3
	D02	6	50	72	54	92	10.75	10	10.7	5.71	4.28	24.18	42.55	1
	D03	3	40	58	70	93	13.5	9	9.85	5.61	4.56	24.13	74.33	1
	D04	5	55	73	72	100	12.5	13	9.35	5.34	3.81	18.82	76.2	1
	D05	7	35	53	59	96	11.75	10	9.95	5.69	4.18	22.05	45.64	5
	D06	4	34	59	55	93	15	9	10.3	5.68	4.16	20.79	50.32	3
	D07	4	46	70	49	83	12.5	13	9.7	5.44	3.91	20.34	31.72	5
	D08	4	48	69	61	81	15.25	13	10.55	6.11	4.94	26.24	43.3	3
	D09	5	47	65	66	100	15	9	10.3	5.21	4.28	27.62	39.78	1
	D10	4	53	78	58	97	13.75	10	9.85	5.04	4.38	24.53	35.57	3
	D11	7	46	62	61	81	14	12	9.95	5.55	4.91	22.22	34	1
	D12	3	43	68	59	95	15.75	9	10.05	5.58	4.7	22.36	60.82	6

Values highlighted red indicate trait means significantly (P<0.05) below the control, while green indicate values significantly (P<0.05) above the control.Key: Trt= Treatment ID=Genotype Identifier, dtg days to germination, dff = days to first flower, d50f = days to 50% flowering, dfh = days to first harvest, d50mp = days to 50% mature pods, pel = peduncle length at first harvest, npp = number of pods per peduncle, nptm = number of pods per plant at maturity, nspt = number of seeds per plant, pdl = pod length, pdw = pod width, ln = number of locules, nsp = number of seeds per pod, psa = percent seed abortion, sdl = seed length, sdw = seed width, sdt = seed thickness, swgt = seed weight, yld = yield per plant

S1 Table: Trait dataset and cluster grouping of individual M_1 cowpea plants across EMS treatments and control *(cont'd)*

Trt	ID	*dtg*	*dff*	*d50f*	*dfh*	*d50mp*	*pdl*	*ln*	*sdl*	*sdw*	*sdt*	*swg*	yld	Cluster group
80mM	D13	6	42	59	69	91	16.5	13	10.05	5.2	4.67	27	64.8	1
80mM	D14	6	52	74	45	81	12.75	12	9.4	5.37	4.46	24.66	54.24	1
80mM	D15	4	43	68	61	92	14.5	9	10.5	5.47	4	21.45	36.03	3
80mM	D16	5	48	68	65	92	13	13	9.9	5.02	3.95	22.74	68.23	1
80mM	D17	6	40	56	64	86	12.75	11	9.4	5.91	4.89	23.07	49.84	6
80mM	D18	3	44	68	64	101	11.45	7	9.95	4.94	3.97	25.55	35.77	1
80mM	D19	5	43	58	59	91	14.5	12	11	5.24	3.41	23.94	55.07	5
80mM	D20	4	55	75	64	103	11.25	13	10.75	5.41	4.21	22.04	65.9	1
80mM	D21	3	37	59	59	97	17.5	8	11	5.35	3.74	24.48	42.83	3
80mM	D22	7	47	62	63	92	14.5	14	9.35	4.73	3.74	20.59	40.77	4
80mM	D23	7	37	55	57	78	16	13	9.35	5.21	3.94	22.7	25.88	3
80mM	D24	4	40	62	72	109	14.25	8	9.15	4.95	4.07	17.35	58.65	4
80mM	D25	3	45	68	68	90	15	9	11	5.62	4.15	24.44	102.66	1
80mM	D26	4	46	63	71	110	17.1	13	8.95	5.02	3.86	22.7	22.7	3
80mM	D27	4	44	62	61	86	16.25	8	10	5.31	4.04	21.91	42.73	4
80mM	D28	6	43	66	60	92	17.1	9	10.45	5.48	4.28	22.32	75	4
80mM	D29	7	39	62	58	82	12.75	12	7.95	4.28	3.45	21.58	69.93	4
80mM	D30	4	35	60	56	84	12.75	11	10.25	5.02	3.79	25.06	36.08	5
80mM	D31	4	35	55	54	87	14.9	17	10.75	5.17	4.02	21.98	24.17	5
80mM	D32	4	44	62	68	96	14.15	11	10.2	6.05	4.82	22.16	37.23	1
80mM	D33	4	37	60	48	85	11.25	7	10.3	4.81	3.92	25.86	46.54	5
80mM	D34	4	45	66	52	83	12.25	12	10.25	4.97	3.46	21.21	23.75	3
80mM	D35	7	41	66	58	81	16.75	13	9.3	5.09	4.06	21.79	35.96	3
80mM	D36	3	53	71	49	73	14	8	11	5.64	4.37	28.29	74.69	6
80mM	D37	4	43	61	59	80	15.25	9	10	5.38	4.08	25.05	50.86	5
80mM	D38	5	42	57	62	88	15.5	10	9.3	5.14	3.5	17.49	44.24	4
80mM	D39	4	40	56	52	90	15.75	9	9.4	5.3	4.06	19.27	48.57	3
80mM	D40	4	44	69	54	77	9.95	13	8.55	5.18	3.96	19.42	55.55	2
80mM	D41	7	45	63	59	81	15	12	9	4.79	3.75	19.76	80.03	4
80mM	D42	3	55	80	66	96	9.95	11	9.9	4.72	3.72	22.02	29.06	3
80mM	D43	3	54	73	48	69	11.7	17	10.5	4.9	3.67	21.98	42.19	1
80mM	D44	6	48	65	52	86	9.5	12	11.6	6.03	4.46	24.35	64.29	1
80mM	D45	4	47	68	60	92	11.5	9	9.8	5.4	4.22	23.58	106.09	1
80mM	D46	3	44	62	57	78	12.5	13	9.8	5.05	4.15	21.53	45.21	3
80mM	D47	3	38	59	58	78	13	13	9.85	5.08	3.99	18.45	18.45	3
80mM	D48	5	47	65	52	84	13.65	8	10	5.3	4.24	22.52	85.13	1
80mM	D49	7	52	72	62	83	11.2	9	10.45	5.48	4.03	22.93	79.1	1
80mM	D50	3	42	60	52	92	13.1	12	9.65	4.82	3.7	22.5	43.19	5
80mM	D51	7	33	56	50	83	8.45	11	9.55	4.76	3.84	22.62	64.7	5
80mM	D52	5	55	77	56	84	14.2	17	10.4	4.87	3.87	19.54	22.48	3
80mM	D53	7	43	62	59	99	11.4	12	9.8	5.51	4.38	22.83	55.25	1
80mM	D54	5	47	71	60	85	11.5	9	9.8	5.44	4.1	20.24	28.33	3
80mM	D55	4	37	62	43	74	14.25	13	9.05	4.85	3.85	20.31	35.34	2
80mM	D56	5	53	69	57	94	13.5	23	10.15	5.03	3.78	28.68	111.85	1

Values highlighted red indicate trait means significantly (P<0.05) below the control, while green indicate values significantly (P<0.05) above the control.Key: Trt= Treatment ID=Genotype Identifier, dtg = days to germination, dff = days to first flower, d50f = days to 50% flowering, dfh = days to first harvest, d50mp = days to 50% mature pods, pdl = pod length, pdw = pod width, ln = number of locules, nsp = number of seeds per pod, psa = percent seed abortion, sdl = seed length, sdw = seed width, sdt = seed thickness, swgt = seed weight, yld = yield per plant
