# Supplementary material for: Effect of ethyl methane sulfonate mutagenesis on phenological, yield-related and yield traits in cowpea (Vigna unguiculata (L.) Walp)
Source: PLoS One. 2026 Jul 20;21(7):e0354037. doi: 10.1371/journal.pone.0354037 (PMC13384270; doi:10.1371/journal.pone.0354037)
Supplement: S2 Table — (PDF) [file pone.0354037.s002.pdf]

S1 Table: PCA loadings and variance explained for traits in M_1 cowpea

**Traits**	**PC1**	**PC2**	**PC3**	**PC4**	**PC5**	**PC6**	**PC7**	**PC8**	**Uniqueness**
sdl	0.73								0.18
sdw	0.70		0.50						0.11
dfh	0.68			0.50				-0.32	0.14
dff	0.61		-0.64						0.10
d50mp	0.59			0.51				-0.40	0.13
d50f	0.57		-0.68						0.09
swg	0.53				0.35			0.43	0.36
sdt	0.38		0.42			0.52	-0.38		0.24
nspt		0.96							0.02
yld		0.95							0.02
nsp		0.75		0.36	-0.38		-0.32		0.03
nptm		0.32		-0.66			0.53		0.04
pdl			0.42						0.77
pdw					0.54	-0.35		0.32	0.40
ln					0.47	0.63			0.22
dtg					0.46	-0.33			0.51
psa					-0.42			0.32	0.52
npp						0.32	0.41	0.43	0.43
**Eigenvalue**	**3.18**	**2.71**	**1.70**	**1.51**	**1.34**	**1.17**	**1.07**	**1.03**	
**Proportion %**	**17.70**	**15.10**	**9.40**	**8.40**	**7.50**	**6.50**	**6.00**	**5.70**	
**Cumulative %**	**17.70**	**32.70**	**42.20**	**50.60**	**58.00**	**64.50**	**70.50**	**76.20**	

PC= Principal component, sdl = seed length, sdw = seed width, dfh = days to first harvest, dff = days to first flower, , d50f = days to 50% flowering, , d50mp = days to 50% mature pods, swgt = seed weight, sdt = seed thickness, nspt = number of seeds per plant npp, yld = yield per plant, nsp = number of seeds per pod, nptm = number of pods per plant at maturity, pdl = pod length, pdw = pod width, ln = number of locules dtg = days to germination, psa = percent seed abortion, npp= number of pods per peduncle
