# Supplementary material for: Effect of ethyl methane sulfonate mutagenesis on phenological, yield-related and yield traits in cowpea (Vigna unguiculata (L.) Walp)
Source: PLoS One. 2026 Jul 20;21(7):e0354037. doi: 10.1371/journal.pone.0354037 (PMC13384270; doi:10.1371/journal.pone.0354037)
Supplement: Attachment — Submitted filename: PONE-D-26-01099_Reviewer.pdf [file pone.0354037.s004.pdf]

PLOS One

Effect of Ethyl Methane Sulfonate Mutagenesis on Phenological, Yield-Related and Yield Traits in Cowpea (Vigna unguiculata (L.) Walp)

--Manuscript Draft--

**Manuscript Number:**	PONE-D-26-01099
**Article Type:**	Research Article
**Full Title:**	Effect of Ethyl Methane Sulfonate Mutagenesis on Phenological, Yield-Related and Yield Traits in Cowpea (Vigna unguiculata (L.) Walp)
**Short Title:**	Effect of EMS on Agronomic Traits in Cowpea
**Corresponding Author:**	HENRY KOFI MENSAH, MPhil Botany<br>University of Ghana<br>ACCRA, GHANA
**Keywords:**	ethyl methanesulfonate (EMS); induced mutagenesis; mutation breeding; cowpea (Vigna unguiculata); genetic variability; yield traits; phenological traits; seed traits; principal component analysis; cluster analysis
**Abstract:**	This study investigated the impact of ethyl methane sulfonate (EMS) on the M1 generation of cowpea (Vigna unguiculata (L.) Walp). Seventy-five seeds of 'Wang Kae' cowpea variety were soaked in water for six hours and later subjected to three different doses of ethyl methane sulfonate (20 mM, 40 mM and 80 mM) with fifty serving as a control. Phenological, yield-related, and yield-specific data were collected in accordance with the International Board for Plant Genetic Resources cowpea descriptor. Data analysis was performed using Jamovi 2.7.15 and Jasp 0.95.4.0 to compute one-way ANOVA and post hoc contrast, principal component biplot, and cluster analyses. No optimal EMS concentration (LD50) for inducing mutations was identified. The maximum seed germination rate of 89.33% and seedling survival rate of 74.67% were recorded at 80 mM, whereas the control group exhibited the lowest rates, with 70.00% germination and 62.00% survival. The trend observed for both seed germination and seedling survival rates was: 0 mM < 20 mM < 40 mM < 80 mM. Significant differences ($P<0.05$) in all phenological traits, pod length, locule number, seed traits, and yield per plant were found between the control and treatments. Yield was significantly higher ($P = 0.047$) at 20 mM, with a mean of 61.19 ± 3.34 g, compared to the control. Contrast analysis identified genotypes B33 and D56 as the most productive mutants, yielding 125.44 g and 111.85 g, respectively. Principal component analysis produced eighteen components, with the first four accounting for 50.60% of total variation. Biplot analysis revealed that PC1 and PC2 encompassed all phenological traits, key seed traits, and yield attributes, especially for genotypes B33 and D56. Cluster analysis divided the 190 genotypes into six groups, with B33 and D56 constituting distinct clusters 4 and 1, respectively. In conclusion, the EMS mutagenesis was effective in inducing the unique variations that will be useful for breeding and development of new farmer preferred varieties.
**Order of Authors:**	HENRY KOFI MENSAH, MPhil Botany
	Ralieva Awura Korkor Nortey, MPhil Botany
	Isaac Kojo Asante
	Felicia Oppong-Adjei
**Additional Information:**	
**Question**	**Response**
**Financial Disclosure**<br><br>Enter a financial disclosure statement that describes the sources of funding for the work included in this submission. Review the submission guidelines for detailed	The author(s) received no specific funding for this work.

Powered by Editorial Manager® and ProduXion Manager® from Aries Systems Corporation

requirements. View published research articles from *PLOS ONE* for specific examples.<br><br>This statement is required for submission and **will appear in the published article** if the submission is accepted. Please make sure it is accurate.<br><br>**Funded studies**<br>Enter a statement with the following details:<br>• Initials of the authors who received each award<br>• Grant numbers awarded to each author<br>• The full name of each funder<br>• URL of each funder website<br>• Did the sponsors or funders play any role in the study design, data collection and analysis, decision to publish, or preparation of the manuscript?<br><br>Did you receive funding for this work?	
**Competing Interests**<br><br>Use the instructions below to enter a competing interest statement for this submission. On behalf of all authors, disclose any competing interests that could be perceived to bias this work—acknowledging all financial support and any other relevant financial or non-financial competing interests.<br><br>This statement is required for submission and **will appear in the published article** if the submission is accepted. Please make sure it is accurate and that any funding sources listed in your Funding Information later in the submission form are also declared in your Financial Disclosure statement.<br><br>View published research articles from *PLOS ONE* for specific examples.	The authors have declared that no competing interests exist.

Powered by Editorial Manager® and ProduXion Manager® from Aries Systems Corporation

**NO authors have competing interests**<br><br>Enter: *The authors have declared that no competing interests exist.*<br><br>**Authors with competing interests**<br><br>Enter competing interest details beginning with this statement:<br><br>*I have read the journal's policy and the authors of this manuscript have the following competing interests: [insert competing interests here]*<br><br>* typeset	
**Ethics Statement**<br><br>Enter an ethics statement for this submission. This statement is required if the study involved:<br><br>• Human participants<br>• Human specimens or tissue<br>• Vertebrate animals or cephalopods<br>• Vertebrate embryos or tissues<br>• Field research<br><br>Write "N/A" if the submission does not require an ethics statement.<br><br>General guidance is provided below. Consult the submission guidelines for detailed instructions. **Make sure that all information entered here is included in the Methods section of the manuscript.**	N/A

**Format for specific study types**<br><br>**Human Subject Research (involving human participants and/or tissue)**<br>• Give the name of the institutional review board or ethics committee that approved the study<br>• Include the approval number and/or a statement indicating approval of this research<br>• Indicate the form of consent obtained (written/oral) or the reason that consent was not obtained (e.g. the data were analyzed anonymously)<br><br>**Animal Research (involving vertebrate animals, embryos or tissues)**<br>• Provide the name of the Institutional Animal Care and Use Committee (IACUC) or other relevant ethics board that reviewed the study protocol, and indicate whether they approved this research or granted a formal waiver of ethical approval<br>• Include an approval number if one was obtained<br>• If the study involved *non-human primates*, add *additional details* about animal welfare and steps taken to ameliorate suffering<br>• If anesthesia, euthanasia, or any kind of animal sacrifice is part of the study, include briefly which substances and/or methods were applied<br><br>**Field Research**<br><br>Include the following details if this study involves the collection of plant, animal, or other materials from a natural setting:<br>• Field permit number<br>• Name of the institution or relevant body that granted permission	
**Data Availability**<br><br>Authors are required to make all data underlying the findings described fully available, without restriction, and from the time of publication. PLOS allows rare exceptions to address legal and ethical concerns. See the PLOS Data Policy and FAQ for detailed information.	Yes - all data are fully available without restriction

A Data Availability Statement describing where the data can be found is required at submission. Your answers to this question constitute the Data Availability Statement and **will be published in the article**, if accepted.<br><br>**Important:** Stating 'data available on request from the author' is not sufficient. If your data are only available upon request, select 'No' for the first question and explain your exceptional situation in the text box.<br><br>Do the authors confirm that all data underlying the findings described in their manuscript are fully available without restriction?	
**Describe where the data may be found in full sentences. If you are copying our sample text, replace any instances of XXX with the appropriate details.**<br><br>• If the data are **held or will be held in a public repository**, include URLs, accession numbers or DOIs. If this information will only be available after acceptance, indicate this by ticking the box below. For example: *All XXX files are available from the XXX database (accession number(s) XXX, XXX.*).<br>• If the data are all contained **within the manuscript and/or Supporting Information files**, enter the following: *All relevant data are within the manuscript and its Supporting Information files.*<br>• If neither of these applies but you are able to provide **details of access elsewhere**, with or without limitations, please do so. For example:<br><br>*Data cannot be shared publicly because of [XXX]. Data are available from the XXX Institutional Data Access / Ethics Committee (contact via XXX) for researchers who meet the criteria for access to confidential data.*<br><br>*The data underlying the results presented in the study are available from (include the name of the third party*	All relevant data are within the manuscript and its Supporting Information files.

and contact information or URL).<br>• This text is appropriate if the data are owned by a third party and authors do not have permission to share the data.<br><br>* typeset	
Additional data availability information:	

Manuscript

Click here to access/download;Manuscript;EFFECT OF EMS ON TRAITS IN COWPEA.docx

Effect Of Ethyl Methane Sulfonate Mutagenesis on Phenological, Yield-Related and Yield Traits In Cowpea *(Vigna unguiculata* (L.) Walp)

Henry .K. Mensah[1¶*], Ralieva A.K. Norety[1¶] , Isaac .K. Asante[1&], Felicia Oppong[1&]

[1]Department of Plant and Environmental Biology, University of Ghana, Accra, Ghana

*Corresponding author

henryquofi@gmail.com (HKM)

.

[¶] These authors contributed equally to this work.

[&]These authors also contributed equally to this work.

Abstract

This study investigated the impact of ethyl methane sulfonate (EMS) on the M_1 generation of cowpea (*Vigna unguiculata* (L.) Walp). Seventy-five seeds of '*Wang Kae*' cowpea variety were soaked in water for six hours and later subjected to three different doses of ethyl methane sulfonate (20 mM, 40 mM and 80 mM) with fifty serving as a control. Phenological, yield-related, and yield-specific data were collected in accordance with the International Board for Plant Genetic Resources cowpea descriptor. Data analysis was performed using Jamovi 2.7.15 and Jasp 0.95.4.0 to compute one-way ANOVA and post hoc contrast, principal component biplot, and cluster analyses. No optimal EMS concentration (LD50) for inducing mutations was identified. The maximum seed germination rate of 89.33% and seedling survival rate of 74.67% were recorded at 80 mM, whereas the control group exhibited the lowest rates, with 70.00% germination and 62.00% survival. The trend observed for both seed germination and seedling survival rates was: 0 mM < 20 mM < 40 mM < 80 mM. Significant differences ($P<0.05$) in all phenological traits, pod length, locule number, seed traits, and yield per plant were found between the control and treatments. Yield was significantly higher ($P = 0.047$) at 20 mM, with a mean of 61.19 ± 3.34 g, compared to the control. Contrast analysis identified genotypes B33 and D56 as the most productive mutants, yielding 125.44 g and 111.85 g, respectively. Principal component analysis produced eighteen components, with the first four accounting for 50.60% of total variation. Biplot analysis revealed that PC1 and PC2 encompassed all phenological traits, key seed traits, and yield attributes, especially for genotypes B33 and D56. Cluster analysis divided the 190 genotypes into six groups, with B33 and D56 constituting distinct clusters 4 and 1, respectively. In conclusion, the EMS mutagenesis was effective in inducing the unique variations that will be useful for breeding and development of new farmer preferred varieties.

Introduction

Cowpea (*Vigna unguiculata)* is an essential legume that is commonly grown in the developing countries of the tropics and sub-tropics especially in Sub-Saharan Africa, Asia, Central, and South America, and in some temperate areas like the Mediterranean region and the Southern states of the USA.

Due to a drop in the market value of cowpeas due to pests, pathogens, and a lack of genetic variability, many scientists around the world have attempted to develop cowpea varieties to fulfil the taste and demand of a growing population [1-3]. The most noteworthy studies have used mutagens to try to boost genetic variability [1-7]. Ethyl Methane Sulfonate (EMS), one of the most effective mutagens, has been successfully used by several researchers in West Africa to improve genetic variability in cowpea [3, 8].

Mutation is a heritable alteration in the structure of a gene that occurs suddenly in an organism. It is caused by a change in the base sequence of genes, and it can happen naturally or intentionally in both seed and vegetatively propagated plants [9, 10].

To create useful mutants in various crop species, a variety of chemical mutagens have been used. The artificial induction of mutation by colchicine (Col), ethyl methane sulphonates (EMS), and sodium azide (SA) provides a tool to overcome the limitations of variability in plants, particularly Carnation (*Dianthus caryophyllus*)., that induces specific improvement without disturbing their better attributes, according to the various researchers [6]. The present study was undertaken to test the efficacy of EMS in inducing variability in phenological, yield-related and yield characters of cowpea for the selection of superior genotypes for these important traits.

Materials & Methods

Experimental site, material and design

The field study was carried out at the Department of Plant and Environmental Biology, University of Ghana early April to July 2024. Seventy-five seeds of '*Wang Kae*' cowpea variety (M_0 generation) were soaked in water for six hours and later subjected to three different doses of ethyl methane sulfonate (20 mM, 40 mM and 80 mM) with 50 serving as a control. Genotypes were labelled according to EMS treatment dose. Genotypes exposed to 0 mM EMS were designated A1–A31 , those treated with 20 mM EMS were labelled B1–B48, genotypes subjected to 40 mM EMS were designated C1–C55 , and those treated with 80 mM EMS were labelled D1–D56. The treated seeds were thoroughly washed in running water for 30 minutes to reduce the residual effect of the mutagen sticking to the seed coat. The treated seeds together with the control were planted to raise the M_1 generation and standard cultural practices were carried out thereafter. The experiment was designed in pots with dimension 3.29 m^2. Spacing used was 0.8m between lines and 0.5 m between rows. One cowpea seed were planted per hole in each pot at a depth 3 cm to 4 cm.

Parameters studied in M_1 generation

The growth, yield, and yield-related parameters studied in the M1 generation were grouped as phenological: days to germination (*dtg*), days to first flower (*dff*), days to 50% flowering (d50f), days to first harvest (*dfh*), days to 50% mature pod (*d50mp*); and yield-related traits: peduncle length at first harvest (*pel*), number of pods per peduncle (*npp*), number of pods per plant at maturity (*nptm*), number of seeds per pod (*nsp*), number of seeds per plant (*nspt*), pod length (*pdl*), pod width (*pdw*), number of locules (*ln*), percent seed abortion (*psa*), seed length (*sdl*), seed width (*sdw*), seed thickness (*sdt*), seed weight (*swg*), and yield per plant (*yld*).

Percentage seed abortion was calculated as;

$$\frac{[(number\ of\ locules - number\ of\ seed\ per\ pod)]}{number\ of\ locules}\ x\ 100\% \ldots\ldots 1$$

Yield (g) was calculated as; $\frac{100\ seed\ weight\ (g)\ x\ number\ of\ seeds\ per\ plant}{100} \ldots\ldots 2$

Statistical analysis

Germination and survival rate

Germination of seeds was recorded 7 days after planting. Survival plants were recorded at 50% flowering and germination and survival rate were recorded using the formula by [11]

$$Germination\ rate\ (\%) = \frac{(number\ of\ germinated\ seeds)}{(number\ of\ (un)treated\ seeds\ planted)}\ x\ 100 \ldots\ldots 3$$

$$Survival\ rate\ (\%) = \frac{(number\ of\ survived\ seedlings)}{(number\ of\ germinated\ seeds)}\ x\ 100 \ldots\ldots 4$$

[11]

XLSTAT was also used to generate the linear regression equation of seedling germination and survival rate.

Distribution and post hoc contrast analysis for the selection of putative traits and mutants'

Descriptive statistics, one-way ANOVA and post hoc contrast analysis were analyzed for each trait to compare differences between treatments using Jamovi 2.7.15.0 software. Plants within treatments whose values fell outside the control range for each character and were statistically significant from the control were selected as putative mutants**.**

Multivariate analysis for the verification of the selected putative mutant traits and genotypes

Principal Component Analysis (PCA) was carried out accompanied by biplot analysis using Jamovi 2.7.15.0. Principal Component Analysis for quantitative traits was employed to determine the percentage contribution of each trait to total genetic variation. The principal

components (PCs) with eigenvalues >1 was selected, and traits that had coefficients >0.3 were considered to have significantly contributed to variability. The biplot plotted computed similarity values to show the relationship among the traits for each treatment and genotypes. Cluster analyses were performed using Jamovi 2.7.15.0 was to group genotypes within treatments using the method of Ward.D2 hierarchical clustering optimization criterion. The circular dendrogram plotted from the computed similarity values showed the relationship among the genotypes and traits that showed significant difference among treatments.

Results and discussion

Percentage germination and survival of M_1 plants

The results for seed germination and survival rates (%) and linear regression fits for M_1 cowpea genotypes subjected to three EMS doses for 6 h are presented in Fig 1. The highest seed germination rate (89.33%) and seedling survival rate (74.67%) were recorded at 80 mM, whereas the lowest germination rate (70.00%) and seedling survival rate (62.00%) were observed in the 0 mM (control) treatment. The percentage seed germination and seedlings survival were in this order; 0 mM < 20 mM < 40 mM < 80 mM.

Linear regression analysis revealed a strong positive relationship between EMS concentration and both seed germination and survival rate (Fig 1). For seed germination rate, the regression equation was $y = 6.4667x + 65.333$ with an R^2 value of 0.9395 Seedling survival showed a positive linear relationship with EMS concentration as follows:

$y = 4.7333x + 56.667$ with an R^2 value of 0.9042.

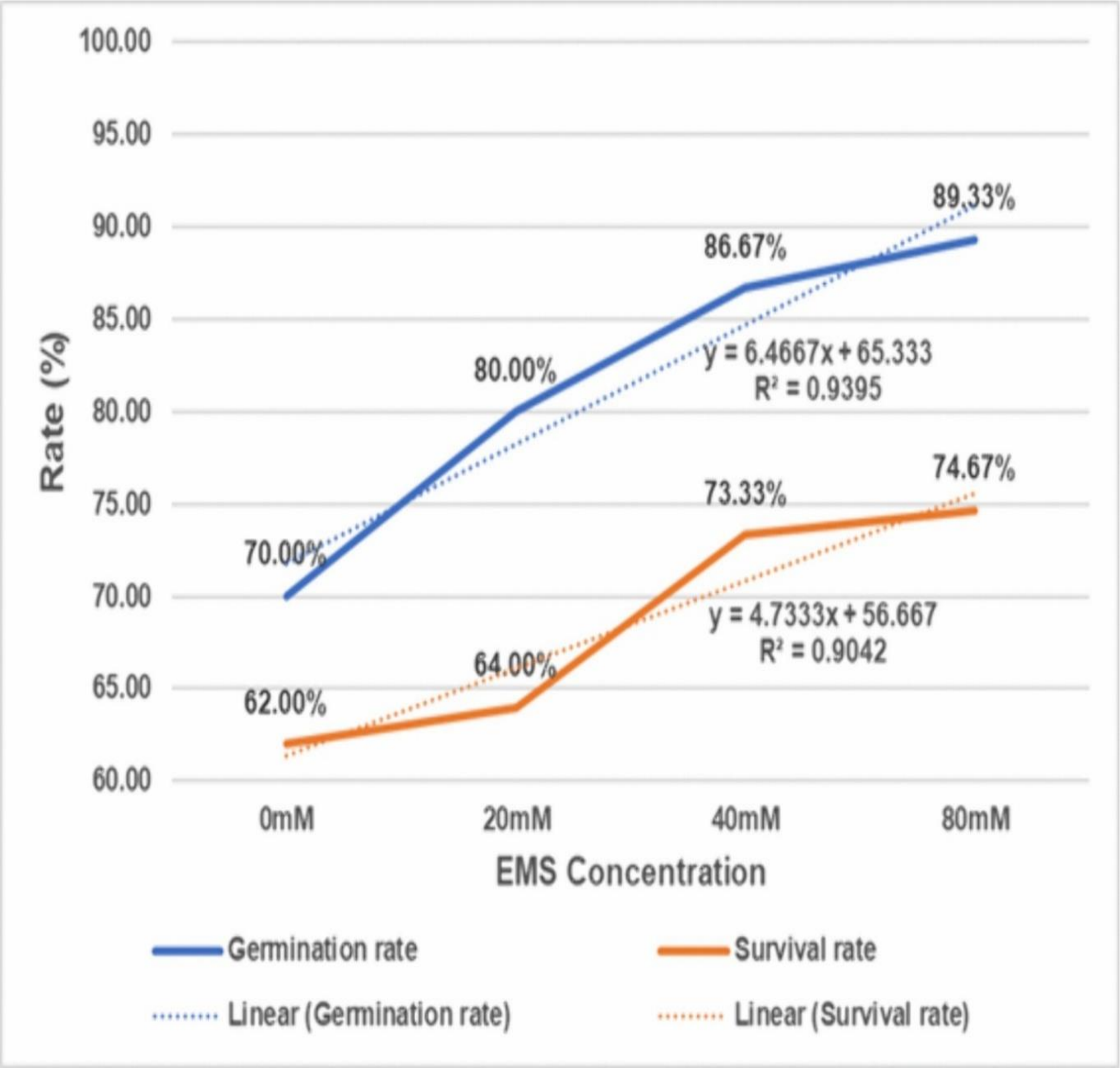

Fig 1: Seed germination and survival rates (%) and linear regression fits for M_1 cowpea genotypes subjected to three ethyl methanesulfonate (EMS) doses for 6 h.

In the present study LD_{50} was not achieved, as seed germination and seedling survival increased with increasing EMS concentration. This response differs from the findings of [9, 12- 14]. [14], who reported a clear dose-dependent reduction in germination and survival in the M_1 generation of cowpea varieties *Pusa Komal* and *V-240 (Rambha)* following 0.1–0.3% EMS treatments, with control germination exceeding 95% and higher doses approaching the LD_{50} threshold. Similarly, [8] observed a strong negative linear relationship between EMS concentration and germination in cowpea and identified an optimal LD_{50} dose at 0.4% EMS.

The contrast observed in this current study where germination and survival were lowest in the control and increased with EMS concentration indicate that LD_{50} expression is highly dependent on genotype, EMS dose range, and exposure duration. Unlike previous studies that employed percentage-based EMS concentrations and different treatment protocols, the present study used millimolar EMS concentrations with a fixed 6-h exposure, which may have altered the physiological response. In addition, the relatively low germination and survival recorded in the control suggest baseline establishment constraints that may have obscured EMS-induced lethality. These findings indicate that the absence of an LD_{50} endpoint in the present study does not imply insufficient mutagenic effect. Rather, it underscores the importance of genotype × dose × exposure-time interactions in EMS mutagenesis.

Contrast analysis of traits in M_1 generation

Phenological traits

Days to germination (*dtg*) differed significantly ($P < 0.001$) among treatments. The control (0 mM) germinated earliest, with a mean of 3.45 ± 0.13 days and a range of 2–5 days. All EMS treatments delayed germination, with the strongest effect at 20 mM, while 40 and 80 mM showed delayed but comparable responses. The frequency of putative mutants was 50.0% at 20 mM, 29.10% at 40 mM, and 30.36% at 80 mM (Table 1). Selected putative mutants can be found in S1 Table.

Days to first flower (*dff*) were significantly ($P < 0.001$) affected by EMS among treatments. *Dff* was earliest in the control, with a mean of 37.87 ± 0.77 days and values ranged from 30 to 45 days, and was progressively delayed with increasing EMS concentration, reaching a maximum mean of 45.04 ± 0.86 days at 40 mM. Mutant frequencies declined from 51.17% at 20 mM to 41.07% at 80 mM (Table 1). Selected putative mutants can be found in S1 Table.

Table 1: Mean, variability, mutant frequency, and pairwise significance of phenological traits in the M_1 generation of cowpea under different EMS concentrations

Trait	Trt	Mean±se	%CV	Min	Max	Freq	P	Significant pairwise comparisons			
								0mM	20mM	40mM	80mM
dtg	0mM	3.45±0.13$_a$	20.90	2	5		<0.001		<0.001	<0.001	<0.001
	20mM	5.38±0.20$_b$	25.30	3	7	50.00		<0.001			
	40mM	4.78±0.19$_b$	29.20	3	7	29.10		<0.001			
	80mM	4.77±0.19$_b$	29.70	3	7	30.36		<0.001			
dff	0mM	37.87±0.77$_a$	11.30	30	45		<0.001		<0.001	<0.001	<0.001
	20mM	44.69±0.91$_b$	14.10	33	54	51.17		<0.001			
	40mM	45.04±0.86$_b$	14.10	34	55	47.27		<0.001			
	80mM	44.46±0.79$_b$	13.30	33	55	41.07		<0.001			
d50f	0mM	57.84±0.92$_a$	8.90	48	68		<0.001		<0.001	<0.001	<0.001
	20mM	64.67±0.99$_b$	10.70	50	75	37.50		<0.001			
	40mM	65.18±0.97$_b$	11.00	49	80	40.00		<0.001			
	80mM	64.79±0.85$_b$	9.80	53	80	28.57		<0.001			
dfh	0mM	49.35±1.15$_a$	13.00	40	67		<0.001				
	20mM	60.17±0.94$_b$	10.80	48	72	18.75		<0.001			
	40mM	58.93±0.97$_b$	12.10	38	72	9.100		<0.001			
	80mM	58.50±0.91$_b$	11.70	43	72	12.50		<0.001			
d50mp	0mM	79.48±1.41$_a$	9.90	66	94		<0.001		<0.001	<0.001	<0.001
	20mM	90.17±1.37$_b$	10.50	68	107	39.58		<0.001			
	40mM	88.65±1.09$_b$	9.10	68	106	25.45		<0.001			
	80mM	88.32±1.16$_b$	9.80	69	110	23.21		<0.001			

Note. Means sharing the same superscript letter within a trait are not significantly different according to Tukey's HSD test ($P \leq 0.05$). Trt= Treatment, Mean ± SE = Mean plus or minus standard error, %CV= Percentage coefficient of variation, Min= minimum, Max, maximum Freq= Percentage frequency of putative mutants; P=Probability value.Traits: dtg = days to germination, dff = days to first flower, d50f = days to 50% flowering, dfh = days to first harvest, d50mp = days to 50% mature pods

Days to 50% flowering (***d50f***) were significantly ($P < 0.001$) affected by EMS among treatments. ***D50f*** increased from a control mean of 57.84 ± 0.92 days, with values ranging from 48 to 68 days, to mean values of approximately 65 days across EMS treatments, with broader ranges of 49–80 days. Corresponding mutant frequencies were 37.50%, 40.00%, and 28.57% at 20, 40, and 80 mM, respectively (Table 1). Selected putative mutants can be found in S1 Table.

Days to first harvest *(dfh)* was earliest in the control, with a mean of 49.35 ± 1.15 days and a range of 40–67 days and was significantly ($P < 0.001$) delayed in all EMS treatments. Mean dfh increased to 60.17 ± 0.94 days at 20 mM, 58.93 ± 0.97 days at 40 mM, and 58.50 ± 0.91 days at 80 mM. Mutant frequencies were 18.75% at 20 mM, 9.10% at 40 mM, and 12.50% at 80 mM (Table 1). Selected putative mutants can be found in S1 Table.

A similar trend was observed for **days to 50% maturity of pods (*d50mp*)**, where the control recorded the earliest maturity of 79.48 ± 1.41 days, with values from 66 to 94 days. EMS treatments significantly (P<0.001) delayed in pod maturity from the control, with mean ***d50mp*** values of 90.17 ± 1.37 days at 20 mM, 88.65 ± 1.09 days at 40 mM, and 88.32 ± 1.16 days at 80 mM. The frequency of selected mutants for d50mp was 39.58%, 25.45%, and 23.21% at 20, 40, and 80 mM respectively (Table 1). Selected putative mutants can be found in S1 Table.

In our study EMS treatments affected the timing of developmental events of flowering and maturity in cowpea. Days to first flowering and days to 50% flowering tended to be delayed in many high-dose EMS mutants, although some early-flowering outliers were observed at lower doses. The delay in flowering under stronger mutagenesis is a common phenomenon, often attributed to physiological stress or disruption of genes controlling flowering time [15-18]. [14] did not directly measure flowering time, but their M_1 mutants showed overall physiological delays, and other researchers have explicitly noted flowering extensions. For example, [19] working in mung bean reported that higher EMS levels prolonged days to flowering and maturity, likely due to mutational interference in developmental pathways. It is also recorded by [20] in black gram, [21] in *Gloriosa superben* and [5, 22] in *Vigna radiata*. In cowpea, [23] interestingly observed an *earlier* onset of flowering, a decrease in days to 50% flowering in an EMS-treated yardlong bean (*Vigna sesquipedalis*) population, accompanied by reduced maturity duration.

Such wide variation in flowering time due to EMS has also been reported by [8], who found that their M_2 cowpea population showed continuous distribution for days to flowering far beyond the range of the wild type. This implies that EMS created both later- and earlier-flowering individuals. Notably, [8] identified dozens of mutants that flowered significantly

(P<0.05) earlier than the parent variety, reflecting new earliness traits. These results suggest that certain mutations can accelerate flowering, potentially by knocking out repressors of flowering even as overall vigor is reduced. In our mutants, while the predominant trend at high EMS dosages was delayed flowering and maturity, a few early blooming mutants from lower EMS treatments were recorded, indicating increased phenological variation.

Our results confirm that EMS mutagenesis increased flowering time variance. While early flowering is advantageous for drought avoidance and multi-cropping systems, our evaluation of the EMS population did not yield any early-flowering mutants; rather, such occurrences were observed in the control group. This outcome aligns with results previously reported in mung bean and other pulse crops.

Yield-related and yield traits

The **pod length (*pdl*)** was highest in the control (0 mM), with a mean of 14.65 ± 0.31 cm and a range of 11.55–17.90 cm whereas 80mM recorded the lowest mean ***pdl*** of 13.52±0.28 cm which was the significantly (P=0.008) different from the control. Based on deviations from the control range, the frequency of selected mutants for ***pdl*** was 10.42, 9.10 and 21.43% in 20 mM, 40 mM and 80 mM respectively (Table 1). Selected putative mutants can be found in S1 Table.

The ***number of locules per pod (ln)*** differed significantly among EMS treatments (P < 0.001). The control recorded a mean of 17.00 ± 0.60, with values ranging from 12 to 24 locules. Higher EMS concentrations resulted in a significant reduction in locule number for 40 mM at 14.93 ± 0.48 locules and further to 11.41 ± 0.39 locules at 80 mM. Pairwise comparisons showed that only 80 mM treatment differed significantly (P < 0.001). from both the control and all other treatments, Phenotypic variability increased with EMS dose, as reflected by the rise in coefficient of variation from 17.5% in 20 mM to 25.8% at 80 mM. Based on deviations

Table 2: Mean, variability, mutant frequency, and pairwise significance of yield-related and yield traits in the M_1 generation of cowpea under different EMS concentrations

Trait	Trt	Mean±se	%CV	Min	Max	Freq	P	Significant pairwise comparisons			
								0mM	20mM	40mM	80mM
npp	0mM	3.84±0.17	25.30	2	5		0.447				
	20mM	4.02±0.13	21.50	2	5						
	40mM	3.82±0.13	25.70	2	5						
	80mM	3.70±0.16	31.80	2	5						
nptm	0mM	24.68±1.31	29.50	14	44		0.412				
	20mM	25.46±1.07	29.10	10	45						
	40mM	23.25±0.78	25.00	11	44						
	80mM	23.87±0.97	30.40	10	46						
nsp	0mM	9.84±0.63	35.60	5	15		0.730				
	20mM	9.92±0.46	32.30	5	15						
	40mM	10.44±0.39	27.80	5	15						
	80mM	9.80±0.46	35.10	5	15						
nspt	0mM	233.10±14.38	34.30	100	406		0.689				
	20mM	246.29±13.29	37.40	100	450						
	40mM	238.20±10.34	32.20	100	435						
	80mM	226.36±12.17	40.20	100	450						
pdl	0mM	14.65±0.31b	11.80	11.55	17.86		0.030				0.008
	20mM	14.34±0.25ab	12.30	10.50	19.88	10.42					
	40mM	13.83±0.26ab	14.10	6.95	16.75	9.10					
	80mM	13.52±0.28a	15.20	8.45	17.50	21.43		0.008			
pdw	0mM	0.85±0.01	9.70	0.70	1.03		0.144				
	20mM	0.89±0.02	16.50	0.61	1.23						
	40mM	0.90±0.03	25.60	0.52	1.28						
	80mM	0.83±0.02	18.00	0.60	1.13						
ln	0mM	17.00±0.60c	19.70	12.00	24.00		<0.001			0.023	<0.001
	20mM	17.06±0.43c	17.50	12.00	23.00					0.005	<0.001
	40mM	14.93±0.48b	23.90	8.00	21.00	20.00		0.023	0.005		<0.001
	80mM	11.41±0.39a	25.80	7.00	23.00	50.00		<0.001	<0.001	<0.001	
psa	0mM	14.27±1.93	75.30	0.00	35.71						
	20mM	18.60±1.68	62.70	0.00	35.71						
	40mM	19.35±1.66	63.80	0.00	35.71						
	80mM	15.43±1.53	74.20	0.00	35.71						
sdl	0mM	7.44±0.24a	18.10	5.68	11.59		<0.001		<0.001	<0.001	<0.001
	20mM	8.77±0.25b	19.60	6.10	11.65	0.42		<0.001		<0.001	<0.001
	40mM	9.79±0.07c	4.90	8.10	11.05			<0.001	<0.001		
	80mM	9.95±0.09c	6.60	7.95	11.60	1.79		<0.001	<0.001		
sdw	0mM	4.78±0.10a	11.90	3.94	6.08		<0.001		<0.001	<0.001	<0.001
	20mM	5.14±0.09b	11.80	4.05	6.44	0.42		<0.001			
	40mM	5.14±0.04b	5.90	4.58	5.69			<0.001			
	80mM	5.25±0.05b	7.00	4.28	6.11	1.79		<0.001			
sdt	0mM	4.09±0.07a	9.80	3.29	4.92		<0.001		0.027		
	20mM	4.35±0.08b	12.20	3.31	5.19	12.50		0.027		<0.001	0.005
	40mM	3.95±0.04a	8.50	3.43	5.59	1.82			<0.001		
	80mM	4.08±0.05a	8.90	3.41	4.94	1.79			0.005		

Note. Means sharing the same superscript letter within a trait are not significantly different according to Tukey's HSD test ($P \leq 0.05$). Trt= Treatment, Mean ± SE = Mean plus or minus standard error, %CV= Percentage coefficient of variation, Min= minimum, Max, maximum Freq= Percentage frequency of putative mutants; P=Probability value. Traits: npp = number of pods per peduncle, nptm = number of pods per plant at maturity, nspt = number of seeds per plant, pdl = pod length, pdw = pod width, ln = number of locules, nsp = number of seeds per pod, psa = percent seed abortion, sdl = seed length, sdw = seed width, sdt = seed thickness, swgt = seed weight, yld = yield per plant.

Table 2: Mean, variability, mutant frequency, and pairwise significance of yield-related and yield traits in the M_1 generation of cowpea under different EMS concentration (*cont'd*)

Trait	Trt	Mean±se	%CV	Min	Max	Freq	P	Significant pairwise comparisons			
								**0mM**	**20mM**	**40mM**	**80mM**
swg	0mM	21.03±0.34a	9.10	17.82	24.87		<0.001		<0.001	<0.001	0.020
	20mM	24.86±0.34c	9.40	17.81	29.99	54.17		<0.001			<0.001
	40mM	24.37±0.35c	10.70	17.70	28.39	49.10		<0.001			<0.001
	80mM	22.60±0.33b	11.00	17.35	28.68	16.07		0.020	<0.001	<0.001	
yld	0mM	48.79±3.01a	34.40	20.61	85.68		0.018		0.047		
	20mM	61.19±3.34b	37.80	18.70	125.44	16.67		0.047			
	40mM	58.02±2.59ab	33.10	19.97	105.43	12.72					
	80mM	51.16±2.87ab	42.00	18.45	111.85	10.71					

Note. Means sharing the same superscript letter within a trait are not significantly different according to Tukey's HSD test (P ≤ 0.05). Trt= Treatment, Mean ± SE = Mean plus or minus standard error, %CV= Percentage coefficient of variation, Min= minimum, Max, maximum Freq= Percentage frequency of putative mutants; P=Probability value. Traits: npp = number of pods per peduncle, nptm = number of pods per plant at maturity, nspt = number of seeds per plant, pdl = pod length, pdw = pod width, ln = number of locules, nsp = number of seeds per pod, psa = percent seed abortion, sdl = seed length, sdw = seed width, sdt = seed thickness, swgt = seed weight, yld = yield per plant.

from the control range, the frequency of putative mutants for ***ln*** reached 20.0% at 40 mM and 50.0% at 80 mM (Table 1). Selected putative mutants can be found in S1 Table.

Seed length (*sdl*) varied significantly across EMS treatments (P < 0.001). The control recorded the lowest mean seed length of 7.44 ± 0.24 mm, with values ranging from 5.68 to 11.59 mm and a CV of 18.1%. EMS treatment groups resulted in a progressive increase in seed length, with means of 8.77 ± 0.25 mm at 20 mM, 9.79 ± 0.07 mm at 40 mM, and 9.95 ± 0.09 mm at 80 mM. Pairwise comparisons showed that all EMS treatments differed significantly from the control (P < 0.001), while the 40 mM and 80 mM treatments did not differ from each other. Based on deviations from the control range, mutant frequencies were low for seed length, recorded at 0.42% for 20 mM and 1.79% for 80 mM (Table 1). Selected putative mutants can be found in S1 Table.

Seed width (*sdw*) was also significantly (P < 0.001) affected by EMS treatment. The control population recorded a mean width of 4.78 ± 0.10 mm and ranged from 3.94–6.08 mm with a CV of 11.9%. All EMS treatments differed significantly from the control (P < 0.001).

Variability declined at higher EMS doses, with CV values as low as 5.9% at 40 mM. Frequencies of putative mutants increased with EMS concentration, reaching 0.42% at 20 mM and 1.79% at 80 mM (Table 1). Selected putative mutants can be found in S1 Table.

Seed thickness (*sdt*) differed significantly among treatments (P < 0.001). The control recorded a mean thickness of 4.09 ± 0.07 mm with values ranging from 3.29 to 4.92 mm. Thickness increased significantly (P=0.027) at 20 mM with a mean of 4.36 ± 0.08 mm. Pairwise comparisons indicated significant differences between 20 mM and both 40 mM (P < 0.001) and 80 mM (P = 0.005). Mutant frequencies for seed thickness were highest at 20 mM (12.5%) but remained low at 40 mM (1.82%) and 80 mM (1.79%) (Table 1). Selected putative mutants can be found in S1 Table.

100-Seed weight (*swg*) showed highly significant variation among EMS treatments (, P < 0.001). The control recorded a mean of 21.03 ± 0.34 g and values ranged from 17.82 to 24.87 g. EMS treatment increased seed weight, with the highest means observed at 20 mM with a value of 24.86 ± 0.34 g and 40 mM weighed 24.37 ± 0.35 g, both of which differed significantly from the control (P < 0.001). The 80 mM treatment weighed 22.60 ± 0.33 g and showed an intermediate response and differed significantly from both the control (P = 0.02) and lower EMS doses (P<0.001). Frequencies of putative mutants were highest at 20 mM (54.17%), followed by 40 mM (49.1%) and 80 mM (16.07%) (Table 1). Selected putative mutants can be found in S1 Table.

Yield per plant (*yld*) differed significantly among treatments (P = 0.018). The control population recorded the lowest mean yield of 48.79 ± 3.01 g, with values ranging from 20.61 to 85.68 g. Yield increased significantly (P=0.047) at 20 mM with a mean of 61.19 ± 3.34 g, which differed from the control (P = 0.047). The means of 40 mM, 58.02 ± 2.59 g and 80 mM, 51.16 ± 2.87 g treatments exhibited intermediate yields and did not differ significantly from

either the control or 20 mM. Phenotypic variability was high across treatments with a CV between 33.10 –42%, and mutant frequencies declined with increasing EMS concentration, from 16.67% at 20 mM to 10.71% at 80 mM (Table 1). Selected putative mutants can be found in S1 Table.

The lack of notable mutants for number of pods per peduncle (*npp*), number of pods per plant at maturity (*nptm*), number of seeds per pod (*nsp*) and number of seeds per plant (*nspt*) in the M_1 generation is likely due to these traits being highly polygenic and developmentally buffered, which restricts observable changes [24- 26]. Yield components may mask mutational effects through compensatory relationships [27, 28]. However, significant mutants were identified for these traits in M_2 populations, where recessive mutations are revealed and chimerism is resolved [8, 29- 31]. Moreover, higher EMS doses often reduce pod and seed numbers due to partial sterility or lower pollen viability, as seen in legumes [14, 20, 32].

In the present study, several putative mutants from the 20 mM, 40 mM, and 80 mM EMS treatments exhibited significantly increased pod length, seed dimensions, seed weight, and yield per plant relative to the control. Comparable yield-enhancing mutations have been reported in earlier cowpea studies. For example, [8] identified M_2 cowpea mutants with significantly higher pods per plant, seeds per plant, seeds per pod, and pod length than the wild type. Those authors further noted that increased pod number, seed weight, and plant stature have frequently been achieved through EMS mutagenesis in cowpea.

Seed size traits also varied widely, with some mutants significantly affected with longer and wider seeds in the present study. Increased seed size due to mutagenesis has been reported previously; [33] isolated bold-seeded mutants in *Vigna mungo* with enhanced yield. In our study, high-yielding mutants, for example B09 typically combined larger seeds with and high

seed weight, a favorable combination for yield improvement. Similarly, [33] and [34] reported cowpea mutants with enlarged seeds and improved yield performance, consistent with the superior lines identified in the present study.

Yield stimulation following mutagenic seed treatments has also been documented in other legumes. Increased yield or yield components after mutagen exposure have been reported in pigeonpea [35], mung bean [36], and mung bean treated with gamma rays, EMS, and epichlorohydrin [37]. These studies collectively support the notion that induced mutagenesis can unlock favorable allelic variation controlling yield traits.

In contrast, many mutants particularly from the highest EMS dose (80 mM) exhibited reductions in yield and its components, including pod length, number of locules, seed size and seed weight. Such negative effects of high mutagen doses are well documented. [38] in okra and [39] in petunia reported that mutagenesis frequently produces lines with reduced reproductive output. In cowpea, [40] observed that EMS doses beyond the optimal range led to sharp declines in pollen fertility and seed yield, while [41] reported positive yield shifts at lower mutagen doses and negative shifts at higher doses. Our results closely mirror this dose-dependent pattern. Yield depression at elevated doses of irradiation or chemical mutagens has been widely reported. Similar declines were observed by [20] in black gram, [21] in *Gloriosa* Khan *superba* under EMS and DES treatments, [42] in *Vigna mungo*, and [43] in *Vigna radiata*.

These findings reflect the accumulation of deleterious mutations at higher mutagen doses, which can impair physiological and reproductive processes. Conversely, [32] reported stimulation of seed weight in pigeon pea under combined mutagenic treatments, suggesting that moderate doses may enhance specific yield components. Similar observations were reported by [44], [45], and Patil and [46] across various pulse crops, reinforcing the dose- and trait-dependent nature of mutagenic responses.

In the present study, one elite mutant (B33) from the 20 mM EMS treatment produced approximately 25% higher seed yield than the control, accompanied by a modest increase in 100-seed weight. This indicates that EMS successfully generated yield-enhancing alleles. [47] similarly reported EMS-derived cowpea lines with significantly increased pod number, confirming the potential of chemical mutagenesis to improve yield potential.

It is also noteworthy that the frequency of desirable mutants was low, consistent with previous reports. [8] estimated that only 0.1–1% of mutants exhibited significantly improved yield related and yield traits. Similarly, although a few promising mutants were identified in the present study, the majority performed at or below the level of the control. This underscores the necessity of screening large mutant populations to capture rare beneficial events.

Multivariate Analysis: PCA Biplot and Cluster Grouping

PCA Biplot of phenological and yield-related and yield traits

Principal component analysis (PCA) performed on the traits and genotypes plant observations across EMS treatments generated a total of 8 principal components with an eigenvalue < 1 (S2 Table). The first four principal components accounted for a total variability greater than 50.60% in all treatments. The first two dimensions/principal components (Dim1 and Dim2) together explained 32.8% of the total phenotypic variation, with PC1 accounting for 17.7% and PC2 accounting for 15.1% of the variation (Fig 2,S2 Table). PC1 represented major seed and phenological traits, including seed length (*sdl*), seed width (*sdw*) days to first flower (*dff*), days to 50% flowering (*d50f*), days to first harvest (*dfh*), days to 50% pod maturity (*d50mp*) and *swg* loaded positively on PC1, indicating that higher PC1 scores were associated with delayed development. On the other hand, yield-related traits such as number of seeds per plant (*nspt*), number of seeds per pod (*nsp*), and yield (*yld*) were oriented in the opposite direction on PC2 reflecting an inverse relationship between prolonged phenology and yield-related and yield traits.

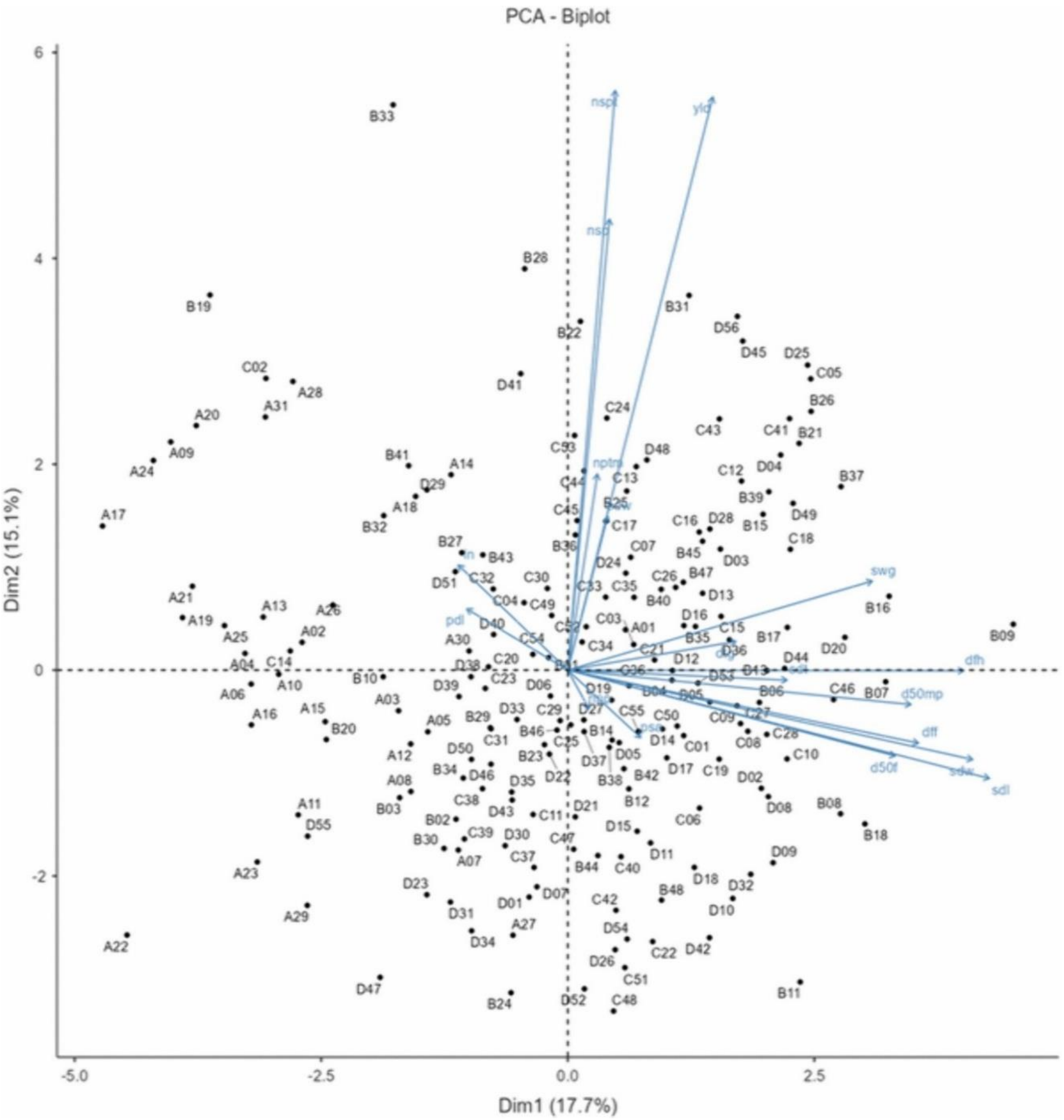

Fig 2: PCA biplot of cowpea genotypes and traits based on PC1 and PC2. Genotypes treated with 0-, 20-, 40- and 80-mM EMS are labelled A01–A31, B01–B48, C01–C55 and D01–D56, respectively. Vectors indicate trait loadings and points represent genotype distribution. Percent variance explained by each PC is shown on the axes. Key: dtg days to germination, dff = days to first flower, d50f = days to 50% flowering, dfh = days to first harvest, d50mp = days to 50% mature pods, npp = number of pods per peduncle, nptm = number of pods per plant at maturity, nspt = number of seeds per plant, pdl = pod length, pdw = pod width, ln = number of locules, nsp = number of seeds per pod, psa = percent seed abortion, sdl = seed length, sdw = seed width, sdt = seed thickness, swgt = seed weight, yld = yield per plant

The distribution of genotypes plants on the PCA biplot revealed most control plants (0 mM) formed a compact cluster near the negative side of PC1, reflecting early phenology, uniform development, and moderate highly yield output. Plants from the 20 mM EMS treatment showed partial overlap with the control group but were moderately displaced toward positive PC1 values, indicating mild delays in phenological development and moderate reductions in yield components. Genotypes from the 40 mM and 80 mM EMS treatments were progressively displaced toward the positive side of PC1 and exhibited greater dispersion along both PC1 and PC2.This shift reflects dose-dependent delays in flowering, harvest, and maturity, coupled with reductions in reproductive traits such as *nsp*, *nspt* and *yld*.

Notably, vectors for seed length (*sdl*) and seed width (*sdw*) were oriented opposite to those for number of seed per pod and plant and yield. Genotypes positioned along the *sdl*/*sdw* vectors from the 40 mM and 80 mM treatments were characterized by larger seed size but reduced yield, suggesting altered resource allocation under EMS-induced mutational stress.

Selection focused on genotypes aligned with key trait vectors representing yield performance, seed size, and phenological timing. High-yielding putative mutants were identified among genotypes positioned in the upper region of the biplot with positive PC2 scores, in close alignment with the ***yld***, ***nsp***, ***nptm***, and ***swg*** vectors. These included B33, B28, B22, B31, D56, D45, D25, C5, C41, B26, and B21, representing EMS-derived plants with enhanced reproductive output relative to the population mean.

Large-seed putative mutants were selected from genotypes located along the negative PC2 axis, in the direction of the sdl and sdw vectors. Representative genotypes included D23, D7, D34, D47, C37, C39, C40, C42, B18, B11, and B8, which were characterized by increased seed size but reduced seed number and yield.

Early-phenology putative mutants were identified from the extreme negative side of PC1, opposite to the late-flowering and maturity vectors. These included B19 and C2, reflecting genotypes with accelerated development and earlier flowering and maturity.

Genotypes exhibiting extreme positive PC1 scores with weak alignment to yield vectors such as B9, B7, C46, and D20 were classified as severely delayed and low-performing and were not considered suitable for advancement but retained as extreme phenotypes for genetic and physiological reference.

The PCA biplot in our study was especially informative, as it highlighted which traits drove most variation among the EMS-treated lines. Notably, yield-related (*npp, nsp* and *swg)* and yield traits loaded strongly on the first two principal components, indicating these had the most influence in differentiating mutants. Traits with minimal impact in PCA were also not highlighted in the clustering. By focusing on the PCA, we found a clear separation between high-yielding mutants and low-yielding ones along PC high-yield lines clustered in the quadrant associated with greater pod and seed numbers. In contrast, mutants with delayed phenology or poor fertility tended to lie on the opposite end of the PCA axes, separated from the productive genotypes. This reflects the underlying trade-offs induced by EMS: lines that suffered heavy damage for example dwarf and sterile mutants clustered away from those that managed to improve or maintain yield traits. The utility of PCA in such mutation studies is well recognized; it helps distill many correlated traits into composite axes of variation.

[48], for instance, used multi-trait analysis to identify superior M_2 individuals and reported that mutants with extreme high values in key traits such as seeds per plant could be distinguished from the population distribution. Our PCA biplot offers similar insights by visually highlighting the direction of trait enhancement, such as higher yield, and

demonstrating that the notable mutants distinctly deviated from the control group's path in multivariate analysis.

Cluster analysis of phenological and yield-related and yield traits

Hierarchical cluster analysis based on phenological, yield-related, and yield traits grouped the cowpea M_2 population into six distinct clusters (Fig 3, S1 Table). Cluster 1 comprised predominantly EMS-derived genotypes, representing 35.42%, 49.10%, and 33.93% of the 20 mM, 40 mM, and 80 mM treatment groups, respectively. Only a single control genotype (A01) clustered within this group, accounting for 3.22% of the 0 mM population. Genotypes in this cluster were characterized by prolonged phenological development coupled with superior yield performance. Days to 50% flowering (*d50f*) ranged from 53 to 80 days, with the most pronounced delays observed in C43 and C41, while days to 50% pod maturity (*d50mp*) ranged from 69 to 109 days, recorded in D43 and C08 respectively.

Despite delayed development, individuals in this cluster exhibited markedly higher seed weight (*swg*), ranging from 18.82 g (D04) to 28.68 g (D56), and elevated yield per plant (*yld),* which ranged from 31.45 g (C37) to 111.85 g (D56), exceeding the control population. Selected putative mutants from Cluster 1 included D56, D45, D25, C41, C5, B31, B28, and B22, all of which showed clear positive deviation from the control range for major yield and yield-related traits.

Cluster 2 was dominated by control genotypes, with 61.29% of the 0 mM population grouped within this cluster. Only 2.08%, 3.64%, and 3.57% of the 20 mM, 40 mM, and 80 mM treatment groups, respectively, were represented in this cluster. Genotypes in Cluster 2 exhibited early germination, flowering, and maturity, with yield levels comparable to the control mean. This cluster therefore represented the baseline phenotypic profile of the population and showed low within-cluster variability. A small number of EMS-treated genotypes within this cluster combined early days to first harvest with improved yield, notably C02 (*38 days*) and C14 (*39 days*), which were selected as early-phenology putative mutants.

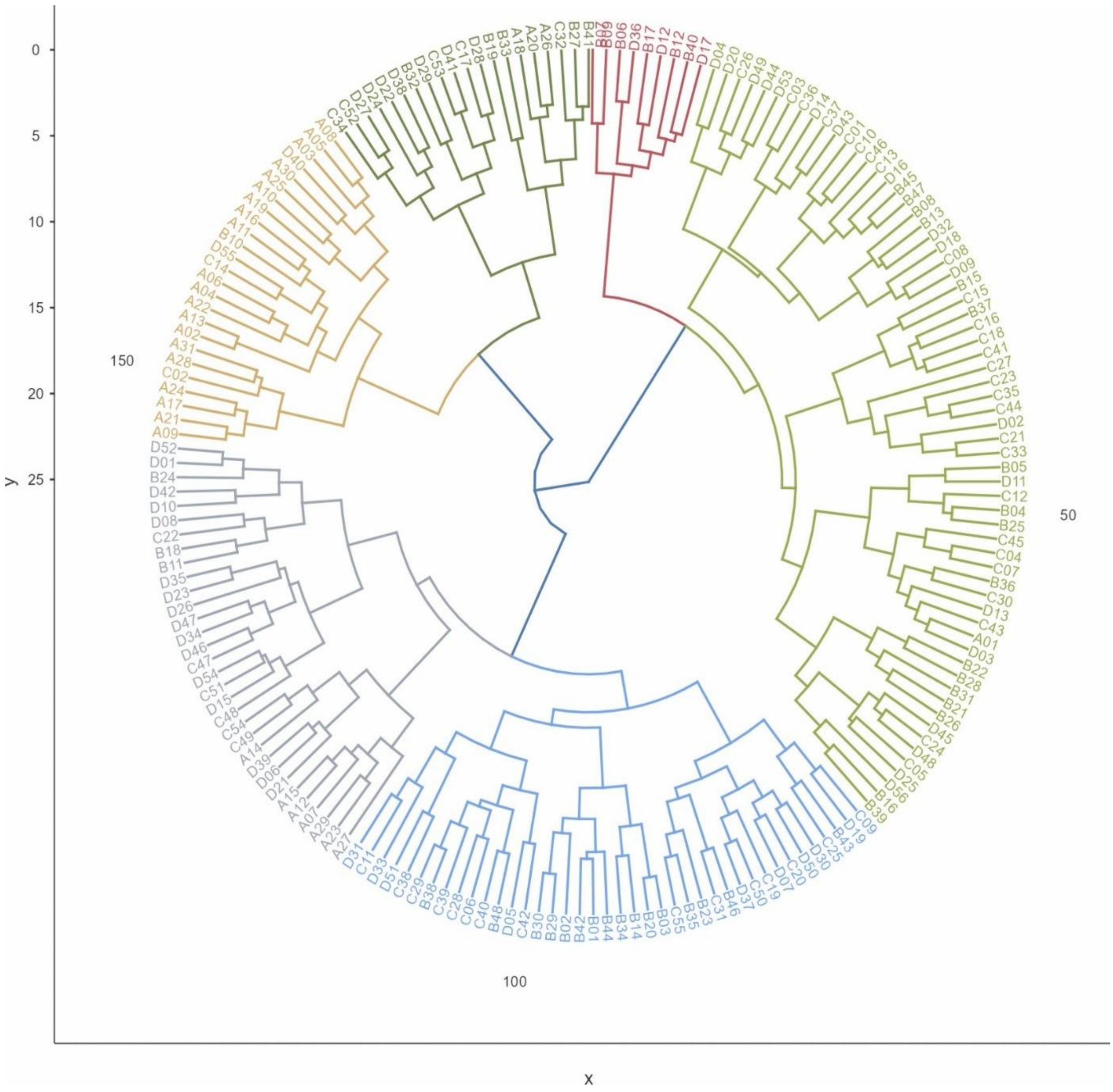

Fig 3: Dendrogram of hierarchical cluster analysis based on quantitative traits using Ward's linkage method showing the genetic relationships among genotypes in the M_1 generation based on average distance. Key: A = 0mM, B = 20mM, C = 60mM and D = 80mM, Green = Cluster 1 [64 genotypes], Brown = Cluster 2 [24 genotypes], Grey = Cluster 3 [31 genotypes], Forest green = Cluster 4 [20 genotypes], Blue = Cluster 5 [40 genotypes], Red = Cluster 6 [9 genotypes]

Cluster 3 comprised mainly high-dose EMS-derived genotypes, with 28.57% of the 80 mM population grouped within this cluster. It was defined by delayed phenological traits, reduced number of locules, and poor yield performance. Representative genotypes such as B7, B9, C46, and D20 showed marked negative deviation from the control and were classified as low-value mutants, not considered for advancement.

Cluster 4 contained genotypes that combined early days to first flower (*dff*) and days to 50% flowering (*d50f*) with extended days to first harvest (*dfh*) and days to 50% mature pod (*d50mp*), resulting in high seed weight and elevated yield, particularly within the 20 mM EMS population. This cluster included 10.42%, 9.09%, and 12.50% of individuals from the 20 mM, 40 mM, and 80 mM treatments, respectively, indicating that favorable phenotypes arose across multiple EMS doses. Notable high-performing putative mutants in this cluster included B19 (*93.59 g*) and B33 (*125.44 g*), both of which substantially exceeded the control in yield while maintaining relatively early phenology.

Cluster 5 was composed exclusively of EMS-treated genotypes, representing 33.33%, 22.27%, and 16.07% of the 20 mM, 40 mM, and 80 mM treatment groups, respectively. These individuals exhibited moderately delayed phenological traits and high seed weight, but suffered from a reduced number of locules, resulting in inferior yield performance relative to the control.

Cluster 6 comprised a small but distinct group of EMS-derived genotypes, including 12.50% and 5.36% of the 20 mM and 80 mM populations, respectively. These genotypes were characterized by moderately delayed phenological traits, large seed size, and high seed weight. Despite relatively low seed number per pod, total yield per plant was high, driven by seed weight. Selected putative mutants from this cluster included B07 and D34, which consistently outperformed the control for seed weight.

The cluster analysis further allowed us to group the EMS-derived cowpea lines into distinct categories based on overall performance. We identified six clusters, but importantly, not all clusters were equal in agronomic value. The clustering effectively segregated the elite putative mutants those combining multiple favorable traits from average performers and poor performers. Cluster membership was closely associated with EMS treatment levels and trait profiles. For example, Cluster 2 in our results consisted predominantly of untreated control plants and a few low-dose mutants, characterized by moderate phenological and yield trait

values (baseline yield and phenology). In contrast, another cluster (Cluster 5) contained high-yielding mutants mostly from the 20 mM and 40 mM EMS treatments, featuring significantly high seed weight than the control, underscoring that this group contained the desirable phenotypes created by the mutagenesis.

A small cluster (Cluster VI) captured a handful of outlier mutants these included some top-yielding lines with respect to weight as well as unusual genotypes suggesting that while most of the best mutants grouped together, a few unique lines had singular trait combinations. On the other hand, it was observed a cluster 3 composed largely of 80 mM EMS mutants with severely reduced yields and late maturation. This stratification by clustering aligns with expectations from other studies employing grouping strategies on mutant populations.

Although formal cluster analysis is not always reported, researchers often categorize mutants by yield or other indices. For instance, [49] and [50] in mutation breeding of pulses have discussed grouping mutant lines by overall performance, noting that mutants from optimal dose treatments tend to form a cohort of superior types, whereas those from high doses populate the inferior groups. Our results mirror this trend: moderate EMS doses produced the bulk of the high-performance cluster, while the highest dose contributed disproportionately to the low-performance cluster, consistent with the dose–performance relationship reported by [41].

The clustering outcomes in our study also have practical breeding implications. The fact that the best-yielding mutants clustered together means they share a similar multi-trait profile, which could be harnessed as a selection index. We can infer that these elite lines possess a favorable combination of genes or alleles for yield, likely with minimal negative pleiotropic effects on other traits. This is promising for developing mutant lines, as high yield was achieved without major negative consequences. [48] selected the top 4% of their M_2 population as high-yield mutants for further breeding. Likewise, our cluster analysis identified 10.71 to 16.67% of lines that significantly exceeded others in yield per plant.

By cross comparing our elite cluster with the control cluster, we found that the improvements in yield traits were statistically significant, reinforcing that these differences are genetically based and not due to random chance. Previous mutation breeding successes in cowpea and related crops have come from recognizing and stabilizing such superior segregants. For example, [51] developed a high-yielding, large-seeded cowpea mutant variety by isolating an outstanding M_2 plant from a 250 Gy treatment and advancing it through selection. This resonates with our strategy of using multivariate clustering to pinpoint candidates for advancement. Moreover, our cluster analysis revealed that mutants derived from the same EMS dose did not always fall into one cluster indeed, some 20 mM mutants ended up in a poor performance cluster and some 80 mM mutants grouped with better performers. This indicates that while dose influences overall trends, the phenotypic expression of each mutant line is unique and must be evaluated on its own merit. Therefore, the use of clustering and PCA helps ensure that selection is based on actual trait patterns rather than preconceived notions of which treatment “should” yield better mutants.

In summary, the elite mutants isolated such as those in high yield related and yield clusters will be further evaluated in advanced generations. If their performance is stable, they could serve as new breeding germplasm or even be released as improved cowpea varieties, analogous to the mutant lines released in other legumes such as mungbean and chickpea as noted by [49].

Conclusion

This study demonstrates the effectiveness of ethyl methanesulfonate (EMS) in inducing substantial genetic variability in cowpea (*Vigna unguiculata* (L.) Walp), resulting in a broadened phenotypic spectrum for key agronomic, yield, and seed traits. Contrast analysis revealed significant treatment effects and highlighted superior mutant lines, while principal

component analysis (PCA) identified yield and reproductive traits as the primary drivers of phenotypic variation. These findings were consistently supported by cluster analysis, which grouped mutants according to overall agronomic performance. The convergence of these analytical approaches strengthened the selection of putative mutants, including high-yielding (B33, B31, B28, B22, C41, C05, D25, D45, D56), early-phenology (B19), and large-seeded or prolific podding lines (D34, C39, C40).

As the study was confined to the M_1 generation, further evaluation in the M_2 and M_3 generations is required to confirm trait stability, heritability, and breeding value. Complementary molecular analyses will be essential to explain the genetic basis of the observed variation and support marker-assisted selection. Overall, the selected mutants constitute valuable genetic resources for cowpea improvement and have strong potential to contribute to enhanced productivity and global food security.

Acknowledgments

HMK, RANK, IKA, and FO conceived and designed the study. HMK and RANK performed the experiments. HMK analyzed the data and drafted the manuscript. RANK and IKA provided critical revisions. All authors read and approved the final manuscript. We gratefully acknowledge the Department of Plant and Environmental Biology, University of Ghana, Legon, for supporting this research.

References

1. Oladosu Y, Rafii MY, Abdullah N, Hussin G, Ramli A, Rahim HA, et al. Principle and application of plant mutagenesis in crop improvement: A review. Biotechnology and Biotechnological Equipment. 2015;30(1):1–16.
2. Owusu EY, Karikari B, Kusi F, Haruna M, Amoah RA, Attamah P, et al. Genetic variability, heritability and correlation analysis among maturity and yield traits in Cowpea (Vigna unguiculata (L) Walp) in Northern Ghana. Heliyon. 2021;7(9).

3. Bhat TA, Khan AH, Parveen S. Comparative analysis of meiotic abnormalities induced by gamma rays, EMS and MMS in Vicia faba L. Journal of Indian Botanical Society. 2005;84:45–58.

4. Olasupo FO, Ilori CO, Forster BP, Bado S. Mutagenic effects of gamma radiation on eight accessions of cowpea (Vigna unguiculata [L.] Walp.). Am J Plant Sci. 2016;7(2):339–51.

5. Hoque ME, Morshad MN. Somaclonal variation in potato (Solanum tuberosum L.) using chemical mutagens. The Agriculturists. 2014;12(1):15–25.

6. Opoku Gyamfi M, Eleblu JSY, Sarfoa LG, Asante IK, Opoku-Agyemang F, Danquah EY. Induced variations of ethyl methane sulfonate mutagenized cowpea (Vigna unguiculata L. Walp) plants. Front Plant Sci. 2022;13:952247.

7. Dhanavel D, Girija M. Effect of EMS, DES and SA on quantitative traits of cowpea (Vigna unguiculata (L.) Walp.) in M1 generation. Crop Research (Hisar). 2009;37(1–3):239–41.

8. Dhanavel D, Girija M. Effect of EMS on induced morphological mutants in cowpea (Vigna unguiculata (L.) Walp.). Crop Research. 2009;38:27–31.

9. Roychowdhury R, Tah J. Assessment of chemical mutagenic effects in mutation breeding programme for M1 generation of carnation (Dianthus caryophyllus). Res Plant Biol. 2011;1(4).

10. Gnankambary TK, Benoit JB, Sawadogo N, Mahamadou S, Tignegre JB, Yonli D, et al. Genetic variability induced by gamma radiation in cowpea (Vigna unguiculata L. Walp) in Burkina Faso. Eur Sci J. 2019;15(15):153–64.

11. Nawale SR, Apte UB, Jadhav BB. Effect of gamma rays and ethyl methane sulfonate on seed germination and survival of seedlings in cowpea (Vigna unguiculata (L.) Walp.). Journal of Arid Legumes. 2006;3(1):102–5.

12. Bind D, Dwivedi VK. EFFECT OF MUTAGENESIS ON GERMINATION, PLANT SURVIVAL AND POLLEN STERILITY IN M 1 GENERATION OF IN COWPEA [VIGNA UNGUICULATA (L.) WALP] [Internet]. Vol. 48, Indian J. Agric. Res. 2014. Available from: www.arccjournals.com

13. Crino P, Lai A, Bonito RD, Veronese P, Saccardo F. Genetic variability in tomato plants regenerated from irradiated cotyledons. In: Jain SM, Brar DS, Ahloowalia BS, editors. Somaclonal Variation and Induced Mutations in Crop Improvement. Dordrecht: Springer Science & Business Media; 2013.

14. Jain SM, Brar DS, Ahloowalia BS, editors. Somaclonal Variation and Induced Mutations in Crop Improvement. Dordrecht: Springer Science & Business Media; 2013.

15. Jain SM, Maluszynski M. In vitro applications in crop improvement: Recent progress. In: Mujib A, Cho M, Predieri S, Banerjee S, editors. Biotechnology for Crop Improvement. New Delhi, India: IBH–Oxford; 2004. p. 169–202.

16. Jain SM. Mutagenesis in crop improvement under the climate change. Rom Biotechnol Lett. 2010;15(2):88–106.

17. Kumar A, Rai PK. Induced mutagenesis in mungbean (Vigna radiata L.). In: Singh DP, editor. Mutation Breeding in Pulse Crops. Kalyani Publishers; 2006. p. 85–93.

18. Kundu SK, Singh DP. Gamma-ray induced variability for quantitative characters in black gram. Madras Agricultural Journal. 1982;69(10):644–8.

19. Chandra N. Cytological and mutational studies on Gloriosa superba Linn. [Nagpur, India]: Nagpur University; 1988.

20. Khan S, Wani MR. Comparison of the effect of chemical mutagens on mung bean. Advances in Plant Sciences. 2005;18(2):533–5.

21. Khan S, Wani MR. Chemomutagenic effect of two alkylating agents on mung bean (Vigna radiata). Bionotes. 2004;6(3):82–3.

22. Nanda S, Rao GM, Nayak S. Mutagenic effects of EMS on yardlong bean (Vigna sesquipedalis). Indian J Agric Res. 1997;31:602–10.

23. Allard RW. Principles of Plant Breeding. 2nd ed. New York: John Wiley & Sons; 1999.

24. Falconer DS, Mackay TFC. Introduction to Quantitative Genetics. 4th ed. Harlow: Longman; 1996.

25. Principles SBDPB, ed. New Delhi: Kalyani Publishers Methods 7th. Singh BD. Plant Breeding: Principles and Methods. 7th ed. New Delhi: Kalyani Publishers; 2006. 2006.

26. Grafius JE. Components of yield in oats: A geometrical interpretation. Agron J. 1959;51:515–8.

27. Donald CM. The breeding of crop ideotypes. Euphytica. 1968;17:385–403.

28. Gaul H. Mutations in plant breeding. Radiation Botany. 1964;4:155–232.

29. Maluszynski M, Nichterlein K, Van Zanten L, Ahloowalia BS. Officially released mutant varieties—The FAO/IAEA database. Mutation Breeding Reviews. 2000;12:1–84.

30. Goyal P, Khan S, Wani MR. EMS-induced genetic variability for yield and yield components in urdbean (Vigna mungo (L.) Hepper). Legume Research. 2021;44:1220–6.

31. Premsekhar S, Appadurai P. Effect of doses of gamma rays and EMS on induced mutation in pigeonpea. Indian Journal of Agricultural Sciences. 1982;51:381–4.

32. Singh BD. Plant Breeding: Principles and Methods. 7th ed. New Delhi: Kalyani Publishers; 2006.

33. Odeigah PGC, Osanyinpeju AO, Myers GO. Induced mutations in cowpea: Morphological and yield traits. Mutation Breeding Newsletter. 1998;44:10–3.

34. Rai A. Effect of Ethyl Methane Sulfonate Mutagen in M1 Generation in Pigeonpea (Cajanas Cajan L. Millasp). Rajmata Vijayaraje Scindia Krishi Vishwa Vidyalaya; 2011.

35. Khan IA. Mutation studies in mung bean (Vigna radiata L. Wilczek): IX. Estimates of genetic variability. Legume Research. 1988;11(2):89–93.

36. Singh M, Singh VP. Genetic analysis of certain mutant lines of urdbean for yield and quality traits in M4 generation. Indian Journal of Pulses Research. 2001;14(1):60–2.

37. Jagajanantham N, Dhanavel D, Pavadai P. EMS-induced variability in okra (Abelmoschus esculentus L.). Int J Sci Res. 2013;2:148–51.

38. Berenschot AS, Zucchi MI, Tulmann Neto A, Quecini V. Mutagenesis in ornamentals: Application and perspective. Plant Cell Tissue Organ Cult. 2009;98:161–72.

39. Premsekar R, Appadurai R. EMS-induced variability in pigeonpea (Cajanus cajan L.). Madras Agricultural Journal. 1982;69:458–66.

40. Mohanasundaram K, Kalamani A, Ramalingam RS. Induced mutagenesis in cowpea (Vigna unguiculata (L.) Walp.). Legume Research. 1999;22:642–8.

41. Anis M, Sharma Pk, Abbasi N. Gamma rays induced variability in urd bean (Vigna mungo (L.) hepper) I M1 generation. Journal of the Indian Botanical Society. 1999;78(1–2):111–3.

42. Khan SB, Siddiqui A, Nadeem M. Variation in quantitative characters of mung bean after seed treatment with DES. Advances in Plant Sciences. 1994;7(1):41–5.

43. Kansagra UM, Shukla PT. Variability studies in induced mutants of mungbean. Madras Agricultural Journal. 1993;80(4):180–4.

44. Iepziba SJ, Subramanian M. Variability in M3 and M4 mutants of urdbean. Indian Journal of Pulses Research. 1993;6(2):197.

45. Patil BN, Guha VY. Growth attributes in induced mutants of chickpea. PKV Research Journal. 1994;15(2):97–101.

46. Gnanamurthy S, Dhanavel D. Effect of EMS on induced morphological mutants and chromosomal variation in cowpea (Vigna unguiculata (L.) Walp.). International Letters of Natural Sciences. 2014;13:33–43.

47. Gyamfi MO. Inducing Genetic Variability In Cowpea (Vigna Unguiculata, L. Walp Var. Asontem) Using Ethyl Methane Sulfonate A Thesis Presented [Internet]. Available from: http://ugspace.ug.edu.gh

48. Wani AA. Induced mutagenesis for improvement of pulse crops. Plant Breed Rev. 2017;41:155–200.

49. Goyal S, Wani MR, Laskar RA, Raina A, Amin R, Khan S. Induction of morphological mutations and mutant phenotyping in black gram [Vigna mungo (L.) Hepper] using gamma rays and EMS. Vegetos. 2019;32(4):464–72.

50. Pandey B, Dhanasekar P. Development of high-yielding mutant varieties in cowpea. Indian Journal of Genetics. 2003;63:151–9.

51. Kumar Y, Mishra VK. Effect of gamma rays and diethyl sulphate on germination,

growth, fertility and yield in greengram (Vigna radiata L. Wilczek). 1999.

Supporting Information

S1: Trait dataset and cluster grouping of individual M_1 cowpea plants across EMS treatments

Trt	ID	*dtg*	*dff*	*d50f*	*dfh*	*d50mp*	*pdl*	*ln*	*sdl*	*sdw*	*sdt*	*swg*	yld	Cluster group
0mM	A01	4	40	60	67	89	12.94	13	8.94	5.6	4.3	23.2	55.68	1
	A02	4	39	60	53	88	17	18	6.62	4.53	3.86	20.34	42.71	2
	A03	3	40	56	49	87	13.24	21	8.98	5.4	4.13	17.94	43.05	2
	A04	3	35	52	50	81	13.2	13	7.03	4.35	3.29	23.96	52.71	2
	A05	3	43	66	58	93	14.24	18	7.22	4.7	4.02	20.08	43.38	2
	A06	3	30	60	60	78	13.58	21	6.72	4.42	3.7	20.97	41.53	2
	A07	4	40	55	47	75	13.8	12	9.26	5.71	4.19	24.87	34.82	3
	A08	4	41	62	53	83	11.55	19	7.91	5.11	4.17	19.93	36.27	2
	A09	4	37	52	41	67	15.4	14	6.16	4.44	4.41	20.92	72.81	2
	A10	4	37	54	58	89	13.09	16	6.23	4.15	3.64	22.13	44.92	2
	A11	4	40	62	45	74	15.29	18	7.37	4.84	4.29	21.1	31.66	2
	A12	4	44	59	46	76	15.95	17	8.98	5.4	4.13	21.01	43.71	3
	A13	3	43	60	49	87	17.58	16	6.99	4.46	3.81	17.82	42.78	2
	A14	5	30	52	53	80	16.64	18	9.65	5.67	4.28	23.13	73.78	3
	A15	4	39	54	47	80	15.57	17	8.24	5.25	4.13	19.75	40.09	3
	A16	3	45	66	40	70	14.08	24	6.77	4.57	4.36	20.79	39.49	2
	A17	4	31	54	43	66	17.21	12	6.74	4.34	3.42	20.36	61.08	2
	A18	3	32	49	56	94	14.25	20	7.57	5.03	4.92	22.64	67.93	4
	A19	2	35	59	42	72	15.3	13	6.21	4.46	4.43	20.61	54.41	2
	A20	3	33	48	47	84	16.62	20	6.38	4.3	4.1	22.57	72.66	4
	A21	4	40	57	44	71	13.33	17	6.43	4.47	4.19	18.36	50.5	2
	A24	4	39	58	49	72	15.42	17	5.68	3.94	3.63	19.63	61.83	2
	A25	3	39	59	50	70	12.83	21	6.24	4.19	3.71	24.67	51.81	2
	A26	3	37	59	54	86	15.3	23	6.76	4.54	4.53	22.04	52.89	4
	A27	4	32	53	53	84	16	16	11.59	6.08	4.82	19.21	22.86	3
	A28	4	40	65	43	79	11.88	12	6.07	4.03	3.85	21.97	85.68	2
	A29	2	39	57	44	81	14.92	17	8.49	5.13	3.92	23.24	23.24	3
	A30	4	43	68	51	88	13.53	18	8.35	5.15	4.48	18.63	50.31	2
	A31	3	40	64	40	69	14.9	13	6.61	4.53	4.52	18.39	74.66	2
20mM	B01	5	50	70	68	92	12.13	18	6.33	4.24	4.06	22.28	55.69	5
	B02	4	46	66	59	98	13.23	21	6.65	4.38	3.66	26.08	28.69	5
	B03	5	42	60	53	79	14.98	13	6.94	4.58	4.23	25.41	37.36	5
	B04	6	43	60	60	100	16.28	18	9.26	5.71	4.19	25.92	54.44	1
	B05	5	47	66	62	82	13.8	21	10.1	5.78	4.92	25.17	49.83	1
	B06	5	51	75	54	76	15.75	12	10.35	5.76	4.91	25.9	62.15	6
	B07	7	38	60	63	89	11.45	19	11.65	6.44	5.19	29.99	67.18	6
	B08	5	49	74	68	102	15.25	14	9.85	5.67	5.12	22.68	41.27	1
	B09	7	52	74	71	98	13	21	11.59	6.08	4.82	27.48	74.2	6
	B10	6	47	63	56	81	14.31	23	7.03	4.48	3.79	20.06	46.14	2
	B11	7	52	70	60	95	13.3	16	9.75	5.66	4.99	26.1	26.1	3
	B12	3	41	59	49	87	17.7	13	10.4	5.9	5.16	26.32	48.7	6
	B13	4	47	62	66	100	14.25	18	9.9	5.72	4.87	23.49	59.2	1
	B14	7	53	69	58	87	14.5	21	7.18	4.88	4.76	24.86	45.75	5
	B15	3	54	71	55	76	15.25	16	9.75	5.88	4.83	27.77	79.15	1
	B16	5	53	75	64	102	14.7	18	9.75	5.68	4.85	26.24	75.56	1
	B17	3	41	65	71	93	13.7	17	10.4	5.82	4.84	27.34	71.37	6
	B18	6	54	71	62	98	15.5	12	10	5.62	4.89	28.33	45.32	3
	B19	5	36	51	48	68	19.88	19	6.23	4.17	4	24.82	93.59	4
	B20	5	34	57	52	72	14.7	14	6.77	4.57	4.36	26.63	42.6	5

Values highlighted red indicate trait means significantly (P<0.05) below the control, while green indicate values significantly (P<0.05) above the control.Key: Trt= Treatment ID=Genotype Identifier, dtg days to germination, dff = days to first flower, d50f = days to 50% flowering, dfh = days to first harvest, d50mp = days to 50% mature pods, pdl = pod length, pdw = pod width, ln = number of locules, nsp = number of seeds per pod, psa = percent seed abortion, sdl = seed length, sdw = seed width, sdt = seed thickness, swgt = seed weight, yld = yield per plant

S1 Table: Trait dataset and cluster grouping of individual M_1 cowpea plants across EMS treatments (*cont'd*)

Trt	ID	*dtg*	*dff*	*d50f*	*dfh*	*d50mp*	*pdl*	*ln*	*sdl*	*sdw*	*sdt*	*swg*	*yld*	Cluster group
20mM	B21	5	47	63	70	91	13.6	13	9.85	5.64	5.09	22.6	81.37	1
	B22	6	36	56	70	97	12.1	20	7.04	4.98	4.85	21.91	88.96	1
	B23	7	48	73	55	79	13.04	17	8.26	5.04	3.93	22.04	46.28	5
	B24	6	50	74	52	75	13.35	13	8.7	5.13	4.91	17.81	18.7	3
	B25	7	39	61	58	89	17.75	18	9.65	5.31	4.52	25.92	76.99	1
	B26	4	47	72	64	100	14	21	10.1	5.54	4.87	24.26	91.45	1
	B27	7	39	55	56	85	16.1	12	7.04	4.68	4.28	25.59	67.56	4
	B28	4	39	60	67	87	12.13	19	7.3	4.75	4.04	23	103.5	1
	B29	6	43	66	61	94	13.27	14	6.62	4.53	3.86	24.2	45.73	5
	B30	7	40	61	63	95	14.1	13	6.46	4.29	4.1	23.82	28.58	5
	B31	3	39	60	58	98	14.85	20	9.9	5.46	5	24.65	107.22	1
	B32	6	47	71	49	85	14.22	17	6.14	4.06	3.87	22.04	66.11	4
	B33	6	37	61	56	85	16.33	19	6.1	4.05	3.31	27.88	125.44	4
	B34	7	41	57	53	85	12.44	17	7.04	4.98	4.85	22.2	38.86	5
	B35	7	51	68	56	91	14.75	13	10.05	5.24	3.56	26.06	63.85	5
	B36	4	33	54	64	100	14.1	19	10.15	5.18	3.91	23.62	64.48	1
	B37	7	53	73	62	82	14.35	17	11.25	5.77	3.71	26.15	82.38	1
	B38	5	43	61	61	81	14	13	10.3	5.01	3.84	27.88	42.94	5
	B39	6	54	71	49	85	13.25	19	10.4	5.67	4.46	26.71	86.53	1
	B40	7	37	58	61	90	16.6	15	10.2	5.5	4.4	26.06	68.8	6
	B41	7	33	50	60	99	17.52	18	6.43	4.47	4.19	25.04	66.61	4
	B42	5	49	74	69	107	12.7	20	6.74	4.34	3.42	26.21	46.13	5
	B43	3	37	56	58	96	12.25	18	9.3	4.73	3.72	23.01	68.33	5
	B44	6	48	70	69	107	14.43	19	6.65	4.66	4.27	21.38	29.94	5
	B45	3	48	67	62	96	13.75	20	10.1	5.57	4.08	23.17	76.45	1
	B46	6	46	65	55	80	10.5	18	9.5	4.7	3.72	27.56	46.86	5
	B47	4	52	70	59	89	14.5	19	10.15	5.15	3.97	24.42	72.54	1
	B48	4	39	59	72	105	14.5	14	9.65	5.36	3.86	25.18	30.22	5
40mM	C01	4	49	68	67	103	13.95	21	10	4.95	3.8	23.44	50.63	1
	C02	3	43	59	38	68	14.05	21	9.25	4.69	3.71	17.7	77.01	2
	C03	4	52	67	61	85	11.75	12	9.6	4.67	3.43	25.95	61.76	1
	C04	4	40	55	50	76	11.5	17	10	5.5	4.18	25.96	68.52	1
	C05	7	47	67	62	91	14.2	11	9.55	5.61	4.29	26.03	105.43	1
	C06	7	45	70	66	92	11.95	12	9.85	4.84	3.68	25.34	44.6	5
	C07	3	35	60	60	89	11.75	17	9.95	5.49	4.2	28.39	78.35	1
	C08	4	46	62	67	106	14	11	9.95	5.48	4.09	23.9	55.22	1
	C09	3	46	71	61	98	13.15	11	9.85	5.27	3.95	27.53	60.57	5
	C10	4	55	76	68	97	16.75	12	9.75	5.09	4	25.64	57.43	1
	C11	5	38	54	58	96	11	14	9.2	5.13	4.05	23.3	39.61	5
	C12	6	44	59	67	98	13.6	18	9.85	5.56	3.97	26.7	82.24	1
	C13	6	45	67	65	93	13.6	18	8.1	4.72	3.82	26.98	87.43	1
	C14	3	43	68	39	74	14.95	17	9.4	4.63	3.49	17.77	52.79	2
	C15	3	50	71	66	88	15.6	19	10	5.18	4.29	27.88	57.98	1
	C16	7	52	69	55	80	12.5	17	9.6	5.14	3.98	27.98	76.93	1
	C17	7	45	64	55	95	16.65	13	10.45	5.01	3.51	20.9	68.96	4
	C18	5	53	78	66	91	12.6	19	9.65	4.87	3.99	26.66	72.78	1
	C19	4	52	72	65	88	14.8	17	9.85	5.25	3.83	26.46	47.62	5
	C20	4	46	71	47	83	13.7	13	9.3	4.79	3.51	21.5	54.19	5
	C21	3	53	73	58	90	10.5	19	9	5.02	4.06	25.8	59.33	1

Values highlighted red indicate trait means significantly ($P<0.05$) below the control, while green indicate values significantly ($P<0.05$) above the control.Key: Trt= Treatment ID=Genotype Identifier, dtg days to germination, dff = days to first flower, d50f = days to 50% flowering, dfh = days to first harvest, d50mp = days to 50% mature pods, pdl = pod length, pdw = pod width, ln = number of locules, nsp = number of seeds per pod, psa = percent seed abortion, sdl = seed length, sdw = seed width, sdt = seed thickness, swgt = seed weight, yld = yield per plant

S2 Table: Trait dataset and cluster grouping of individual M$_1$ cowpea plants across EMS treatments *(cont'd)*

Trt	ID	*dtg*	*dff*	*d50f*	*dfh*	*d50mp*	*pdl*	*ln*	*sdl*	*sdw*	*sdt*	*swg*	*yld*	Cluster group
40mM	C22	5	55	73	51	78	14.9	15	10	5.5	4.32	25.76	27.04	3
	C23	5	43	64	55	81	12.05	18	9.6	4.79	3.84	21.7	42.96	1
	C24	4	43	65	56	76	13.5	11	9.8	5.31	3.85	26.02	94.7	1
	C25	4	40	59	62	102	12.5	12	9.4	4.81	3.63	23.51	55.96	5
	C26	7	51	70	54	88	11.95	14	9.85	5.25	3.77	23.6	69.4	1
	C27	5	48	69	67	95	6.95	19	9	4.58	5.59	23.01	48.33	1
	C28	7	49	72	67	91	15.95	17	10	5.33	3.86	26.46	47.62	5
	C29	5	39	62	56	92	15.4	13	10	4.98	3.81	25.45	42.76	5
	C30	3	37	57	62	93	14.9	20	10	5.58	3.98	21.34	59.76	1
	C31	5	42	59	50	83	12.75	18	10	4.88	3.57	27.56	48.24	5
	C32	7	34	49	59	84	14.95	19	9.85	4.91	3.87	27.16	62.74	4
	C33	3	52	77	50	76	12.25	19	9.35	5.33	4.21	24.58	63.91	1
	C34	3	44	68	57	93	15.75	15	8.65	5.03	4.35	24.24	56.71	4
	C35	5	48	70	58	94	15.45	18	9.85	4.88	4.01	23.82	60.04	1
	C36	3	54	74	54	83	13.95	12	10.05	4.78	3.63	24.73	61.83	1
	C37	4	49	67	51	76	13.9	17	9.8	5.28	4.08	23.83	31.45	1
	C38	6	34	59	62	89	8.77	11	8.95	5.06	3.81	19.95	39.5	5
	C39	6	37	57	52	92	15.5	19	10.1	4.98	3.91	22.71	27.02	5
	C40	6	35	58	72	98	13.5	17	10	5.4	3.68	24.03	28.83	5
	C41	5	55	80	64	89	14.5	13	10	4.83	3.71	26.36	90.96	1
	C42	7	36	59	58	95	13.35	19	10	5.59	4.49	25.19	25.19	5
	C43	5	35	53	71	95	14	15	10	5.55	4.45	27.28	90.01	1
	C44	5	41	57	55	95	15.5	18	10	4.97	4.14	25.65	76.17	1
	C45	3	40	59	54	84	15.75	12	11.05	5.07	3.8	27.77	83.32	1
	C46	5	52	71	64	103	16.15	17	10.9	5.69	4.25	24.74	59.87	1
	C47	4	40	57	63	87	13.95	11	10	5.58	4.09	23.3	37.51	3
	C48	4	50	73	62	92	16.75	13	10	5.19	3.99	19.97	19.97	3
	C49	7	39	55	59	80	14.75	8	10.4	5.29	3.92	22.82	60.26	3
	C50	3	51	69	62	84	14.5	9	10.1	5.39	3.93	23.53	54.59	5
	C51	4	53	70	62	87	12.5	13	9.9	5.3	3.96	19.66	21.63	3
	C52	4	39	60	64	86	15.9	8	9.9	5.17	4.02	23.13	60.13	4
	C53	6	39	63	62	85	14.75	9	9.75	5.22	3.9	19.28	75.19	4
	C54	5	41	59	54	80	16.55	12	10.15	5.58	3.55	25.23	55.51	3
	C55	7	53	70	51	89	14.5	11	9.85	4.76	3.69	25.06	52.62	5
80mM	D01	7	49	69	49	80	13.5	17	10	4.94	3.95	22.58	31.17	3
	D02	6	50	72	54	92	10.75	10	10.7	5.71	4.28	24.18	42.55	1
	D03	3	40	58	70	93	13.5	9	9.85	5.61	4.56	24.13	74.33	1
	D04	5	55	73	72	100	12.5	13	9.35	5.34	3.81	18.82	76.2	1
	D05	7	35	53	59	96	11.75	10	9.95	5.69	4.18	22.05	45.64	5
	D06	4	34	59	55	93	15	9	10.3	5.68	4.16	20.79	50.32	3
	D07	4	46	70	49	83	12.5	13	9.7	5.44	3.91	20.34	31.72	5
	D08	4	48	69	61	81	15.25	13	10.55	6.11	4.94	26.24	43.3	3
	D09	5	47	65	66	100	15	9	10.3	5.21	4.28	27.62	39.78	1
	D10	4	53	78	58	97	13.75	10	9.85	5.04	4.38	24.53	35.57	3
	D11	7	46	62	61	81	14	12	9.95	5.55	4.91	22.22	34	1
	D12	3	43	68	59	95	15.75	9	10.05	5.58	4.7	22.36	60.82	6

Values highlighted red indicate trait means significantly (P<0.05) below the control, while green indicate values significantly (P<0.05) above the control.Key: Trt= Treatment ID=Genotype Identifier, dtg days to germination, dff = days to first flower, d50f = days to 50% flowering, dfh = days to first harvest, d50mp = days to 50% mature pods, pel = peduncle length at first harvest, npp = number of pods per peduncle, nptm = number of pods per plant at maturity, nspt = number of seeds per plant, pdl = pod length, pdw = pod width, ln = number of locules, nsp = number of seeds per pod, psa = percent seed abortion, sdl = seed length, sdw = seed width, sdt = seed thickness, swgt = seed weight, yld = yield per plant

S1 Table: Trait dataset and cluster grouping of individual M_1 cowpea plants across EMS treatments *(cont'd)*

Trt	ID	*dtg*	*dff*	*d50f*	*dfh*	*d50mp*	*pdl*	*ln*	*sdl*	*sdw*	*sdt*	*swg*	yld	Cluster group
80mM	D13	6	42	59	69	91	16.5	13	10.05	5.2	4.67	27	64.8	1
80mM	D14	6	52	74	45	81	12.75	12	9.4	5.37	4.46	24.66	54.24	1
80mM	D15	4	43	68	61	92	14.5	9	10.5	5.47	4	21.45	36.03	3
80mM	D16	5	48	68	65	92	13	13	9.9	5.02	3.95	22.74	68.23	1
80mM	D17	6	40	56	64	86	12.75	11	9.4	5.91	4.89	23.07	49.84	6
80mM	D18	3	44	68	64	101	11.45	7	9.95	4.94	3.97	25.55	35.77	1
80mM	D19	5	43	58	59	91	14.5	12	11	5.24	3.41	23.94	55.07	5
80mM	D20	4	55	75	64	103	11.25	13	10.75	5.41	4.21	22.04	65.9	1
80mM	D21	3	37	59	59	97	17.5	8	11	5.35	3.74	24.48	42.83	3
80mM	D22	7	47	62	63	92	14.5	14	9.35	4.73	3.74	20.59	40.77	4
80mM	D23	7	37	55	57	78	16	13	9.35	5.21	3.94	22.7	25.88	3
80mM	D24	4	40	62	72	109	14.25	8	9.15	4.95	4.07	17.35	58.65	4
80mM	D25	3	45	68	68	90	15	9	11	5.62	4.15	24.44	102.66	1
80mM	D26	4	46	63	71	110	17.1	13	8.95	5.02	3.86	22.7	22.7	3
80mM	D27	4	44	62	61	86	16.25	8	10	5.31	4.04	21.91	42.73	4
80mM	D28	6	43	66	60	92	17.1	9	10.45	5.48	4.28	22.32	75	4
80mM	D29	7	39	62	58	82	12.75	12	7.95	4.28	3.45	21.58	69.93	4
80mM	D30	4	35	60	56	84	12.75	11	10.25	5.02	3.79	25.06	36.08	5
80mM	D31	4	35	55	54	87	14.9	17	10.75	5.17	4.02	21.98	24.17	5
80mM	D32	4	44	62	68	96	14.15	11	10.2	6.05	4.82	22.16	37.23	1
80mM	D33	4	37	60	48	85	11.25	7	10.3	4.81	3.92	25.86	46.54	5
80mM	D34	4	45	66	52	83	12.25	12	10.25	4.97	3.46	21.21	23.75	3
80mM	D35	7	41	66	58	81	16.75	13	9.3	5.09	4.06	21.79	35.96	3
80mM	D36	3	53	71	49	73	14	8	11	5.64	4.37	28.29	74.69	6
80mM	D37	4	43	61	59	80	15.25	9	10	5.38	4.08	25.05	50.86	5
80mM	D38	5	42	57	62	88	15.5	10	9.3	5.14	3.5	17.49	44.24	4
80mM	D39	4	40	56	52	90	15.75	9	9.4	5.3	4.06	19.27	48.57	3
80mM	D40	4	44	69	54	77	9.95	13	8.55	5.18	3.96	19.42	55.55	2
80mM	D41	7	45	63	59	81	15	12	9	4.79	3.75	19.76	80.03	4
80mM	D42	3	55	80	66	96	9.95	11	9.9	4.72	3.72	22.02	29.06	3
80mM	D43	3	54	73	48	69	11.7	17	10.5	4.9	3.67	21.98	42.19	1
80mM	D44	6	48	65	52	86	9.5	12	11.6	6.03	4.46	24.35	64.29	1
80mM	D45	4	47	68	60	92	11.5	9	9.8	5.4	4.22	23.58	106.09	1
80mM	D46	3	44	62	57	78	12.5	13	9.8	5.05	4.15	21.53	45.21	3
80mM	D47	3	38	59	58	78	13	13	9.85	5.08	3.99	18.45	18.45	3
80mM	D48	5	47	65	52	84	13.65	8	10	5.3	4.24	22.52	85.13	1
80mM	D49	7	52	72	62	83	11.2	9	10.45	5.48	4.03	22.93	79.1	1
80mM	D50	3	42	60	52	92	13.1	12	9.65	4.82	3.7	22.5	43.19	5
80mM	D51	7	33	56	50	83	8.45	11	9.55	4.76	3.84	22.62	64.7	5
80mM	D52	5	55	77	56	84	14.2	17	10.4	4.87	3.87	19.54	22.48	3
80mM	D53	7	43	62	59	99	11.4	12	9.8	5.51	4.38	22.83	55.25	1
80mM	D54	5	47	71	60	85	11.5	9	9.8	5.44	4.1	20.24	28.33	3
80mM	D55	4	37	62	43	74	14.25	13	9.05	4.85	3.85	20.31	35.34	2
80mM	D56	5	53	69	57	94	13.5	23	10.15	5.03	3.78	28.68	111.85	1

Values highlighted red indicate trait means significantly ($P<0.05$) below the control, while green indicate values significantly ($P<0.05$) above the control.Key: Trt= Treatment ID=Genotype Identifier, dtg = days to germination, dff = days to first flower, d50f = days to 50% flowering, dfh = days to first harvest, d50mp = days to 50% mature pods, pdl = pod length, pdw = pod width, ln = number of locules, nsp = number of seeds per pod, psa = percent seed abortion, sdl = seed length, sdw = seed width, sdt = seed thickness, swgt = seed weight, yld = yield per plant

S2 Table: PCA loadings and variance explained for traits in M_1 cowpea

Traits	PC1	PC2	PC3	PC4	PC5	PC6	PC7	PC8	Uniqueness
sdl	0.73								0.18
sdw	0.70		0.50						0.11
dfh	0.68			0.50				-0.32	0.14
dff	0.61		-0.64						0.10
d50mp	0.59			0.51				-0.40	0.13
d50f	0.57		-0.68						0.09
swg	0.53				0.35			0.43	0.36
sdt	0.38		0.42			0.52	-0.38		0.24
nspt		0.96							0.02
yld		0.95							0.02
nsp		0.75		0.36	-0.38		-0.32		0.03
nptm		0.32		-0.66			0.53		0.04
pdl			0.42						0.77
pdw					0.54	-0.35		0.32	0.40
ln					0.47	0.63			0.22
dtg					0.46	-0.33			0.51
psa					-0.42			0.32	0.52
npp						0.32	0.41	0.43	0.43
**Eigenvalue**	**3.18**	**2.71**	**1.70**	**1.51**	**1.34**	**1.17**	**1.07**	**1.03**	
**Proportion %**	**17.70**	**15.10**	**9.40**	**8.40**	**7.50**	**6.50**	**6.00**	**5.70**	
**Cumulative %**	**17.70**	**32.70**	**42.20**	**50.60**	**58.00**	**64.50**	**70.50**	**76.20**	

PC= principal component, dtg = days to germination, dff = days to first flower, d50f = days to 50% flowering, dfh = days to first harvest, d50mp = days to 50% mature pods, npp = number of pods per peduncle, nptm = number of pods per plant at maturity, nspt = number of seeds per plant, pdl = pod length, pdw = pod width, ln = number of locules, nsp = number of seeds per pod, psa = percent seed abortion, sdl = seed length, sdw = seed width, sdt = seed thickness, swgt = seed weight, yld = yield per plant

Figure 1

Click here to access/download;Figure;Figure1.tif

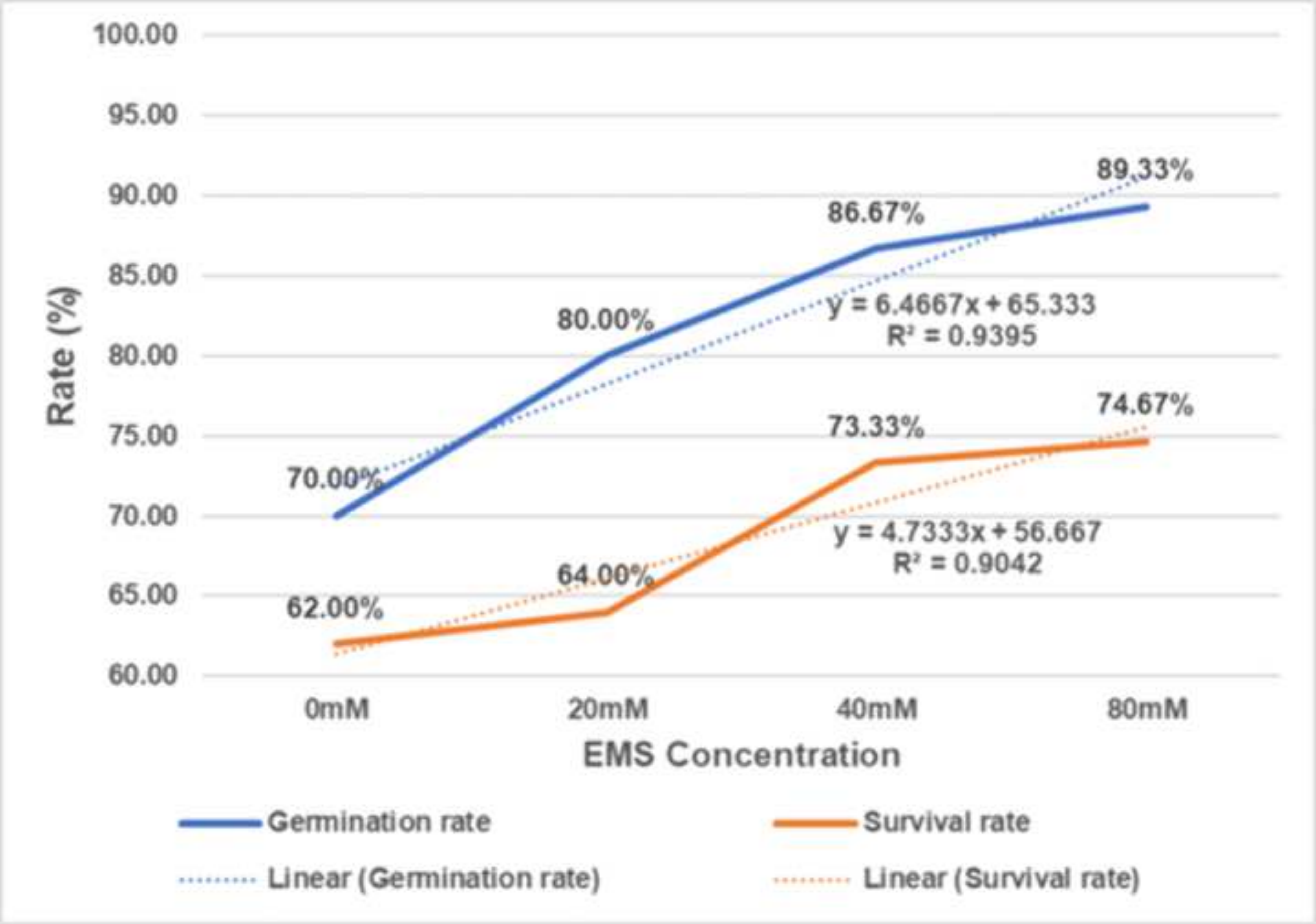

Figure 2

Click here to access/download;Figure;Figure2.tif

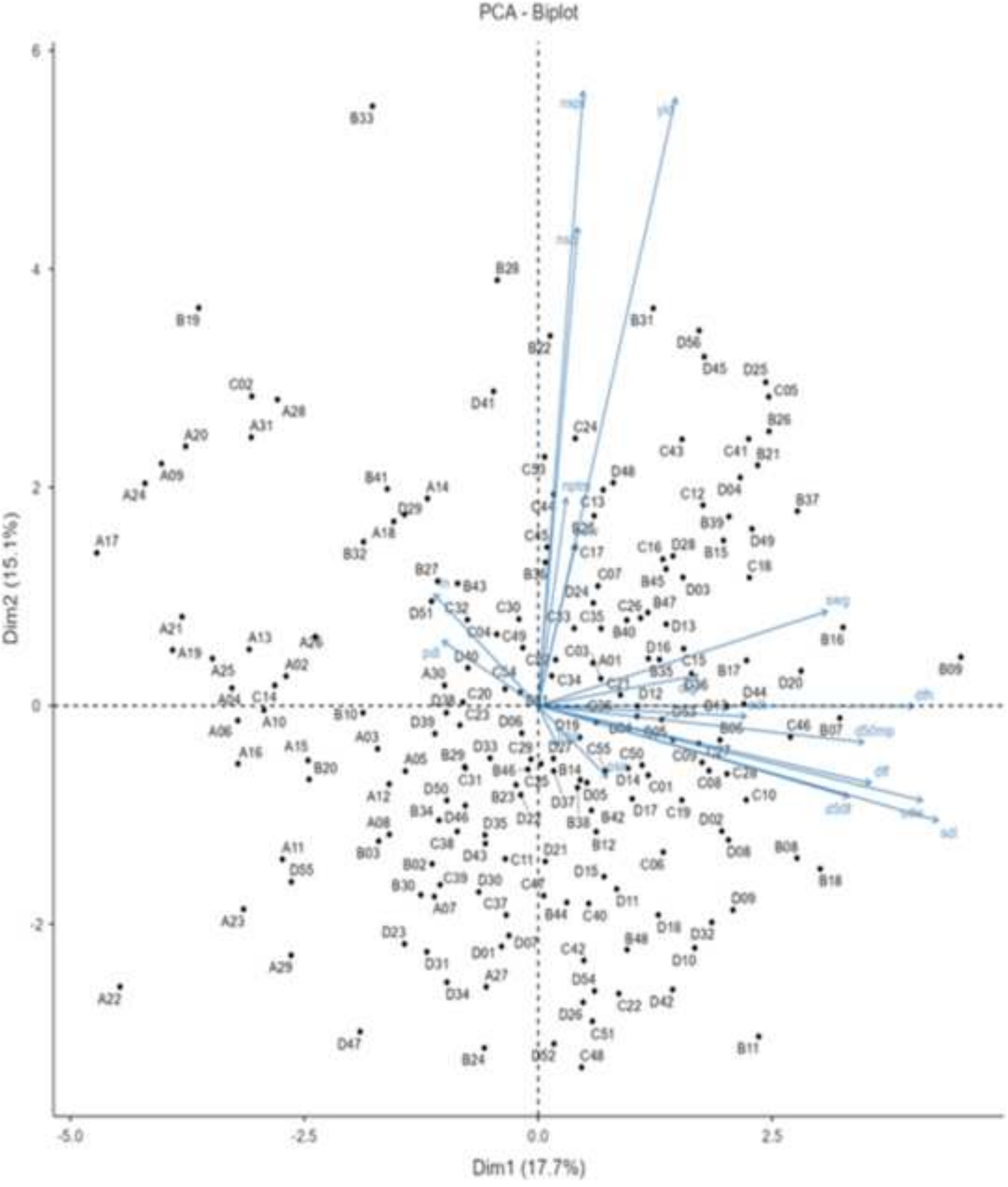

Figure 3

Click here to access/download;Figure;Figure3.tif

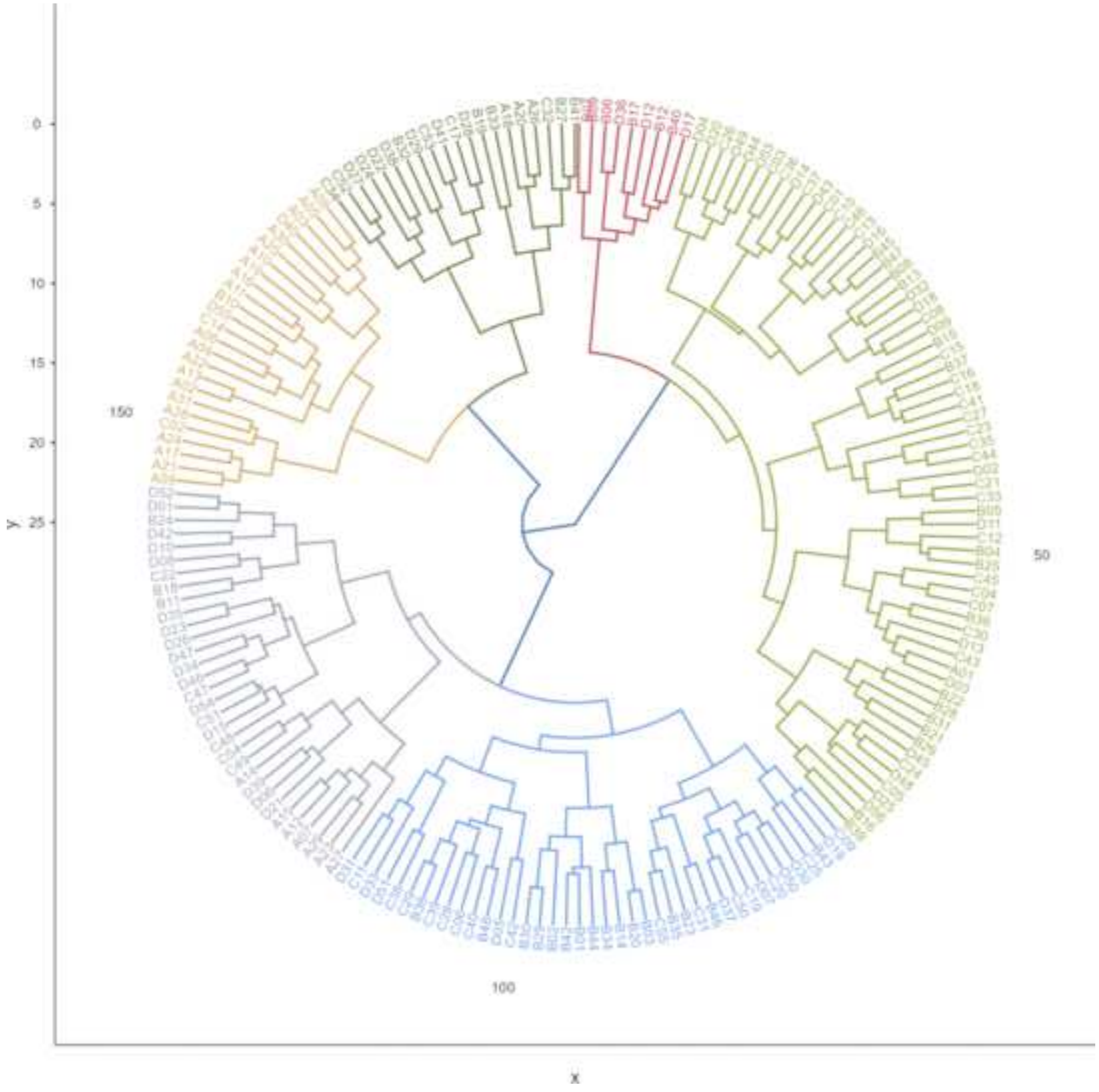

Supporting Information

Click here to access/download
Supporting Information
Supporting Information.docx
