# Supplementary material for: Effect of ethyl methane sulfonate mutagenesis on phenological, yield-related and yield traits in cowpea (Vigna unguiculata (L.) Walp)
Source: PLoS One. 2026 Jul 20;21(7):e0354037. doi: 10.1371/journal.pone.0354037 (PMC13384270; doi:10.1371/journal.pone.0354037)
Supplement: Attachment — Submitted filename: Response_to_Reviewers.pdf [file pone.0354037.s007.pdf]

HENRY KOFI MENSAH
Department of Plant and Environmental Biology
University of Ghana
P. O. Box LG 55, Legon–Accra, Ghana
Email: henryquofi@gmail.com
Tel: +1 437 383 2410

09th April 2026

The Academic Editor and Reviewers
PLOS ONE

Dear Academic Editor and Reviewers,

Response to Reviewers – Manuscript ID: PONE-D-26-01099

We sincerely thank the academic editor and reviewers for the careful and constructive evaluation of our manuscript. We have carefully considered all comments raised and have addressed each one fully in the revised manuscript. The specific revisions made in response to each comment are detailed in the accompanying point-by-point response letter. We are confident that these changes have substantially improved the quality, clarity and depth of the manuscript, and we hope the revised submission meets the standards of PLOS ONE.

Sincerely yours,

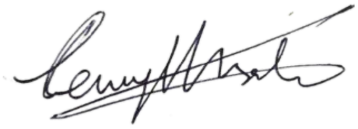

Henry Kofi Mensah (Corresponding author)
On behalf of all co-authors

RESPONSE TO EDITOR AND REVIEWERS

Reviewer #1 Comment 1:
The rationale for selecting the EMS concentrations and the fixed exposure duration should be clarified in relation to previous studies, and the unexpectedly lower germination and survival rates observed in the control compared with EMS treatments should be discussed more explicitly to avoid potential misinterpretation.

Response:
We thank the reviewer for raising both points.
Regarding EMS concentrations and exposure duration: We have now clarified in the revised manuscript that 20-, 40-, and 80-mM EMS concentrations with a fixed 6-hour exposure were selected based on prior mutagenesis studies in cowpea and closely related legumes that reported effective mutation induction at similar concentration ranges and exposure durations. This selection ensured biological comparability with published literature while covering a dose range sufficient to produce measurable variation in phenological, yield-related and yield traits.

Regarding the lower germination and survival in the control: We agree that this result required explicit discussion to prevent misinterpretation. We now attribute the lower germination and survival in the 0 mM control group primarily to variability in seed physiological status specifically, pre-existing dormancy or uneven seed viability within the control lot, which received no pre-treatment. It is additionally plausible that low-dose EMS exposure elicited a mild hormetic or priming-like response, stimulating metabolic activation in treated seeds at sub-lethal concentrations, a phenomenon that has been reported in leguminous crops subjected to sub-lethal chemical mutagen doses. Both explanations have been incorporated into the revised discussion section to prevent misinterpretation.

Reviewer #1 Comment 2:
Sample sizes should be reported consistently across all traits.

Response:
We agree with this observation. The revised manuscript now consistently reports sample sizes for each treatment group across all traits. Treatment-wise sample sizes, N = 31 (0 mM), 48 (20 mM), 55 (40 mM), and 56 (80 mM) are now stated in all relevant tables and sections.

Reviewer #1 Comment 3:
The presentation of statistical significance in tables should be standardized for clarity.

Response:
We thank the reviewer for this observation. All tables in the revised manuscript have been reviewed and standardized to present statistical significance consistently. Significance codes (‘***’ for $p \leq 0.001$, ‘**’ for $p \leq 0.01$, ‘*’ for $p \leq 0.05$, and ‘ns’ for non-significant) are now uniformly applied and clearly defined in all table footnotes. Pairwise t-values and overall P-values are presented in a consistent format across all trait tables.

Reviewer #1 Comment 4:
Since the study is confined to the M_1 generation, interpretations related to breeding value and selection should be slightly moderated, with clearer emphasis on the need for confirmation in M_2 and subsequent generations due to chimerism and the limited expression of recessive mutations in M_1.

Response:

We fully agree with this important observation. Throughout the revised manuscript particularly in the Discussion and Conclusion sections we have moderated all statements relating to breeding value and selection. We now clearly emphasize that observations in the M_1 generation are preliminary in nature and may be influenced by chimerism and by the limited phenotypic expression of recessive mutations, which are detectable only upon homozygosity in M_2 and later generations. Specifically, we have added explicit statements to the effect that the putative mutants identified through biplot and cluster analysis must be confirmed through M_2 progeny evaluation before any breeding value can be attributed, and that trait stabilization requires evaluation across multiple selfing generations. This framing is now consistent with established principles of mutation breeding and aligned with the interpretive conventions applied in comparable M_1 studies in cowpea and related legumes.

Reviewer #1 Comment 5:
Although the manuscript is generally well written, minor grammatical issues, long sentences, and occasional repetition should be corrected during revision.

Response:
We thank the reviewer for this feedback. The entire manuscript has been carefully proofread. Long sentences have been broken into shorter, clearer statements; repeated phrases and redundant expressions have been removed or consolidated; and minor grammatical issues throughout the text have been corrected. We trust that these revisions have meaningfully improved the readability and overall presentation of the manuscript.

Reviewer #2 Comment 1:
Statement from manuscript:
"This study investigated the impact of ethyl methane sulfonate (EMS) on the M1 generation of cowpea (Vigna unguiculata (L.) Walp). Seventy-five seeds of 'Wang Kae' cowpea variety were soaked in water for six hours and later subjected to three different doses of ethyl methane sulfonate (20 mM, 40 mM and 80 mM) with fifty serving as a control."
Reviewer Comment:
What do you mean by this statement?

Response:
We thank the reviewer for flagging this ambiguity. The original statement was poorly worded and could be misinterpreted to suggest that seeds were first soaked in water for six hours and then additionally soaked in EMS, which would be incorrect. The revised manuscript now makes the protocol explicit and unambiguous. Seventy-five seeds per EMS treatment group were pre-soaked in distilled water for six hours to facilitate imbibition and improve mutagen uptake, after which they were immediately transferred to and soaked in the respective EMS solutions (20 mM, 40 mM, or 80 mM) for a further six hours. A separate group of fifty seeds was soaked in distilled water only for six hours and served as the untreated control (0 mM). Following treatment, all seeds treated, and control were thoroughly washed in running water for 30 minutes to remove residual EMS, then planted to raise the M_1 generation. This clarified protocol has been rewritten in the Materials and Methods section to remove any ambiguity regarding the treatment sequence.

Reviewer #2 Comment 2:
1. The introduction is very shallow. No clear picture of previous studies and outcome. No evidence of justification of study. Problem statement not convincing or not there at all. The crop not well explained and described.
2. Your paragraphs are too short to be termed as paragraphs. Kindly structure the introduction well to give a clear picture of the study review.
3. Needs to be referenced.

Response:
We fully accept this criticism and have substantially revised and expanded the Introduction in the revised manuscript. The original Introduction was underdeveloped, consisting of short, underdeveloped paragraphs that did not adequately describe the crop, review the existing literature, or justify the study. The following specific changes have been made:

First, the description of cowpea has been expanded to include its economic and nutritional importance, its role in food and income security for smallholder farmers in Sub-Saharan Africa, its protein content and drought tolerance, and its significance as a climate-resilient legume in the context of food security in the tropics. Appropriate citations have been added to all factual statements about the crop.

Second, a structured review of previous EMS mutagenesis studies in cowpea and related legumes has been added. This now includes a summary of outcomes from key studies, including Opoku Gyamfi et al. (2022) in cowpea, Gnanamurthy and Dhanavel (2014) in cowpea, and Odeigah et al. (1998), along with comparable EMS studies in mung bean, black gram, and pigeonpea. The review highlights both the successes of mutation breeding in pulses and the gaps that remain, particularly the limited work in West African cowpea germplasm.

Third, a clear and convincing problem statement has been added, noting that: (i) the genetic base of cowpea in West Africa is narrow; (ii) conventional breeding has made limited progress in improving yield-related traits due to this limited variability; (iii) EMS mutagenesis offers a cost-effective and well-established route to broadening genetic diversity; and (iv) no previous study has characterized the M_1 EMS mutagenesis response of the '*Wang Kae*' cultivar across the full spectrum of phenological, yield-related, and yield traits using multivariate analysis.

Fourth, the short, disjointed paragraphs have been consolidated into three well-developed paragraphs covering: (a) the importance and constraints of cowpea production; (b) the rationale for and evidence base of mutation breeding as a strategy; and (c) the specific objectives of the present study. All statements in the revised Introduction are fully referenced.

Reviewer #2 Comment 3:
Statement from manuscript:
"Germination and survival rate. Germination of seeds was recorded 7 days after planting. Survival plants were recorded at 50% flowering and germination and survival rate were recorded using the formula by [11] ..."
Reviewer Comment:
This should be part of parameters recorded, not Statistical analysis.

Response:
The reviewer is entirely correct. The formulas for germination rate and seedling survival rate are measurement and data collection procedures, not statistical analyses, and should therefore

appear under the ‘Parameters Studied’ sub-section rather than the ‘Statistical Analysis’ sub-section. In the revised manuscript, this content has been moved to the ‘Parameters Studied in M_1 Generation’ section, where it now appears as the first sub-section under data collection and measurement, immediately after the list of phenological and yield-related traits.

Reviewer #2 Comment 4:
To ensure a proper review of this study, I humbly suggest authors separate results from the discussion. As it stands now some parts are well discussed and others are not discussed at all. This makes the manuscript very shallow and not presentable. Conventionally, a separate discussion will give a clear picture of the study since it will capture the review done in the introduction and your findings together with previous studies to help in making the manuscript solid.

Response:
We thank the reviewer for this important and well-founded observation. We agree entirely that the combined Results and Discussion format led to uneven treatment of the data: some findings were well discussed while others lacked contextualization, and the overall narrative failed to build a coherent argument connecting the results to the broader literature.

In the revised manuscript, the Results and Discussion sections have been fully separated. The Results section now presents all findings in a logical, structured sequence beginning with percentage germination and seedling survival, followed by the contrast analysis of phenological traits, yield-related traits, and yield per plant (with Tables 1 and 2), and concluding with the multivariate analysis results (PCA biplot and cluster analysis). Each sub-section reports the data clearly and objectively without interpretation.

The Discussion section has been substantially expanded and now constitutes a standalone section that: (i) interprets the key findings in the context of the hypotheses and study objectives; (ii) compares and contrasts the results with findings from previous EMS and mutagen studies in cowpea and related legumes; (iii) explains the biological mechanisms underlying the observed patterns, including the dose–response relationships, phenological delay, seed trait changes, and yield responses; (iv) discusses the significance of the PCA and cluster analysis results for putative mutant selection; and (v) acknowledges the limitations of M_1 generation data and the need for M_2 confirmation. The revised Discussion is directly informed by and refers to the expanded Introduction, creating a coherent narrative throughout the manuscript.
