# Supplementary material for: Effect of ethyl methane sulfonate mutagenesis on phenological, yield-related and yield traits in cowpea (Vigna unguiculata (L.) Walp)
Source: PLoS One. 2026 Jul 20;21(7):e0354037. doi: 10.1371/journal.pone.0354037 (PMC13384270; doi:10.1371/journal.pone.0354037)
Supplement: Attachment — Submitted filename: PONE-D-26-01099_R1_Review.pdf [file pone.0354037.s006.pdf]

PLOS One

Effect of Ethyl Methane Sulfonate Mutagenesis on Phenological, Yield-Related and Yield Traits in Cowpea (Vigna unguiculata (L.) Walp)

--Manuscript Draft--

**Manuscript Number:**	PONE-D-26-01099R1
**Article Type:**	Research Article
**Full Title:**	Effect of Ethyl Methane Sulfonate Mutagenesis on Phenological, Yield-Related and Yield Traits in Cowpea (Vigna unguiculata (L.) Walp)
**Short Title:**	Effect of EMS on Agronomic Traits in Cowpea
**Corresponding Author:**	HENRY KOFI MENSAH, MPhil Botany<br>University of Ghana<br>ACCRA, GHANA
**Keywords:**	ethyl methanesulfonate (EMS); induced mutagenesis; mutation breeding; cowpea (Vigna unguiculata); genetic variability; yield traits; phenological traits; seed traits; principal component analysis; Cluster analysis
**Abstract:**	This study investigated the mutagenic effects of ethyl methane sulfonate (EMS) on the M_1 generation of cowpea (Vigna unguiculata (L.) Walp.) cultivar 'Wang Kae'. A total of 275 M_0 seeds were treated with EMS concentrations of 20 mM, 40 mM, and 80 mM (75 seeds per treatment) by soaking for six hours, while 50 untreated seeds served as the control (0 mM). Phenological, yield-related and yield traits were recorded, and data were analysed using Jamovi 2.7.15 and JASP 0.95.4.0 through one-way ANOVA with post hoc contrast, principal component biplot, and cluster analyses. No optimal mutagenic concentration (LD50) was identified. Seed germination and seedling survival rates increased with increasing EMS concentration, ranging from 70.00% and 62.00% in the control (0 mM) to 89.33% and 74.67% at 80 mM, following the trend 0 mM < 20 mM < 40 mM < 80 mM. Significant differences ($P < 0.05$) were observed among treatments for all phenological traits, pod length, locule number, seed traits, and yield per plant. Yield was significantly higher ($P = 0.047$) at 20 mM (61.19 ± 3.34 g) compared to the control. Contrast analysis identified genotypes B33 and D56 as the most productive mutants, with yields of 125.44 g and 111.85 g, respectively. Principal component analysis extracted eighteen components, with the first four cumulatively explaining 50.60% of total variation. Biplot analysis of PC1 and PC2 captured all phenological traits, key seed traits, and yield attributes, highlighting the superior performance of B33 and D56. Cluster analysis partitioned the 190 genotypes into six groups, with B33 and D56 constituting distinct clusters. EMS mutagenesis effectively induced heritable phenotypic variation, with putative superior genotypes identified for advancement to M_2 and evaluation in replicated multi-environment trials toward the development of farmer- and consumer-preferred cowpea varieties.
**Order of Authors:**	HENRY KOFI MENSAH, MPhil Botany
	Ralieva Awura Korkor Nortey, MPhil Botany
	Isaac Kojo Asante
	Felicia Oppong-Adjei
**Response to Reviewers:**	Dear Editorial Office,<br>Thank you for the reviewers' constructive feedback on manuscript PONE-D-26-01099. We have carefully addressed all comments raised by both reviewers and the Academic Editor. The revised manuscript, tracked changes version, and a detailed point-by-point Response to Reviewers have been uploaded accordingly. All PLOS ONE formatting and Supporting Information requirements have also been met.<br>Yours sincerely,<br>Dr. Mensah
**Additional Information:**	
**Question**	**Response**
**Financial Disclosure**	The author(s) received no specific funding for this work.

Powered by Editorial Manager® and ProduXion Manager® from Aries Systems Corporation

Enter a financial disclosure statement that describes the sources of funding for the work included in this submission. Review the submission guidelines for detailed requirements. View published research articles from *PLOS ONE* for specific examples.<br><br>This statement is required for submission and **will appear in the published article** if the submission is accepted. Please make sure it is accurate.<br><br>**Funded studies**<br>Enter a statement with the following details:<br>• Initials of the authors who received each award<br>• Grant numbers awarded to each author<br>• The full name of each funder<br>• URL of each funder website<br>• Did the sponsors or funders play any role in the study design, data collection and analysis, decision to publish, or preparation of the manuscript?<br><br>Did you receive funding for this work?	
**Competing Interests**<br><br>Use the instructions below to enter a competing interest statement for this submission. On behalf of all authors, disclose any competing interests that could be perceived to bias this work—acknowledging all financial support and any other relevant financial or non-financial competing interests.<br><br>This statement is required for submission and **will appear in the published article** if the submission is accepted. Please make sure it is accurate and that any funding sources listed in your Funding Information later in the submission form are also declared in your Financial Disclosure statement.	The authors have declared that no competing interests exist.

View published research articles from *PLOS ONE* for specific examples.<br><br>**NO authors have competing interests**<br><br>Enter: *The authors have declared that no competing interests exist.*<br><br>**Authors with competing interests**<br><br>Enter competing interest details beginning with this statement:<br><br>*I have read the journal's policy and the authors of this manuscript have the following competing interests: [insert competing interests here]*<br><br>* typeset	
**Data Availability**<br><br>Authors are required to make all data underlying the findings described fully available, without restriction, and from the time of publication. PLOS allows rare exceptions to address legal and ethical concerns. See the PLOS Data Policy and FAQ for detailed information.<br><br>A Data Availability Statement describing where the data can be found is required at submission. Your answers to this question constitute the Data Availability Statement and **will be published in the article**, if accepted.<br><br>**Important:** Stating 'data available on request from the author' is not sufficient. If your data are only available upon request, select 'No' for the first question and explain your exceptional situation in the text box.<br><br>Do the authors confirm that all data underlying the findings described in their manuscript are fully available without restriction?	Yes - all data are fully available without restriction
Additional data availability information:	

**Ethics Statement**<br><br>Enter an ethics statement for this submission. This statement is required if the study involved:<br><br>• Human participants<br>• Human specimens or tissue<br>• Vertebrate animals or cephalopods<br>• Vertebrate embryos or tissues<br>• Field research<br><br>Write "N/A" if the submission does not require an ethics statement.<br><br>General guidance is provided below. Consult the submission guidelines for detailed instructions. **Make sure that all information entered here is included in the Methods section of the manuscript.**	N/A

Powered by Editorial Manager® and ProduXion Manager® from Aries Systems Corporation

**Format for specific study types**<br><br>**Human Subject Research (involving human participants and/or tissue)**<br>• Give the name of the institutional review board or ethics committee that approved the study<br>• Include the approval number and/or a statement indicating approval of this research<br>• Indicate the form of consent obtained (written/oral) or the reason that consent was not obtained (e.g. the data were analyzed anonymously)<br><br>**Animal Research (involving vertebrate animals, embryos or tissues)**<br>• Provide the name of the Institutional Animal Care and Use Committee (IACUC) or other relevant ethics board that reviewed the study protocol, and indicate whether they approved this research or granted a formal waiver of ethical approval<br>• Include an approval number if one was obtained<br>• If the study involved *non-human primates*, add *additional details* about animal welfare and steps taken to ameliorate suffering<br>• If anesthesia, euthanasia, or any kind of animal sacrifice is part of the study, include briefly which substances and/or methods were applied<br><br>**Field Research**<br><br>Include the following details if this study involves the collection of plant, animal, or other materials from a natural setting:<br>• Field permit number<br>• Name of the institution or relevant body that granted permission	
Data Availability<br><br>Before publication, Authors are required to make fully available and without restriction all data underlying their findings. Sharing the data underlying your findings increases the visibility, credibility, and impact of your work, while supporting transparency and future scientific discovery. Please see our PLOS Data Policy page for detailed information on this policy.</p> A Data Availability	All relevant data are within the manuscript and its Supporting Information files.

Powered by Editorial Manager® and ProduXion Manager® from Aries Systems Corporation

Statement, detailing where the data can be accessed, is required at first submission. Insert your Data Availability Statement in the box below.</p><br><br>Please see the data reporting of our submission guidelines for instructions on what you need to include in your Data Availability Statement. Examples of acceptable Data Availability Statements are included here for reference:</p><br> <br> - The minimal data set [and accompanying code, where generated] is available at [repository name] via [DOI hyperlink] <br> - No data was generated by this study. The following existing data sources were used: [dataset name] from [dataset location] available via [web address or DOI hyperlink]. <br> - For Study Protocols: No datasets were generated or analysed during the current study. All relevant data from this study will be made available upon study completion. <br> <br><br><i>PLOS allows rare exemptions to address legal and ethical concerns. If you have legal or ethical restrictions, please use the box below to detail these in full sentences for the Journal team to consider.</i></p><br><br>This statement will be typeset if the manuscript is accepted for publication.</p>	

Powered by Editorial Manager® and ProduXion Manager® from Aries Systems Corporation

Effect of Ethyl Methane Sulfonate Mutagenesis on Phenological, Yield-Related and Yield Traits in Cowpea *(Vigna unguiculata* (L.) Walp)

Henry .K. Mensah[1¶*], Ralieva A.K. Norety[1¶] , Isaac .K. Asante[1&], Felicia Oppong[1&]

[1]Department of Plant and Environmental Biology, University of Ghana, Accra, Ghana

*Corresponding author

henryquofi@gmail.com (HKM)

.

[¶] These authors contributed equally to this work.

[&]These authors also contributed equally to this work.

Abstract

This study investigated the mutagenic effects of ethyl methane sulfonate (EMS) on the M_1 generation of cowpea (*Vigna unguiculata* (L.) Walp.) cultivar '*Wang Kae'*. A total of 275 M_0 seeds were treated with EMS concentrations of 20 mM, 40 mM, and 80 mM (75 seeds per treatment) by soaking for six hours, while 50 untreated seeds served as the control (0 mM). Phenological, yield-related and yield traits were recorded, and data were analysed using Jamovi 2.7.15 and JASP 0.95.4.0 through one-way ANOVA with post hoc contrast, principal component biplot, and cluster analyses. No optimal mutagenic concentration (LD50) was identified. Seed germination and seedling survival rates increased with increasing EMS concentration, ranging from 70.00% and 62.00% in the control (0 mM) to 89.33% and 74.67% at 80 mM, following the trend 0 mM < 20 mM < 40 mM < 80 mM. Significant differences ($P < 0.05$) were observed among treatments for all phenological traits, pod length, locule number, seed traits, and yield per plant. Yield was significantly higher ($P = 0.047$) at 20 mM (61.19 ± 3.34 g) compared to the control. Contrast analysis identified genotypes B33 and D56 as the most productive mutants, with yields of 125.44 g and 111.85 g, respectively. Principal component analysis extracted eighteen components, with the first four cumulatively explaining 50.60% of total variation. Biplot analysis of PC1 and PC2 captured all phenological traits, key seed traits, and yield attributes, highlighting the superior performance of B33 and D56. Cluster analysis partitioned the 190 genotypes into six groups, with B33 and D56 constituting distinct clusters. EMS mutagenesis effectively induced heritable phenotypic variation, with putative superior genotypes identified for advancement to M_2 and evaluation in replicated multi-environment trials toward the development of farmer- and consumer-preferred cowpea varieties.

Introduction

Cowpea (*Vigna unguiculata* [L.] Walp.) is a widely cultivated and nutritionally important legume, predominantly grown in tropical and subtropical regions, particularly in Sub-Saharan Africa, Asia, and Central and South America, while also being adapted to certain temperate zones such as the Mediterranean basin and the southern regions of the United States [1,2]. Cowpea (*Vigna unguiculata* L. Walp.), is an important staple food legume and cheap source of protein for many Africans in the low-land humid and dry savannah tropics. It is an excellent substitute for animal proteins by resource-poor people and vegetarians because of its high seed protein content (about 25%) and rich amino acids [3]. Indeed, some cultivars with seed protein content of about 30%, close to that obtained for soybean (*Glycine max*) have been reported [4,5]. Due to a decline in cowpea market value caused by pests, pathogens, and a lack of genetic variability, many scientists worldwide have sought to develop cowpea varieties that meet the taste and demand of a growing population [6-9]. Previous studies have demonstrated substantial genetic variability in cowpea for agronomic and economically important traits, including protein and mineral composition, underscoring the need to broaden the genetic base through breeding or mutagenesis [10,11]. The most noteworthy studies have used mutagens to increase genetic variability [6-9,12-14]. Ethyl Methane Sulfonate (EMS), one of the most effective mutagens, has been successfully used by several researchers in West Africa to improve genetic variability in cowpea [8,15].

A mutation is a heritable alteration in the structure or nucleotide sequence of a gene that arises suddenly and can be transmitted to subsequent generations. It results from changes in DNA such as substitutions, insertions, deletions, or rearrangements, and may occur naturally through replication or repair errors or be intentionally induced by physical and chemical mutagens. In plants, mutations can be fixed in both seed-propagated and vegetatively propagated species, allowing stable transmission of new traits. Consequently, mutations constitute a fundamental source of genetic variation and play a critical role in plant breeding

and crop improvement programs [16,17]. To create functional mutants in various crop species, a variety of chemical mutagens have been used. Artificial mutation induction through chemical mutagens, including colchicine (Col), ethyl methanesulfonate (EMS) and sodium azide (SA), has been widely recognized as an effective approach for enhancing genetic variability in plants. This technique facilitates the improvement of specific traits while preserving the inherent desirable attributes of the plant, as documented by several researchers [16]. Chemical mutagens generally produce induced mutations which lead to base pair substitutions, especially GC→AT resulting in amino acid changes, which change the function of proteins but do not abolish their functions as like deletions or frame shift mutations. These chemo mutagens induce a broad variation in morphological characters when compared to normal plants [18].

Ethyl methane sulfonate is a colorless mutagenic, carcinogenic and teratogenic chemical compound with the chemical formula $CH_3OSO_2C_2H_5$. It is formed from the condensation of ethanal with methane sulfonic acid [19]. Ethyl methane sulphonate is an alkylating chemical mutagen that has been widely utilized in plant breeding because it can generate a high frequency of gene mutations. EMS produces O_6-ethyl guanine by alkylating guanine residues. This matches thymine (T) but not cytosine (C) as a result, unrepaired alkylation replication occurs. A damaged pair A/T will effectively replace the G/C base pair. This mechanism foresees a strong G/C to A/T bond shortly [20]. According to [21,22], EMS is a monofunctional ethylating agent that has been reported to be mutagenic in a range of genetic test systems, ranging from viruses to mammals. Effectiveness and efficiency mean the extent to which the mutagenic agent produced a desired effect on the plant which may be positive or negative effect. [23] reported the order of effectiveness of chemical mutagens as hydrazine hydrate (HZ)> sodium azide (SA)> ethyl methane sulphonate (EMS) as they did a systematic and comparative study of induced chlorophyll mutation using HZ, SA and EMS and found that EMS treatments caused the highest frequency of chlorophyll mutations followed by

HZ and then SA. [23] & [24] reported that mutations of chemical mutagens and their effectiveness is dose-dependent and increase with the concentration of mutagens. [24] reported that lower concentrations of EMS are more effective than higher concentrations. Ethyl methane sulfonate is used by plant breeders because of its ease of use, good penetration, reproducibility, high mutation frequency and lack of disposal issues.

According to [6], plant breeders' major goal is to create crops that outperform existing cultivars in terms of production and quality, and this is dependent on the availability of genetic variation, preferably in the primary gene pool. Genetic variety is necessary for crop improvement. Plant breeders, therefore, have begun to use mutation breeding more frequently such as [13] who in their research induced variations using EMS in cowpea (*Vigna unguiculata* L. Walp Var. *Asontem*) in the M_1 and M_2 generations at different concentrations of EMS (0.0, 0.2, 0.4, 0.6, and 0.8%) for 16h which showed effective variations in traits such as germination and percentage survival, yield and yield related traits in M_1 population. [25] and [26] both subjected cowpea to EMS at different does for 6 hours, also reported higher number of pods per plant, early and late phenological traits, changes in pigmentation of leaf, pod and flower colour, plant height, increased pod length and high seed weights as a result of EMS-induced mutations. During the M_1 generation, only dominant or co-dominant mutations are easily detected by phenotypic observations [27]. Some viable mutations in the M_1 generation include reduction in plant height, pollen sterility, late or early flowering and podding. Furthermore, most previous studies have focused on later generations (M_2 and beyond), where mutations are more stably expressed, with fewer studies providing detailed analyses of variability in the M_1 generation. Understanding the immediate effects of EMS in the M_1 population is crucial for optimizing mutagenesis protocols and identifying early indicators of useful genetic variation.

Therefore, this study aimed to evaluate the effects of different EMS concentrations on seed germination and survival, phenological, yield-related and yield traits in the M_1 generation

of cowpea cultivar *'Wang Kae'*, with the objective of identifying putative superior genotypes for advancement to the M_2 generation and subsequent evaluation in replicated multi-environment trials. The ultimate goal was to support the development of improved cowpea varieties with farmer- and consumer-preferred traits for integration into breeding programs. By characterizing the phenotypic variability induced at the M_1 stage, this study contributes to refining EMS-based mutation breeding strategies in cowpea.

Materials & Methods

Experimental site, material and design

The field study was carried out at the Department of Plant and Environmental Biology, University of Ghana, from early April to July 2025. A total of 275 seeds of the '*Wang Kae*' cowpea variety (M_0 generation) were used in this study. Seventy-five seeds each were treated with three different concentrations of ethyl methane sulfonate (EMS): 20 mM, 40 mM, and 80 mM, and were soaked in the respective EMS solutions for six hours. Fifty seeds were soaked without treatment and served as the control (0 mM EMS). Genotypes were assigned alphanumeric labels to indicate both the EMS treatment level and the individual plant number. The alphabetic prefix represented the EMS concentration, while the numeric suffix denoted the individual plant within that treatment group. Specifically, '***A***' represented the control group (0 mM EMS), whereas '***B***', '***C***', and '***D***' represented the 20 mM, 40 mM, and 80 mM EMS concentrations, respectively. The numbers following each letter corresponded to the sequential numbering of plants within each treatment group. Thus, genotypes from the control (0 mM EMS) were labelled A1–A31, those treated with 20 mM EMS were labelled B1–B48, genotypes exposed to 40 mM EMS were designated C1–C55 and those treated with 80 mM EMS were labelled D1–D56.

The treated seeds were thoroughly washed in running water for 30 minutes to reduce the residual effect of the mutagen sticking to the seed coat. The treated seeds, along with the

control, were planted to raise the M_1 generation, and standard cultural practices were carried out thereafter. The experiment was designed in pots measuring 3.29 m^2. Spacing used was 0.8m between lines and 0.5 m between rows. One cowpea seed was planted per hole in each pot, at a depth of 3-4 cm.

Parameters studied in the M_1 generation

All traits studied were measured and/or counted using standard cowpea descriptor from [28]. The growth, yield-related and yield parameters studied in the M1 generation were grouped as phenological: days to germination (*dtg*), days to first flower (*dff*), days to 50% flowering (*d50f*), days to first harvest (*dfh*), days to 50% mature pod (*d50mp*); and yield-related traits: peduncle length at first harvest (*pel*), number of pods per peduncle (*npp*), number of pods per plant at maturity (*nptm*), number of seeds per pod (*nsp*), number of seeds per plant (*nspt*), pod length (*pdl*), pod width (*pdw*), number of locules (*ln*), percent seed abortion (*psa*). Seed dimensions included seed length (*sdl*), seed width (*sdw*) and seed thickness (*sdt*),while other parameters measured were seed weight (*swg*) and yield per plant (*yld*).

Percentage seed abortion was calculated as;

$$\frac{[(number\ of\ locules - number\ of\ seed\ per\ pod)]}{number\ of\ locules}\ x\ 100\% \ldots\ldots 1$$

Yield (g) was calculated as*:* $\frac{100\ seed\ weight\ (g)\ x\ number\ of\ seeds\ per\ plant}{100} \ldots\ldots 2$

Seed germination was recorded 7 days after planting. Survival plants were recorded at 50% flowering, and germination and survival rate were recorded using the formula by [29]

$$Germination\ rate\ (\%) =$$

$$\frac{(number\ of\ germinated\ seeds)}{(number\ of\ (un)treated\ seeds\ planted\ )}\ x\ 100 \ldots\ldots 3$$

$$Survival\ rate\ (\%)\ = \frac{(number\ of\ survived\ seedlings)}{(number\ of\ germinated\ seeds\ )}\ x\ 100 \ldots\ldots 4$$

[29]

Statistical analysis

Regression seedling germination and survival rate

XLSTAT was also used to generate the linear regression equation of seedling germination and survival rate.

Distribution and post hoc contrast analysis for the selection of putative traits and mutants'

Descriptive statistics, one-way ANOVA and post hoc contrast analysis were used to compare treatment effects for each trait in Jamovi 2.7.15.0. Plants within treatments whose values fell outside the control range for each character and were statistically significant from the control were selected as putative mutants**.**

Multivariate analysis for the verification of the selected putative mutant traits and genotypes

Principal Component Analysis (PCA) was performed, accompanied by biplot analysis in Jamovi 2.7.15.0. Principal Component Analysis for quantitative traits was employed to determine the percentage contribution of each trait to total genetic variation. The principal components (PCs) with eigenvalues >1 were selected and traits with coefficients >0.3 were considered to have significantly contributed to the variability. The biplot of computed similarity values was used to visualize relationships among traits across EMS treatment groups and genotypes, to assess the distribution of treatment groups, examine trait vector orientations and identify putative mutants based on their displacement from the control.Cluster analyses were performed using Jamovi 2.7.15.0 to group genotypes within treatments using the Ward D2 hierarchical clustering optimization criterion. A circular dendrogram constructed from computed similarity values was used to visualize relationships among genotypes and traits that differed significantly among treatments, with the resulting clusters interpreted to characterize the phenotypic groupings produced by mutagenic treatment.

Results

Percentage germination and survival of M_1 plants

The results for seed germination and survival rates (%) and linear regression fits for M_1 cowpea genotypes subjected to three EMS doses for 6 h are presented in Fig 1. The highest seed germination rate (89.33%) and seedling survival rate (74.67%) were recorded at 80 mM, whereas the lowest germination rate (70.00%) and seedling survival rate (62.00%) were observed in the 0 mM (control) treatment. The percentages of seed germination and seedling survival were in this order: 0 mM < 20 mM < 40 mM < 80 mM. Linear regression analysis revealed a strong positive relationship between EMS concentration and both seed germination and survival rate (Fig 1). For seed germination rate, the regression equation was $y = 6.47x + 65.33$ with an R^2 value of 0.94. Seedling survival showed a positive linear relationship with EMS concentration as follows: $y = 4.73x + 56.67$ with an R^2 value of 0.90.

Fig 1: Seed germination and survival rates (%) and linear regression fits for M_1 cowpea genotypes subjected to three ethyl methanesulfonate (EMS) doses for 6 h.

Contrast analysis of traits in the M_1 generation

Phenological traits

***Days to germination* (*dtg*)** differed significantly among treatments ($P < 0.001$). The control (0 mM) germinated earliest, with a mean of 3.45 ± 0.13 days and a range of 2–5 days. All EMS treatments delayed germination, with the most substantial effect at 20 mM, while 40 and 80 mM showed delayed but comparable responses. The frequency of putative mutants was 50.0% at 20 mM, 29.10% at 40 mM, and 30.36% at 80 mM (Table 1). Selected putative mutants can be found in S1 Table.

Table 1 : Mean, variability, mutant frequency, and pairwise significance of phenological traits in the M_1 generation of cowpea under different EMS concentrations.

Trait	N	Trt	Mean±se	%CV	Min	Max	Freq	P	T-values of significant pairwise comparisons			
									0mM	20mM	40mM	80mM
dtg	31	0mM	3.45±0.13$_a$	20.90	2	5		***		-6.38***	-4.53***	-4.50***
	48	20mM	5.38±0.20$_b$	25.30	3	7	50.00		-6.38***			
	55	40mM	4.78±0.19$_b$	29.20	3	7	29.10		-4.53***			
	56	80mM	4.77±0.19$_b$	29.70	3	7	30.36		-4.50***			
dff	31	0mM	37.87±0.77$_a$	11.30	30	45		***		-5.00***	-5.40***	-4.97***
	48	20mM	44.69±0.91$_b$	14.10	33	54	51.17		-5.00***			
	55	40mM	45.04±0.86$_b$	14.10	34	55	47.27		-5.40***			
	56	80mM	44.46±0.79$_b$	13.30	33	55	41.07		-4.97***			
d50f	31	0mM	57.84±0.92$_a$	8.90	48	68		***		-4.51***	-4.98***	-4.72***
	48	20mM	64.67±0.99$_b$	10.70	50	75	37.50		-4.51***			
	55	40mM	65.18±0.97$_b$	11.00	49	80	40.00		-4.98***			
	56	80mM	64.79±0.85$_b$	9.80	53	80	28.57		-4.72***			
dfh	31	0mM	49.35±1.15$_a$	13.00	40	67		***		-6.93***	-6.93***	-6.04***
	48	20mM	60.17±0.94$_b$	10.80	48	72	18.75		-6.93***			
	55	40mM	58.93±0.97$_b$	12.10	38	72	9.100		-6.93***			
	56	80mM	58.50±0.91$_b$	11.70	43	72	12.50		-6.04***			
d50mp	31	0mM	79.48±1.41$_a$	9.90	66	94		***		-5.40***	-4.75***	-4.59***
	48	20mM	90.17±1.37$_b$	10.50	68	107	39.58		-5.40***			
	55	40mM	88.65±1.09$_b$	9.10	68	106	25.45		-4.75***			
	56	80mM	88.32±1.16$_b$	9.80	69	110	23.21		-4.59***			

Note. Means sharing the same superscript letter within a trait are not significantly different according to Tukey's HSD test (P ≤ 0.05). Trt= Treatment, N=Sample size, Mean ± SE = Mean plus or minus standard error, %CV= Percentage coefficient of variation, Min= minimum, Max= maximum, Freq= Percentage frequency of putative mutants; P=Probability value. Traits: dtg = days to germination, dff = days to first flower, d50f = days to 50% flowering, dfh = days to first harvest, d50mp = days to 50% mature pods, *=significance at 5% level, **=significance at 1% level, ***=significance at 0.1% level

Days to first flower (dff) were significantly affected by EMS across treatments (P < 0.001). *DFF* was the earliest in the control, with a mean of 37.87 ± 0.77 days and a range of 30 to 45 days, and was progressively delayed with increasing EMS concentration, reaching a maximum mean of 45.04 ± 0.86 days at 40 mM. Mutant frequencies declined from 51.17% at 20 mM to 41.07% at 80 mM (Table 1). Selected putative mutants can be found in S1 Table.

Days to 50% flowering (d50f) were significantly affected by EMS across treatments (P < 0.001). *D50f* increased from a control mean of 57.84 ± 0.92 days, with values ranging from 48 to 68 days, to mean values of approximately 65 days across EMS treatments, with broader range of 49–80 days. Corresponding mutant frequencies were 37.50%, 40.00%, and 28.57% at 20, 40, and 80 mM, respectively (Table 1). Selected putative mutants can be found in S1 Table.

Days to first harvest (dfh) were the earliest in the control, with a mean of 49.35 ± 1.15 days and a range of 40–67 days and were significantly (P < 0.001) delayed in all EMS treatments. Mean dfh increased to 60.17 ± 0.94 days at 20 mM, 58.93 ± 0.97 days at 40 mM,

and 58.50 ± 0.91 days at 80 mM. Mutant frequencies were 18.75% at 20 mM, 9.10% at 40 mM, and 12.50% at 80 mM (Table 1). Selected putative mutants can be found in S1 Table.

A similar trend was observed for days to ***50% maturity of pods (d50mp)***, with the control recording the earliest maturity at 79.48 ± 1.41 days, and values ranging from 66 to 94 days. EMS treatments significantly (P<0.001) delayed pod maturity compared with the control, with mean d50mp values of 90.17 ± 1.37 days at 20 mM, 88.65 ± 1.09 days at 40 mM, and 88.32 ± 1.16 days at 80 mM. The frequency of selected mutants for d50mp was 39.58%, 25.45%, and 23.21% at 20, 40, and 80 mM, respectively (Table 1). Selected putative mutants can be found in S*1* Table.

Yield-related and yield traits

Pod length (pdl) was highest in the control (0 mM), with a mean of 14.65 ± 0.31 cm and a range of 11.55–17.90 cm, whereas 80mM recorded the lowest mean pdl of 13.52±0.28 cm, which was significantly different (P=0.008) from the control. Based on deviations from the control range, the frequency of selected mutants for pdl was 10.42%, 9.10%, and 21.43% in 20 mM, 40 mM, and 80 mM, respectively (Table 2). Selected putative mutants can be found in S1 Table.

Number of locules per pod (ln) differed significantly among EMS treatments (P < 0.001). The control recorded a mean of 17.00 ± 0.60, with a range of 12 to 24 locules. Higher EMS concentrations resulted in a significant reduction in ln: 14.93 ± 0.48 locules at 40 mM and 11.41 ± 0.39 locules at 80 mM. Pairwise comparisons showed that only the 80 mM treatment differed significantly (P < 0.001). From both the control and all other treatments, Phenotypic variability increased with EMS dose, as reflected by a rise in the coefficient of variation from 17.5% at 20 mM to 25.8% at 80 mM. Based on deviations from the control range, the frequency of putative mutants for ln reached 20.0% at 40 mM and 50.0% at 80 mM (Table 2). Selected putative mutants can be found in S1 Table.

Table 2: Mean, variability, mutant frequency, and pairwise significance of yield-related and yield traits in the M_1 generation of cowpea under different EMS concentrations

Trait	Trt	N	Mean±se	%CV	Min	Max	Freq	P	T-values of significant pairwise comparisons			
									**0mM**	**20mM**	**40mM**	**80mM**
npp	0mM	31	3.84±0.17	25.30	2.00	5.00		ns				
	20mM	48	4.02±0.13	21.50	2.00	5.00						
	40mM	55	3.82±0.13	25.70	2.00	5.00						
	80mM	56	3.70±0.16	31.80	2.00	5.00						
nptm	0mM	31	24.68±1.31	29.50	14.00	44.00		ns				
	20mM	48	25.46±1.07	29.10	10.00	45.00						
	40mM	55	23.25±0.78	25.00	11.00	44.00						
	80mM	56	23.87±0.97	30.40	10.00	46.00						
nsp	0mM	31	9.84±0.63	35.60	5.00	15.00		ns				
	20mM	48	9.92±0.46	32.30	5.00	15.00						
	40mM	55	10.44±0.39	27.80	5.00	15.00						
	80mM	56	9.80±0.46	35.10	5.00	15.00						
nspt	0mM	31	233.10±14.38	34.30	100.00	406.00		ns				
	20mM	48	246.29±13.29	37.40	100.00	450.00						
	40mM	55	238.20±10.34	32.20	100.00	435.00						
	80mM	56	226.36±12.17	40.20	100.00	450.00						
pdl	0mM	31	14.65±0.31$_{b}$	11.80	11.55	17.86		*				2.66**
	20mM	48	14.34±0.25$_{ab}$	12.30	10.50	19.88	10.42					
	40mM	55	13.83±0.26$_{ab}$	14.10	6.95	16.75	9.10					
	80mM	56	13.52±0.28$_{a}$	15.20	8.45	17.50	21.43		2.66**			
pdw	0mM	31	0.85±0.01	9.70	0.70	1.03		ns				
	20mM	48	0.89±0.02	16.50	0.61	1.23						
	40mM	55	0.90±0.03	25.60	0.52	1.28						
	80mM	56	0.83±0.02	18.00	0.60	1.13						
ln	0mM	31	17.00±0.60$_{c}$	19.70	12.00	24.00		***			2.87*	7.77***
	20mM	48	17.06±0.43$_{c}$	17.50	12.00	23.00					3.67**	8.94***
	40mM	55	14.93±0.48$_{b}$	23.90	8.00	21.00	20.00		2.87*	3.67**		5.77***
	80mM	56	11.41±0.39$_{a}$	25.80	7.00	23.00	50.00		7.77***	8.94***	5.77***	
psa	0mM	31	14.27±1.93	75.30	0.00	35.71		ns				
	20mM	48	18.60±1.68	62.70	0.00	35.71						
	40mM	55	19.35±1.66	63.80	0.00	35.71						
	80mM	56	15.43±1.53	74.20	0.00	35.71						
sdl	0mM	31	7.44±0.24$_{a}$	18.10	5.68	11.59		***		-5.19***	-9.41***	-10.10***
	20mM	48	8.77±0.25$_{b}$	19.60	6.10	11.65	0.42		-5.19***		-4.64***	-5.40***
	40mM	55	9.79±0.07$_{c}$	4.90	8.10	11.05			-9.41***	-4.64***		
	80mM	56	9.95±0.09$_{c}$	6.60	7.95	11.60	1.79		-10.1***	-5.4***		
sdw	0mM	31	4.78±0.10$_{a}$	11.90	3.94	6.08		***		-3.44**	-3.52**	-4.63***
	20mM	48	5.14±0.09$_{b}$	11.80	4.05	6.44	0.42		-3.44**			
	40mM	55	5.14±0.04$_{b}$	5.90	4.58	5.69			-3.52**			
	80mM	56	5.25±0.05b	7.00	4.28	6.11	1.79		-4.63***			

Note. Means sharing the same superscript letter within a trait are not significantly different according to Tukey's HSD test ($P \leq 0.05$). Trt= Treatment, Mean ± SE = Mean plus or minus standard error, %CV= Percentage coefficient of variation, Min= minimum, Max= maximum, Freq= Percentage frequency of putative mutants; P=Probability value. Traits: npp = number of pods per peduncle, nptm = number of pods per plant at maturity, nspt = number of seeds per plant, pdl = pod length, pdw = pod width, ln = number of locules, nsp = number of seeds per pod, psa = percent seed abortion, sdl = seed length, sdw = seed width, sdt = seed thickness, swgt = seed weight, yld = yield per plant, *=significance at 5% level, **=significance at 1% level, ***=significance at 0.1% level

Table 2: Mean, variability, mutant frequency, and pairwise significance of yield-related and yield traits in the M_1 generation of cowpea under different EMS concentrations (cont'd)

Trait	Trt	N	Mean±se	%CV	Min	Max	Freq	P	T-values of significant pairwise comparisons			
									0mM	20mM	40mM	80mM
sdt	0mM	31	$4.09 \pm 0.07_a$	9.80	3.29	4.92		***		-2.82*		
	20mM	48	$4.35 \pm 0.08_b$	12.20	3.31	5.19	12.50		-2.82*		4.92***	3.35**
	40mM	55	$3.95 \pm 0.04_a$	8.50	3.43	5.59	1.82			4.92***		
	80mM	56	$4.08 \pm 0.05_a$	8.90	3.41	4.94	1.79			3.35**		
swg	0mM	31	$21.03 \pm 0.34_a$	9.10	17.82	24.87		***		-6.91***	-6.18***	-2.92*
	20mM	48	$24.86 \pm 0.34_c$	9.40	17.81	29.99	54.17		-6.91***			4.77***
	40mM	55	$24.37 \pm 0.35_c$	10.70	17.70	28.39	49.10		-6.18***			3.87***
	80mM	56	$22.60 \pm 0.33_b$	11.00	17.35	28.68	16.07		-2.92*	4.77***	3.87***	
yld	0mM	31	$48.79 \pm 3.01_a$	34.40	20.61	85.68		*		-2.61*		
	20mM	48	$61.19 \pm 3.34_b$	37.80	18.70	125.44	16.67		-2.61*			
	40mM	55	$58.02 \pm 2.59_{ab}$	33.10	19.97	105.43	12.72					
	80mM	56	$51.16 \pm 2.87_{ab}$	42.00	18.45	111.85	10.71					

Note. Means sharing the same superscript letter within a trait are not significantly different according to Tukey's HSD test ($P \leq 0.05$). Trt= Treatment, Mean ± SE = Mean plus or minus standard error, %CV= Percentage coefficient of variation, Min= minimum, Max= maximum, Freq= Percentage frequency of putative mutants; P=Probability value. Traits: npp = number of pods per peduncle, nptm = number of pods per plant at maturity, nspt = number of seeds per plant, pdl = pod length, pdw = pod width, ln = number of locules, nsp = number of seeds per pod, psa = percent seed abortion, sdl = seed length, sdw = seed width, sdt = seed thickness, swgt = seed weight, yld = yield per plant, *=significance at 5% level, **=significance at 1% level, ***=significance at 0.1% level

Seed length (sdl) varied significantly across EMS treatments (P < 0.001). The control recorded the lowest mean seed length, with a value of 7.44 ± 0.24 mm, with a range of 5.68 to 11.59 mm and a CV of 18.1%. EMS treatment groups resulted in a progressive increase in seed length, with means of 8.77 ± 0.25 mm at 20 mM, 9.79 ± 0.07 mm at 40 mM, and 9.95 ± 0.09 mm at 80 mM. Pairwise comparisons showed that all EMS treatments differed significantly from the control (P < 0.001), while the 40 mM and 80 mM treatments did not differ from each other. Based on deviations from the control range, mutant frequencies were low for seed length recorded at 0.42% for 20 mM and 1.79% for 80 mM (Table 2). Selected putative mutants can be found in S1 Table.

Seed width (sdw) was also significantly (P < 0.001) affected by EMS treatment. The control population had a mean width of 4.78 ± 0.10 mm, ranging from 3.94 to 6.08 mm, with a CV of 11.9%. All EMS treatments differed significantly from the control (P < 0.001). Variability declined at higher EMS doses, with CV values as low as 5.9% at 40 mM.

Frequencies of putative mutants increased with EMS concentration, reaching 0.42% at 20 mM and 1.79% at 80 mM (Table 2). Selected putative mutants can be found in S1 Table.

Seed thickness (sdt) differed significantly among treatments ($P < 0.001$). The control recorded a mean thickness of 4.09 ± 0.07 mm with values ranging from 3.29 to 4.92 mm. Thickness increased significantly ($P=0.027$) at 20 mM with a mean of 4.36 ± 0.08 mm. Pairwise comparisons indicated significant differences between 20 mM and both 40 mM ($P < 0.001$) and 80 mM ($P = 0.005$). Mutant frequencies for seed thickness were highest at 20 mM (12.5%) but remained low at 40 mM (1.82%) and 80 mM (1.79%) (Table 2). Selected putative mutants can be found in S1 Table.

100-Seed weight (swg) showed highly significant variation among EMS treatments ($P < 0.001$). The control recorded a mean of 21.03 ± 0.34 g, with a range of 17.82 to 24.87 g. EMS treatment increased swg, with the highest means observed at 20 mM (24.86 ± 0.34 g) and 40 mM (24.37 ± 0.35 g), both of which differed significantly from the control ($P < 0.001$). The 80 mM treatment weighed 22.60 ± 0.33 g and showed an intermediate response, differing considerably from both the control ($P = 0.02$) and lower EMS doses ($P < 0.001$). Frequencies of putative mutants were highest at 20 mM (54.17%), followed by 40 mM (49.1%) and 80 mM (16.07%) (Table 2). Selected putative mutants can be found in S1 Table.

Yield per plant (yld) differed significantly among treatments ($P = 0.018$). The control population recorded the lowest mean yield of 48.79 ± 3.01 g, with a range of 20.61 to 85.68 g. Yield increased significantly ($P = 0.047$) at 20 mM, with a mean of 61.19 ± 3.34 g, which differed from the control ($P = 0.047$). The 40 mM (58.02 ± 2.59 g) and 80 mM (51.16 ± 2.87 g) treatments, which exhibited intermediate yields, did not differ significantly from either the control or the 20 mM treatment. Phenotypic variability was high across treatments, with a CV of 33.10–42%, and mutant frequencies declined with increasing EMS concentration, from

16.67% at 20 mM to 10.71% at 80 mM (Table 2). Selected putative mutants can be found in S1 Table.

Multivariate Analysis of Phenological and Yield-Related Traits

PCA Biplot of phenological, yield-related and yield traits

Variance Explained by Principal Components. Principal component analysis (PCA) of the traits and genotypes of plant observations across EMS treatments yielded 8 principal components with eigenvalues < 1 (S2 Table). The first four principal components accounted for a total variability greater than 50.60% in all treatments. The first two principal components (Dim1 and Dim2) together explained 32.8% of the total phenotypic variation, with PC1 accounting for 17.7% and PC2 accounting for 15.1% (Fig 2, S2 Table) with an eigenvalues of 3.18 and 2.71 respectively.

Fig 2: PCA biplot of cowpea genotypes and traits based on PC1 and PC2. (a)Genotypes treated with 0-, 20-, 40- and 80-mM EMS are labelled A01–A31, B01–B48, C01–C55 and D01–D56, respectively. (b) Vectors indicate trait loadings, and points represent genotype distribution. (c) dtg= days to germination, dff = days to first flower, d50f = days to 50% flowering, dfh = days to first harvest, d50mp = days to 50% mature pods, npp = number of pods per peduncle, nptm = number of pods per plant at maturity, nspt = number of seeds per plant, pdl = pod length, pdw = pod width, ln = number of locules, nsp = number of seeds per pod, psa = percent seed abortion, sdl = seed length, sdw = seed width, sdt = seed thickness, swgt = seed weight, yld = yield per plant

Distribution of EMS Treatment Groups in the PCA Biplot.The PCA biplot (Fig 2) plotted genotype scores on PC1 (Dim1, 17.7%) against PC2 (Dim2, 15.1%). Control genotypes (A01–A31, 0 mM) clustered predominantly on the negative side of PC1, spanning approximately −5.0 to −0.5, with limited spread along PC2. Genotypes from the 20 mM treatment (B01–B48) occupied a broader area, with partial overlap with the control cluster and moderate displacement toward positive PC1 values. Genotypes from the 40 mM treatment (C01–C55) were more widely dispersed across both axes, with a concentration in the central to positive PC1 region. Genotypes from the 80 mM treatment (D01–D56) showed the widest

distribution across the biplot, with the greatest displacement toward positive PC1 and the greatest scatter along PC2 of any treatment group.

Biplot Trait Vector Orientations. Vectors for *dff*, *d50f*, *dfh*, and *d50mp* projected horizontally toward the positive PC1 axis. Vectors for sdl and sdw were oriented toward the lower-right quadrant (positive PC1, negative PC2). The *swg* vector projected toward the positive region of both PC1 and PC2. Vectors for *yld*, *nspt*, and *nsp* were directed upward toward positive PC2, with *yld* and nspt nearly collinear. The *nptm* vector was oriented downward toward negative PC2. Vectors for *pdl* and *ln* projected toward the upper-central region of the biplot. The angle between the *sdl*/*sdw* vectors and the *yld*/*nspt*/*nsp* vectors was approximately perpendicular (Fig 2).

Identification and Selection of Putative Mutants from the Biplot.Based on positional alignment with trait vectors of agronomic interest, three classes of putative mutants were identified in Fig 2. Eleven genotypes positioned in the upper biplot region with positive PC2 scores, in alignment with the *yld*, *nsp*, *nspt*, and *swg* vectors, were classified as high-yielding putative mutants: **B33, B28, B22, B31, D56, D45, D25, C5, C41, B26, and B21**. Eleven genotypes positioned along the negative PC2 axis in alignment with the *sdl* and *sdw* vectors, showing above-average seed dimensions with reduced seed number and yield, were classified as large-seed putative mutants: **D23, D7, D34, D47, C37, C39, C40, C42, B18, B11** and **B8**.

Two genotypes positioned on the extreme negative PC1 side, in the direction opposite to the phenological delay vectors, were classified as early-phenology putative mutants: **B19** and **C2**. Four genotypes **B9, B7, C46 and D20** were positioned at the extreme positive PC1 end with weak alignment to yield vectors and were classified as severely delayed and low performing. These genotypes were not selected for advancement but were retained as extreme phenotypic reference individuals.

Cluster analysis of phenological and yield-related traits

Hierarchical cluster analysis of 18 phenological and yield-related traits across 190 M_1 cowpea genotypes using Ward's linkage method resolved six distinct clusters (Fig 3, S1 Table). The six clusters contained 64, 24, 31, 20, 40, and 9 genotypes, respectively.

Fig 3: Dendrogram of hierarchical cluster analysis of M_1 cowpea genotypes based on quantitative traits using Ward's linkage method.
(a)Genotypes treated with 0-, 20-, 40-, and 80-mM EMS are labelled A, B, C, and D, respectively. (b) Six clusters are delineated by colour: Cluster 1 (green, n = 64); Cluster 2 (brown, n = 24); Cluster 3 (grey, n = 31); Cluster 4 (forest green, n = 20); Cluster 5 (blue, n = 40); Cluster 6 (red, n = 9).

Cluster 1 (n = 64). Cluster 1 contained predominantly EMS-treated genotypes, with 35.42%, 49.10%, and 33.93% of the 20 mM, 40 mM, and 80 mM treatment groups represented (Fig 3, S1 Table). Only one control genotype (A01) was assigned to this cluster, accounting for 3.22% of the 0 mM population. Days to 50% flowering ranged from 53 to 80 days across cluster members, with the longest delays recorded in C41 and C43. Days to 50% pod maturity ranged from 69 to 109 days, with the most extended values recorded in D43 and C08. Seed weight ranged from 18.82 g (D04) to 28.68 g (D56). Yield per plant ranged from 31.45 g (C37) to 111.85 g (D56). Putative mutants selected from this cluster included D56 (111.85 g), D45 (*106.09 g*), D25 (*102.66 g*), B31 (*107.22 g*), B28 (*103.50 g*), B22 (*88.96 g*), C41 (*90.96 g*) and C5 (*105.43 g*).

Cluster 2 (n = 24). Cluster 2 was dominated by control genotypes, which accounted for 61.29% of the 0 mM population (Fig 3, S1 Table). Representation from EMS treatments was low: 2.08% (20 mM), 3.64% (40 mM), and 3.57% (80 mM). Genotypes in this cluster had the earliest germination, flowering, and maturity values across the full population, with yield levels comparable to the control mean and low within-cluster variability. EMS-treated genotypes assigned to this cluster included C02, with days to first harvest of 38 days, and C14, with days to first harvest of 39 days. Both were identified as early-phenology putative mutants.

Cluster 3 (n = 31). Cluster 3 contained genotypes primarily from the high-dose EMS treatments, with 28.57% of the 80 mM population represented (Fig 3, S1 Table). Genotypes in this cluster showed delayed phenological development, reduced locule number, and low yield per plant. Representative genotypes included B7, B9, C46, and D20, all of which showed negative deviation from the control range across multiple yield traits. These genotypes were not selected for advancement.

Cluster 4 (n = 20). Cluster 4 included genotypes from all three EMS treatment groups: 10.42% of 20 mM, 9.09% of 40 mM, and 12.50% of 80 mM (Fig 3, S1 Table). Genotypes in this cluster combined relatively early days to first flower and days to 50% flowering with extended days to first harvest and days to 50% mature pods. Seed weight and yield per plant were elevated relative to the control in several members. The highest-yielding genotype in this cluster was B33 (125.44 g), followed by B19 (93.59 g). Both substantially exceeded the control population range for yield per plant while retaining comparatively early flowering phenology — B19 at 36 days to first flower and B33 at 37 days.

Cluster 5 (n = 40). Cluster 5 comprised exclusively EMS-treated genotypes, with 33.33% of the 20 mM population, 22.27% of the 40 mM population, and 16.07% of the 80 mM population assigned to this cluster (Fig 3, S1 Table). No control genotypes were present. Genotypes in this cluster showed moderately delayed phenological development and high seed weight but reduced locule number per pod, which was associated with lower yield per plant relative to the control mean.

Cluster 6 (n = 9). Cluster 6 was the smallest cluster, containing 12.50% of the 20 mM population and 5.36% of the 80 mM population (Fig 3, S1Table). No control or 40 mM genotypes were present. Genotypes in this cluster showed moderately delayed phenological development, large seed dimensions, and high seed weight. Seed number per pod was relatively low, but total yield per plant was elevated, driven by seed weight. Putative mutants selected

from this cluster included B07 (yld = 67.18 g, swg = 29.99 g) and D36 (yld = 74.69 g, swg = 28.29 g), both of which exceeded the control mean for seed weight.

Discussion

Percentage germination and survival of M1 plants

The EMS concentrations of 20, 40, and 80 mM and the fixed 6 h exposure duration were selected to provide a graded mutagenic treatment range while maintaining sufficient plant survival for evaluation, consistent with previous studies showing that EMS response depends on both concentration and exposure time [13,30,31]. In the present study, LD_{50} was not achieved, as seed germination and seedling survival increased with increasing EMS concentration. Although most EMS mutagenesis studies report a decline in germination and survival with increasing dose [16,32-34], several studies have documented non-linear or hormetic responses, where low to moderate EMS doses stimulate germination and early growth before inhibitory effects appear at higher concentrations. In legumes, responses similar to the present study have been reported. For instance, in cowpea relatives, [35] observed in faba bean (*Vicia faba* L.) that six varieties exhibited higher germination and survival at 0.05% EMS compared to the control, followed by a decline at higher concentrations. This hormesis-like response mirrors the increasing germination trend observed in the present study. Similarly, in soybean (*Glycine max* L.), [36] reported that EMS treatments at 0.1–0.2% improved germination and early seedling growth relative to untreated seeds, with inhibitory effects only becoming evident at higher doses. In cucumber (*Cucumis sativus* L.), [37] demonstrated that post-treatment handling influenced germination outcomes, with a sodium thiosulfate rinse yielding higher germination (84.4%) than a water rinse (80.0%) at 0.5% EMS, indicating that detoxification and recovery processes can enhance germination performance. Likewise, in tef (*Eragrostis tef*), [38] reported that a treatment combination of 2.50% EMS for 4 h resulted in significantly higher germination (77.1%) compared to lower doses, emphasizing the

importance of genotype × dose × exposure-time interactions. In barley (*Hordeum vulgare* L.), [39] also noted a non-monotonic response, where low EMS exposure improved germination relative to some treatments, although optimal survival occurred at intermediate doses. Collectively, these studies support the interpretation that the increasing germination and survival rates observed with rising EMS concentration in the present study may reflect a hormetic or priming-like effect, rather than a deviation from expected mutagenic behavior. This reinforces the role of genotype-specific responses and treatment conditions in shaping EMS-induced physiological outcomes.

Our results differs from the findings of [16, 32-34], [32] reported an apparent dose-dependent reduction in germination and survival in the M_1 generation of cowpea varieties *Pusakomal* and *V-240 (Rambha)* following EMS treatments at 0.1–0.3%, with control germination exceeding 95% and higher doses approaching the LD_{50} threshold. Similarly, [14] observed a strong negative linear relationship between EMS concentration and germination in cowpea and identified an optimal LD_{50} dose at 0.4% EMS. The contrast observed in this current study was that germination and survival were lowest in the control. It increased with EMS concentration, indicating that LD_{50} expression is highly dependent on genotype, EMS dose range, and exposure duration. Unlike previous studies that employed percentage-based EMS concentrations and different treatment protocols, the present study used millimolar EMS concentrations with a fixed 6-h exposure, which may have altered the physiological response. In addition, the relatively low germination and survival recorded in the control suggest baseline establishment constraints that may have obscured EMS-induced lethality. These findings indicate that the absence of an LD_{50} endpoint in the present study does not imply insufficient mutagenic effect. Instead, it underscores the importance of genotype × dose × exposure-time interactions in EMS mutagenesis. [40,41]. Such responses have been linked to priming-like effects, where mild stress exposure improves germination performance [40].

Contrast analysis of traits in the M_1 generation

Effects of EMS Mutagenesis on Phenological traits

Effect of EMS on Days to Germination.The present study revealed that EMS treatment significantly delayed days to germination (*dtg*) in the M_1 generation of cowpea (*Vigna unguiculata* (L.) Walp). The control seeds germinated earliest, while all EMS-treated groups exhibited delayed germination. This pattern of EMS-induced germination delay is consistent with the well-established understanding that chemical mutagens impose physiological stress during seed imbibition, activating DNA repair mechanisms that temporarily arrest the cell cycle until lesions are repaired [31]. The alkylating activity of EMS, which produces O^6-ethylguanine and other base modifications, necessitates repair via base excision repair pathways before the resumption of cell division in the embryonic axis [21]. This repair process delays radicle emergence, resulting in a measurable increase in days to germination.

Comparable delays in days to germination have been documented in other crops. Similarly, research on marigold (*Tagetes* spp.) demonstrated that higher EMS concentrations and longer exposure times reduced germination rate and delayed progress, although the effect was largely inhibitory rather than showing a peak delay at a lower dose [42]. The high frequency of putative mutants for *dtg* at 20 mM (50.0%) compared to 40 mM (29.1%) and 80 mM (30.4%) aligns with the observation that lower EMS doses often yield a higher proportion of viable mutants because they cause less overall lethality and permit the expression of a wider range of phenotypic variation [23,24]. In cowpea, [13] similarly found that lower EMS doses produced useful variability while higher doses reduced survival and limited the number of recoverable mutants. The reduction in mutant frequency at higher concentrations in our study may reflect the increased lethality and physiological damage that suppress the recovery of germination-related mutants.

Effects of EMS on Days to First Flower. In the present study, EMS treatment at all three concentrations caused a highly significant delay in *dff* relative to the control. Pairwise comparisons confirmed that all EMS treatments differed significantly from the control, while no significant differences were detected among the three EMS concentrations themselves. This pattern suggests a threshold effect, where even the lowest dose was sufficient to disrupt normal floral transition, with no further meaningful delay introduced at higher concentrations. The delayed onset of flowering observed here aligns with findings reported in other EMS mutagenesis studies on cowpea. [26] documented early and late maturity as among the viable mutant classes induced by EMS in *Vigna unguiculata*, noting that EMS can modify important components of plant cells and affect the morphology, anatomy, biochemistry, and physiology of plants differentially depending on concentration level. Similarly, [14] reported effects of EMS, DES, and SA on quantitative traits of cowpea in the M_1 generation, providing early evidence that flowering-related traits are susceptible to chemical mutagenesis in this species.

The mechanism underlying EMS-induced delays in flowering time is most likely attributable to point mutations disrupting key genes involved in floral transition pathways. [31] noted that M_1 plants subjected to EMS mutagenesis have shown variation in flower organs and delayed flowering, consistent with the disruption of genes regulating the floral transition, and that such phenotypic changes in flower-related traits may reflect the broad mutagenic spectrum of EMS across multiple developmental pathways. In cowpea specifically, flowering time is known to be a quantitatively inherited and environmentally sensitive trait. [43] demonstrated that time to flowering in cowpea is strongly influenced by photoperiod, with photoperiod-sensitive genotypes under long natural days exhibiting considerable delays in floral development. EMS-induced mutations affecting photoperiod perception genes or floral integrators such as *GIGANTEA*, *CONSTANS*, or *FLOWERING LOCUS T* homologs could

therefore plausibly extend the time to first flower, even at relatively low mutagen concentrations.

The mutation frequencies observed for days to first flower as 51.17%, 47.27%, and 41.07% for 20, 40, and 80 mM, respectively showed a declining trend with increasing EMS concentration. This is consistent with the well-documented inverse relationship between mutagen dose and the proportion of viable, phenotypically expressed mutations, where higher doses tend to induce increasingly lethal or phenotypically cryptic mutations rather than productive, viable phenotypic variants. [44] reported a similar pattern in cowpea var. Arka Garima, where a linear decline in morphological mutation frequency was observed with increasing EMS concentration, suggesting that higher doses induce proportionally more deleterious rather than viable mutations.

Effects of EMS on days to 50% flowering. In this study, we observed that the pairwise comparisons confirmed that all mutagenized populations differed significantly from the control but not from each other, reinforcing the threshold dose interpretation. The coefficient of variation was relatively low across all groups (8.9–11%), indicating that although the mean was shifted by EMS treatment, population-level variation in the timing of 50% flowering remained moderate. Notably, the range of days to 50% flowering was broader in the EMS treatments (50–80 days) than in the control (48–68 days), reflecting the expanded phenotypic diversity generated by mutagenesis. These findings are in agreement with results reported from induced mutagenesis experiments in related *Vigna* species and cowpea populations. For instance, [45] evaluated M_5 Tswana cowpea mutant lines across two seasons reported significant variation in days to 50% flowering, with the Tswana control consistently flowering earlier than many of the mutant lines and noted that delayed flowering in mutagenized populations may be attributed to genetic changes created by the mutation that affect normal flowering mechanisms.

In EMS-mutagenized cowpea (cv. *Asontem*) evaluated in M_2 populations, [46] similarly reported wide distribution of days to flowering among mutagenized individuals compared to the wild type, demonstrating that EMS is effective in generating significant phenotypic variation in flowering-related quantitative traits in cowpea. The parallel shifts in both days to first flower and days to 50% flowering across all EMS treatments suggested that EMS disrupted the floral initiation process rather than the speed of floral development once initiated. If the mutagen had primarily affected floral development after initiation, one would expect a disproportionate shift in days to 50% flowering without a corresponding shift in days to first flower. The approximately equal delays of 6–7 days in both traits across all treatments instead point to a disruption of the upstream signaling or perception pathways governing the transition from vegetative to reproductive growth. Research on contrasting cowpea genotypes by [47] established that delays in flowering are primarily driven by delays in the first appearance of floral buds rather than in bud development itself, supporting the interpretation that the mutagenic effects observed here act at or upstream of bud initiation.

The observations in this study were made in the M_1 generation, where induced mutations were expected to be heterozygous, and chimerism may confound phenotypic expression. [46] noted that during the M_1 generation, only dominant or co-dominant mutations are easily detected by phenotypic observation, as the functional mutations induced by EMS through point mutation have a greater chance of being dominant or co-dominant. The delayed flowering documented in this study likely reflected the expression of dominant or semi-dominant mutation effects on floral pathway genes, while recessive mutations which may represent early or altered flowering phenotypes of greater breeding value will only become apparent upon selfing and selection in the M_2 and subsequent generations.

The delay in flowering under stronger mutagenesis is a common phenomenon, often attributed to physiological stress or to disruption of genes controlling flowering time [48-

51].[49] did not directly measure flowering time, but their M_1 mutants showed overall physiological delays, and other researchers have explicitly noted flowering extensions. For example, [52] working with mung bean reported that higher EMS levels prolonged the time to flowering, likely due to mutational interference with developmental pathways. It is also recorded by [53,54], [55] in black gram (*Vigna radiata*). In cowpea, [56] observed an earlier onset of flowering and a decrease in days to 50% flowering in an EMS-treated yardlong bean (*Vigna sesquipedalis*) population, accompanied by reduced maturity duration. Such wide variation in flowering time due to EMS has also been reported by [15], who found that their M_2 cowpea population showed continuous distribution for days to flowering far beyond the range of the wild type. This implies that EMS created both later- and earlier-flowering individuals. Notably, [15] identified dozens of mutants that flowered significantly ($P<0.05$) earlier than the parent variety, indicating the emergence of new earliness traits. These results suggest that specific mutations can accelerate flowering, potentially by knocking out repressors of flowering even as overall vigor is reduced. In our mutants, while the predominant trend at high EMS dosages was delayed flowering and maturity, a few early-blooming mutants were observed at lower EMS treatments, indicating increased phenological variation. Our results confirm that EMS mutagenesis increased the variance in flowering time. While early flowering is advantageous for drought avoidance and multi-cropping systems, our evaluation of the EMS population did not yield any early-flowering mutants; instead, such occurrences were observed in the control group. This outcome aligns with results previously reported in mung bean and other pulse crops.

Effect of EMS on Days to First Harvest.The present study showed EMS treatment at all three concentrations produced a highly significant delay in days to first harvest (*dfh*) compared to the untreated control. No significant differences were detected among the three EMS concentrations themselves, again pointing to a threshold response wherein even the

lowest concentration was sufficient to substantially delay the onset of first harvest, with no additive effect at higher doses. The delays in days to first harvest observed in the present study are a logical downstream consequence of the earlier-documented delays in days to first flower and days to 50% flowering and are consistent with the general expectation that any disruption of floral transition will propagate through the reproductive developmental timeline. In cowpea, pod development and seed fill are closely coupled to the timing of anthesis, and a delay of in flowering onset would be expected to produce a delay of similar harvestable pod maturation as observed here. [25], applied EMS at 10 mM for 6 hours to cowpea varieties *IT84E-124* and *Vita 7*, reported variants in maturity date among the spectrum of mutations recovered in the M_2 generation, demonstrating that EMS can disturb the genetic determinants of cowpea maturation timing even at concentrations below those used in the present study.

In this study, the mutation frequencies recorded as 18.75%, 9.10%, and 12.50% for 20, 40, and 80 mM respectively, do not follow a simple linear dose-response pattern, with the lowest EMS concentration producing the highest proportion of phenotypic variants for this trait. This is consistent with the broader mutagenesis literature indicating that higher doses of chemical mutagens tend to produce proportionally more lethal or cryptic mutations rather than phenotypically viable and selectable variants. The dose of EMS concentration and exposure duration both affect the viability of induced mutations, and the frequency of viable mutations declined at concentrations that approach or exceed the LD_{50}, as higher doses shift the mutation spectrum increasingly toward deleterious rather than functional alleles [57]. A similar study by [58] who evaluated EMS effects on quantitative traits in garden pea (*Pisum sativum* L.) in the M_1 generation found that variation due to EMS treatment was significant for days to maturity, with increasing EMS concentration generally associated with delayed maturity, and noted that such delays in maturity were consistent across concentrations in a manner similar to flowering time responses. The strong similarity between *dfh* results across 20–80 mM in the present

cowpea study, and the absence of a further dose-dependent increment beyond the initial shift from the control, mirrors this threshold pattern observed in garden pea. The relatively higher mutation frequency at 20 mM for days to first harvest therefore suggests that sub-lethal doses may be more productive for generating harvestable maturity variants in cowpea, though this interpretation must be qualified by the relatively small population sizes in this M_1 study.

Effects of EMS on Days to 50% Mature Pods.Coefficients of variation were low and comparable across all treatments (9.1–10.5%), indicating that EMS did not increase population-level variance in pod maturation timing, but instead produced a coherent directional shift in mean maturity across the mutagenized populations. Mutation frequencies of 39.58%, 25.45%, and 23.21% for 20, 40, and 80 mM respectively again followed the declining trend with increasing EMS dose observed for *dfh*, reinforcing the interpretation that the 20 mM dose generated the highest proportion of viable phenotypic variants for maturation-related traits. The delay in *d50mp* closely parallelled the delay in *dfh* across all treatments, with the absolute number of days between first harvest and 50% pod maturity remaining stable across the control (approximately 30 days) and EMS treatments (approximately 28–30 days). This similarity suggests that EMS disrupted the upstream developmental program governing the onset of reproductive maturation, most plausibly through mutations affecting the floral transition pathway, rather than the rate of pod filling or seed development intrinsically. A study on EMS mutagenesis of fodder cowpea (var. *Aiswarya*) by [59] reported that days to maturity was among the quantitative traits showing significant variation in the mutated population in M_2, with EMS-induced mutants covering a broad range of maturity classes including both early and late maturing individuals relative to the control[44] .The observation of delayed maturity in the present M_1 study without evidence of any early-maturing variants at the population mean level is consistent with the expectation that dominant or co-dominant mutations, which are the only classes detectable at the phenotypic level in M_1, are more likely to disrupt rather than accelerate

developmental programs. Stable phenotypic variation in both directions, including earlier maturity, is expected to be more fully expressed from M_2 onwards, as the first non-chimeric generation in which homozygous recessive mutations can be phenotypically detected [13,60].

The agronomic implications of delayed maturity in EMS-mutagenized cowpea M_1 populations deserve consideration. While late maturity is undesirable from a production standpoint in environments with short growing seasons, it may represent a useful source of variation for adaptation to longer-season cropping systems or for intercropping arrangements where extended vegetative and reproductive periods are advantageous. The importance of early maturing mutations for short-season environments has long been recognized in cowpea improvement programs, with early maturing selections serving as candidates for areas with limited rainfall and as relay crops in rice paddies. In contrast, the late-maturing phenotype observed in the current M_1 population may harbor individuals carrying alleles that, in heterozygous combination, mask early maturing recessive mutations, suggesting that early maturity mutants with direct breeding utility may emerge from the M_2 onwards upon selfing and screening [61].

The results for *dfh* and *d50mp* demonstrated that EMS mutagenesis at concentrations of 20–80 mM induces a coherent and significant delay of 9–11 days in both harvest maturity and pod maturation in cowpea under M_1 conditions. This delay is may be attributed to the dominant expression of EMS-induced mutations disrupting the genetic flow of floral transition and reproductive development, consistent with findings in cowpea and other legumes. The absence of dose-dependent differentiation among EMS treatments suggested a threshold effect for maturity traits, while declining mutation frequencies at higher doses indicate that 20 mM may represent the most productive concentration for generating viable phenotypic variants in maturity-related traits. Evaluation of M_2 and subsequent generations will be essential to

characterize the full spectrum of maturity variation induced, including the recovery of potentially valuable early-maturing mutant classes.

Effects of EMS Mutagenesis on Yield-related and Yield traits

Non-Significant Traits: Number of Pods per Peduncle, Number of Pods per Plant at Maturity, Number of Seeds per Pod, Number of Seeds per Plant, Pod Width, and Percent Seed Abortion. EMS treatment at all three concentrations (20, 40, and 80 mM) produced no statistically significant differences from the control in number of pods per peduncle (*npp*), number of pods per plant at maturity (*nptm*), number of seeds per pod (*nsp*), number of seeds per plant (*nspt*), pod width (*pdw*), or percent seed abortion (*psa*). The lack of significant and notable mutants in the M_1 generation is likely due to these traits being highly polygenic and developmentally buffered, which restricts observable changes [4,62-64]. These findings suggest that a single generation of EMS exposure at these doses were insufficient to consistently disrupt the genetic architecture underlying these yield components in the M_1 generation. However, significant mutants were identified for these traits in M_2 populations, where recessive mutations are revealed, and chimerism is resolved [15,65-Click or tap here to enter text.67]. Moreover, higher EMS doses often reduce pod and seed numbers due to partial sterility or lower pollen viability, as seen in legumes [4,34,53].

[13] reported wide distribution of yield-related traits including number of pods per plant, number of seeds per pod, and number of seeds per plant primarily in the M_2 population rather than M_1, noting that the frequency of viable mutations depends strongly on treatment conditions and that functional mutations induced by EMS through point mutation have a greater chance of being dominant or co-dominant with only those classes easily detected by phenotypic observation in the M_1 generation[13]. The absence of significant mean shifts in these traits in the current M_1 study therefore may reflects the recessive nature of many yield-component mutations rather than an absence of induced mutations.

[31] reported highly significant mutants with improved performance for number of pods per plant, number of seeds per pod, number of locules per pod, and pod length were identified in EMS-mutagenized cowpea populations, but their detection required evaluation of the M_2 generation, reinforcing the need to advance mutagenized material to subsequent generations for meaningful yield screening. Similarly, [25] reported increases in number of seeds per pod, peduncles per plant, and 100-seed weight in advanced mutant lines of cowpea varieties ***IT84E-124*** and ***Vita 7*** following mutagenesis with EMS at 10 mM and NaN_3, though these improvements were characterized from M_2 onwards rather than M_1 plants [59] . The high coefficients of variation recorded in this study for *nsp*, *nptm*, *nspt*, and *psa* across all treatments, including the control, indicated that these traits are inherently variable and may require larger sample sizes or later generation evaluation to detect treatment effects. The notably high CV for *psa* reflected the expected wide individual variation in seed set across a mutagenized population. The higher performance in other yield traits exhibited by mutants may be attributed to pleiotropy, whereby mutation in one trait affects others [61], which further complicates detection of significant mean shifts in individual yield components when evaluation is confined to M_1.

Effects of EMS on Pod Length.Pod length (*pdl*) showed a significant overall treatment effect, with a dose-dependent reduction across EMS concentrations. Pairwise comparison confirmed a significant difference only between the control and the 80 mM treatment, this suggest that the reduction in pod length became statistically detectable only at the highest EMS concentration. The highest mutation frequency for *pdl* of 21.43% was observed in 80-mM treatment, which indicated that the 80 mM dose induced the greatest proportion of detectable variants, consistent with a dose-response relationship in this trait. The reduction in pod length with increasing EMS concentration is consistent with findings in the broader mutagenesis literature for legumes. Studies on fodder cowpea in the M_1 generation reported reductions in

several quantitative characters including pod length with increasing EMS concentrations, with reductions in quantitative traits observed to be colinear with increased mutagen dose irrespective of the mutagen used [59]. This pattern has been attributed to the mutagenic disruption of genes governing cell elongation and pod development [26]. In legumes, the activity of the inflorescence meristem determines the number and size of flowers and pods at each node through the growth of the floral meristem, and mutations affecting this developmental pathway can alter pod size and architecture [26]. EMS-induced point mutations at loci controlling pod wall cell division or expansion would therefore be expected to reduce pod length in a dose-dependent manner [68], as observed here.

In contrast, some advanced-generation mutagenesis studies have reported enhanced pod length in selected mutant lines. [69],obtained aphid-resistant cowpea mutants from gamma-irradiated populations with increased pod length among their improved attributes, while the same study noted that lines with significant increases in yield parameters were selected from M_2 screening [25]. The apparent contradiction between M_1 reductions and M_2 improvements in pod length likely reflects the phenomenon of heterozygous M_1 plants masking recessive gain-of-function alleles, which only become homozygously expressed and selectable in subsequent generations [70]

Effects of EMS on Number of Locules per Pod.Number of locules per pod (*ln*) showed the most pronounced and dose-dependent response to EMS treatment among all traits evaluated. All pairwise comparisons among treatments were statistically significant, and the mutation frequency increased with concentration, which reached 50% at 80 mM. The CV also increased with EMS dose, which reflected the expanding phenotypic diversity generated at higher concentrations. The strong dose-dependent reduction in locule number is particularly notable from a breeding standpoint, as locule number is a primary determinant of the number of seeds per pod and therefore of pod yield potential. In legumes, the identity and activity of

the inflorescence meristem governs flower and pod architecture, and mutations in genes regulating meristematic activity can directly alter the number of seeds per pod and overall pod structure[68].

The significant reduction in *ln* at 40 and 80 mM, without a corresponding significant reduction in *nsp*, suggests that while EMS disrupted the developmental program responsible for locule number, the individual seed development process within remaining locules were largely maintained. This dissociation between locule number and seeds per pod may indicate that some locule positions failed to initiate rather than that initiated locules failed to complete seed set. [31] noted that highly significant mutants with improved performance for number of locules per pod were identified in EMS-mutagenized cowpea in M_2 populations, demonstrating that both directional increases and decreases in locule number are achievable through EMS mutagenesis depending on the specific mutation event. The predominance of locule-reducing variants in the current M_1 population is consistent with the expectation that dominant loss-of-function mutations more likely to be expressed in heterozygous M_1 plants tend to disrupt rather than enhance developmental programs. Alleles conferring additional locules, which would likely require gain-of-function or additive effects, may be present in the population but will only become phenotypically apparent upon homozygosity in M_2.

Effects of EMS on Seed Dimensions — Length, Width, and Thickness.The three seed dimension traits; seed length (*sdl*), seed width (*sdw*) and seed thickness (*sdt*) collectively exhibited highly significant responses to EMS treatment, but with notably different dose-response patterns across the three concentrations. ***Seed length*** increased with EMS treatment, as all comparisons between the control and treated groups were highly significant. However, there was no significant difference between the 40- and 80-mM treatments, indicating a plateau above 40 mM. The coefficient of variation decreased with increasing EMS dose, showing reduced variability. This suggested that EMS caused a consistent shift toward longer seeds

rather than increasing variation. This pattern was consistent with the expression of mutations that promoted seed elongation, leading to a uniform directional effect across the population.

Seed width followed a consistent but shallower pattern of EMS-induced increase. All three EMS concentrations were significantly wider than the control, though none differed significantly from one another indicating again a threshold effect where even the lowest dose captured the full directional shift achievable for this trait. The absence of dose-dependent differentiation among EMS treatments for seed width, combined with the significant and consistent departure from the control, points to a single effective threshold below 20 mM at which EMS disrupted or altered the genetic regulation of seed width, with no further phenotypic increment at higher concentrations.

Seed thickness, by contrast, produced the most complex and non-linear response of the three dimensions. The 20 mM treatment was the only concentration to produce a significant increase over the control, while both the 40 mM and 80 mM treatments were statistically indistinguishable from the control but significantly lower than the 20 mM treatment. The mutation frequencies for *sdt* , 12.50%, 1.82%, and 1.79% at 20, 40, and 80 mM respectively showed by far the steepest drop between the lowest and intermediate doses of any trait in this study, suggesting that the genetic targets for seed thickness are highly sensitive to EMS dose and that concentrations above 20 mM largely neutralized or reversed the thickness-enhancing mutations, possibly by simultaneously inducing compensatory or antagonistic mutations in related seed developmental pathways.

The divergence in response among the three seed dimensions is agronomically and biologically meaningful. Seed length responded with a strong, progressive, dose-dependent increase that plateaued above 40 mM; seed width responded with a uniform, threshold-type increase independent of dose; and seed thickness showed a transient, concentration-restricted increase that was not maintained above 20 mM. This dissociation among the three axes of seed

growth implies that seed length, width, and thickness in cowpea are at least partially under distinct genetic control, and that EMS mutagenesis differentially targeted these control systems across the tested concentration range. Consistent with this interpretation, QTL mapping studies in cowpea have identified distinct genomic regions controlling pod and seed dimensions, including pod length and 100-seed weight, suggesting that seed size traits can be genetically decoupled and are governed by a combination of shared and independent loci [71]. The increases in seed length and width documented here are consistent with findings from induced mutagenesis studies in cowpea and related legumes. [25] reported significant increases in seed weight among EMS-mutagenized cowpea lines, with traits including plant height, seed weight, and protein content increasing in mutant populations compared to untreated controls. The outcomes were attributed to EMS altering structural gene loci governing these quantitative traits[25]. [72] similarly documented substantial increases in 100-seed weight in gamma-irradiated cowpea mutants, with 100-seed weight rising from *16.95 g* in the control to *22.80 g* in selected M_2 mutant lines, and observed that seed size traits displayed high heritability, indicating a strong genetic basis amenable to selection.

The pattern across all three seed dimensions, thus progressive elongation in length, uniform widening, and transient thickening points toward a net increase in overall seed volume in EMS-treated populations, predominantly driven by enhanced length and, to a lesser degree, width. This three-dimensional enlargement is likely to translate into increased individual seed weight, which is consistent with the significant increases in seed weight (*swg*) documented in this study. [13]attributed high performance in multiple yield traits exhibited by EMS-mutagenized cowpea mutants partly to pleiotropy, whereby mutation in one gene affects several related developmental traits. This mechanism may explain the coordinated, if not perfectly similar, increases across seed length, width, weight, and, at 20 mM, thickness observed in the current M_1 population. As with all M_1 data, the observed means represented

population-level responses from heterozygous individuals and should be interpreted cautiously as indicators of induced genetic variation rather than fixed trait values. Stable phenotypic expression of EMS-induced seed dimension changes is expected from M_2 onwards, as the first non-chimeric generation in which homozygous alleles become expressed and individually selectable [13]. Selection in M_2 for individuals combining large seed length, adequate width, and maintained thickness particularly among progeny of 20 and 40 mM-treated plants where mutation frequencies for seed dimension traits were highest, offers the most promising route to isolating fixed large-seeded mutant lines of breeding value.

Effects of EMS on Seed Weight. Seed weight (*swg*) showed the most pronounced and consistently positive response to EMS treatment among all yield traits evaluated. All three EMS concentrations produced significantly heavier seeds than the control. Pairwise comparisons confirmed that 20 and 40 mM did not differ significantly from each other, but both were significantly heavier than 80 mM, which in turn remained significantly heavier than the control. This pattern describes a partial dose-dependent attenuation: maximum seed weight enhancement occurred at 20–40 mM, with a decline though still significant improvement over the control at 80 mM. Mutation frequencies of 54.17%, 49.10%, and 16.07% at 20, 40, and 80 mM respectively confirm the well-documented inverse relationship between EMS dose and the proportion of viable, selectable phenotypic variants, as higher concentrations shift the mutation spectrum increasingly toward lethal or cryptic alleles rather than productive, phenotypically expressed changes[13]. The significant increase in seed weight across all EMS treatments is consistent with findings in cowpea and related grain legumes subjected to chemical and physical mutagenesis. [25], applying EMS at 10 mM for 6 hours to cowpea varieties ***IT84E-124*** and ***Vita 7***, reported significant increases in seed weight and other yield parameters among selected mutant lines, attributing improvements in polygenic characters like yield to changes in simply inherited traits or mutations at structural loci. [72] similarly reported significant

increments in 100-seed weight in gamma-irradiated cowpea mutants at M_2 and M_3 generations, observing increases from *16.95 g* in the control to *22.80 g* in selected mutant lines, a proportional gain broadly comparable to that recorded in the 20- and 40-mM treatments of the current study.

The comparable increase in seed weight with the previously documented increases in seed length and seed width supports an interpretation of coordinated enhancement of seed size across multiple dimensions at low-to-intermediate EMS concentrations. [59], who used EMS to develop improved fodder cowpea populations, reported significant variation in seed yield per plant in M_2 populations, noting that mutations altering seed morphology traits tend to produce coordinated changes in associated yield components, consistent with pleiotropic gene action or tight genetic linkage among seed size and weight loci. The reduction in seed weight at 80 mM, while still above the control, is likely attributable to higher mutagenic load at this concentration interfering with genes governing seed filling efficiency, endosperm development, or photosynthate partitioning effects that would be expected to partially offset the seed-enlarging mutations still being expressed from this dose.

Effects of EMS on Yield Per Plant. Yield per plant (*yld*) showed a significant overall treatment effect, but with a more limited and dose-restricted pattern than seed weight. Only the 20 mM treatment produced a statistically significant increase in yield relative to the control (*61.19 ± 3.34 g* vs. *48.79 ± 3.01 g*), representing an approximate 25% yield advantage. The 40 mM (*58.02 ± 2.59 g*) and 80 mM (*51.16 ± 2.87 g*) treatments produced numerically higher yields than the control but were not significantly different from it, nor from the 20 mM treatment. The high coefficients of variation across all groups (33.1–42.0%) reflect the inherently variable nature of yield per plant in M_1 populations, a consequence of the heterogeneous mutational load across individuals and indicate that a portion of the variance attributable to EMS effects may have been obscured by this within-treatment variability.

Mutation frequencies of 16.67%, 12.72%, and 10.71% for 20, 40, and 80 mM respectively were low and declined modestly with increasing EMS concentration, consistent with the modest treatment effect sizes recorded for this trait.

The significant yield increase at 20 mM is agronomically important, even within the context of M_1 limitations. [59] documented significant variation in seed yield per plant in EMS-mutagenized cowpea M_2 populations and concluded that EMS mutagenesis is effective in inducing yield-relevant genetic changes, and that seed yield and days to maturity were among the quantitative traits showing significant variation in the mutated population. The pattern observed in the current study which showed significant yield improvement at the lowest EMS concentration but non-significant trends at higher doses is consistent with the principle that sub-lethal mutagen doses are most productive for generating viable, yield-enhancing alleles. [13] attributed high yield performance in EMS-mutagenized cowpea mutants to EMS altering genes responsible for this trait, and additionally to pleiotropy, whereby mutation in one trait affects others a mechanism that is particularly relevant here, as the yield increases at 20 mM likely reflect the combined downstream effects of increased seed weight, enhanced seed dimensions, and maintained seed number documented in the same treatment group.

The overall yield results, viewed alongside the seed weight and seed dimension data, present an internally consistent picture: 20 mM EMS induced the broadest suite of positive seed quality and yield mutations in this M_1 population, while 40 and 80 mM produced proportionally more neutral or disruptive mutations that dampened the yield response. As with all M_1 observations, however, these means represent population averages across heterozygous individuals and cannot directly predict the yield potential of specific mutant lines. The identification and selection of superior individual mutant plants rather than evaluation of population means will be the critical next step, as demonstrated by, [72] who identified twelve putative cowpea mutants combining early maturity with significantly higher seed yields than

the parental control through individual plant selection from M_2 and M_3 generations. Advancing the current population to M_2 and screening individual lines for the convergence of high seed weight, appropriate seed dimensions, and elevated yield per plant represents the most productive strategy for exploiting the genetic variation induced by EMS in this study.

In our research, high-yielding mutants, such as B09, typically produce larger seeds with higher seed weight, a favorable combination for yield improvement. Similarly, [25] and [72] reported cowpea mutants with enlarged seeds and improved yield performance, consistent with the superior lines identified in the present study. Yield stimulation following mutagenic seed treatments has also been documented in other legumes. Increased yield or yield components after mutagen exposure have been reported in pigeonpea [73], mung bean [74], and urdbean treated with gamma rays, EMS, and sodium azide [75]. These studies collectively support the notion that induced mutagenesis can unlock favorable allelic variation controlling yield traits.

In contrast, many mutants, particularly from the highest EMS dose (80 mM), exhibited reductions in yield and its components, including pod length, number of locules, seed size, and seed weight. Such adverse effects of high mutagen doses are well documented. [76] In okra and [77] in petunia, it was reported that mutagenesis frequently produces lines with reduced reproductive output. In pigeon pea (*Cajanus cajan*), [78] observed that EMS doses beyond the optimal range led to sharp declines in pollen fertility and seed yield. At the same time, [79] reported positive yield shifts at lower mutagen doses and negative shifts at higher doses. Our results closely mirror this dose-dependent pattern. Yield depression at elevated doses of irradiation or chemical mutagens has been widely reported. Similar declines were observed by [52] in *Gloriosa superba*, [53] in *Vigna radiata* under EMS and DES treatments, [80] in *Vigna mungo*, and [81] in *Vigna radiata*. These findings reflect the accumulation of deleterious mutations at higher mutagen doses, which can impair physiological and reproductive processes. Conversely, [4] reported stimulation of seed weight in pigeon pea under combined mutagenic

treatments, suggesting that moderate doses may enhance specific yield components. Similar observations were reported by [82], [83], and [26] across various pulse crops, reinforcing the dose- and trait-dependent nature of mutagenic responses.

One elite mutant (B33) from the 20 mM EMS treatment in the current study produced approximately 25% higher seed yield than the control, accompanied by a modest increase in 100-seed weight. This indicates that EMS successfully generated yield-enhancing alleles. Similarly, [59] reported EMS-derived cowpea lines with significantly increased pod numbers confirm the potential of chemical mutagenesis to improve yield. It is also noteworthy that the frequency of desirable mutants was low, consistent with previous reports. [15] estimated that only 0.1–1% of mutants exhibited significantly improved yield-related and yield traits. Similarly, although a few promising mutants were identified in the present study, the majority performed at or below the level of the control. This underscores the need to screen large mutant populations to capture rare beneficial events.

Multivariate Analysis: PCA Biplot and Cluster Grouping

PCA Biplot of phenological, yield-related and yield traits

Variance structure and principal component interpretation. Principal component analysis was applied to the full set of 18 phenological and yield-related traits across 190 M_1 cowpea genotypes to characterize the multivariate structure of EMS-induced variation. The first eight principal components collectively explained 76.2% of the total phenotypic variance, with PC1 and PC2 accounting for 17.7% and 15.1% respectively a combined 32.8% of variance captured in two dimensions. The relatively distributed variance structure across eight components reflects the complexity of the trait matrix, the phenotypically heterogeneous nature of M_1 mutagenized populations, and the partial independence of phenological, reproductive, and seed morphology trait clusters. [85], evaluated cowpea breeding lines using multivariate analysis, similarly found that the first three principal components explained 76% of total

variation and noted that groupings in biplots conformed well with dendrogram results, demonstrating that PCA effectively captures the major sources of agronomic differentiation in cowpea populations [7]. PC1 carried strong positive loadings from seed length (*sdl* = 0.73), seed width (*sdw* = 0.70), days to first harvest (*dfh* = 0.68), days to first flower (*dff* = 0.61), days to 50% mature pods (*d50mp* = 0.59), days to 50% flowering (d50f = 0.57), and seed weight (*swg* = 0.53). This axis therefore captures a composite gradient of phenological delay and seed dimensional enlargement, two trait groups that co-vary positively across EMS treatments in this present study, this reflects the tendency of mutagenized lines to simultaneously delay reproductive development and produce morphologically larger seeds. PC2 was dominated by highly positive loadings from number of seeds per plant (*nspt* = 0.96), yield per plant (*yld* = 0.95), and number of seeds per pod (*nsp* = 0.75), alongside negative loadings from number of pods per plant at maturity (nptm = −0.66), days to first flower (*dff* = −0.64), and days to 50% flowering (*d50f* = −0.68). PC2 thus represented a yield productivity axis, where high positive scores reflect superior reproductive output in terms of seed number and total yield, decoupled from phenological timing. Together, PC1 and PC2 defined a two-dimensional space within which the fundamental EMS-induced trade-off between phenological disruption, seed enlargement, and reproductive yield can be visualized at the individual genotype level.

Treatment group separation in the biplot.The biplot distribution of genotype scores revealed a clear dose-dependent spatial separation among EMS treatment groups. Control genotypes (A01–A31, 0 mM) formed a compact cluster on the extreme negative side of PC1, consistent with their early phenology, uniform developmental timing, and moderate to high yield performance relative to the mutagenized population. This clustering confirmed the low phenotypic variance expected of untreated plants within a uniform cultivar background and validates the internal coherence of the control group. [86], applied PCA to M_5 Tswana cowpea mutant lines and similarly found that the control variety formed a separate cluster that diverged

from all mutant lines in multivariate space, confirming that induced mutagenesis effectively altered the genetic makeup of the treated population in ways detectable by multi-trait analysis.

Genotypes from the 20 mM treatment (B01–B48) showed partial overlap with the control group but were moderately displaced toward the positive PC1 region and exhibited greater within-group dispersion, particularly along PC2. This intermediate positioning reflects the milder and more heterogeneous mutational load associated with the lowest EMS concentration were consistent with individual-level variation in mutation induction and expression at sub-lethal doses. Genotypes from the 40 mM (C01–C55) and 80 mM (D01–D56) treatments were progressively displaced toward the positive PC1 axis and displayed substantially greater scatter across both PC1 and PC2, indicating dose-dependent increases in phenological delay alongside widened variation in reproductive output. Multivariate PCA of cowpea germplasm has consistently shown that high phenotypic diversity, reflected by wider scatter in biplot space, creates greater opportunity for selection of superior genotypes, as variation among traits provides the basis for discriminating among individuals for multiple desirable characteristics simultaneously [87].

Trait vector interpretation and EMS-induced trade-offs.The present study showed the orientation of trait vectors in the biplot provided insight into the relationships among traits and the nature of EMS-induced phenotypic trade-offs. Vectors for the four phenological traits (*dff*, *d50f*, *dfh*, *d50mp*) were oriented horizontally towards the positive PC1 axis, this confirmed their strong association with the right-side positioning of high-dose EMS genotypes. The *sdl* and *sdw* vectors were directed toward the lower-right quadrant, positive PC1 and negative PC2 which indicated that seed dimensional enlargement was associated with phenological delay but inversely related to yield productivity. In contrast, the *yld*, *nspt*, and *nsp* vectors pointed strongly toward the upper region of the biplot (positive PC2), which formed an approximately perpendicular angle to the *sdl*/*sdw* vectors. This near-orthogonal relationship between yield

and seed size vectors quantitatively confirms the resource allocation trade-off implied by the individual trait analyses: EMS-induced genotypes that developed larger seeds tended to produce fewer seeds per pod and per plant, resulting in reduced total yield. PCA-driven multivariate analyses in other legume systems have similarly identified trade-offs between plant architectural and yield productivity traits loading on different principal components, where biplots revealed that selection based on single traits may inadvertently compromise performance in correlated traits and reinforced the need for multi-trait selection indices[88].

The *swg* vector was oriented toward the positive PC1 and moderately positive PC2 region which indicated that heavier seeds were associated with both phenological delay and, to a degree, maintained or enhanced yield performance, a positioning distinct from *sdl* and *sdw*, which loaded more strongly on the negative PC2 side. This suggested that seed weight integration partly captured the influence of both seed size and seed number components, reflecting its composite nature as a product of seed dimensions and density.

The *pdl* and *ln* vectors were directed upward left, with *pdl* loading strongly on PC2 and *ln* on both PC1 and PC2. These vectors were oriented broadly toward the central and upper biplot region, indicating that genotypes with longer pods and higher locule numbers were not confined to a single EMS treatment group and could be found across the treatment spectrum consistent with the individual trait analyses which showed that pod length was significantly reduced only at 80 mM while locule number showed a progressive dose-dependent reduction.

Identification of putative mutant classes. The biplot facilitated the graphical identification of three distinct classes of agronomically relevant putative mutants, each positioned in a different region of the two-dimensional space. High-yielding putative mutants were identified from genotypes clustered in the upper region of the biplot with positive PC2 scores, in close alignment with the *yld*, *nsp*, *nspt*, and *swg* vectors. These included B33, B28, B22, B31, D56, D45, D25, C5, C41, B26, and B21. The presence of genotypes from both 20

mM (B-series) and 40–80 mM (C and D series) treatments among this group indicated that yield-enhancing mutations were not exclusively associated with the lowest EMS dose, though most B-series genotypes in this cluster is consistent with the higher mutation frequencies and significant mean yield increase documented at 20 mM in the individual trait analyses. [86] similarly identified mutant lines with improved grain yield through PCA biplot analysis and noted that the distribution of mutagenized populations in biplot space, this reflected the interaction of genotype and mutagen dose that generated significant quantitative trait variation that could be exploited through targeted selection [86].

Large-seed putative mutants were identified from genotypes located along the negative PC2 axis in the direction of the *sdl* and *sdw* vectors, including D23, D7, D34, D47, C37, C39, C40, C42, B18, B11, and B8. These genotypes, from the 40- and 80-mM treatments, produced larger seeds but fewer in number, which resulted in lower overall yield. This pattern suggested that resources were redirected to produce fewer but larger seeds. Although this was not ideal for total yield, these genotypes were useful for breeding large-seeded varieties preferred by consumers and for studying the genetic basis of seed development in cowpea.

Early-phenology putative mutants were identified from the extreme negative side of PC1, opposite to the late-flowering and maturity vectors, and included B19 and C2 notably from the 20- and 40-mM treatments respectively. These genotypes showed accelerated phenological development relative to the control group, suggesting the presence of rare dominant or semi-dominant early-flowering mutations within the mutagenized population. Their identification is agronomically significant: previous studies in cowpea mutagenesis have confirmed that EMS causes mutations in genes responsible for maturity-related traits, and early maturing mutants are particularly valuable for short-season production systems and as relay crops in rice paddies where a compressed growing season is critical[13].

Genotypes exhibiting extreme positive PC1 scores with weak alignment to yield vectors including B9, B7, C46, and D20 were classified as severely delayed and low performing. These lines, positioned in the far-right region of the biplot with little positive PC2 displacement, represented the most mutagenized damaged individuals in the population, they likely carried multiple loss-of-function mutations with pleiotropic negative effects on reproductive development and yield. While unsuitable for direct advancement, they were retained as extreme phenotypes for genetic and physiological characterization, and as potential donors in targeted crosses where specific mutant alleles may segregate independently from the broader phenotypic impairment.

Broader utility of PCA in EMS mutation studies.In this study we reduced the dimensionality of the 18-trait dataset into two interpretable axes, the biplot enabled simultaneous visualization of dose-treatment group separation, inter-trait relationships, and individual genotype positioning relative to trait vectors all within a single analytical framework. This multi-trait perspective is particularly important in M_1 studies where high within-treatment variance, heterozygosity, and chimerism create complex phenotypic distributions that are difficult to interpret through single-trait analyses alone. PCA biplot analysis of cowpea populations has been shown to effectively identify which traits account for the most variation among genotypes, allowing breeders to focus selection on the most discriminated trait combinations and to identify individuals that deviate meaningfully from the population center in the direction of target trait improvement. [89]. Also, [90], used multi-trait analysis to identify superior M_2 individuals and reported that mutants with extremely high values in key traits, such as seeds per plant, could be distinguished from the population distribution. In the present study, the biplot confirmed that yield-related traits (*npp*, *nsp* and *swg*) and seed dimension traits loaded most strongly on the first two principal components, this provided the clearest discrimination among EMS-treated lines, while traits with minimal biplot

contribution such as *psa* and *pdw* had limited power to differentiate among genotypes at the multivariate level.

The clear spatial separation between high- and low-yielding mutants, and between early- and late-phenology groups, demonstrated that the genetic variation induced by EMS at 20–80 mM was sufficient to generate agronomically meaningful divergence, detectable at the population level in the M_1 generation. Confirmation of the most promising putative mutants particularly the high-yielding group (B33, B28, B22, B31, D56, D45, D25, C5, C41, B26, B21) and the early-phenology group (B19, C2) through M_2 progeny testing and subsequent generation advancement remains essential before any breeding value can be assigned.

Hierarchical Cluster Analysis of EMS-Mutagenized Cowpea

Hierarchical cluster analysis using Ward's linkage method effectively partitioned the 190 M_1 cowpea genotypes into six phenotypically distinct clusters, each characterized by a unique combination of phenological, yield-related and yield trait profiles. The resolution of six clusters from a single mutagenized population is consistent with reports from other induced mutagenesis studies in cowpea and related legumes, which have commonly observed between three and six discrete phenotypic groupings following mutagen treatment, reflecting the breadth of the genetic variation induced [86,91]. The clustering outcome in the present study demonstrated that EMS mutagenesis at 20–80 mM generated sufficient phenotypic divergence across 18 traits to resolve multiple distinct genotypic classes within a single cultivar background, a result that underscores the utility of Ward's method for structuring genotype selection in M_1 mutation populations.

Cluster 1: high-yielding EMS-derived genotypes. Cluster 1, characterized by prolonged phenological development and superior yield performance, contained disproportionately high proportions of the 20- and 40-mM treatment groups, with 35.42% and 49.10% of these populations respectively assigned to this cluster. Cluster 1 contained the

highest proportion of agronomically desirable genotypes, and most putative mutants selected for advancement. The combination of elevated seed weight (up to 28.68 g in D56), extended phenological development, and high yield per plant (up to 111.85 g in D56) in this cluster is consistent with reports of yield-enhancing mutations in EMS-mutagenized cowpea populations. [13] selected the top twenty mutants with high yield performance in terms of number of seeds per plant from their M_2 EMS-mutagenized cowpea population, they reported that traits including higher number of pods per plant, increased protein content and high seed weights had been obtained through EMS mutagenesis in earlier cowpea studies. The genotypes identified in Cluster 1 in the present study include D56, D45, D25, B31, B28, B22, C41, and C5 exhibited yield values significantly exceeding the control range and represented the primary candidates for advancement to M_2 and subsequent generation evaluation.

Cluster 2: control-dominated baseline cluster. Cluster 2, which represented the population's baseline phenotypic profile and was dominated by control genotypes (61.29% of the 0 mM population), received minimal representation from EMS-treated lines. The concentration of control genotypes in Cluster 2 confirmed that the untreated population occupied a phenotypically distinct region of the multivariate trait space, separated from the majority of EMS-treated genotypes. The small number of EMS-treated lines assigned to this cluster notably C02 and C14, both from the 40 mM treatment, with early days to first harvest values of 38 and 39 days respectively, represented a class of putative early-phenology mutants that maintained control-like performance across most traits while showing accelerated crop development. [91], who developed new cowpea mutant genotypes through gamma irradiation, also reported that cluster groupings could be attributed to the origin of genotypes and the degree of genetic alteration induced, with control varieties consistently forming separate clusters that diverged from mutagenized lines in multivariate space [92]. This confirming the effectiveness of mutagenesis in altering the genetic makeup of the treated population [92].

Cluster 3: severely impaired high-dose genotypes. In contrast, Cluster 3 was characterized by delayed phenology, reduced locule number, and low yield, and it was mainly composed of genotypes from the 80 mM treatment, with 28.57% of that group assigned to this cluster. This cluster showed the most severely impaired phenotypes in the population. Representative genotypes B7, B9, C46, and D20 showed marked negative deviations from the control range across multiple traits simultaneously and were classified as low-value mutants unsuitable for direct advancement. This cluster is analogous to the poor-performance groups consistently reported in high-dose mutagenesis studies. [93] noted that higher mutagen doses tend to produce populations with reduced fertility, delayed maturity, and inferior agronomic performance, and recommended against their use in breeding programmes where the objective is to recover viable, productive mutant lines[94]. While the genotypes in Cluster 3 were not selected for advancement, they were retained as phenotypic reference material, as the extreme mutations they carry may be informative for gene function studies or for identifying linkage between yield-impaired and yield-neutral alleles in subsequent molecular characterization.

Cluster 4: early-flowering high-yield mutants.Cluster 4 contained a distinctive combination of early days to first flower and days to 50% flowering with extended days to first harvest and elevated yield, a trait profile not represented in any other cluster. The presence of B19 (*yld* = 93.59 g, *dff* = 36 days) and B33 (*yld* = 125.44 g, *dff* = 37 days) in this cluster is particularly noteworthy: these genotypes achieved the highest individual yields in the entire population while retaining comparatively early flowering phenology, a combination of traits of direct relevance to short-season cowpea production. EMS-induced early maturing mutants have been specifically highlighted as valuable in cowpea breeding because of their ability to escape drought or tolerate insect damage due to their short reproductive phase, with early mutants with days to flowering of 38–41 days having been isolated from M_2 EMS-mutagenized cowpea populations in earlier studies [13]. The presence of such genotypes in Cluster 4 across

the 20- and 40-mM treatments, but not exclusively at the lowest dose, reinforces the value of evaluating mutant lines individually rather than making selection decisions based solely on treatment concentration.

Cluster 5: moderate performance with seed weight improvement. Cluster 5, composed exclusively of EMS-treated genotypes from all three treatment groups, was characterized by moderately delayed phenological traits, elevated seed weight, and reduced locule number, resulting in yield performance generally below the Cluster 1 and Cluster 4 groups but above the impaired lines of Cluster 3. The absence of control genotypes from this cluster confirms that the trait profile it represented, elevated seed weight combined with moderately delayed development and was not expressed within the untreated population and is therefore a product of mutagenesis. Genotypes in this cluster may still harbor breeding value as parents for crosses targeting seed quality improvement, even if their direct yield performance does not match the elite lines identified in Clusters 1 and 4.

Cluster 6: large-seeded, seed-weight-driven yield. Cluster 6 was the smallest and most phenotypically distinctive group in the analysis, contained only 9 genotypes from the 20- and 80-mM treatments. These genotypes combined large seed dimensions and high seed weight with moderate phenological delay and relatively low seed number per pod, producing elevated yield per plant primarily driven by individual seed weight rather than seed number. Selected genotypes from this cluster, including B07 (*swg* = 29.99 g, *yld* = 67.18 g) and D36 (*swg* = 28.29 g, *yld* = 74.69 g), represented a distinct agronomic type that may be particularly valuable for breeding programmes targeted at large-seeded, consumer-preferred market classes. [95], developed high-yielding cowpea mutant lines through gamma irradiation and sodium azide, similarly used dendrogram analysis to separate control and mutant plants into distinct clusters, confirmed that considerable genetic variability was generated and that genetically diverged

mutants could be employed directly as mutant varieties or as parents in crossbreeding programmes for advanced improvement and trait fixation[95].

Broader utility of cluster analysis in EMS mutation studies. This dose-stratified clustering pattern is consistent with the well-established principle that lower and intermediate mutagen doses preferentially generate viable, selectable phenotypic variants, while higher doses shift the mutation spectrum toward lethal or disruptive alleles. [93] confirmed this pattern in cowpea, they demonstrated that lower and intermediate mutagen doses induced higher frequencies of morphological mutations with the least biological damage and recommended these doses for future breeding programmes to obtain a high spectrum of desirable mutants[94]. The dominance of 40 mM-derived genotypes in Cluster 1 and of 80 mM-derived genotypes in Cluster 3 precisely mirrors this dose–performance gradient. However, EMS dose was not a perfect predictor of cluster assignment and individual genotype performance must be considered on its own merits. Some 20 mM genotypes were assigned to lower-performing clusters, while some 80 mM genotypes clustered with better-performing lines. [13] , in their EMS mutagenesis study of cowpea cv. *Asontem,* emphasized that the phenotypic expression of each mutant line is unique and determined by the specific gene(s) altered by the mutagen. [13] noted that the high yield performance of selected mutants may reflect EMS altering genes responsible for traits as well as pleiotropic effects whereby mutations in one trait affect related traits[13]. Results from the present study reinforced this interpretation: multivariate clustering based on actual trait performance provided a more reliable selection criterion than EMS dose alone.

The clustering outcomes confirmed that the elite putative mutants identified across Clusters 1, 4, and 6 shared a multi-trait profile that were not present in the control population, suggesting they carried functional alleles which favored yield, seed size, or early phenology that were induced by EMS treatment. Agronomic evaluation of cowpea mutant lines in

Eswatini demonstrated that cluster analysis is a key non-parametric statistical technique that facilitates the identification of diverse genotypes with contrasting traits for breeding and production, and that genotypes grouped in high-yielding sub-clusters can be directly recommended for production or use as crossing parents in further improvement programmes[92]. The present study reinforces this conclusion, with the elite clusters identified here providing a structured and evidence-based shortlist of M_1 genotypes for advancement to M_2 and evaluation in replicated trials across environments.

Conclusion

This study demonstrates the effectiveness of ethyl methane sulfonate (EMS) in inducing substantial genetic variability in cowpea (*Vigna unguiculata* (L.) Walp), thereby broadening the phenotypic spectrum for key agronomic, yield, and seed traits. Contrast analysis revealed significant treatment effects and highlighted superior mutant lines, while principal component analysis (PCA) identified yield and reproductive traits as the primary drivers of phenotypic variation. These findings were consistently supported by cluster analysis, which grouped mutants according to overall agronomic performance. The convergence of these analytical approaches strengthened the selection of putative mutants, including high-yielding (B33, B31, B28, B22, C41, C05, D25, D45, D56), early-phenology (B19), and large-seeded or prolific podding lines (D34, C39, C40).

As the study was confined to the M_1 generation, further evaluation in the M_2 and M_3 generations is required to confirm trait stability, heritability, and breeding value. Complementary molecular analyses will be essential to explain the genetic basis of the observed variation and support marker-assisted selection. Overall, the selected mutants constitute valuable genetic resources for cowpea improvement and have strong potential to enhance productivity and global food security. Considering the documented genetic variability of cowpea for nutritional traits such as protein and mineral content, the EMS-induced mutants

identified in this study may serve as valuable genetic resources for future breeding programs targeting both yield and nutritional quality improvement .

Acknowledgments

HMK, RANK, IKA, and FO conceived and designed the study. HMK and RANK performed the experiments. HMK analyzed the data and drafted the manuscript. RANK and IKA provided critical revisions. All authors read and approved the final manuscript. We gratefully acknowledge the Department of Plant and Environmental Biology at the University of Ghana, Legon, for supporting this research.

References

1. Sert H, Ceyhan E. The effects of seed yield and some agricultural characters of different plant density on cowpea (Vigna sinensis (L.) Savi) in Hatay ecological conditions. Selcuk Journal of Agriculture and Food Sciences. 2012;26(1):34–43.
2. Harmankaya M, Ceyhan E, Çelik AS, Sert H, Kahraman A, Özcan MM. Some chemical properties, mineral content and amino acid composition of cowpeas (Vigna sinensis (L.) Savi). Quality Assurance and Safety of Crops & Foods. 2016;8(1):111–6. doi:10.3920/QAS2014.0487
3. Boukar O, Massawe F, Muranaka S, Franco J, Maziya-Dixon B, Singh B. Evaluation of cowpea germplasm lines for protein and mineral concentrations in grains. Plant Genetic Resources. 2011;9:515–22.
4. Singh BD. Plant Breeding: Principles and Methods. 7th ed. New Delhi: Kalyani Publishers; 2006.
5. dos Santos J V, Valliyodan B, Joshi T, Khan SM, Liu Y, Wang J, et al. Evaluation of genetic variation among Brazilian soybean cultivars through genome resequencing. BMC Genomics. 2016;17(1):110. doi:10.1186/s12864-016-2431-x
6. Oladosu Y, Rafii MY, Abdullah N, Hussin G, Ramli A, Rahim HA, et al. Principle and application of plant mutagenesis in crop improvement: A review. Biotechnology & Biotechnological Equipment. 2016;30(1):1–16.
7. Owusu EY, Karikari B, Kusi F, Haruna M, Amoah RA, Attamah P, et al. Genetic variability, heritability, and correlation analysis of maturity and yield traits in Cowpea (Vigna unguiculata (L.) Walp.) in Northern Ghana. Heliyon. 2021;7(9).
8. Bhat TA, Khan AH, Parveen S. Comparative analysis of meiotic abnormalities induced by gamma rays, EMS, and MMS in Vicia faba L. Journal of Indian Botanical Society. 2005;84:45–58.
9. Olasupo FO, Ilori CO, Forster BP, Bado S. Mutagenic effects of gamma radiation on eight accessions of cowpea (Vigna unguiculata [L.] Walp.). Am J Plant Sci. 2016;7(2):339–51.
10. Jou-Nteufa C, Ceyhan E. Genetic analysis of seed yield and some traits in cowpea using diallel analysis. Turkish Journal of Agriculture and Forestry. 2024;48(3):278–93. doi:10.55730/1300-011X.3180

11. Dalgıç H, Ali A, Karadaş S, Korkmaz A, Ayran Çolak İ, Harmankaya M, et al. Diallel analysis of cowpea (Vigna unguiculata L. Walp) for protein and mineral composition: Genetic variability and heritability. Euphytica. 2025;221:168. doi:10.1007/s10681-025-03618-0
12. Hoque ME, Morshad MN. Somaclonal variation in potato (Solanum tuberosum L.) using chemical mutagens. The Agriculturists. 2014;12(1):15–25.
13. Opoku Gyamfi M, Eleblu JSY, Sarfoa LG, Asante IK, Opoku-Agyemang F, Danquah EY. Induced variations of ethyl methane sulfonate mutagenized cowpea (Vigna unguiculata L. Walp) plants. Front Plant Sci. 2022 Aug;13:952247.
14. Dhanavel D, Girija M. Effect of EMS, DES, and SA on quantitative traits of cowpea (Vigna unguiculata (L.) Walp.) in M1 generation. Crop Research (Hisar). 2009;37(1–3):239–41.
15. Dhanavel D, Girija M. Effect of EMS on induced morphological mutants in cowpea (Vigna unguiculata (L.) Walp.). Crop Research. 2009;38:27–31.
16. Roychowdhury R, Tah J. Assessment of chemical mutagenic effects in mutation breeding programme for M1 generation of carnation (Dianthus caryophyllus). Res Plant Biol. 2011;1(4).
17. Gnankambary TK, Benoit JB, Sawadogo N, Mahamadou S, Tignegre JB, Yonli D, et al. Genetic variability induced by gamma radiation in cowpea (Vigna unguiculata L. Walp) in Burkina Faso. Eur Sci J. 2019;15(15):153–64.
18. Adamu AK, Aliyu H. Morphological effects of sodium azide on tomato (Lycopersicon esculentum Mill). Science World Journal. 2007;2(4).
19. Amini M. Ethyl methane sulfonate. In: Wexler P, editor. Encyclopedia of Toxicology. 3rd ed. Academic Press; 2014. p. 522–4.
20. Kavina J, Ranjith VS, Sathya B. Effect of EMS on chlorophyll mutagen in fenugreek (Trigonella foenum-graecum L.). Journal of Medicinal Plants Studies. 2020;8(2):1–5.
21. Sega GA. A review of the genetic effects of ethyl methanesulfonate. Mutation Research/Reviews in Genetic Toxicology. 1984;134(2–3):113–42.
22. Prakash L, Higgins D. Role of DNA repair in ethyl methanesulfonate-induced mutagenesis in Saccharomyces cerevisiae. Carcinogenesis. 1982;3(4):439–44.
23. Wani MR, Khan S, Kozgar MI. Induced chlorophyll mutations. I. Mutagenic effectiveness and efficiency of EMS, HZ and SA in mungbean. Front Agric China. 2011;5(4):514–8.
24. Shah D, Kamili AN, Wani AA, Majeed U, Wani ZA, Sajjad N, et al. Promoting the accumulation of scopolamine and hyoscyamine in Hyoscyamus niger L. through EMS based mutagenesis. PLoS One. 2020;15(5):e0231355.
25. Odeigah PGC, Osanyinpeju AO, Myers GO. Induced mutations in cowpea: Morphological and yield traits. Mutation Breeding Newsletter. 1998;44:10–3.
26. Gnanamurthy S, Dhanavel D. Effect of EMS on induced morphological mutants and chromosomal variation in cowpea (Vigna unguiculata (L.) Walp.). International Letters of Natural Sciences. 2014;13:33–43.
27. Shu QY, Forster BP, Nakagawa H, editors. Plant Mutation Breeding and Biotechnology. CABI; 2012.
28. International Board for Plant Genetic Resources (IBPGR). Descriptors for Cowpea [Internet]. Rome, Italy: IBPGR; 1983. Available from:

https://www.bioversityinternational.org/fileadmin/_migrated/uploads/tx_news/Descriptors_for_cowpea_377.pdf

29. Nawale SR, Apte UB, Jadhav BB. Effect of gamma rays and ethyl methane sulfonate on seed germination and survival of seedlings in cowpea (Vigna unguiculata (L.) Walp.). Journal of Arid Legumes. 2006;3(1):102–5.
30. Bayhan M, Özkan R, Albayrak Ö, Akinci C, Yildirim M. Effects of ethyl methanesulfonate on growth and yield parameters of wheat and tolerance to imazamox. Bangladesh J Bot. 2024;53(3):487–93. doi:10.3329/bjb.v53i3.76260
31. Chen L, Hao L, Parry MAJ, Phillips AL, Hu YG. Current trends and insights on EMS mutagenesis application to studies on plant abiotic stress tolerance and development. Int J Mol Sci. 2023;24(3):2945.
32. Bind D, Dwivedi VK. Effect of mutagenesis on germination, plant survival, and pollen sterility in M1 generation of cowpea [Vigna unguiculata (L.) Walp]. Indian J Agric Res. 2014;48.
33. Crino P, Lai A, Bonito RD, Veronese P, Saccardo F. Genetic variability in tomato plants regenerated from irradiated cotyledons. In: Jain SM, Brar DS, Ahloowalia BS, editors. Somaclonal Variation and Induced Mutations in Crop Improvement. Dordrecht: Springer Science & Business Media; 2013.
34. Jain SM, Brar DS, Ahloowalia BS, editors. Somaclonal Variation and Induced Mutations in Crop Improvement. Dordrecht: Springer Science & Business Media; 2013.
35. Omar MA, El-Sayed HA, Hassan RM. Differential response of faba bean (Vicia faba L.) genotypes to EMS mutagenesis: Germination and early growth traits. Legume Research. 2025;48(1):55–62.
36. Ramadan SM, Abdel-Hamid AM, El-Metwally MA. Influence of EMS mutagenesis on germination and seedling traits of soybean (Glycine max L.). J Crop Improv. 2025;39(2):210–22.
37. Chen L, Wang T, Zhao M, Zhang Y. Effect of EMS mutagenesis and post-treatment handling on seed germination in cucumber (Cucumis sativus L.). Mutation Breeding Journal. 2015;45(2):123–30.
38. Rakau M, Tesfaye K, Bekele E. Optimization of EMS mutagenesis in tef (Eragrostis tef) reveals genotype-dependent germination responses. Plant Genetic Resources. 2025;23(1):34–42.
39. Sharamo MA, Hussein S, Mohammed A. Evaluation of EMS-induced mutagenesis on germination and survival of barley (Hordeum vulgare L.). Afr J Agric Res. 2021;17(4):567–74.
40. Rhaman MS, Rauf F, Tania SS, Khatun M. Seed priming before the sprout: Revisiting an established technique for stress-resilient germination. Seeds. 2025;4(3):29.
41. Ke C, Guan W, Bu S, Li X, Deng Y, Wei Z, et al. Determination of absorption dose in chemical mutagenesis in plants. PLoS One. 2019;14(1):e0210596.
42. Kumari P. Effect of EMS and colchicine treated seeds on plant growth and flowering in marigold. [Solan, India]: Dr Yashwant Singh Parmar University of Horticulture and Forestry; 2024.
43. Ishiyaku MF, Singh BB. Inheritance of time to flowering in cowpea (Vigna unguiculata (L.) Walp.). Euphytica. 2005;142:291–300. doi:10.1007/s10681-005-2435-0

44. Nair R, Mehta AK. Induced mutagenesis in cowpea [Vigna unguiculata (L.) Walp.] var. Arka Garima. Indian J Agric Res. 2014;48(4):247–57. doi:10.5958/0976-058X.2014.00658.1
45. Ramolekwa K, Pholo-Tait M, Parker T, Malambane G, Ngwako S, Lekgari L. Estimating genetic variability and heritability of morpho-agronomic traits of M_5 cowpea (Vigna unguiculata (L.) Walp) mutant lines. Int J Mol Sci. 2025;26(15):7543. doi:10.3390/ijms26157543
46. Adomako-Mensah K, others. Induced variations of ethyl methane sulfonate mutagenized cowpea (Vigna unguiculata L. Walp) plants. Front Plant Sci. 2022;13:952247. doi:10.3389/fpls.2022.952247
47. Dow el-Madina IM, Hall AE. Flowering of contrasting cowpea (Vigna unguiculata (L.) Walp.) genotypes under different temperatures and photoperiods. Field Crops Res. 1986;14:87–104.
48. Jain SM. Mutagenesis in crop improvement under the climate change. Rom Biotechnol Lett. 2010;15(2):88–106.
49. Jain SM, Maluszynski M. In vitro applications in crop improvement: Recent progress. In: Mujib A, Cho M, Predieri S, Banerjee S, editors. Biotechnology for Crop Improvement. New Delhi, India: IBH–Oxford; 2004. p. 169–202.
50. Kumar A, Rai PK. Induced mutagenesis in mungbean (Vigna radiata L.). In: Singh DP, editor. Mutation Breeding in Pulse Crops. Kalyani Publishers; 2006. p. 85–93.
51. Kundu SK, Singh DP. Gamma-ray induced variability for quantitative characters in black gram. Madras Agricultural Journal. 1982;69(10):644–8.
52. Chandra N. Cytological and mutational studies on Gloriosa superba Linn. [Nagpur, India]: Nagpur University; 1988.
53. Khan S, Wani MR. Comparison of the effect of chemical mutagens on mung bean. Advances in Plant Sciences. 2005;18(2):533–5.
54. Khan S, Wani MR. Chemomutagenic effect of two alkylating agents on mung bean (Vigna radiata). Bionotes. 2004;6(3):82–3.
55. Nanda S, Rao GM, Nayak S. Mutagenic effects of EMS on yardlong bean (Vigna sesquipedalis). Indian J Agric Res. 1997;31:602–10.
56. Allard RW. Principles of Plant Breeding. 2nd ed. New York: John Wiley & Sons; 1999.
57. Eswaramoorthy V, Kandasamy T, Thiyagarajan K, Chockalingam V, Jegadeesan S, Natesan S, et al. Characterization of terminal flowering cowpea (Vigna unguiculata (L.) Walp.) mutants obtained by induced mutagenesis reveals loss-of-function of phosphatidylethanolamine-binding protein. PLoS One. 2023;18(12):e0295509. doi:10.1371/journal.pone.0295509 PubMed PMID: 38096151.
58. Vasanthrao T, Sarma D, Barua PK, Sharma KK, Kalita P, Gogoi S. Mutagenic Effects of Ethyl Methane Sulphonate (EMS) on Quantitative Traits in M 1 Generation and the Effectiveness and Efficiency of Different Doses in Garden Pea (Pisum sativum L.). Legume Research: An International Journal. 2025;48(8).
59. Nair AS, Gayathri G, Thomas UC, Thomas B, Varanya A, Hulsure P. Genetic improvement of fodder cowpea through induced mutagenesis. RANGE MANAGEMENT AND AGROFORESTRY. 2025;46(1):165–9.
60. Gyamfi MO. Inducing Genetic Variability in Cowpea (Vigna unguiculata, L. Walp Var. Asontem) Using Ethyl Methane Sulfonate [Internet]. 2022. Available from: http://ugspace.ug.edu.gh

61. Zhang X, Zhai H, Wang Y, Tian X, Zhang Y, Wu H, et al. Functional conservation and diversification of the soybean maturity gene E1 and its homologs in legumes. Sci Rep. 2016;6(1):29548.
62. Falconer DS, Mackay TFC. Introduction to Quantitative Genetics. 4th ed. Harlow: Longman; 1996.
63. Grafius JE. Components of yield in oats: A geometrical interpretation. Agron J. 1959;51:515–8.
64. Gaul H. Mutations in plant breeding. Radiation Botany. 1964;4:155–232.
65. Maluszynski M, Nichterlein K, Van Zanten L, Ahloowalia BS. Officially released mutant varieties—The FAO/IAEA database. Mutation Breeding Reviews. 2000;12:1–84.
66. Goyal P, Khan S, Wani MR. EMS-induced genetic variability for yield and yield components in urdbean (Vigna mungo (L.) Hepper). Legume Research. 2021;44:1220–6.
67. Premsekhar S, Appadurai P. Effect of doses of gamma rays and EMS on induced mutation in pigeonpea. Indian Journal of Agricultural Sciences. 1982;51:381–4.
68. Weller JL, Ortega R. Genetic control of flowering time in legumes. Front Plant Sci. 2015;6:207. doi:10.3389/fpls.2015.00207 PubMed PMID: 25914700.
69. Pathak RS. Genetic evaluation of two aphid resistant cowpea mutants in Kenya. In: Proceedings of the IAEA Symposium on Plant Mutation Breeding for Crop Improvement. Vienna, Austria: International Atomic Energy Agency; 1991. p. 463.
70. Fujii T, Tano S. Mutagenic activities of EMS on somatic (M1) and recessive (M2) mutations in the soybean test system. Environ Exp Bot. 1986;26(2):191–5. doi:https://doi.org/10.1016/0098-8472(86)90015-8
71. Lo S, Muñoz-Amatriaín M, Boukar O, Herniter I, Cisse N, Guo YN, et al. Identification of QTL controlling domestication-related traits in cowpea (Vigna unguiculata L. Walp). Sci Rep. 2018;8(1):6261. doi:10.1038/s41598-018-24349-4 PubMed PMID: 29674702.
72. Dorvlo KI, Acquah AS, Armah OJ, Kutufam TJ, Afutu E, Darkwa AA, et al. Induction of genetic variability in cowpea variety Videza for extra earliness and high seed yield using gamma irradiation mutagenesis. Legume Research. 2022;45(9):1074–81. doi:10.18805/LRF-685
73. Rai A. Effect of Ethyl Methane Sulfonate Mutagen in M1 Generation in Pigeonpea (Cajanus cajan L. Millasp). 2011.
74. Khan IA. Mutation studies in mung bean (Vigna radiata L. Wilczek): IX. Estimates of genetic variability. Legume Research. 1988;11(2):89–93.
75. Singh M, Singh VP. Genetic analysis of certain mutant lines of urdbean for yield and quality traits in M4 generation. Indian Journal of Pulses Research. 2001;14(1):60–2.
76. Jagajanantham N, Dhanavel D, Pavadai P. EMS-induced variability in okra (Abelmoschus esculentus L.). International Journal of Science and Research. 2013;2:148–51.
77. Berenschot AS, Zucchi MI, Tulmann Neto A, Quecini V. Mutagenesis in ornamentals: Application and perspective. Plant Cell Tissue Organ Cult. 2009;98:161–72.
78. Premsekar R, Appadurai R. EMS-induced variability in pigeonpea (Cajanus cajan L.). Madras Agricultural Journal. 1982;69:458–66.
79. Mohanasundaram K, Kalamani A, Ramalingam RS. Induced mutagenesis in cowpea (Vigna unguiculata (L.) Walp.). Legume Research. 1999;22:642–8.

80. Anis M, Sharma PK, Abbasi N. Gamma rays induced variability in urd bean (Vigna mungo (L.) Hepper) in M1 generation. Journal of the Indian Botanical Society. 1999;78(1–2):111–3.
81. Khan SB, Siddiqui A, Nadeem M. Variation in quantitative characters of mung bean after seed treatment with DES. Advances in Plant Sciences. 1994;7(1):41–5.
82. Kansagra UM, Shukla PT. Variability studies in induced mutants of mungbean. Madras Agricultural Journal. 1993;80(4):180–4.
83. Iepziba SJ, Subramanian M. Variability in M3 and M4 mutants of urdbean. Indian Journal of Pulses Research. 1993;6(2):197.
84. Patil BN, Guha VY. Growth attributes in induced mutants of chickpea. PKV Research Journal. 1994;15(2):97–101.
85. Sie EK, Issahaku M, others. Genetic variability, heritability and correlation analysis among maturity and yield traits in cowpea (Vigna unguiculata (L.) Walp) in northern Ghana. Heliyon. 2021;7(9):e07955. doi:10.1016/j.heliyon.2021.e07955
86. Ramolekwa K, Pholo-Tait M, Parker T, Malambane G, Ngwako S, Lekgari L. Estimating genetic variability and heritability of morpho-agronomic traits of M5 cowpea (Vigna unguiculata (L.) Walp) mutant lines. Int J Mol Sci. 2025;26(15):7543. doi:10.3390/ijms26157543
87. Chaudhary D, Gore PG, Wankhede DP, Patil DK, Choudhary SB, Rao AM, et al. Morphological variability and genetic diversity assessment of agronomic traits in cowpea (Vigna unguiculata (L.) Walp.) core collection using multivariate analysis. Genet Resour Crop Evol. 2025;72(5):6027–42. doi:10.1007/s10722-024-02299-3
88. Cao Z, Li J, Lei H, Yan M, Wang Q, Ji R, et al. PCA-Driven Multivariate Trait Integration in Alfalfa Breeding: A Selection Model for High-Yield and Stable Progenies. Plants. 2025;14(18):2906. doi:10.3390/plants14182906 PubMed PMID: 41012057.
89. Nkomo G V, Sedibe MM, Mofokeng MA. Phenotyping cowpea accessions at the seedling stage for drought tolerance using the pot method. bioRxiv. 2020. doi:10.1101/2020.07.10.196915
90. Wani AA. Induced mutagenesis for improvement of pulse crops. Plant Breed Rev. 2017;41:155–200.
91. Diouf M, Diallo S, Badiane FA, Diack O, Diouf D. Development of new cowpea [Vigna unguiculata (L.) Walp] mutant genotypes, analysis of their agromorphological variation, genetic diversity and population structure. Biocell. 2021;45(2):345–62. doi:10.32604/biocell.2021.013706
92. Mkhonta KAK, Shimelis H, Abady S, Ngidi A. Agronomic Performance of Newly Developed Elite Cowpea Mutant Lines in Eswatini. Agriculture. 2025;15(15). doi:10.3390/agriculture15151631
93. Raina A, Khan S. Field assessment of yield and its contributing traits in cowpea treated with lower, intermediate, and higher doses of gamma rays and sodium azide. Front Plant Sci. 2023;14:1188077. doi:10.3389/fpls.2023.1188077
94. Raina A, Laskar RA, Wani MR, Jan BL, Ali S, Khan S. Comparative Mutagenic Effectiveness and Efficiency of Gamma Rays and Sodium Azide in Inducing Chlorophyll and Morphological Mutants of Cowpea. Plants. 2022;11(10):1322. doi:10.3390/plants11101322 PubMed PMID: 35631747.

95. Raina A, Laskar RA, Wani MR, Jan BL, Khan S. Characterization of induced high yielding cowpea mutant lines using physiological, biochemical and molecular markers. Sci Rep. 2020;10:3687. doi:10.1038/s41598-020-60601-6

Supporting Information

S1 Table. Trait dataset and cluster grouping of individual M_1 cowpea plants across EMS treatments and control

S2 Table. PCA loadings and variance explained for traits in M_1 cowpea

Fig 1

Click here to access/download;Figure;Fig 1.tif.tif

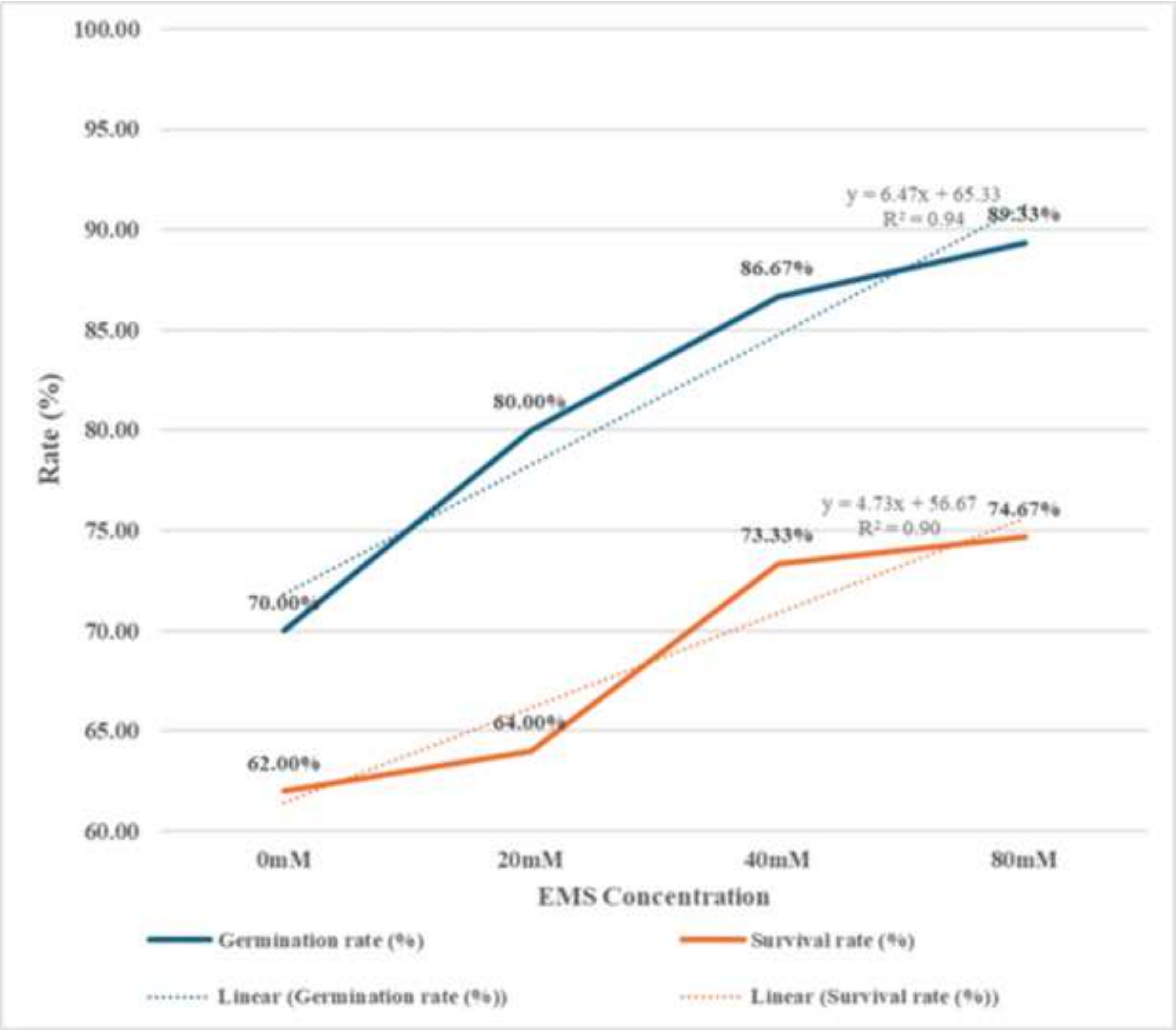

Fig 2

Click here to access/download;Figure;Fig 2.tif.tif

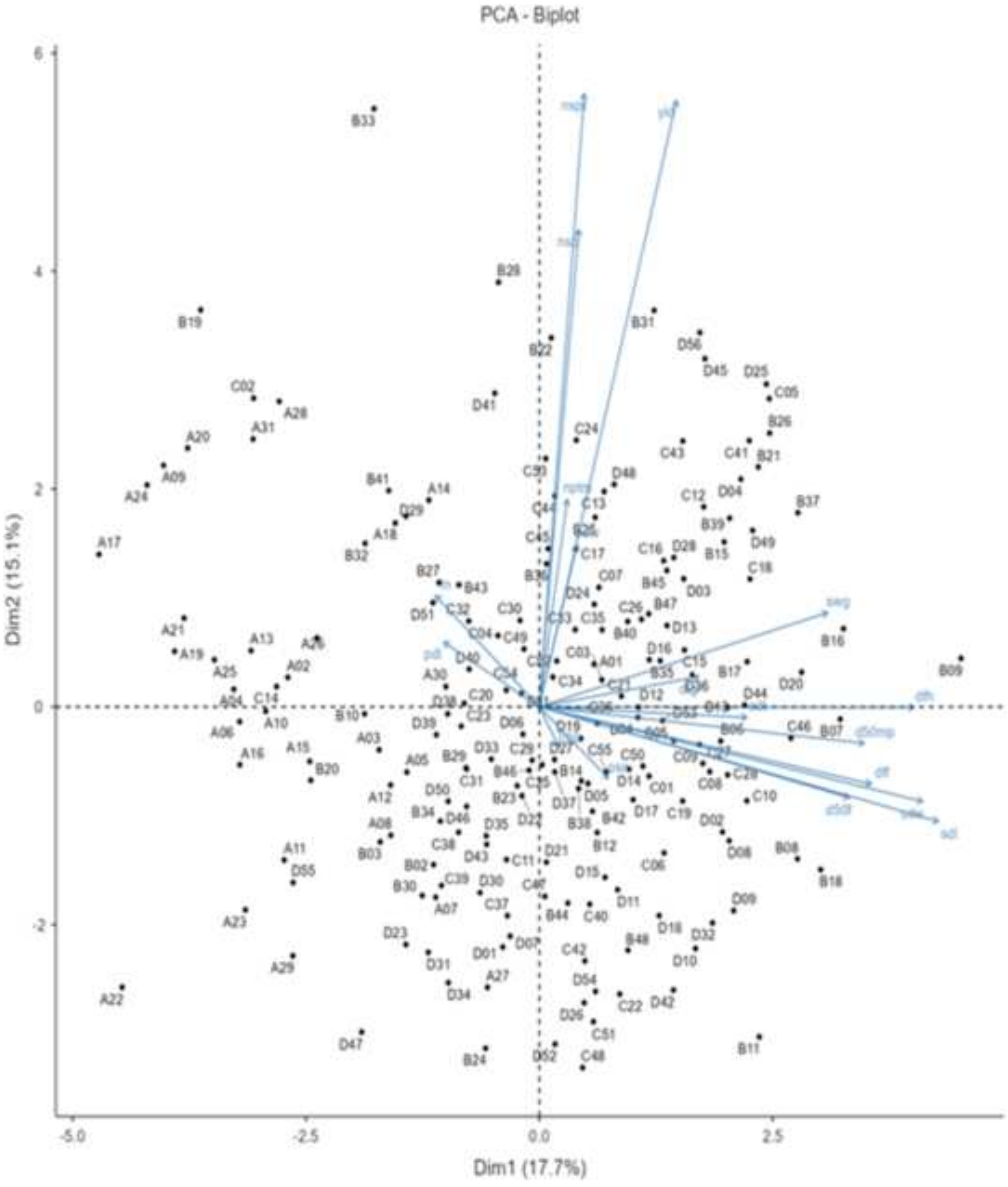

Fig 3

Click here to access/download;Figure;Fig 3.tif.tif

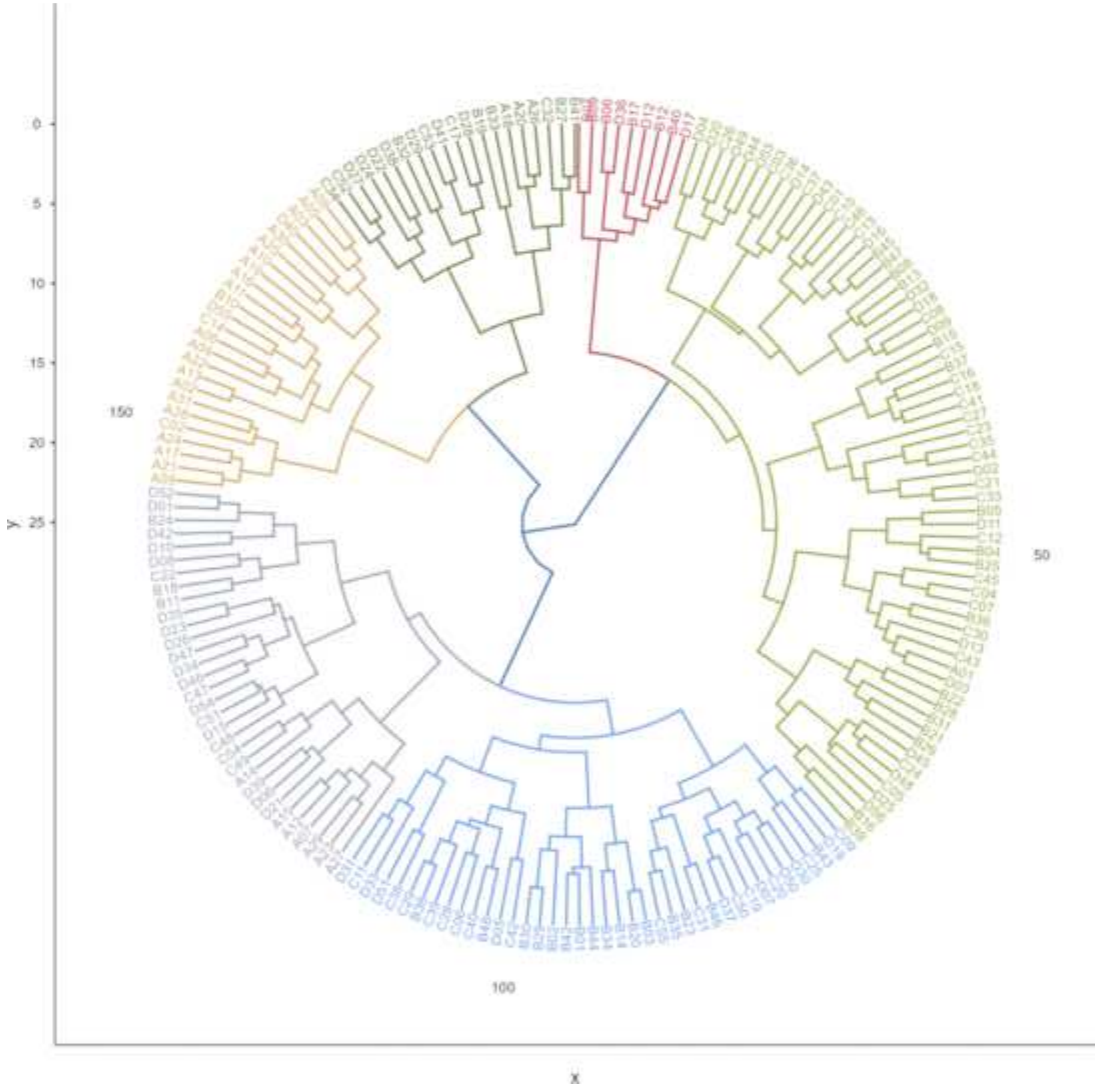

S1 Table

Click here to access/download
Supporting Information
S1_Table.pdf

S2 Table

Click here to access/download
Supporting Information
S2_Table.pdf

Effect of Ethyl Methane Sulfonate Mutagenesis on Phenological, Yield-Related and Yield Traits in Cowpea *(Vigna unguiculata* (L.) Walp)

Henry .K. Mensah[1¶*], Ralieva A.K. Norety[1¶] , Isaac .K. Asante[1&], Felicia Oppong[1&]

[1]Department of Plant and Environmental Biology, University of Ghana, Accra, Ghana

*Corresponding author

henryquofi@gmail.com (HKM)

.

[¶] These authors contributed equally to this work.

[&]These authors also contributed equally to this work.

Abstract

This study investigated the mutagenic effects of ethyl methane sulfonate (EMS) on the M_1 generation of cowpea (Vigna unguiculata (L.) Walp.) cultivar 'Wang Kae'. A total of 275 M_0 seeds were treated with EMS concentrations of 20 mM, 40 mM, and 80 mM (75 seeds per treatment) by soaking for six hours, while 50 untreated seeds served as the control (0 mM). Phenological, yield-related and yield traits were recorded, and data were analysed using Jamovi 2.7.15 and JASP 0.95.4.0 through one-way ANOVA with post hoc contrast, principal component biplot, and cluster analyses. No optimal mutagenic concentration (LD50) was identified. Seed germination and seedling survival rates increased with increasing EMS concentration, ranging from 70.00% and 62.00% in the control (0 mM) to 89.33% and 74.67% at 80 mM, following the trend 0 mM < 20 mM < 40 mM < 80 mM. Significant differences ($P < 0.05$) were observed among treatments for all phenological traits, pod length, locule number, seed traits, and yield per plant. Yield was significantly higher ($P = 0.047$) at 20 mM (61.19 ± 3.34 g) compared to the control. Contrast analysis identified genotypes B33 and D56 as the most productive mutants, with yields of 125.44 g and 111.85 g, respectively. Principal component analysis extracted eighteen components, with the first four cumulatively explaining 50.60% of total variation. Biplot analysis of PC1 and PC2 captured all phenological traits, key seed traits, and yield attributes, highlighting the superior performance of B33 and D56. Cluster analysis partitioned the 190 genotypes into six groups, with B33 and D56 constituting distinct clusters. EMS mutagenesis effectively induced heritable phenotypic variation, with putative superior genotypes identified for advancement to M_2 and evaluation in replicated multi-environment trials toward the development of farmer- and consumer-preferred cowpea varieties.~~This study investigated the mutagenic effects of ethyl methane sulfonate (EMS) on the M_1 generation of cowpea (*Vigna unguiculata* (L.) Walp.) cultivar '*Wang Kae*'. A total of 275 M_0 seeds were treated with EMS concentrations of 20 mM, 40 mM, and 80 mM (75 seeds~~

Formatted: Line spacing: Double

per treatment) by soaking for six hours, while 50 untreated seeds served as the control (0 mM). Phenological, yield-related and yield traits were recorded, and data were analysed using Jamovi 2.7.15 and JASP 0.95.4.0 through one-way ANOVA with post hoc contrast, principal component biplot, and cluster analyses.

No optimal mutagenic concentration (LD50) was identified. Seed germination and seedling survival rates increased with increasing EMS concentration, ranging from 70.00% and 62.00% in the control (0 mM) to 89.33% and 74.67% at 80 mM, following the trend 0 mM < 20 mM < 40 mM < 80 mM. Significant differences ($P < 0.05$) were observed among treatments for all phenological traits, pod length, locule number, seed traits, and yield per plant. Yield was significantly higher ($P = 0.047$) at 20 mM (61.19 ± 3.34 g) compared to the control. Contrast analysis identified genotypes B33 and D56 as the most productive mutants, with yields of 125.44 g and 111.85 g, respectively.

Principal component analysis extracted eighteen components, with the first four cumulatively explaining 50.60% of total variation. Biplot analysis of PC1 and PC2 captured all phenological traits, key seed traits, and yield attributes, highlighting the superior performance of B33 and D56. Cluster analysis partitioned the 190 genotypes into six groups, with B33 and D56 constituting distinct clusters. EMS mutagenesis effectively induced heritable phenotypic variation, with putative superior genotypes identified for advancement to M_2 and evaluation in replicated multi-environment trials toward the development of farmer- and consumer-preferred cowpea varieties.

Introduction

Cowpea (*Vigna unguiculata* [L.] Walp.) is a widely cultivated and nutritionally important legume, predominantly grown in tropical and subtropical regions, particularly in Sub-Saharan Africa, Asia, and Central and South America, while also being adapted to certain temperate zones such as the Mediterranean basin and the southern regions of the United States [1,2]. Cowpea (*Vigna unguiculata* L. Walp.), is an important staple food legume and cheap source of protein for many Africans in the low-land humid and dry savannah tropics. It is an excellent substitute for animal proteins by resource-poor people and vegetarians because of its high seed protein content (about 25%) and rich amino acids [3]. Indeed, some cultivars with seed protein content of about 30%, close to that obtained for soybean (*Glycine max*) have been reported [4,5]. Due to a decline in cowpea market value caused by pests, pathogens, and a lack of genetic variability, many scientists worldwide have sought to develop cowpea varieties that meet the taste and demand of a growing population [6-9]. Previous studies have demonstrated substantial genetic variability in cowpea for agronomic and economically important traits, including protein and mineral composition, underscoring the need to broaden the genetic base through breeding or mutagenesis [10,11]. The most noteworthy studies have used mutagens to increase genetic variability [6-9,12-14]. Ethyl Methane Sulfonate (EMS), one of the most effective mutagens, has been successfully used by several researchers in West Africa to improve genetic variability in cowpea [8,15].

A mutation is a heritable alteration in the structure or nucleotide sequence of a gene that arises suddenly and can be transmitted to subsequent generations. It results from changes in DNA such as substitutions, insertions, deletions, or rearrangements, and may occur naturally through replication or repair errors or be intentionally induced by physical and chemical mutagens. In plants, mutations can be fixed in both seed-propagated and vegetatively propagated species, allowing stable transmission of new traits. Consequently, mutations constitute a fundamental source of genetic variation and play a critical role in plant breeding

and crop improvement programs [16,17]. To create functional mutants in various crop species, a variety of chemical mutagens have been used. Artificial mutation induction through chemical mutagens, including colchicine (Col), ethyl methanesulfonate (EMS) and sodium azide (SA), has been widely recognized as an effective approach for enhancing genetic variability in plants. This technique facilitates the improvement of specific traits while preserving the inherent desirable attributes of the plant, as documented by several researchers [16]. Chemical mutagens generally produce induced mutations which lead to base pair substitutions, especially GC→AT resulting in amino acid changes, which change the function of proteins but do not abolish their functions as like deletions or frame shift mutations. These chemo mutagens induce a broad variation in morphological characters when compared to normal plants [18].

Ethyl methane sulfonate is a colorless mutagenic, carcinogenic and teratogenic chemical compound with the chemical formula $CH_3OSO_2C_2H_5$. It is formed from the condensation of ethanal with methane sulfonic acid [19]. Ethyl methane sulphonate is an alkylating chemical mutagen that has been widely utilized in plant breeding because it can generate a high frequency of gene mutations. EMS produces O_6-ethyl guanine by alkylating guanine residues. This matches thymine (T) but not cytosine (C) as a result, unrepaired alkylation replication occurs. A damaged pair A/T will effectively replace the G/C base pair. This mechanism foresees a strong G/C to A/T bond shortly [20]. According to [21,22], EMS is a monofunctional ethylating agent that has been reported to be mutagenic in a range of genetic test systems, ranging from viruses to mammals. Effectiveness and efficiency mean the extent to which the mutagenic agent produced a desired effect on the plant which may be positive or negative effect. [23] reported the order of effectiveness of chemical mutagens as hydrazine hydrate (HZ)> sodium azide (SA)> ethyl methane sulphonate (EMS) as they did a systematic and comparative study of induced chlorophyll mutation using HZ, SA and EMS and found that EMS treatments caused the highest frequency of chlorophyll mutations followed by

HZ and then SA. [23] & [24] reported that mutations of chemical mutagens and their effectiveness is dose-dependent and increase with the concentration of mutagens. [24] reported that lower concentrations of EMS are more effective than higher concentrations. Ethyl methane sulfonate is used by plant breeders because of its ease of use, good penetration, reproducibility, high mutation frequency and lack of disposal issues.

According to [6], plant breeders' major goal is to create crops that outperform existing cultivars in terms of production and quality, and this is dependent on the availability of genetic variation, preferably in the primary gene pool. Genetic variety is necessary for crop improvement. Plant breeders, therefore, have begun to use mutation breeding more frequently such as [13] who in their research induced variations using EMS in cowpea (*Vigna unguiculata* L. Walp Var. *Asontem*) in the M_1 and M_2 generations at different concentrations of EMS (0.0, 0.2, 0.4, 0.6, and 0.8%) for 16h which showed effective variations in traits such as germination and percentage survival, yield and yield related traits in M_1 population. [25] and [26] both subjected cowpea to EMS at different does for 6 hours, also reported higher number of pods per plant, early and late phenological traits, changes in pigmentation of leaf, pod and flower colour, plant height, increased pod length and high seed weights as a result of EMS-induced mutations. During the M_1 generation, only dominant or co-dominant mutations are easily detected by phenotypic observations [27]. Some viable mutations in the M_1 generation include reduction in plant height, pollen sterility, late or early flowering and podding. Furthermore, most previous studies have focused on later generations (M_2 and beyond), where mutations are more stably expressed, with fewer studies providing detailed analyses of variability in the M_1 generation. Understanding the immediate effects of EMS in the M_1 population is crucial for optimizing mutagenesis protocols and identifying early indicators of useful genetic variation.

Therefore, this study aimed to evaluate the effects of different EMS concentrations on seed germination and survival, phenological, yield-related and yield traits in the M_1 generation

of cowpea cultivar *'Wang Kae'*, with the objective of identifying putative superior genotypes for advancement to the M_2 generation and subsequent evaluation in replicated multi-environment trials. The ultimate goal was to support the development of improved cowpea varieties with farmer- and consumer-preferred traits for integration into breeding programs. By characterizing the phenotypic variability induced at the M_1 stage, this study contributes to refining EMS-based mutation breeding strategies in cowpea.

Materials & Methods

Experimental site, material and design

The field study was carried out at the Department of Plant and Environmental Biology, University of Ghana, from early April to July 2025. A total of 275 seeds of the '*Wang Kae*' cowpea variety (M_0 generation) were used in this study. Seventy-five seeds each were treated with three different concentrations of ethyl methane sulfonate (EMS): 20 mM, 40 mM, and 80 mM, and were soaked in the respective EMS solutions for six hours. Fifty seeds were soaked without treatment and served as the control (0 mM EMS). Genotypes were assigned alphanumeric labels to indicate both the EMS treatment level and the individual plant number. The alphabetic prefix represented the EMS concentration, while the numeric suffix denoted the individual plant within that treatment group. Specifically, '***A***' represented the control group (0 mM EMS), whereas '***B***', '***C***', and '***D***' represented the 20 mM, 40 mM, and 80 mM EMS concentrations, respectively. The numbers following each letter corresponded to the sequential numbering of plants within each treatment group. Thus, genotypes from the control (0 mM EMS) were labelled A1–A31, those treated with 20 mM EMS were labelled B1–B48, genotypes exposed to 40 mM EMS were designated C1–C55 and those treated with 80 mM EMS were labelled D1–D56.

The treated seeds were thoroughly washed in running water for 30 minutes to reduce the residual effect of the mutagen sticking to the seed coat. The treated seeds, along with the

control, were planted to raise the M_1 generation, and standard cultural practices were carried out thereafter. The experiment was designed in pots measuring 3.29 m^2. Spacing used was 0.8m between lines and 0.5 m between rows. One cowpea seed was planted per hole in each pot, at a depth of 3-4 cm.

Parameters studied in the M_1 generation

All traits studied were measured and/or counted using standard cowpea descriptor from [28]. The growth, yield-related and yield parameters studied in the M1 generation were grouped as phenological: days to germination (*dtg*), days to first flower (*dff*), days to 50% flowering (*d50f*), days to first harvest (*dfh*), days to 50% mature pod (*d50mp*); and yield-related traits: peduncle length at first harvest (*pel*), number of pods per peduncle (*npp*), number of pods per plant at maturity (*nptm*), number of seeds per pod (*nsp*), number of seeds per plant (*nspt*), pod length (*pdl*), pod width (*pdw*), number of locules (*ln*), percent seed abortion (*psa*). Seed dimensions included seed length (*sdl*), seed width (*sdw*) and seed thickness (*sdt*),while other parameters measured were seed weight (*swg*) and yield per plant (*yld*).

Percentage seed abortion was calculated as;

$$\frac{[(number\ of\ locules - number\ of\ seed\ per\ pod)]}{number\ of\ locules}\ x\ 100\% \ldots\ldots\ldots 1$$

Yield (g) was calculated as: $\frac{100\ seed\ weight\ (g)\ x\ number\ of\ seeds\ per\ plant}{100} \ldots\ldots\ldots 2$

Seed germination was recorded 7 days after planting. Survival plants were recorded at 50% flowering, and germination and survival rate were recorded using the formula by [29]

$$Germination\ rate\ (\%) =$$

$$\frac{(number\ of\ germinated\ seeds)}{(number\ of\ (un)treated\ seeds\ planted\ )}\ x\ 100 \ldots\ldots\ldots 3$$

$$Survival\ rate\ (\%) = \frac{(number\ of\ survived\ seedlings)}{(number\ of\ germinated\ seeds\ )}\ x\ 100 \ldots\ldots\ldots 4$$

[29]

Statistical analysis

Regression seedling germination and survival rate

XLSTAT was also used to generate the linear regression equation of seedling germination and survival rate.

Distribution and post hoc contrast analysis for the selection of putative traits and mutants'

Descriptive statistics, one-way ANOVA and post hoc contrast analysis were used to compare treatment effects for each trait in Jamovi 2.7.15.0. Plants within treatments whose values fell outside the control range for each character and were statistically significant from the control were selected as putative mutants**.**

Multivariate analysis for the verification of the selected putative mutant traits and genotypes

Principal Component Analysis (PCA) was performed, accompanied by biplot analysis in Jamovi 2.7.15.0. Principal Component Analysis for quantitative traits was employed to determine the percentage contribution of each trait to total genetic variation. The principal components (PCs) with eigenvalues >1 were selected and traits with coefficients >0.3 were considered to have significantly contributed to the variability. The biplot of computed similarity values was used to visualize relationships among traits across EMS treatment groups and genotypes, to assess the distribution of treatment groups, examine trait vector orientations and identify putative mutants based on their displacement from the control.Cluster analyses were performed using Jamovi 2.7.15.0 to group genotypes within treatments using the Ward D2 hierarchical clustering optimization criterion. A circular dendrogram constructed from computed similarity values was used to visualize relationships among genotypes and traits that differed significantly among treatments, with the resulting clusters interpreted to characterize the phenotypic groupings produced by mutagenic treatment.

Results

Percentage germination and survival of M_1 plants

The results for seed germination and survival rates (%) and linear regression fits for M_1 cowpea genotypes subjected to three EMS doses for 6 h are presented in Fig 1. The highest seed germination rate (89.33%) and seedling survival rate (74.67%) were recorded at 80 mM, whereas the lowest germination rate (70.00%) and seedling survival rate (62.00%) were observed in the 0 mM (control) treatment. The percentages of seed germination and seedling survival were in this order: 0 mM < 20 mM < 40 mM < 80 mM. Linear regression analysis revealed a strong positive relationship between EMS concentration and both seed germination and survival rate (Fig 1). For seed germination rate, the regression equation was $y = 6.47x + 65.33$ with an R^2 value of 0.94. Seedling survival showed a positive linear relationship with EMS concentration as follows: $y = 4.73x + 56.67$ with an R^2 value of 0.90.

Fig 1: Seed germination and survival rates (%) and linear regression fits for M_1 cowpea genotypes subjected to three ethyl methanesulfonate (EMS) doses for 6 h.

Contrast analysis of traits in the M_1 generation

Phenological traits

Days to germination (dtg) differed significantly among treatments ($P < 0.001$). The control (0 mM) germinated earliest, with a mean of 3.45 ± 0.13 days and a range of 2–5 days. All EMS treatments delayed germination, with the most substantial effect at 20 mM, while 40 and 80 mM showed delayed but comparable responses. The frequency of putative mutants was 50.0% at 20 mM, 29.10% at 40 mM, and 30.36% at 80 mM (Table 1). Selected putative mutants can be found in S1 Table.

Table 1 : Mean, variability, mutant frequency, and pairwise significance of phenological traits in the M_1 generation of cowpea under different EMS concentrations.

Trait	N	Trt	Mean±se	%CV	Min	Max	Freq	P	T-values of significant pairwise comparisons			
									0mM	20mM	40mM	80mM
dtg	31	0mM	$3.45 \pm 0.13_{a}$	20.90	2	5		***		-6.38***	-4.53***	-4.50***
	48	20mM	$5.38 \pm 0.20_{b}$	25.30	3	7	50.00		-6.38***			
	55	40mM	$4.78 \pm 0.19_{b}$	29.20	3	7	29.10		-4.53***			
	56	80mM	$4.77 \pm 0.19_{b}$	29.70	3	7	30.36		-4.50***			
dff	31	0mM	$37.87 \pm 0.77_{a}$	11.30	30	45		***		-5.00***	-5.40***	-4.97***
	48	20mM	$44.69 \pm 0.91_{b}$	14.10	33	54	51.17		-5.00***			
	55	40mM	$45.04 \pm 0.86_{b}$	14.10	34	55	47.27		-5.40***			
	56	80mM	$44.46 \pm 0.79_{b}$	13.30	33	55	41.07		-4.97***			
d50f	31	0mM	$57.84 \pm 0.92_{a}$	8.90	48	68		***		-4.51***	-4.98***	-4.72***
	48	20mM	$64.67 \pm 0.99_{b}$	10.70	50	75	37.50		-4.51***			
	55	40mM	$65.18 \pm 0.97_{b}$	11.00	49	80	40.00		-4.98***			
	56	80mM	$64.79 \pm 0.85_{b}$	9.80	53	80	28.57		-4.72***			
dfh	31	0mM	$49.35 \pm 1.15_{a}$	13.00	40	67		***		-6.93***	-6.93***	-6.04***
	48	20mM	$60.17 \pm 0.94_{b}$	10.80	48	72	18.75		-6.93***			
	55	40mM	$58.93 \pm 0.97_{b}$	12.10	38	72	9.100		-6.93***			
	56	80mM	$58.50 \pm 0.91_{b}$	11.70	43	72	12.50		-6.04***			
d50mp	31	0mM	$79.48 \pm 1.41_{a}$	9.90	66	94		***		-5.40***	-4.75***	-4.59***
	48	20mM	$90.17 \pm 1.37_{b}$	10.50	68	107	39.58		-5.40***			
	55	40mM	$88.65 \pm 1.09_{b}$	9.10	68	106	25.45		-4.75***			
	56	80mM	$88.32 \pm 1.16_{b}$	9.80	69	110	23.21		-4.59***			

Note. Means sharing the same superscript letter within a trait are not significantly different according to Tukey's HSD test ($P \leq 0.05$). Trt= Treatment, N=Sample size, Mean ± SE = Mean plus or minus standard error, %CV= Percentage coefficient of variation, Min= minimum, Max= maximum, Freq= Percentage frequency of putative mutants; P=Probability value. Traits: dtg = days to germination, dff = days to first flower, d50f = days to 50% flowering, dfh = days to first harvest, d50mp = days to 50% mature pods, *=significance at 5% level, **=significance at 1% level, ***=significance at 0.1% level

Days to first flower (dff) were significantly affected by EMS across treatments ($P < 0.001$). *DFF* was the earliest in the control, with a mean of 37.87 ± 0.77 days and a range of 30 to 45 days, and was progressively delayed with increasing EMS concentration, reaching a maximum mean of 45.04 ± 0.86 days at 40 mM. Mutant frequencies declined from 51.17% at 20 mM to 41.07% at 80 mM (Table 1). Selected putative mutants can be found in S1 Table.

Days to 50% flowering (d50f) were significantly affected by EMS across treatments ($P < 0.001$). *D50f* increased from a control mean of 57.84 ± 0.92 days, with values ranging from 48 to 68 days, to mean values of approximately 65 days across EMS treatments, with broader range of 49–80 days. Corresponding mutant frequencies were 37.50%, 40.00%, and 28.57% at 20, 40, and 80 mM, respectively (Table 1). Selected putative mutants can be found in S1 Table.

Days to first harvest (dfh) were the earliest in the control, with a mean of 49.35 ± 1.15 days and a range of 40–67 days and were significantly ($P < 0.001$) delayed in all EMS treatments. Mean dfh increased to 60.17 ± 0.94 days at 20 mM, 58.93 ± 0.97 days at 40 mM,

and 58.50 ± 0.91 days at 80 mM. Mutant frequencies were 18.75% at 20 mM, 9.10% at 40 mM, and 12.50% at 80 mM (Table 1). Selected putative mutants can be found in S1 Table.

A similar trend was observed for days to ***50% maturity of pods (d50mp)***, with the control recording the earliest maturity at 79.48 ± 1.41 days, and values ranging from 66 to 94 days. EMS treatments significantly ($P<0.001$) delayed pod maturity compared with the control, with mean d50mp values of 90.17 ± 1.37 days at 20 mM, 88.65 ± 1.09 days at 40 mM, and 88.32 ± 1.16 days at 80 mM. The frequency of selected mutants for d50mp was 39.58%, 25.45%, and 23.21% at 20, 40, and 80 mM, respectively (Table 1). Selected putative mutants can be found in S*1* Table.

Yield-related and yield traits

Pod length (pdl) was highest in the control (0 mM), with a mean of 14.65 ± 0.31 cm and a range of 11.55–17.90 cm, whereas 80mM recorded the lowest mean pdl of 13.52±0.28 cm, which was significantly different ($P=0.008$) from the control. Based on deviations from the control range, the frequency of selected mutants for pdl was 10.42%, 9.10%, and 21.43% in 20 mM, 40 mM, and 80 mM, respectively (Table 2). Selected putative mutants can be found in S1 Table.

Number of locules per pod (ln) differed significantly among EMS treatments ($P < 0.001$). The control recorded a mean of 17.00 ± 0.60, with a range of 12 to 24 locules. Higher EMS concentrations resulted in a significant reduction in ln: 14.93 ± 0.48 locules at 40 mM and 11.41 ± 0.39 locules at 80 mM. Pairwise comparisons showed that only the 80 mM treatment differed significantly ($P < 0.001$). From both the control and all other treatments, Phenotypic variability increased with EMS dose, as reflected by a rise in the coefficient of variation from 17.5% at 20 mM to 25.8% at 80 mM. Based on deviations from the control range, the frequency of putative mutants for ln reached 20.0% at 40 mM and 50.0% at 80 mM (Table 2). Selected putative mutants can be found in S1 Table.

Table 2: Mean, variability, mutant frequency, and pairwise significance of yield-related and yield traits in the M_1 generation of cowpea under different EMS concentrations

Trait	Trt	N	Mean±se	%CV	Min	Max	Freq	P	T-values of significant pairwise comparisons			
									0mM	20mM	40mM	80mM
npp	0mM	31	3.84±0.17	25.30	2.00	5.00		ns				
	20mM	48	4.02±0.13	21.50	2.00	5.00						
	40mM	55	3.82±0.13	25.70	2.00	5.00						
	80mM	56	3.70±0.16	31.80	2.00	5.00	-	-				
nptm	0mM	31	24.68±1.31	29.50	14.00	44.00		ns	-	-	-	-
	20mM	48	25.46±1.07	29.10	10.00	45.00						
	40mM	55	23.25±0.78	25.00	11.00	44.00						
	80mM	56	23.87±0.97	30.40	10.00	46.00		-	-			
nsp	0mM	31	9.84±0.63	35.60	5.00	15.00	-	ns		-	-	-
	20mM	48	9.92±0.46	32.30	5.00	15.00						
	40mM	55	10.44±0.39	27.80	5.00	15.00						
	80mM	56	9.80±0.46	35.10	5.00	15.00	-	-	-			
nspt	0mM	31	233.10±14.38	34.30	100.00	406.00		ns		-	-	-
	20mM	48	246.29±13.29	37.40	100.00	450.00						
	40mM	55	238.20±10.34	32.20	100.00	435.00						
	80mM	56	226.36±12.17	40.20	100.00	450.00						
pdl	0mM	31	14.65±0.31$_b$	11.80	11.55	17.86	-	*	-	-	-	2.66**
	20mM	48	14.34±0.25$_{ab}$	12.30	10.50	19.88	10.42					
	40mM	55	13.83±0.26$_{ab}$	14.10	6.95	16.75	9.10					
	80mM	56	13.52±0.28$_a$	15.20	8.45	17.50	21.43		2.66**			
pdw	0mM	31	0.85±0.01	9.70	0.70	1.03	-	ns	-	-	-	-
	20mM	48	0.89±0.02	16.50	0.61	1.23						
	40mM	55	0.90±0.03	25.60	0.52	1.28						
	80mM	56	0.83±0.02	18.00	0.60	1.13						
ln	0mM	31	17.00±0.60$_c$	19.70	12.00	24.00	-	***	-	-	2.87*	7.77***
	20mM	48	17.06±0.43$_c$	17.50	12.00	23.00					3.67**	8.94***
	40mM	55	14.93±0.48$_b$	23.90	8.00	21.00	20.00		2.87*	3.67**		5.77***
	80mM	56	11.41±0.39$_a$	25.80	7.00	23.00	50.00		7.77***	8.94***	5.77***	
psa	0mM	31	14.27±1.93	75.30	0.00	35.71	-	ns	-	-	-	-
	20mM	48	18.60±1.68	62.70	0.00	35.71						
	40mM	55	19.35±1.66	63.80	0.00	35.71						
	80mM	56	15.43±1.53	74.20	0.00	35.71						
sdl	0mM	31	7.44±0.24$_a$	18.10	5.68	11.59	-	***	-	-5.19***	-9.41***	-10.10***
	20mM	48	8.77±0.25$_b$	19.60	6.10	11.65	0.42		-5.19***		-4.64***	-5.40***
	40mM	55	9.79±0.07$_c$	4.90	8.10	11.05			-9.41***	-4.64***		
	80mM	56	9.95±0.09$_c$	6.60	7.95	11.60	1.79		-10.1***	-5.4***		
sdw	0mM	31	4.78±0.10$_a$	11.90	3.94	6.08	-	***	-	-3.44**	-3.52**	-4.63***
	20mM	48	5.14±0.09$_b$	11.80	4.05	6.44	0.42		-3.44**			
	40mM	55	5.14±0.04$_b$	5.90	4.58	5.69			-3.52**			
	80mM	56	5.25±0.05b	7.00	4.28	6.11	1.79		-4.63***			

Note. Means sharing the same superscript letter within a trait are not significantly different according to Tukey's HSD test ($P \leq 0.05$). Trt= Treatment, Mean ± SE = Mean plus or minus standard error, %CV= Percentage coefficient of variation, Min= minimum, Max= maximum, Freq= Percentage frequency of putative mutants; P=Probability value. Traits: npp = number of pods per peduncle, nptm = number of pods per plant at maturity, nspt = number of seeds per plant, pdl = pod length, pdw = pod width, ln = number of locules, nsp = number of seeds per pod, psa = percent seed abortion, sdl = seed length, sdw = seed width, sdt = seed thickness, swgt = seed weight, yld = yield per plant, *=significance at 5% level, **=significance at 1% level, ***=significance at 0.1% level

Table 2: Mean, variability, mutant frequency, and pairwise significance of yield-related and yield traits in the M_1 generation of cowpea under different EMS concentrations (cont'd)

Trait	Trt	N	Mean±se	%CV	Min	Max	Freq	P	T-values of significant pairwise comparisons			
									0mM	20mM	40mM	80mM
sdt	0mM	31	$4.09 \pm 0.07_a$	9.80	3.29	4.92		***		-2.82*		
	20mM	48	$4.35 \pm 0.08_b$	12.20	3.31	5.19	12.50		-2.82*		4.92***	3.35**
	40mM	55	$3.95 \pm 0.04_a$	8.50	3.43	5.59	1.82			4.92***		
	80mM	56	$4.08 \pm 0.05_a$	8.90	3.41	4.94	1.79			3.35**		
swg	0mM	31	$21.03 \pm 0.34_a$	9.10	17.82	24.87		***		-6.91***	-6.18***	-2.92*
	20mM	48	$24.86 \pm 0.34_c$	9.40	17.81	29.99	54.17		-6.91***			4.77***
	40mM	55	$24.37 \pm 0.35_c$	10.70	17.70	28.39	49.10		-6.18***			3.87***
	80mM	56	$22.60 \pm 0.33_b$	11.00	17.35	28.68	16.07		-2.92*	4.77***	3.87***	
yld	0mM	31	$48.79 \pm 3.01_a$	34.40	20.61	85.68		*		-2.61*		
	20mM	48	$61.19 \pm 3.34_b$	37.80	18.70	125.44	16.67		-2.61*			
	40mM	55	$58.02 \pm 2.59_{ab}$	33.10	19.97	105.43	12.72					
	80mM	56	$51.16 \pm 2.87_{ab}$	42.00	18.45	111.85	10.71					

Note. Means sharing the same superscript letter within a trait are not significantly different according to Tukey's HSD test ($P \leq 0.05$). Trt= Treatment, Mean ± SE = Mean plus or minus standard error, %CV= Percentage coefficient of variation, Min= minimum, Max= maximum, Freq= Percentage frequency of putative mutants; P=Probability value. Traits: npp = number of pods per peduncle, nptm = number of pods per plant at maturity, nspt = number of seeds per plant, pdl = pod length, pdw = pod width, ln = number of locules, nsp = number of seeds per pod, psa = percent seed abortion, sdl = seed length, sdw = seed width, sdt = seed thickness, swgt = seed weight, yld = yield per plant, *=significance at 5% level, **=significance at 1% level, ***=significance at 0.1% level

Seed length (sdl) varied significantly across EMS treatments ($P < 0.001$). The control recorded the lowest mean seed length, with a value of 7.44 ± 0.24 mm, with a range of 5.68 to 11.59 mm and a CV of 18.1%. EMS treatment groups resulted in a progressive increase in seed length, with means of 8.77 ± 0.25 mm at 20 mM, 9.79 ± 0.07 mm at 40 mM, and 9.95 ± 0.09 mm at 80 mM. Pairwise comparisons showed that all EMS treatments differed significantly from the control ($P < 0.001$), while the 40 mM and 80 mM treatments did not differ from each other. Based on deviations from the control range, mutant frequencies were low for seed length recorded at 0.42% for 20 mM and 1.79% for 80 mM (Table 2). Selected putative mutants can be found in S1 Table.

Seed width (sdw) was also significantly ($P < 0.001$) affected by EMS treatment. The control population had a mean width of 4.78 ± 0.10 mm, ranging from 3.94 to 6.08 mm, with a CV of 11.9%. All EMS treatments differed significantly from the control ($P < 0.001$). Variability declined at higher EMS doses, with CV values as low as 5.9% at 40 mM.

Frequencies of putative mutants increased with EMS concentration, reaching 0.42% at 20 mM and 1.79% at 80 mM (Table 2). Selected putative mutants can be found in S1 Table.

Seed thickness (sdt) differed significantly among treatments (P < 0.001). The control recorded a mean thickness of 4.09 ± 0.07 mm with values ranging from 3.29 to 4.92 mm. Thickness increased significantly (P=0.027) at 20 mM with a mean of 4.36 ± 0.08 mm. Pairwise comparisons indicated significant differences between 20 mM and both 40 mM (P < 0.001) and 80 mM (P = 0.005). Mutant frequencies for seed thickness were highest at 20 mM (12.5%) but remained low at 40 mM (1.82%) and 80 mM (1.79%) (Table 2). Selected putative mutants can be found in S1 Table.

100-Seed weight (swg) showed highly significant variation among EMS treatments (P < 0.001). The control recorded a mean of 21.03 ± 0.34 g, with a range of 17.82 to 24.87 g. EMS treatment increased swg, with the highest means observed at 20 mM (24.86 ± 0.34 g) and 40 mM (24.37 ± 0.35 g), both of which differed significantly from the control (P < 0.001). The 80 mM treatment weighed 22.60 ± 0.33 g and showed an intermediate response, differing considerably from both the control (P = 0.02) and lower EMS doses (P < 0.001). Frequencies of putative mutants were highest at 20 mM (54.17%), followed by 40 mM (49.1%) and 80 mM (16.07%) (Table 2). Selected putative mutants can be found in S1 Table.

Yield per plant (yld) differed significantly among treatments (P = 0.018). The control population recorded the lowest mean yield of 48.79 ± 3.01 g, with a range of 20.61 to 85.68 g. Yield increased significantly (P = 0.047) at 20 mM, with a mean of 61.19 ± 3.34 g, which differed from the control (P = 0.047). The 40 mM (58.02 ± 2.59 g) and 80 mM (51.16 ± 2.87 g) treatments, which exhibited intermediate yields, did not differ significantly from either the control or the 20 mM treatment. Phenotypic variability was high across treatments, with a CV of 33.10–42%, and mutant frequencies declined with increasing EMS concentration, from

16.67% at 20 mM to 10.71% at 80 mM (Table 2). Selected putative mutants can be found in S1 Table.

Multivariate Analysis of Phenological and Yield-Related Traits

PCA Biplot of phenological, yield-related and yield traits

Variance Explained by Principal Components. Principal component analysis (PCA) of the traits and genotypes of plant observations across EMS treatments yielded 8 principal components with eigenvalues < 1 (Table). The first four principal components accounted for a total variability greater than 50.60% in all treatments. The first two principal components (Dim1 and Dim2) together explained 32.8% of the total phenotypic variation, with PC1 accounting for 17.7% and PC2 accounting for 15.1% (, Table) with an eigenvalues of 3.18 and 2.71 respectively.

Fig 2: PCA biplot of cowpea genotypes and traits based on PC1 and PC2. (a)Genotypes treated with 0-, 20-, 40- and 80-mM EMS are labelled A01–A31, B01–B48, C01–C55 and D01–D56, respectively. (b) Vectors indicate trait loadings, and points represent genotype distribution. (c) dtg= days to germination, dff = days to first flower, d50f = days to 50% flowering, dfh = days to first harvest, d50mp = days to 50% mature pods, npp = number of pods per peduncle, nptm = number of pods per plant at maturity, nspt = number of seeds per plant, pdl = pod length, pdw = pod width, ln = number of locules, nsp = number of seeds per pod, psa = percent seed abortion, sdl = seed length, sdw = seed width, sdt = seed thickness, swgt = seed weight, yld = yield per plant

Distribution of EMS Treatment Groups in the PCA Biplot. The PCA biplot () plotted genotype scores on PC1 (Dim1, 17.7%) against PC2 (Dim2, 15.1%). Control genotypes (A01–A31, 0 mM) clustered predominantly on the negative side of PC1, spanning approximately −5.0 to −0.5, with limited spread along PC2. Genotypes from the 20 mM treatment (B01–B48) occupied a broader area, with partial overlap with the control cluster and moderate displacement toward positive PC1 values. Genotypes from the 40 mM treatment (C01–C55) were more widely dispersed across both axes, with a concentration in the central to positive PC1 region. Genotypes from the 80 mM treatment (D01–D56) showed the widest distribution

across the biplot, with the greatest displacement toward positive PC1 and the greatest scatter along PC2 of any treatment group.

Biplot Trait Vector Orientations. Vectors for *dff*, *d50f*, *dfh*, and *d50mp* projected horizontally toward the positive PC1 axis. Vectors for sdl and sdw were oriented toward the lower-right quadrant (positive PC1, negative PC2). The *swg* vector projected toward the positive region of both PC1 and PC2. Vectors for *yld*, *nspt*, and *nsp* were directed upward toward positive PC2, with *yld* and nspt nearly collinear. The *nptm* vector was oriented downward toward negative PC2. Vectors for *pdl* and *ln* projected toward the upper-central region of the biplot. The angle between the *sdl*/*sdw* vectors and the *yld*/*nspt*/*nsp* vectors was approximately perpendicular ().

Identification and Selection of Putative Mutants from the Biplot. Based on positional alignment with trait vectors of agronomic interest, three classes of putative mutants were identified in . Eleven genotypes positioned in the upper biplot region with positive PC2 scores, in alignment with the *yld*, *nsp*, *nspt*, and *swg* vectors, were classified as high-yielding putative mutants: **B33, B28, B22, B31, D56, D45, D25, C5, C41, B26, and B21**. Eleven genotypes positioned along the negative PC2 axis in alignment with the *sdl* and *sdw* vectors, showing above-average seed dimensions with reduced seed number and yield, were classified as large-seed putative mutants: **D23, D7, D34, D47, C37, C39, C40, C42, B18, B11** and **B8**.

Two genotypes positioned on the extreme negative PC1 side, in the direction opposite to the phenological delay vectors, were classified as early-phenology putative mutants: **B19** and **C2**. Four genotypes **B9, B7, C46 and D20** were positioned at the extreme positive PC1 end with weak alignment to yield vectors and were classified as severely delayed and low performing. These genotypes were not selected for advancement but were retained as extreme phenotypic reference individuals.

Cluster analysis of phenological and yield-related traits

Hierarchical cluster analysis of 18 phenological and yield-related traits across 190 M_1 cowpea genotypes using Ward's linkage method resolved six distinct clusters (Fig 3, S1 Table). The six clusters contained 64, 24, 31, 20, 40, and 9 genotypes, respectively.

Fig 3: Dendrogram of hierarchical cluster analysis of M_1 cowpea genotypes based on quantitative traits using Ward's linkage method.
(a)Genotypes treated with 0-, 20-, 40-, and 80-mM EMS are labelled A, B, C, and D, respectively. (b) Six clusters are delineated by colour: Cluster 1 (green, n = 64); Cluster 2 (brown, n = 24); Cluster 3 (grey, n = 31); Cluster 4 (forest green, n = 20); Cluster 5 (blue, n = 40); Cluster 6 (red, n = 9).

Cluster 1 (n = 64). Cluster 1 contained predominantly EMS-treated genotypes, with 35.42%, 49.10%, and 33.93% of the 20 mM, 40 mM, and 80 mM treatment groups represented (Fig 3, S1 Table). Only one control genotype (A01) was assigned to this cluster, accounting for 3.22% of the 0 mM population. Days to 50% flowering ranged from 53 to 80 days across cluster members, with the longest delays recorded in C41 and C43. Days to 50% pod maturity ranged from 69 to 109 days, with the most extended values recorded in D43 and C08. Seed weight ranged from 18.82 g (D04) to 28.68 g (D56). Yield per plant ranged from 31.45 g (C37) to 111.85 g (D56). Putative mutants selected from this cluster included D56 (111.85 g), D45 (*106.09 g*), D25 (*102.66 g*), B31 (*107.22 g*), B28 (*103.50 g*), B22 (*88.96 g*), C41 (*90.96 g*) and C5 (*105.43 g*).

Cluster 2 (n = 24). Cluster 2 was dominated by control genotypes, which accounted for 61.29% of the 0 mM population (Fig 3, S1 Table). Representation from EMS treatments was low: 2.08% (20 mM), 3.64% (40 mM), and 3.57% (80 mM). Genotypes in this cluster had the earliest germination, flowering, and maturity values across the full population, with yield levels comparable to the control mean and low within-cluster variability. EMS-treated genotypes assigned to this cluster included C02, with days to first harvest of 38 days, and C14, with days to first harvest of 39 days. Both were identified as early-phenology putative mutants.

Cluster 3 (n = 31). Cluster 3 contained genotypes primarily from the high-dose EMS treatments, with 28.57% of the 80 mM population represented (Fig 3, S1 Table). Genotypes in this cluster showed delayed phenological development, reduced locule number, and low yield per plant. Representative genotypes included B7, B9, C46, and D20, all of which showed negative deviation from the control range across multiple yield traits. These genotypes were not selected for advancement.

Cluster 4 (n = 20). Cluster 4 included genotypes from all three EMS treatment groups: 10.42% of 20 mM, 9.09% of 40 mM, and 12.50% of 80 mM (Fig 3, S1 Table). Genotypes in this cluster combined relatively early days to first flower and days to 50% flowering with extended days to first harvest and days to 50% mature pods. Seed weight and yield per plant were elevated relative to the control in several members. The highest-yielding genotype in this cluster was B33 (125.44 g), followed by B19 (93.59 g). Both substantially exceeded the control population range for yield per plant while retaining comparatively early flowering phenology — B19 at 36 days to first flower and B33 at 37 days.

Cluster 5 (n = 40). Cluster 5 comprised exclusively EMS-treated genotypes, with 33.33% of the 20 mM population, 22.27% of the 40 mM population, and 16.07% of the 80 mM population assigned to this cluster (Fig 3, S1 Table). No control genotypes were present. Genotypes in this cluster showed moderately delayed phenological development and high seed weight but reduced locule number per pod, which was associated with lower yield per plant relative to the control mean.

Cluster 6 (n = 9). Cluster 6 was the smallest cluster, containing 12.50% of the 20 mM population and 5.36% of the 80 mM population (Fig 3, S1Table). No control or 40 mM genotypes were present. Genotypes in this cluster showed moderately delayed phenological development, large seed dimensions, and high seed weight. Seed number per pod was relatively low, but total yield per plant was elevated, driven by seed weight. Putative mutants selected

from this cluster included B07 (yld = 67.18 g, swg = 29.99 g) and D36 (yld = 74.69 g, swg = 28.29 g), both of which exceeded the control mean for seed weight.

Discussion

Percentage germination and survival of M1 plants

The EMS concentrations of 20, 40, and 80 mM and the fixed 6 h exposure duration were selected to provide a graded mutagenic treatment range while maintaining sufficient plant survival for evaluation, consistent with previous studies showing that EMS response depends on both concentration and exposure time [13,30,31]. In the present study, LD_{50} was not achieved, as seed germination and seedling survival increased with increasing EMS concentration. Although most EMS mutagenesis studies report a decline in germination and survival with increasing dose [16,32-34], several studies have documented non-linear or hormetic responses, where low to moderate EMS doses stimulate germination and early growth before inhibitory effects appear at higher concentrations. In legumes, responses similar to the present study have been reported. For instance, in cowpea relatives, [35] observed in faba bean (*Vicia faba* L.) that six varieties exhibited higher germination and survival at 0.05% EMS compared to the control, followed by a decline at higher concentrations. This hormesis-like response mirrors the increasing germination trend observed in the present study. Similarly, in soybean (*Glycine max* L.), [36] reported that EMS treatments at 0.1–0.2% improved germination and early seedling growth relative to untreated seeds, with inhibitory effects only becoming evident at higher doses. In cucumber (*Cucumis sativus* L.), [37] demonstrated that post-treatment handling influenced germination outcomes, with a sodium thiosulfate rinse yielding higher germination (84.4%) than a water rinse (80.0%) at 0.5% EMS, indicating that detoxification and recovery processes can enhance germination performance. Likewise, in tef (*Eragrostis tef*), [38] reported that a treatment combination of 2.50% EMS for 4 h resulted in

significantly higher germination (77.1%) compared to lower doses, emphasizing the importance of genotype × dose × exposure-time interactions. In barley (*Hordeum vulgare* L.), [39] also noted a non-monotonic response, where low EMS exposure improved germination relative to some treatments, although optimal survival occurred at intermediate doses. Collectively, these studies support the interpretation that the increasing germination and survival rates observed with rising EMS concentration in the present study may reflect a hormetic or priming-like effect, rather than a deviation from expected mutagenic behavior. This reinforces the role of genotype-specific responses and treatment conditions in shaping EMS-induced physiological outcomes.

Our results differs from the findings of [16, 32-34], [32] reported an apparent dose-dependent reduction in germination and survival in the M_1 generation of cowpea varieties *Pusakomal* and *V-240 (Rambha)* following EMS treatments at 0.1–0.3%, with control germination exceeding 95% and higher doses approaching the LD_{50} threshold. Similarly, [14] observed a strong negative linear relationship between EMS concentration and germination in cowpea and identified an optimal LD_{50} dose at 0.4% EMS. The contrast observed in this current study was that germination and survival were lowest in the control. It increased with EMS concentration, indicating that LD_{50} expression is highly dependent on genotype, EMS dose range, and exposure duration. Unlike previous studies that employed percentage-based EMS concentrations and different treatment protocols, the present study used millimolar EMS concentrations with a fixed 6-h exposure, which may have altered the physiological response. In addition, the relatively low germination and survival recorded in the control suggest baseline establishment constraints that may have obscured EMS-induced lethality. These findings indicate that the absence of an LD_{50} endpoint in the present study does not imply insufficient mutagenic effect. Instead, it underscores the importance of genotype × dose × exposure-time

interactions in EMS mutagenesis. [40,41]. Such responses have been linked to priming-like effects, where mild stress exposure improves germination performance [40].

Contrast analysis of traits in the M_1 generation

Effects of EMS Mutagenesis on Phenological traits

Effect of EMS on Days to Germination.The present study revealed that EMS treatment significantly delayed days to germination (*dtg*) in the M_1 generation of cowpea (*Vigna unguiculata* (L.) Walp). The control seeds germinated earliest, while all EMS-treated groups exhibited delayed germination. This pattern of EMS-induced germination delay is consistent with the well-established understanding that chemical mutagens impose physiological stress during seed imbibition, activating DNA repair mechanisms that temporarily arrest the cell cycle until lesions are repaired [31]. The alkylating activity of EMS, which produces O^6-ethylguanine and other base modifications, necessitates repair via base excision repair pathways before the resumption of cell division in the embryonic axis [21]. This repair process delays radicle emergence, resulting in a measurable increase in days to germination.

Comparable delays in days to germination have been documented in other crops. Similarly, research on marigold (*Tagetes* spp.) demonstrated that higher EMS concentrations and longer exposure times reduced germination rate and delayed progress, although the effect was largely inhibitory rather than showing a peak delay at a lower dose [42]. The high frequency of putative mutants for *dtg* at 20 mM (50.0%) compared to 40 mM (29.1%) and 80 mM (30.4%) aligns with the observation that lower EMS doses often yield a higher proportion of viable mutants because they cause less overall lethality and permit the expression of a wider range of phenotypic variation [23,24]. In cowpea, [13] similarly found that lower EMS doses produced useful variability while higher doses reduced survival and limited the number of recoverable mutants. The reduction in mutant frequency at higher concentrations in our study may reflect the increased lethality and physiological damage that suppress the recovery of germination-related mutants.

Effects of EMS on Days to First Flower. In the present study, EMS treatment at all three concentrations caused a highly significant delay in *dff* relative to the control. Pairwise comparisons confirmed that all EMS treatments differed significantly from the control, while no significant differences were detected among the three EMS concentrations themselves. This pattern suggests a threshold effect, where even the lowest dose was sufficient to disrupt normal floral transition, with no further meaningful delay introduced at higher concentrations. The delayed onset of flowering observed here aligns with findings reported in other EMS mutagenesis studies on cowpea. [26] documented early and late maturity as among the viable mutant classes induced by EMS in *Vigna unguiculata*, noting that EMS can modify important components of plant cells and affect the morphology, anatomy, biochemistry, and physiology of plants differentially depending on concentration level. Similarly, [14] reported effects of EMS, DES, and SA on quantitative traits of cowpea in the M_1 generation, providing early evidence that flowering-related traits are susceptible to chemical mutagenesis in this species.

The mechanism underlying EMS-induced delays in flowering time is most likely attributable to point mutations disrupting key genes involved in floral transition pathways. [31] noted that M_1 plants subjected to EMS mutagenesis have shown variation in flower organs and delayed flowering, consistent with the disruption of genes regulating the floral transition, and that such phenotypic changes in flower-related traits may reflect the broad mutagenic spectrum of EMS across multiple developmental pathways. In cowpea specifically, flowering time is known to be a quantitatively inherited and environmentally sensitive trait. [43] demonstrated that time to flowering in cowpea is strongly influenced by photoperiod, with photoperiod-sensitive genotypes under long natural days exhibiting considerable delays in floral development. EMS-induced mutations affecting photoperiod perception genes or floral integrators such as *GIGANTEA*, *CONSTANS*, or *FLOWERING LOCUS T* homologs could

therefore plausibly extend the time to first flower, even at relatively low mutagen concentrations.

The mutation frequencies observed for days to first flower as 51.17%, 47.27%, and 41.07% for 20, 40, and 80 mM, respectively showed a declining trend with increasing EMS concentration. This is consistent with the well-documented inverse relationship between mutagen dose and the proportion of viable, phenotypically expressed mutations, where higher doses tend to induce increasingly lethal or phenotypically cryptic mutations rather than productive, viable phenotypic variants. [44] reported a similar pattern in cowpea var. Arka Garima, where a linear decline in morphological mutation frequency was observed with increasing EMS concentration, suggesting that higher doses induce proportionally more deleterious rather than viable mutations.

Effects of EMS on days to 50% flowering. In this study, we observed that the pairwise comparisons confirmed that all mutagenized populations differed significantly from the control but not from each other, reinforcing the threshold dose interpretation. The coefficient of variation was relatively low across all groups (8.9–11%), indicating that although the mean was shifted by EMS treatment, population-level variation in the timing of 50% flowering remained moderate. Notably, the range of days to 50% flowering was broader in the EMS treatments (50–80 days) than in the control (48–68 days), reflecting the expanded phenotypic diversity generated by mutagenesis. These findings are in agreement with results reported from induced mutagenesis experiments in related *Vigna* species and cowpea populations. For instance, [45] evaluated M_5 Tswana cowpea mutant lines across two seasons reported significant variation in days to 50% flowering, with the Tswana control consistently flowering earlier than many of the mutant lines and noted that delayed flowering in mutagenized populations may be attributed to genetic changes created by the mutation that affect normal flowering mechanisms.

In EMS-mutagenized cowpea (cv. *Asontem*) evaluated in M_2 populations, [46] similarly reported wide distribution of days to flowering among mutagenized individuals compared to the wild type, demonstrating that EMS is effective in generating significant phenotypic variation in flowering-related quantitative traits in cowpea. The parallel shifts in both days to first flower and days to 50% flowering across all EMS treatments suggested that EMS disrupted the floral initiation process rather than the speed of floral development once initiated. If the mutagen had primarily affected floral development after initiation, one would expect a disproportionate shift in days to 50% flowering without a corresponding shift in days to first flower. The approximately equal delays of 6–7 days in both traits across all treatments instead point to a disruption of the upstream signaling or perception pathways governing the transition from vegetative to reproductive growth. Research on contrasting cowpea genotypes by [47] established that delays in flowering are primarily driven by delays in the first appearance of floral buds rather than in bud development itself, supporting the interpretation that the mutagenic effects observed here act at or upstream of bud initiation.

The observations in this study were made in the M_1 generation, where induced mutations were expected to be heterozygous, and chimerism may confound phenotypic expression. [46] noted that during the M_1 generation, only dominant or co-dominant mutations are easily detected by phenotypic observation, as the functional mutations induced by EMS through point mutation have a greater chance of being dominant or co-dominant. The delayed flowering documented in this study likely reflected the expression of dominant or semi-dominant mutation effects on floral pathway genes, while recessive mutations which may represent early or altered flowering phenotypes of greater breeding value will only become apparent upon selfing and selection in the M_2 and subsequent generations.

The delay in flowering under stronger mutagenesis is a common phenomenon, often attributed to physiological stress or to disruption of genes controlling flowering time [48-

51].[49] did not directly measure flowering time, but their M_1 mutants showed overall physiological delays, and other researchers have explicitly noted flowering extensions. For example, [52] working with mung bean reported that higher EMS levels prolonged the time to flowering, likely due to mutational interference with developmental pathways. It is also recorded by [53,54], [55] in black gram (*Vigna radiata*). In cowpea, [56] observed an earlier onset of flowering and a decrease in days to 50% flowering in an EMS-treated yardlong bean (*Vigna sesquipedalis*) population, accompanied by reduced maturity duration. Such wide variation in flowering time due to EMS has also been reported by [15], who found that their M_2 cowpea population showed continuous distribution for days to flowering far beyond the range of the wild type. This implies that EMS created both later- and earlier-flowering individuals. Notably, [15] identified dozens of mutants that flowered significantly ($P<0.05$) earlier than the parent variety, indicating the emergence of new earliness traits. These results suggest that specific mutations can accelerate flowering, potentially by knocking out repressors of flowering even as overall vigor is reduced. In our mutants, while the predominant trend at high EMS dosages was delayed flowering and maturity, a few early-blooming mutants were observed at lower EMS treatments, indicating increased phenological variation. Our results confirm that EMS mutagenesis increased the variance in flowering time. While early flowering is advantageous for drought avoidance and multi-cropping systems, our evaluation of the EMS population did not yield any early-flowering mutants; instead, such occurrences were observed in the control group. This outcome aligns with results previously reported in mung bean and other pulse crops.

Effect of EMS on Days to First Harvest. The present study showed EMS treatment at all three concentrations produced a highly significant delay in days to first harvest (*dfh*) compared to the untreated control. No significant differences were detected among the three EMS concentrations themselves, again pointing to a threshold response wherein even the

lowest concentration was sufficient to substantially delay the onset of first harvest, with no additive effect at higher doses. The delays in days to first harvest observed in the present study are a logical downstream consequence of the earlier-documented delays in days to first flower and days to 50% flowering and are consistent with the general expectation that any disruption of floral transition will propagate through the reproductive developmental timeline. In cowpea, pod development and seed fill are closely coupled to the timing of anthesis, and a delay of in flowering onset would be expected to produce a delay of similar harvestable pod maturation as observed here. [25], applied EMS at 10 mM for 6 hours to cowpea varieties *IT84E-124* and *Vita 7*, reported variants in maturity date among the spectrum of mutations recovered in the M_2 generation, demonstrating that EMS can disturb the genetic determinants of cowpea maturation timing even at concentrations below those used in the present study.

In this study, the mutation frequencies recorded as 18.75%, 9.10%, and 12.50% for 20, 40, and 80 mM respectively, do not follow a simple linear dose-response pattern, with the lowest EMS concentration producing the highest proportion of phenotypic variants for this trait. This is consistent with the broader mutagenesis literature indicating that higher doses of chemical mutagens tend to produce proportionally more lethal or cryptic mutations rather than phenotypically viable and selectable variants. The dose of EMS concentration and exposure duration both affect the viability of induced mutations, and the frequency of viable mutations declined at concentrations that approach or exceed the LD_{50}, as higher doses shift the mutation spectrum increasingly toward deleterious rather than functional alleles [57]. A similar study by [58] who evaluated EMS effects on quantitative traits in garden pea (*Pisum sativum* L.) in the M_1 generation found that variation due to EMS treatment was significant for days to maturity, with increasing EMS concentration generally associated with delayed maturity, and noted that such delays in maturity were consistent across concentrations in a manner similar to flowering time responses. The strong similarity between *dfh* results across 20–80 mM in the present

cowpea study, and the absence of a further dose-dependent increment beyond the initial shift from the control, mirrors this threshold pattern observed in garden pea. The relatively higher mutation frequency at 20 mM for days to first harvest therefore suggests that sub-lethal doses may be more productive for generating harvestable maturity variants in cowpea, though this interpretation must be qualified by the relatively small population sizes in this M_1 study.

Effects of EMS on Days to 50% Mature Pods.Coefficients of variation were low and comparable across all treatments (9.1–10.5%), indicating that EMS did not increase population-level variance in pod maturation timing, but instead produced a coherent directional shift in mean maturity across the mutagenized populations. Mutation frequencies of 39.58%, 25.45%, and 23.21% for 20, 40, and 80 mM respectively again followed the declining trend with increasing EMS dose observed for *dfh*, reinforcing the interpretation that the 20 mM dose generated the highest proportion of viable phenotypic variants for maturation-related traits. The delay in *d50mp* closely parallelled the delay in *dfh* across all treatments, with the absolute number of days between first harvest and 50% pod maturity remaining stable across the control (approximately 30 days) and EMS treatments (approximately 28–30 days). This similarity suggests that EMS disrupted the upstream developmental program governing the onset of reproductive maturation, most plausibly through mutations affecting the floral transition pathway, rather than the rate of pod filling or seed development intrinsically. A study on EMS mutagenesis of fodder cowpea (var. *Aiswarya*) by [59] reported that days to maturity was among the quantitative traits showing significant variation in the mutated population in M_2, with EMS-induced mutants covering a broad range of maturity classes including both early and late maturing individuals relative to the control[44] .The observation of delayed maturity in the present M_1 study without evidence of any early-maturing variants at the population mean level is consistent with the expectation that dominant or co-dominant mutations, which are the only classes detectable at the phenotypic level in M_1, are more likely to disrupt rather than accelerate

developmental programs. Stable phenotypic variation in both directions, including earlier maturity, is expected to be more fully expressed from M_2 onwards, as the first non-chimeric generation in which homozygous recessive mutations can be phenotypically detected [13,60].

The agronomic implications of delayed maturity in EMS-mutagenized cowpea M_1 populations deserve consideration. While late maturity is undesirable from a production standpoint in environments with short growing seasons, it may represent a useful source of variation for adaptation to longer-season cropping systems or for intercropping arrangements where extended vegetative and reproductive periods are advantageous. The importance of early maturing mutations for short-season environments has long been recognized in cowpea improvement programs, with early maturing selections serving as candidates for areas with limited rainfall and as relay crops in rice paddies. In contrast, the late-maturing phenotype observed in the current M_1 population may harbor individuals carrying alleles that, in heterozygous combination, mask early maturing recessive mutations, suggesting that early maturity mutants with direct breeding utility may emerge from the M_2 onwards upon selfing and screening [61].

The results for *dfh* and *d50mp* demonstrated that EMS mutagenesis at concentrations of 20–80 mM induces a coherent and significant delay of 9–11 days in both harvest maturity and pod maturation in cowpea under M_1 conditions. This delay is may be attributed to the dominant expression of EMS-induced mutations disrupting the genetic flow of floral transition and reproductive development, consistent with findings in cowpea and other legumes. The absence of dose-dependent differentiation among EMS treatments suggested a threshold effect for maturity traits, while declining mutation frequencies at higher doses indicate that 20 mM may represent the most productive concentration for generating viable phenotypic variants in maturity-related traits. Evaluation of M_2 and subsequent generations will be essential to

characterize the full spectrum of maturity variation induced, including the recovery of potentially valuable early-maturing mutant classes.

Effects of EMS Mutagenesis on Yield-related and Yield traits

Non-Significant Traits: Number of Pods per Peduncle, Number of Pods per Plant at Maturity, Number of Seeds per Pod, Number of Seeds per Plant, Pod Width, and Percent Seed Abortion. EMS treatment at all three concentrations (20, 40, and 80 mM) produced no statistically significant differences from the control in number of pods per peduncle (*npp*), number of pods per plant at maturity (*nptm*), number of seeds per pod (*nsp*), number of seeds per plant (*nspt*), pod width (*pdw*), or percent seed abortion (*psa*). The lack of significant and notable mutants in the M_1 generation is likely due to these traits being highly polygenic and developmentally buffered, which restricts observable changes [4,62-64]. These findings suggest that a single generation of EMS exposure at these doses were insufficient to consistently disrupt the genetic architecture underlying these yield components in the M_1 generation. However, significant mutants were identified for these traits in M_2 populations, where recessive mutations are revealed, and chimerism is resolved [15,65-Click or tap here to enter text.67]. Moreover, higher EMS doses often reduce pod and seed numbers due to partial sterility or lower pollen viability, as seen in legumes [4,34,53].

[13] reported wide distribution of yield-related traits including number of pods per plant, number of seeds per pod, and number of seeds per plant primarily in the M_2 population rather than M_1, noting that the frequency of viable mutations depends strongly on treatment conditions and that functional mutations induced by EMS through point mutation have a greater chance of being dominant or co-dominant with only those classes easily detected by phenotypic observation in the M_1 generation[13]. The absence of significant mean shifts in these traits in the current M_1 study therefore may reflects the recessive nature of many yield-component mutations rather than an absence of induced mutations.

[31] reported highly significant mutants with improved performance for number of pods per plant, number of seeds per pod, number of locules per pod, and pod length were identified in EMS-mutagenized cowpea populations, but their detection required evaluation of the M_2 generation, reinforcing the need to advance mutagenized material to subsequent generations for meaningful yield screening. Similarly, [25] reported increases in number of seeds per pod, peduncles per plant, and 100-seed weight in advanced mutant lines of cowpea varieties ***IT84E-124*** and ***Vita 7*** following mutagenesis with EMS at 10 mM and NaN_3, though these improvements were characterized from M_2 onwards rather than M_1 plants [59] . The high coefficients of variation recorded in this study for *nsp*, *nptm*, *nspt*, and *psa* across all treatments, including the control, indicated that these traits are inherently variable and may require larger sample sizes or later generation evaluation to detect treatment effects. The notably high CV for *psa* reflected the expected wide individual variation in seed set across a mutagenized population. The higher performance in other yield traits exhibited by mutants may be attributed to pleiotropy, whereby mutation in one trait affects others [61], which further complicates detection of significant mean shifts in individual yield components when evaluation is confined to M_1.

Effects of EMS on Pod Length. Pod length (*pdl*) showed a significant overall treatment effect, with a dose-dependent reduction across EMS concentrations. Pairwise comparison confirmed a significant difference only between the control and the 80 mM treatment, this suggest that the reduction in pod length became statistically detectable only at the highest EMS concentration. The highest mutation frequency for *pdl* of 21.43% was observed in 80-mM treatment, which indicated that the 80 mM dose induced the greatest proportion of detectable variants, consistent with a dose-response relationship in this trait. The reduction in pod length with increasing EMS concentration is consistent with findings in the broader mutagenesis literature for legumes. Studies on fodder cowpea in the M_1 generation reported reductions in

several quantitative characters including pod length with increasing EMS concentrations, with reductions in quantitative traits observed to be colinear with increased mutagen dose irrespective of the mutagen used [59]. This pattern has been attributed to the mutagenic disruption of genes governing cell elongation and pod development [26]. In legumes, the activity of the inflorescence meristem determines the number and size of flowers and pods at each node through the growth of the floral meristem, and mutations affecting this developmental pathway can alter pod size and architecture [26]. EMS-induced point mutations at loci controlling pod wall cell division or expansion would therefore be expected to reduce pod length in a dose-dependent manner [68], as observed here.

In contrast, some advanced-generation mutagenesis studies have reported enhanced pod length in selected mutant lines. [69],obtained aphid-resistant cowpea mutants from gamma-irradiated populations with increased pod length among their improved attributes, while the same study noted that lines with significant increases in yield parameters were selected from M_2 screening [25]. The apparent contradiction between M_1 reductions and M_2 improvements in pod length likely reflects the phenomenon of heterozygous M_1 plants masking recessive gain-of-function alleles, which only become homozygously expressed and selectable in subsequent generations [70]

Effects of EMS on Number of Locules per Pod. Number of locules per pod (*ln*) showed the most pronounced and dose-dependent response to EMS treatment among all traits evaluated. All pairwise comparisons among treatments were statistically significant, and the mutation frequency increased with concentration, which reached 50% at 80 mM. The CV also increased with EMS dose, which reflected the expanding phenotypic diversity generated at higher concentrations. The strong dose-dependent reduction in locule number is particularly notable from a breeding standpoint, as locule number is a primary determinant of the number of seeds per pod and therefore of pod yield potential. In legumes, the identity and activity of

the inflorescence meristem governs flower and pod architecture, and mutations in genes regulating meristematic activity can directly alter the number of seeds per pod and overall pod structure[68].

The significant reduction in *ln* at 40 and 80 mM, without a corresponding significant reduction in *nsp*, suggests that while EMS disrupted the developmental program responsible for locule number, the individual seed development process within remaining locules were largely maintained. This dissociation between locule number and seeds per pod may indicate that some locule positions failed to initiate rather than that initiated locules failed to complete seed set. [31] noted that highly significant mutants with improved performance for number of locules per pod were identified in EMS-mutagenized cowpea in M_2 populations, demonstrating that both directional increases and decreases in locule number are achievable through EMS mutagenesis depending on the specific mutation event. The predominance of locule-reducing variants in the current M_1 population is consistent with the expectation that dominant loss-of-function mutations more likely to be expressed in heterozygous M_1 plants tend to disrupt rather than enhance developmental programs. Alleles conferring additional locules, which would likely require gain-of-function or additive effects, may be present in the population but will only become phenotypically apparent upon homozygosity in M_2.

Effects of EMS on Seed Dimensions — Length, Width, and Thickness.The three seed dimension traits; seed length (*sdl*), seed width (*sdw*) and seed thickness (*sdt*) collectively exhibited highly significant responses to EMS treatment, but with notably different dose-response patterns across the three concentrations. ***Seed length*** increased with EMS treatment, as all comparisons between the control and treated groups were highly significant. However, there was no significant difference between the 40- and 80-mM treatments, indicating a plateau above 40 mM. The coefficient of variation decreased with increasing EMS dose, showing reduced variability. This suggested that EMS caused a consistent shift toward longer seeds

rather than increasing variation. This pattern was consistent with the expression of mutations that promoted seed elongation, leading to a uniform directional effect across the population.

Seed width followed a consistent but shallower pattern of EMS-induced increase. All three EMS concentrations were significantly wider than the control, though none differed significantly from one another indicating again a threshold effect where even the lowest dose captured the full directional shift achievable for this trait. The absence of dose-dependent differentiation among EMS treatments for seed width, combined with the significant and consistent departure from the control, points to a single effective threshold below 20 mM at which EMS disrupted or altered the genetic regulation of seed width, with no further phenotypic increment at higher concentrations.

Seed thickness, by contrast, produced the most complex and non-linear response of the three dimensions. The 20 mM treatment was the only concentration to produce a significant increase over the control, while both the 40 mM and 80 mM treatments were statistically indistinguishable from the control but significantly lower than the 20 mM treatment. The mutation frequencies for *sdt* , 12.50%, 1.82%, and 1.79% at 20, 40, and 80 mM respectively showed by far the steepest drop between the lowest and intermediate doses of any trait in this study, suggesting that the genetic targets for seed thickness are highly sensitive to EMS dose and that concentrations above 20 mM largely neutralized or reversed the thickness-enhancing mutations, possibly by simultaneously inducing compensatory or antagonistic mutations in related seed developmental pathways.

The divergence in response among the three seed dimensions is agronomically and biologically meaningful. Seed length responded with a strong, progressive, dose-dependent increase that plateaued above 40 mM; seed width responded with a uniform, threshold-type increase independent of dose; and seed thickness showed a transient, concentration-restricted increase that was not maintained above 20 mM. This dissociation among the three axes of seed

growth implies that seed length, width, and thickness in cowpea are at least partially under distinct genetic control, and that EMS mutagenesis differentially targeted these control systems across the tested concentration range. Consistent with this interpretation, QTL mapping studies in cowpea have identified distinct genomic regions controlling pod and seed dimensions, including pod length and 100-seed weight, suggesting that seed size traits can be genetically decoupled and are governed by a combination of shared and independent loci [71]. The increases in seed length and width documented here are consistent with findings from induced mutagenesis studies in cowpea and related legumes. [25] reported significant increases in seed weight among EMS-mutagenized cowpea lines, with traits including plant height, seed weight, and protein content increasing in mutant populations compared to untreated controls. The outcomes were attributed to EMS altering structural gene loci governing these quantitative traits[25]. [72] similarly documented substantial increases in 100-seed weight in gamma-irradiated cowpea mutants, with 100-seed weight rising from *16.95 g* in the control to *22.80 g* in selected M_2 mutant lines, and observed that seed size traits displayed high heritability, indicating a strong genetic basis amenable to selection.

The pattern across all three seed dimensions, thus progressive elongation in length, uniform widening, and transient thickening points toward a net increase in overall seed volume in EMS-treated populations, predominantly driven by enhanced length and, to a lesser degree, width. This three-dimensional enlargement is likely to translate into increased individual seed weight, which is consistent with the significant increases in seed weight (*swg*) documented in this study. [13]attributed high performance in multiple yield traits exhibited by EMS-mutagenized cowpea mutants partly to pleiotropy, whereby mutation in one gene affects several related developmental traits. This mechanism may explain the coordinated, if not perfectly similar, increases across seed length, width, weight, and, at 20 mM, thickness observed in the current M_1 population. As with all M_1 data, the observed means represented

population-level responses from heterozygous individuals and should be interpreted cautiously as indicators of induced genetic variation rather than fixed trait values. Stable phenotypic expression of EMS-induced seed dimension changes is expected from M_2 onwards, as the first non-chimeric generation in which homozygous alleles become expressed and individually selectable [13]. Selection in M_2 for individuals combining large seed length, adequate width, and maintained thickness particularly among progeny of 20 and 40 mM-treated plants where mutation frequencies for seed dimension traits were highest, offers the most promising route to isolating fixed large-seeded mutant lines of breeding value.

Effects of EMS on Seed Weight. Seed weight (*swg*) showed the most pronounced and consistently positive response to EMS treatment among all yield traits evaluated. All three EMS concentrations produced significantly heavier seeds than the control. Pairwise comparisons confirmed that 20 and 40 mM did not differ significantly from each other, but both were significantly heavier than 80 mM, which in turn remained significantly heavier than the control. This pattern describes a partial dose-dependent attenuation: maximum seed weight enhancement occurred at 20–40 mM, with a decline though still significant improvement over the control at 80 mM. Mutation frequencies of 54.17%, 49.10%, and 16.07% at 20, 40, and 80 mM respectively confirm the well-documented inverse relationship between EMS dose and the proportion of viable, selectable phenotypic variants, as higher concentrations shift the mutation spectrum increasingly toward lethal or cryptic alleles rather than productive, phenotypically expressed changes[13]. The significant increase in seed weight across all EMS treatments is consistent with findings in cowpea and related grain legumes subjected to chemical and physical mutagenesis. [25], applying EMS at 10 mM for 6 hours to cowpea varieties ***IT84E-124*** and ***Vita 7***, reported significant increases in seed weight and other yield parameters among selected mutant lines, attributing improvements in polygenic characters like yield to changes in simply inherited traits or mutations at structural loci. [72] similarly reported significant

increments in 100-seed weight in gamma-irradiated cowpea mutants at M_2 and M_3 generations, observing increases from *16.95 g* in the control to *22.80 g* in selected mutant lines, a proportional gain broadly comparable to that recorded in the 20- and 40-mM treatments of the current study.

The comparable increase in seed weight with the previously documented increases in seed length and seed width supports an interpretation of coordinated enhancement of seed size across multiple dimensions at low-to-intermediate EMS concentrations. [59], who used EMS to develop improved fodder cowpea populations, reported significant variation in seed yield per plant in M_2 populations, noting that mutations altering seed morphology traits tend to produce coordinated changes in associated yield components, consistent with pleiotropic gene action or tight genetic linkage among seed size and weight loci. The reduction in seed weight at 80 mM, while still above the control, is likely attributable to higher mutagenic load at this concentration interfering with genes governing seed filling efficiency, endosperm development, or photosynthate partitioning effects that would be expected to partially offset the seed-enlarging mutations still being expressed from this dose.

Effects of EMS on Yield Per Plant. Yield per plant (*yld*) showed a significant overall treatment effect, but with a more limited and dose-restricted pattern than seed weight. Only the 20 mM treatment produced a statistically significant increase in yield relative to the control (*61.19 ± 3.34 g* vs. *48.79 ± 3.01 g*), representing an approximate 25% yield advantage. The 40 mM (*58.02 ± 2.59 g*) and 80 mM (*51.16 ± 2.87 g*) treatments produced numerically higher yields than the control but were not significantly different from it, nor from the 20 mM treatment. The high coefficients of variation across all groups (33.1–42.0%) reflect the inherently variable nature of yield per plant in M_1 populations, a consequence of the heterogeneous mutational load across individuals and indicate that a portion of the variance attributable to EMS effects may have been obscured by this within-treatment variability.

Mutation frequencies of 16.67%, 12.72%, and 10.71% for 20, 40, and 80 mM respectively were low and declined modestly with increasing EMS concentration, consistent with the modest treatment effect sizes recorded for this trait.

The significant yield increase at 20 mM is agronomically important, even within the context of M_1 limitations. [59] documented significant variation in seed yield per plant in EMS-mutagenized cowpea M_2 populations and concluded that EMS mutagenesis is effective in inducing yield-relevant genetic changes, and that seed yield and days to maturity were among the quantitative traits showing significant variation in the mutated population. The pattern observed in the current study which showed significant yield improvement at the lowest EMS concentration but non-significant trends at higher doses is consistent with the principle that sub-lethal mutagen doses are most productive for generating viable, yield-enhancing alleles. [13] attributed high yield performance in EMS-mutagenized cowpea mutants to EMS altering genes responsible for this trait, and additionally to pleiotropy, whereby mutation in one trait affects others a mechanism that is particularly relevant here, as the yield increases at 20 mM likely reflect the combined downstream effects of increased seed weight, enhanced seed dimensions, and maintained seed number documented in the same treatment group.

The overall yield results, viewed alongside the seed weight and seed dimension data, present an internally consistent picture: 20 mM EMS induced the broadest suite of positive seed quality and yield mutations in this M_1 population, while 40 and 80 mM produced proportionally more neutral or disruptive mutations that dampened the yield response. As with all M_1 observations, however, these means represent population averages across heterozygous individuals and cannot directly predict the yield potential of specific mutant lines. The identification and selection of superior individual mutant plants rather than evaluation of population means will be the critical next step, as demonstrated by, [72] who identified twelve putative cowpea mutants combining early maturity with significantly higher seed yields than

the parental control through individual plant selection from M_2 and M_3 generations. Advancing the current population to M_2 and screening individual lines for the convergence of high seed weight, appropriate seed dimensions, and elevated yield per plant represents the most productive strategy for exploiting the genetic variation induced by EMS in this study.

In our research, high-yielding mutants, such as B09, typically produce larger seeds with higher seed weight, a favorable combination for yield improvement. Similarly, [25] and [72] reported cowpea mutants with enlarged seeds and improved yield performance, consistent with the superior lines identified in the present study. Yield stimulation following mutagenic seed treatments has also been documented in other legumes. Increased yield or yield components after mutagen exposure have been reported in pigeonpea [73], mung bean [74], and urdbean treated with gamma rays, EMS, and sodium azide [75]. These studies collectively support the notion that induced mutagenesis can unlock favorable allelic variation controlling yield traits.

In contrast, many mutants, particularly from the highest EMS dose (80 mM), exhibited reductions in yield and its components, including pod length, number of locules, seed size, and seed weight. Such adverse effects of high mutagen doses are well documented. [76] In okra and [77] in petunia, it was reported that mutagenesis frequently produces lines with reduced reproductive output. In pigeon pea (*Cajanus cajan*), [78] observed that EMS doses beyond the optimal range led to sharp declines in pollen fertility and seed yield. At the same time, [79] reported positive yield shifts at lower mutagen doses and negative shifts at higher doses. Our results closely mirror this dose-dependent pattern. Yield depression at elevated doses of irradiation or chemical mutagens has been widely reported. Similar declines were observed by [52] in *Gloriosa superba*, [53] in *Vigna radiata* under EMS and DES treatments, [80] in *Vigna mungo*, and [81] in *Vigna radiata*. These findings reflect the accumulation of deleterious mutations at higher mutagen doses, which can impair physiological and reproductive processes. Conversely, [4] reported stimulation of seed weight in pigeon pea under combined mutagenic

treatments, suggesting that moderate doses may enhance specific yield components. Similar observations were reported by [82], [83], and [26] across various pulse crops, reinforcing the dose- and trait-dependent nature of mutagenic responses.

One elite mutant (B33) from the 20 mM EMS treatment in the current study produced approximately 25% higher seed yield than the control, accompanied by a modest increase in 100-seed weight. This indicates that EMS successfully generated yield-enhancing alleles. Similarly, [59] reported EMS-derived cowpea lines with significantly increased pod numbers confirm the potential of chemical mutagenesis to improve yield. It is also noteworthy that the frequency of desirable mutants was low, consistent with previous reports. [15] estimated that only 0.1–1% of mutants exhibited significantly improved yield-related and yield traits. Similarly, although a few promising mutants were identified in the present study, the majority performed at or below the level of the control. This underscores the need to screen large mutant populations to capture rare beneficial events.

Multivariate Analysis: PCA Biplot and Cluster Grouping

PCA Biplot of phenological, yield-related and yield traits

Variance structure and principal component interpretation. Principal component analysis was applied to the full set of 18 phenological and yield-related traits across 190 M_1 cowpea genotypes to characterize the multivariate structure of EMS-induced variation. The first eight principal components collectively explained 76.2% of the total phenotypic variance, with PC1 and PC2 accounting for 17.7% and 15.1% respectively a combined 32.8% of variance captured in two dimensions. The relatively distributed variance structure across eight components reflects the complexity of the trait matrix, the phenotypically heterogeneous nature of M_1 mutagenized populations, and the partial independence of phenological, reproductive, and seed morphology trait clusters. [85], evaluated cowpea breeding lines using multivariate analysis, similarly found that the first three principal components explained 76% of total

variation and noted that groupings in biplots conformed well with dendrogram results, demonstrating that PCA effectively captures the major sources of agronomic differentiation in cowpea populations [7]. PC1 carried strong positive loadings from seed length (*sdl* = 0.73), seed width (*sdw* = 0.70), days to first harvest (*dfh* = 0.68), days to first flower (*dff* = 0.61), days to 50% mature pods (*d50mp* = 0.59), days to 50% flowering (d50f = 0.57), and seed weight (*swg* = 0.53). This axis therefore captures a composite gradient of phenological delay and seed dimensional enlargement, two trait groups that co-vary positively across EMS treatments in this present study, this reflects the tendency of mutagenized lines to simultaneously delay reproductive development and produce morphologically larger seeds. PC2 was dominated by highly positive loadings from number of seeds per plant (*nspt* = 0.96), yield per plant (*yld* = 0.95), and number of seeds per pod (*nsp* = 0.75), alongside negative loadings from number of pods per plant at maturity (nptm = −0.66), days to first flower (*dff* = −0.64), and days to 50% flowering (*d50f* = −0.68). PC2 thus represented a yield productivity axis, where high positive scores reflect superior reproductive output in terms of seed number and total yield, decoupled from phenological timing. Together, PC1 and PC2 defined a two-dimensional space within which the fundamental EMS-induced trade-off between phenological disruption, seed enlargement, and reproductive yield can be visualized at the individual genotype level.

Treatment group separation in the biplot. The biplot distribution of genotype scores revealed a clear dose-dependent spatial separation among EMS treatment groups. Control genotypes (A01–A31, 0 mM) formed a compact cluster on the extreme negative side of PC1, consistent with their early phenology, uniform developmental timing, and moderate to high yield performance relative to the mutagenized population. This clustering confirmed the low phenotypic variance expected of untreated plants within a uniform cultivar background and validates the internal coherence of the control group. [86], applied PCA to M_5 Tswana cowpea mutant lines and similarly found that the control variety formed a separate cluster that diverged

from all mutant lines in multivariate space, confirming that induced mutagenesis effectively altered the genetic makeup of the treated population in ways detectable by multi-trait analysis.

Genotypes from the 20 mM treatment (B01–B48) showed partial overlap with the control group but were moderately displaced toward the positive PC1 region and exhibited greater within-group dispersion, particularly along PC2. This intermediate positioning reflects the milder and more heterogeneous mutational load associated with the lowest EMS concentration were consistent with individual-level variation in mutation induction and expression at sub-lethal doses. Genotypes from the 40 mM (C01–C55) and 80 mM (D01–D56) treatments were progressively displaced toward the positive PC1 axis and displayed substantially greater scatter across both PC1 and PC2, indicating dose-dependent increases in phenological delay alongside widened variation in reproductive output. Multivariate PCA of cowpea germplasm has consistently shown that high phenotypic diversity, reflected by wider scatter in biplot space, creates greater opportunity for selection of superior genotypes, as variation among traits provides the basis for discriminating among individuals for multiple desirable characteristics simultaneously [87].

Trait vector interpretation and EMS-induced trade-offs. The present study showed the orientation of trait vectors in the biplot provided insight into the relationships among traits and the nature of EMS-induced phenotypic trade-offs. Vectors for the four phenological traits (*dff, d50f, dfh, d50mp*) were oriented horizontally towards the positive PC1 axis, this confirmed their strong association with the right-side positioning of high-dose EMS genotypes. The *sdl* and *sdw* vectors were directed toward the lower-right quadrant, positive PC1 and negative PC2 which indicated that seed dimensional enlargement was associated with phenological delay but inversely related to yield productivity. In contrast, the *yld*, *nspt*, and *nsp* vectors pointed strongly toward the upper region of the biplot (positive PC2), which formed an approximately perpendicular angle to the *sdl*/*sdw* vectors. This near-orthogonal relationship between yield

and seed size vectors quantitatively confirms the resource allocation trade-off implied by the individual trait analyses: EMS-induced genotypes that developed larger seeds tended to produce fewer seeds per pod and per plant, resulting in reduced total yield. PCA-driven multivariate analyses in other legume systems have similarly identified trade-offs between plant architectural and yield productivity traits loading on different principal components, where biplots revealed that selection based on single traits may inadvertently compromise performance in correlated traits and reinforced the need for multi-trait selection indices[88].

The *swg* vector was oriented toward the positive PC1 and moderately positive PC2 region which indicated that heavier seeds were associated with both phenological delay and, to a degree, maintained or enhanced yield performance, a positioning distinct from *sdl* and *sdw*, which loaded more strongly on the negative PC2 side. This suggested that seed weight integration partly captured the influence of both seed size and seed number components, reflecting its composite nature as a product of seed dimensions and density.

The *pdl* and *ln* vectors were directed upward left, with *pdl* loading strongly on PC2 and *ln* on both PC1 and PC2. These vectors were oriented broadly toward the central and upper biplot region, indicating that genotypes with longer pods and higher locule numbers were not confined to a single EMS treatment group and could be found across the treatment spectrum consistent with the individual trait analyses which showed that pod length was significantly reduced only at 80 mM while locule number showed a progressive dose-dependent reduction.

Identification of putative mutant classes. The biplot facilitated the graphical identification of three distinct classes of agronomically relevant putative mutants, each positioned in a different region of the two-dimensional space. High-yielding putative mutants were identified from genotypes clustered in the upper region of the biplot with positive PC2 scores, in close alignment with the *yld*, *nsp*, *nspt*, and *swg* vectors. These included B33, B28, B22, B31, D56, D45, D25, C5, C41, B26, and B21. The presence of genotypes from both 20

mM (B-series) and 40–80 mM (C and D series) treatments among this group indicated that yield-enhancing mutations were not exclusively associated with the lowest EMS dose, though most B-series genotypes in this cluster is consistent with the higher mutation frequencies and significant mean yield increase documented at 20 mM in the individual trait analyses. [86] similarly identified mutant lines with improved grain yield through PCA biplot analysis and noted that the distribution of mutagenized populations in biplot space, this reflected the interaction of genotype and mutagen dose that generated significant quantitative trait variation that could be exploited through targeted selection [86].

Large-seed putative mutants were identified from genotypes located along the negative PC2 axis in the direction of the *sdl* and *sdw* vectors, including D23, D7, D34, D47, C37, C39, C40, C42, B18, B11, and B8. These genotypes, from the 40- and 80-mM treatments, produced larger seeds but fewer in number, which resulted in lower overall yield. This pattern suggested that resources were redirected to produce fewer but larger seeds. Although this was not ideal for total yield, these genotypes were useful for breeding large-seeded varieties preferred by consumers and for studying the genetic basis of seed development in cowpea.

Early-phenology putative mutants were identified from the extreme negative side of PC1, opposite to the late-flowering and maturity vectors, and included B19 and C2 notably from the 20- and 40-mM treatments respectively. These genotypes showed accelerated phenological development relative to the control group, suggesting the presence of rare dominant or semi-dominant early-flowering mutations within the mutagenized population. Their identification is agronomically significant: previous studies in cowpea mutagenesis have confirmed that EMS causes mutations in genes responsible for maturity-related traits, and early maturing mutants are particularly valuable for short-season production systems and as relay crops in rice paddies where a compressed growing season is critical[13].

Genotypes exhibiting extreme positive PC1 scores with weak alignment to yield vectors including B9, B7, C46, and D20 were classified as severely delayed and low performing. These lines, positioned in the far-right region of the biplot with little positive PC2 displacement, represented the most mutagenized damaged individuals in the population, they likely carried multiple loss-of-function mutations with pleiotropic negative effects on reproductive development and yield. While unsuitable for direct advancement, they were retained as extreme phenotypes for genetic and physiological characterization, and as potential donors in targeted crosses where specific mutant alleles may segregate independently from the broader phenotypic impairment.

Broader utility of PCA in EMS mutation studies. In this study we reduced the dimensionality of the 18-trait dataset into two interpretable axes, the biplot enabled simultaneous visualization of dose-treatment group separation, inter-trait relationships, and individual genotype positioning relative to trait vectors all within a single analytical framework. This multi-trait perspective is particularly important in M_1 studies where high within-treatment variance, heterozygosity, and chimerism create complex phenotypic distributions that are difficult to interpret through single-trait analyses alone. PCA biplot analysis of cowpea populations has been shown to effectively identify which traits account for the most variation among genotypes, allowing breeders to focus selection on the most discriminated trait combinations and to identify individuals that deviate meaningfully from the population center in the direction of target trait improvement. [89]. Also, [90], used multi-trait analysis to identify superior M_2 individuals and reported that mutants with extremely high values in key traits, such as seeds per plant, could be distinguished from the population distribution. In the present study, the biplot confirmed that yield-related traits (*npp*, *nsp* and *swg*) and seed dimension traits loaded most strongly on the first two principal components, this provided the clearest discrimination among EMS-treated lines, while traits with minimal biplot

contribution such as *psa* and *pdw* had limited power to differentiate among genotypes at the multivariate level.

The clear spatial separation between high- and low-yielding mutants, and between early- and late-phenology groups, demonstrated that the genetic variation induced by EMS at 20–80 mM was sufficient to generate agronomically meaningful divergence, detectable at the population level in the M_1 generation. Confirmation of the most promising putative mutants particularly the high-yielding group (B33, B28, B22, B31, D56, D45, D25, C5, C41, B26, B21) and the early-phenology group (B19, C2) through M_2 progeny testing and subsequent generation advancement remains essential before any breeding value can be assigned.

Hierarchical Cluster Analysis of EMS-Mutagenized Cowpea

Hierarchical cluster analysis using Ward's linkage method effectively partitioned the 190 M_1 cowpea genotypes into six phenotypically distinct clusters, each characterized by a unique combination of phenological, yield-related and yield trait profiles. The resolution of six clusters from a single mutagenized population is consistent with reports from other induced mutagenesis studies in cowpea and related legumes, which have commonly observed between three and six discrete phenotypic groupings following mutagen treatment, reflecting the breadth of the genetic variation induced [86,91]. The clustering outcome in the present study demonstrated that EMS mutagenesis at 20–80 mM generated sufficient phenotypic divergence across 18 traits to resolve multiple distinct genotypic classes within a single cultivar background, a result that underscores the utility of Ward's method for structuring genotype selection in M_1 mutation populations.

Cluster 1: high-yielding EMS-derived genotypes. Cluster 1, characterized by prolonged phenological development and superior yield performance, contained disproportionately high proportions of the 20- and 40-mM treatment groups, with 35.42% and 49.10% of these populations respectively assigned to this cluster. Cluster 1 contained the

highest proportion of agronomically desirable genotypes, and most putative mutants selected for advancement. The combination of elevated seed weight (up to 28.68 g in D56), extended phenological development, and high yield per plant (up to 111.85 g in D56) in this cluster is consistent with reports of yield-enhancing mutations in EMS-mutagenized cowpea populations. [13] selected the top twenty mutants with high yield performance in terms of number of seeds per plant from their M_2 EMS-mutagenized cowpea population, they reported that traits including higher number of pods per plant, increased protein content and high seed weights had been obtained through EMS mutagenesis in earlier cowpea studies. The genotypes identified in Cluster 1 in the present study include D56, D45, D25, B31, B28, B22, C41, and C5 exhibited yield values significantly exceeding the control range and represented the primary candidates for advancement to M_2 and subsequent generation evaluation.

Cluster 2: control-dominated baseline cluster. Cluster 2, which represented the population's baseline phenotypic profile and was dominated by control genotypes (61.29% of the 0 mM population), received minimal representation from EMS-treated lines. The concentration of control genotypes in Cluster 2 confirmed that the untreated population occupied a phenotypically distinct region of the multivariate trait space, separated from the majority of EMS-treated genotypes. The small number of EMS-treated lines assigned to this cluster notably C02 and C14, both from the 40 mM treatment, with early days to first harvest values of 38 and 39 days respectively, represented a class of putative early-phenology mutants that maintained control-like performance across most traits while showing accelerated crop development. [91], who developed new cowpea mutant genotypes through gamma irradiation, also reported that cluster groupings could be attributed to the origin of genotypes and the degree of genetic alteration induced, with control varieties consistently forming separate clusters that diverged from mutagenized lines in multivariate space [92]. This confirming the effectiveness of mutagenesis in altering the genetic makeup of the treated population [92].

Cluster 3: severely impaired high-dose genotypes. In contrast, Cluster 3 was characterized by delayed phenology, reduced locule number, and low yield, and it was mainly composed of genotypes from the 80 mM treatment, with 28.57% of that group assigned to this cluster. This cluster showed the most severely impaired phenotypes in the population. Representative genotypes B7, B9, C46, and D20 showed marked negative deviations from the control range across multiple traits simultaneously and were classified as low-value mutants unsuitable for direct advancement. This cluster is analogous to the poor-performance groups consistently reported in high-dose mutagenesis studies. [93] noted that higher mutagen doses tend to produce populations with reduced fertility, delayed maturity, and inferior agronomic performance, and recommended against their use in breeding programmes where the objective is to recover viable, productive mutant lines[94]. While the genotypes in Cluster 3 were not selected for advancement, they were retained as phenotypic reference material, as the extreme mutations they carry may be informative for gene function studies or for identifying linkage between yield-impaired and yield-neutral alleles in subsequent molecular characterization.

Cluster 4: early-flowering high-yield mutants.Cluster 4 contained a distinctive combination of early days to first flower and days to 50% flowering with extended days to first harvest and elevated yield, a trait profile not represented in any other cluster. The presence of B19 (*yld* = 93.59 g, *dff* = 36 days) and B33 (*yld* = 125.44 g, *dff* = 37 days) in this cluster is particularly noteworthy: these genotypes achieved the highest individual yields in the entire population while retaining comparatively early flowering phenology, a combination of traits of direct relevance to short-season cowpea production. EMS-induced early maturing mutants have been specifically highlighted as valuable in cowpea breeding because of their ability to escape drought or tolerate insect damage due to their short reproductive phase, with early mutants with days to flowering of 38–41 days having been isolated from M_2 EMS-mutagenized cowpea populations in earlier studies [13]. The presence of such genotypes in Cluster 4 across

the 20- and 40-mM treatments, but not exclusively at the lowest dose, reinforces the value of evaluating mutant lines individually rather than making selection decisions based solely on treatment concentration.

Cluster 5: moderate performance with seed weight improvement. Cluster 5, composed exclusively of EMS-treated genotypes from all three treatment groups, was characterized by moderately delayed phenological traits, elevated seed weight, and reduced locule number, resulting in yield performance generally below the Cluster 1 and Cluster 4 groups but above the impaired lines of Cluster 3. The absence of control genotypes from this cluster confirms that the trait profile it represented, elevated seed weight combined with moderately delayed development and was not expressed within the untreated population and is therefore a product of mutagenesis. Genotypes in this cluster may still harbor breeding value as parents for crosses targeting seed quality improvement, even if their direct yield performance does not match the elite lines identified in Clusters 1 and 4.

Cluster 6: large-seeded, seed-weight-driven yield. Cluster 6 was the smallest and most phenotypically distinctive group in the analysis, contained only 9 genotypes from the 20- and 80-mM treatments. These genotypes combined large seed dimensions and high seed weight with moderate phenological delay and relatively low seed number per pod, producing elevated yield per plant primarily driven by individual seed weight rather than seed number. Selected genotypes from this cluster, including B07 (*swg* = 29.99 g, *yld* = 67.18 g) and D36 (*swg* = 28.29 g, *yld* = 74.69 g), represented a distinct agronomic type that may be particularly valuable for breeding programmes targeted at large-seeded, consumer-preferred market classes. [95], developed high-yielding cowpea mutant lines through gamma irradiation and sodium azide, similarly used dendrogram analysis to separate control and mutant plants into distinct clusters, confirmed that considerable genetic variability was generated and that genetically diverged

mutants could be employed directly as mutant varieties or as parents in crossbreeding programmes for advanced improvement and trait fixation[95].

Broader utility of cluster analysis in EMS mutation studies. This dose-stratified clustering pattern is consistent with the well-established principle that lower and intermediate mutagen doses preferentially generate viable, selectable phenotypic variants, while higher doses shift the mutation spectrum toward lethal or disruptive alleles. [93] confirmed this pattern in cowpea, they demonstrated that lower and intermediate mutagen doses induced higher frequencies of morphological mutations with the least biological damage and recommended these doses for future breeding programmes to obtain a high spectrum of desirable mutants[94]. The dominance of 40 mM-derived genotypes in Cluster 1 and of 80 mM-derived genotypes in Cluster 3 precisely mirrors this dose–performance gradient. However, EMS dose was not a perfect predictor of cluster assignment and individual genotype performance must be considered on its own merits. Some 20 mM genotypes were assigned to lower-performing clusters, while some 80 mM genotypes clustered with better-performing lines. [13] , in their EMS mutagenesis study of cowpea cv. *Asontem*, emphasized that the phenotypic expression of each mutant line is unique and determined by the specific gene(s) altered by the mutagen. [13] noted that the high yield performance of selected mutants may reflect EMS altering genes responsible for traits as well as pleiotropic effects whereby mutations in one trait affect related traits[13]. Results from the present study reinforced this interpretation: multivariate clustering based on actual trait performance provided a more reliable selection criterion than EMS dose alone.

The clustering outcomes confirmed that the elite putative mutants identified across Clusters 1, 4, and 6 shared a multi-trait profile that were not present in the control population, suggesting they carried functional alleles which favored yield, seed size, or early phenology that were induced by EMS treatment. Agronomic evaluation of cowpea mutant lines in

Eswatini demonstrated that cluster analysis is a key non-parametric statistical technique that facilitates the identification of diverse genotypes with contrasting traits for breeding and production, and that genotypes grouped in high-yielding sub-clusters can be directly recommended for production or use as crossing parents in further improvement programmes[92]. The present study reinforces this conclusion, with the elite clusters identified here providing a structured and evidence-based shortlist of M_1 genotypes for advancement to M_2 and evaluation in replicated trials across environments.

Conclusion

This study demonstrates the effectiveness of ethyl methane sulfonate (EMS) in inducing substantial genetic variability in cowpea (*Vigna unguiculata* (L.) Walp), thereby broadening the phenotypic spectrum for key agronomic, yield, and seed traits. Contrast analysis revealed significant treatment effects and highlighted superior mutant lines, while principal component analysis (PCA) identified yield and reproductive traits as the primary drivers of phenotypic variation. These findings were consistently supported by cluster analysis, which grouped mutants according to overall agronomic performance. The convergence of these analytical approaches strengthened the selection of putative mutants, including high-yielding (B33, B31, B28, B22, C41, C05, D25, D45, D56), early-phenology (B19), and large-seeded or prolific podding lines (D34, C39, C40).

As the study was confined to the M_1 generation, further evaluation in the M_2 and M_3 generations is required to confirm trait stability, heritability, and breeding value. Complementary molecular analyses will be essential to explain the genetic basis of the observed variation and support marker-assisted selection. Overall, the selected mutants constitute valuable genetic resources for cowpea improvement and have strong potential to enhance productivity and global food security. Considering the documented genetic variability of cowpea for nutritional traits such as protein and mineral content, the EMS-induced mutants

identified in this study may serve as valuable genetic resources for future breeding programs targeting both yield and nutritional quality improvement .

Acknowledgments

HMK, RANK, IKA, and FO conceived and designed the study. HMK and RANK performed the experiments. HMK analyzed the data and drafted the manuscript. RANK and IKA provided critical revisions. All authors read and approved the final manuscript. We gratefully acknowledge the Department of Plant and Environmental Biology at the University of Ghana, Legon, for supporting this research.

References

Sert H, Ceyhan E. The effects of seed yield and some agricultural characters of different plant density on cowpea (Vigna sinensis (L.) Savi) in Hatay ecological conditions. Selcuk Journal of Agriculture and Food Sciences. 2012;26(1):34–43.

Harmankaya M, Ceyhan E, Çelik AS, Sert H, Kahraman A, Özcan MM. Some chemical properties, mineral content and amino acid composition of cowpeas (Vigna sinensis (L.) Savi). Quality Assurance and Safety of Crops & Foods. 2016;8(1):111–6. doi:10.3920/QAS2014.0487

Boukar O, Massawe F, Muranaka S, Franco J, Maziya-Dixon B, Singh B. Evaluation of cowpea germplasm lines for protein and mineral concentrations in grains. Plant Genetic Resources. 2011;9:515–22.

Singh BD. Plant Breeding: Principles and Methods. 7th ed. New Delhi: Kalyani Publishers; 2006.

dos Santos J V, Valliyodan B, Joshi T, Khan SM, Liu Y, Wang J, et al. Evaluation of genetic variation among Brazilian soybean cultivars through genome resequencing. BMC Genomics. 2016;17(1):110. doi:10.1186/s12864-016-2431-x

Oladosu Y, Rafii MY, Abdullah N, Hussin G, Ramli A, Rahim HA, et al. Principle and application of plant mutagenesis in crop improvement: A review. Biotechnology & Biotechnological Equipment. 2016;30(1):1–16.

Owusu EY, Karikari B, Kusi F, Haruna M, Amoah RA, Attamah P, et al. Genetic variability, heritability, and correlation analysis of maturity and yield traits in Cowpea (Vigna unguiculata (L.) Walp.) in Northern Ghana. Heliyon. 2021;7(9).

Bhat TA, Khan AH, Parveen S. Comparative analysis of meiotic abnormalities induced by gamma rays, EMS, and MMS in Vicia faba L. Journal of Indian Botanical Society. 2005;84:45–58.

Olasupo FO, Ilori CO, Forster BP, Bado S. Mutagenic effects of gamma radiation on eight accessions of cowpea (Vigna unguiculata [L.] Walp.). Am J Plant Sci. 2016;7(2):339–51.

Jou-Nteufa C, Ceyhan E. Genetic analysis of seed yield and some traits in cowpea using diallel analysis. Turkish Journal of Agriculture and Forestry. 2024;48(3):278–93. doi:10.55730/1300-011X.3180

Dalgıç H, Ali A, Karadaş S, Korkmaz A, Ayran Çolak İ, Harmankaya M, et al. Diallel analysis of cowpea (Vigna unguiculata L. Walp) for protein and mineral composition: Genetic variability and heritability. Euphytica. 2025;221:168. doi:10.1007/s10681-025-03618-0

Hoque ME, Morshad MN. Somaclonal variation in potato (Solanum tuberosum L.) using chemical mutagens. The Agriculturists. 2014;12(1):15–25.

Opoku Gyamfi M, Eleblu JSY, Sarfoa LG, Asante IK, Opoku-Agyemang F, Danquah EY. Induced variations of ethyl methane sulfonate mutagenized cowpea (Vigna unguiculata L. Walp) plants. Front Plant Sci. 2022 Aug;13:952247.

Dhanavel D, Girija M. Effect of EMS, DES, and SA on quantitative traits of cowpea (Vigna unguiculata (L.) Walp.) in M1 generation. Crop Research (Hisar). 2009;37(1–3):239–41.

Dhanavel D, Girija M. Effect of EMS on induced morphological mutants in cowpea (Vigna unguiculata (L.) Walp.). Crop Research. 2009;38:27–31.

Roychowdhury R, Tah J. Assessment of chemical mutagenic effects in mutation breeding programme for M1 generation of carnation (Dianthus caryophyllus). Res Plant Biol. 2011;1(4).

Gnankambary TK, Benoit JB, Sawadogo N, Mahamadou S, Tignegre JB, Yonli D, et al. Genetic variability induced by gamma radiation in cowpea (Vigna unguiculata L. Walp) in Burkina Faso. Eur Sci J. 2019;15(15):153–64.

Adamu AK, Aliyu H. Morphological effects of sodium azide on tomato (Lycopersicon esculentum Mill). Science World Journal. 2007;2(4).

Amini M. Ethyl methane sulfonate. In: Wexler P, editor. Encyclopedia of Toxicology. 3rd ed. Academic Press; 2014. p. 522–4.

Kavina J, Ranjith VS, Sathya B. Effect of EMS on chlorophyll mutagen in fenugreek (Trigonella foenum-graecum L.). Journal of Medicinal Plants Studies. 2020;8(2):1–5.

Sega GA. A review of the genetic effects of ethyl methanesulfonate. Mutation Research/Reviews in Genetic Toxicology. 1984;134(2–3):113–42.

Prakash L, Higgins D. Role of DNA repair in ethyl methanesulfonate-induced mutagenesis in Saccharomyces cerevisiae. Carcinogenesis. 1982;3(4):439–44.

Wani MR, Khan S, Kozgar MI. Induced chlorophyll mutations. I. Mutagenic effectiveness and efficiency of EMS, HZ and SA in mungbean. Front Agric China. 2011;5(4):514–8.

Shah D, Kamili AN, Wani AA, Majeed U, Wani ZA, Sajjad N, et al. Promoting the accumulation of scopolamine and hyoscyamine in Hyoscyamus niger L. through EMS based mutagenesis. PLoS One. 2020;15(5):e0231355.

Odeigah PGC, Osanyinpeju AO, Myers GO. Induced mutations in cowpea: Morphological and yield traits. Mutation Breeding Newsletter. 1998;44:10–3.

Gnanamurthy S, Dhanavel D. Effect of EMS on induced morphological mutants and chromosomal variation in cowpea (Vigna unguiculata (L.) Walp.). International Letters of Natural Sciences. 2014;13:33–43.

Shu QY, Forster BP, Nakagawa H, editors. Plant Mutation Breeding and Biotechnology. CABI; 2012.

International Board for Plant Genetic Resources (IBPGR). Descriptors for Cowpea [Internet]. Rome, Italy: IBPGR; 1983. Available from: https://www.bioversityinternational.org/fileadmin/_migrated/uploads/tx_news/Descriptors_for_cowpea_377.pdf

Nawale SR, Apte UB, Jadhav BB. Effect of gamma rays and ethyl methane sulfonate on seed germination and survival of seedlings in cowpea (Vigna unguiculata (L.) Walp.). Journal of Arid Legumes. 2006;3(1):102–5.

Bayhan M, Özkan R, Albayrak Ö, Akinci C, Yildirim M. Effects of ethyl methanesulfonate on growth and yield parameters of wheat and tolerance to imazamox. Bangladesh J Bot. 2024;53(3):487–93. doi:10.3329/bjb.v53i3.76260

Chen L, Hao L, Parry MAJ, Phillips AL, Hu YG. Current trends and insights on EMS mutagenesis application to studies on plant abiotic stress tolerance and development. Int J Mol Sci. 2023;24(3):2945.

Bind D, Dwivedi VK. Effect of mutagenesis on germination, plant survival, and pollen sterility in M1 generation of cowpea [Vigna unguiculata (L.) Walp]. Indian J Agric Res. 2014;48.

Crino P, Lai A, Bonito RD, Veronese P, Saccardo F. Genetic variability in tomato plants regenerated from irradiated cotyledons. In: Jain SM, Brar DS, Ahloowalia BS, editors. Somaclonal Variation and Induced Mutations in Crop Improvement. Dordrecht: Springer Science & Business Media; 2013.

Jain SM, Brar DS, Ahloowalia BS, editors. Somaclonal Variation and Induced Mutations in Crop Improvement. Dordrecht: Springer Science & Business Media; 2013.

Omar MA, El-Sayed HA, Hassan RM. Differential response of faba bean (Vicia faba L.) genotypes to EMS mutagenesis: Germination and early growth traits. Legume Research. 2025;48(1):55–62.

Ramadan SM, Abdel-Hamid AM, El-Metwally MA. Influence of EMS mutagenesis on germination and seedling traits of soybean (Glycine max L.). J Crop Improv. 2025;39(2):210–22.

Chen L, Wang T, Zhao M, Zhang Y. Effect of EMS mutagenesis and post-treatment handling on seed germination in cucumber (Cucumis sativus L.). Mutation Breeding Journal. 2015;45(2):123–30.

Rakau M, Tesfaye K, Bekele E. Optimization of EMS mutagenesis in tef (Eragrostis tef) reveals genotype-dependent germination responses. Plant Genetic Resources. 2025;23(1):34–42.

Sharamo MA, Hussein S, Mohammed A. Evaluation of EMS-induced mutagenesis on germination and survival of barley (Hordeum vulgare L.). Afr J Agric Res. 2021;17(4):567–74.

Rhaman MS, Rauf F, Tania SS, Khatun M. Seed priming before the sprout: Revisiting an established technique for stress-resilient germination. Seeds. 2025;4(3):29.

Ke C, Guan W, Bu S, Li X, Deng Y, Wei Z, et al. Determination of absorption dose in chemical mutagenesis in plants. PLoS One. 2019;14(1):e0210596.

Kumari P. Effect of EMS and colchicine treated seeds on plant growth and flowering in marigold. [Solan, India]: Dr Yashwant Singh Parmar University of Horticulture and Forestry; 2024.

Ishiyaku MF, Singh BB. Inheritance of time to flowering in cowpea (Vigna unguiculata (L.) Walp.). Euphytica. 2005;142:291–300. doi:10.1007/s10681-005-2435-0

Nair R, Mehta AK. Induced mutagenesis in cowpea [Vigna unguiculata (L.) Walp.] var. Arka Garima. Indian J Agric Res. 2014;48(4):247–57. doi:10.5958/0976-058X.2014.00658.1

Ramolekwa K, Pholo-Tait M, Parker T, Malambane G, Ngwako S, Lekgari L. Estimating genetic variability and heritability of morpho-agronomic traits of M_5 cowpea (Vigna

unguiculata (L.) Walp) mutant lines. Int J Mol Sci. 2025;26(15):7543. doi:10.3390/ijms26157543

Adomako-Mensah K, others. Induced variations of ethyl methane sulfonate mutagenized cowpea (Vigna unguiculata L. Walp) plants. Front Plant Sci. 2022;13:952247. doi:10.3389/fpls.2022.952247

Dow el-Madina IM, Hall AE. Flowering of contrasting cowpea (Vigna unguiculata (L.) Walp.) genotypes under different temperatures and photoperiods. Field Crops Res. 1986;14:87–104.

Jain SM. Mutagenesis in crop improvement under the climate change. Rom Biotechnol Lett. 2010;15(2):88–106.

Jain SM, Maluszynski M. In vitro applications in crop improvement: Recent progress. In: Mujib A, Cho M, Predieri S, Banerjee S, editors. Biotechnology for Crop Improvement. New Delhi, India: IBH–Oxford; 2004. p. 169–202.

Kumar A, Rai PK. Induced mutagenesis in mungbean (Vigna radiata L.). In: Singh DP, editor. Mutation Breeding in Pulse Crops. Kalyani Publishers; 2006. p. 85–93.

Kundu SK, Singh DP. Gamma-ray induced variability for quantitative characters in black gram. Madras Agricultural Journal. 1982;69(10):644–8.

Chandra N. Cytological and mutational studies on Gloriosa superba Linn. [Nagpur, India]: Nagpur University; 1988.

Khan S, Wani MR. Comparison of the effect of chemical mutagens on mung bean. Advances in Plant Sciences. 2005;18(2):533–5.

Khan S, Wani MR. Chemomutagenic effect of two alkylating agents on mung bean (Vigna radiata). Bionotes. 2004;6(3):82–3.

Nanda S, Rao GM, Nayak S. Mutagenic effects of EMS on yardlong bean (Vigna sesquipedalis). Indian J Agric Res. 1997;31:602–10.

Allard RW. Principles of Plant Breeding. 2nd ed. New York: John Wiley & Sons; 1999.

Eswaramoorthy V, Kandasamy T, Thiyagarajan K, Chockalingam V, Jegadeesan S, Natesan S, et al. Characterization of terminal flowering cowpea (Vigna unguiculata (L.) Walp.) mutants obtained by induced mutagenesis reveals loss-of-function of phosphatidylethanolamine-binding protein. PLoS One. 2023;18(12):e0295509. doi:10.1371/journal.pone.0295509 PubMed PMID: 38096151.

Vasanthrao T, Sarma D, Barua PK, Sharma KK, Kalita P, Gogoi S. Mutagenic Effects of Ethyl Methane Sulphonate (EMS) on Quantitative Traits in M 1 Generation and the Effectiveness and Efficiency of Different Doses in Garden Pea (Pisum sativum L.). Legume Research: An International Journal. 2025;48(8).

Nair AS, Gayathri G, Thomas UC, Thomas B, Varanya A, Hulsure P. Genetic improvement of fodder cowpea through induced mutagenesis. RANGE MANAGEMENT AND AGROFORESTRY. 2025;46(1):165–9.

Gyamfi MO. Inducing Genetic Variability in Cowpea (Vigna unguiculata, L. Walp Var. Asontem) Using Ethyl Methane Sulfonate [Internet]. 2022. Available from: http://ugspace.ug.edu.gh

Zhang X, Zhai H, Wang Y, Tian X, Zhang Y, Wu H, et al. Functional conservation and diversification of the soybean maturity gene E1 and its homologs in legumes. Sci Rep. 2016;6(1):29548.

Falconer DS, Mackay TFC. Introduction to Quantitative Genetics. 4th ed. Harlow: Longman; 1996.

Grafius JE. Components of yield in oats: A geometrical interpretation. Agron J. 1959;51:515–8.

Gaul H. Mutations in plant breeding. Radiation Botany. 1964;4:155–232.

Maluszynski M, Nichterlein K, Van Zanten L, Ahloowalia BS. Officially released mutant varieties—The FAO/IAEA database. Mutation Breeding Reviews. 2000;12:1–84.

Goyal P, Khan S, Wani MR. EMS-induced genetic variability for yield and yield components in urdbean (Vigna mungo (L.) Hepper). Legume Research. 2021;44:1220–6.

Premsekhar S, Appadurai P. Effect of doses of gamma rays and EMS on induced mutation in pigeonpea. Indian Journal of Agricultural Sciences. 1982;51:381–4.

Weller JL, Ortega R. Genetic control of flowering time in legumes. Front Plant Sci. 2015;6:207. doi:10.3389/fpls.2015.00207 PubMed PMID: 25914700.

Pathak RS. Genetic evaluation of two aphid resistant cowpea mutants in Kenya. In: Proceedings of the IAEA Symposium on Plant Mutation Breeding for Crop Improvement. Vienna, Austria: International Atomic Energy Agency; 1991. p. 463.

Fujii T, Tano S. Mutagenic activities of EMS on somatic (M1) and recessive (M2) mutations in the soybean test system. Environ Exp Bot. 1986;26(2):191–5. doi:https://doi.org/10.1016/0098-8472(86)90015-8

Lo S, Muñoz-Amatriaín M, Boukar O, Herniter I, Cisse N, Guo YN, et al. Identification of QTL controlling domestication-related traits in cowpea (Vigna unguiculata L. Walp). Sci Rep. 2018;8(1):6261. doi:10.1038/s41598-018-24349-4 PubMed PMID: 29674702.

Dorvlo KI, Acquah AS, Armah OJ, Kutufam TJ, Afutu E, Darkwa AA, et al. Induction of genetic variability in cowpea variety Videza for extra earliness and high seed yield using gamma irradiation mutagenesis. Legume Research. 2022;45(9):1074–81. doi:10.18805/LRF-685

Rai A. Effect of Ethyl Methane Sulfonate Mutagen in M1 Generation in Pigeonpea (Cajanus cajan L. Millasp). 2011.

Khan IA. Mutation studies in mung bean (Vigna radiata L. Wilczek): IX. Estimates of genetic variability. Legume Research. 1988;11(2):89–93.

Singh M, Singh VP. Genetic analysis of certain mutant lines of urdbean for yield and quality traits in M4 generation. Indian Journal of Pulses Research. 2001;14(1):60–2.

Jagajanantham N, Dhanavel D, Pavadai P. EMS-induced variability in okra (Abelmoschus esculentus L.). International Journal of Science and Research. 2013;2:148–51.

Berenschot AS, Zucchi MI, Tulmann Neto A, Quecini V. Mutagenesis in ornamentals: Application and perspective. Plant Cell Tissue Organ Cult. 2009;98:161–72.

Premsekar R, Appadurai R. EMS-induced variability in pigeonpea (Cajanus cajan L.). Madras Agricultural Journal. 1982;69:458–66.

Mohanasundaram K, Kalamani A, Ramalingam RS. Induced mutagenesis in cowpea (Vigna unguiculata (L.) Walp.). Legume Research. 1999;22:642–8.

Anis M, Sharma PK, Abbasi N. Gamma rays induced variability in urd bean (Vigna mungo (L.) Hepper) in M1 generation. Journal of the Indian Botanical Society. 1999;78(1–2):111–3.

Khan SB, Siddiqui A, Nadeem M. Variation in quantitative characters of mung bean after seed treatment with DES. Advances in Plant Sciences. 1994;7(1):41–5.

Kansagra UM, Shukla PT. Variability studies in induced mutants of mungbean. Madras Agricultural Journal. 1993;80(4):180–4.

Iepziba SJ, Subramanian M. Variability in M3 and M4 mutants of urdbean. Indian Journal of Pulses Research. 1993;6(2):197.

Patil BN, Guha VY. Growth attributes in induced mutants of chickpea. PKV Research Journal. 1994;15(2):97–101.

Sie EK, Issahaku M, others. Genetic variability, heritability and correlation analysis among maturity and yield traits in cowpea (Vigna unguiculata (L.) Walp) in northern Ghana. Heliyon. 2021;7(9):e07955. doi:10.1016/j.heliyon.2021.e07955

Ramolekwa K, Pholo-Tait M, Parker T, Malambane G, Ngwako S, Lekgari L. Estimating genetic variability and heritability of morpho-agronomic traits of M5 cowpea (Vigna unguiculata (L.) Walp) mutant lines. Int J Mol Sci. 2025;26(15):7543. doi:10.3390/ijms26157543

Chaudhary D, Gore PG, Wankhede DP, Patil DK, Choudhary SB, Rao AM, et al. Morphological variability and genetic diversity assessment of agronomic traits in cowpea (Vigna unguiculata (L.) Walp.) core collection using multivariate analysis. Genet Resour Crop Evol. 2025;72(5):6027–42. doi:10.1007/s10722-024-02299-3

Cao Z, Li J, Lei H, Yan M, Wang Q, Ji R, et al. PCA-Driven Multivariate Trait Integration in Alfalfa Breeding: A Selection Model for High-Yield and Stable Progenies. Plants. 2025;14(18):2906. doi:10.3390/plants14182906 PubMed PMID: 41012057.

Nkomo G V, Sedibe MM, Mofokeng MA. Phenotyping cowpea accessions at the seedling stage for drought tolerance using the pot method. bioRxiv. 2020. doi:10.1101/2020.07.10.196915

Wani AA. Induced mutagenesis for improvement of pulse crops. Plant Breed Rev. 2017;41:155–200.

Diouf M, Diallo S, Badiane FA, Diack O, Diouf D. Development of new cowpea [Vigna unguiculata (L.) Walp] mutant genotypes, analysis of their agromorphological variation, genetic diversity and population structure. Biocell. 2021;45(2):345–62. doi:10.32604/biocell.2021.013706

Mkhonta KAK, Shimelis H, Abady S, Ngidi A. Agronomic Performance of Newly Developed Elite Cowpea Mutant Lines in Eswatini. Agriculture. 2025;15(15). doi:10.3390/agriculture15151631

Raina A, Khan S. Field assessment of yield and its contributing traits in cowpea treated with lower, intermediate, and higher doses of gamma rays and sodium azide. Front Plant Sci. 2023;14:1188077. doi:10.3389/fpls.2023.1188077

Raina A, Laskar RA, Wani MR, Jan BL, Ali S, Khan S. Comparative Mutagenic Effectiveness and Efficiency of Gamma Rays and Sodium Azide in Inducing Chlorophyll and Morphological Mutants of Cowpea. Plants. 2022;11(10):1322. doi:10.3390/plants11101322 PubMed PMID: 35631747.

Raina A, Laskar RA, Wani MR, Jan BL, Khan S. Characterization of induced high yielding cowpea mutant lines using physiological, biochemical and molecular markers. Sci Rep. 2020;10:3687. doi:10.1038/s41598-020-60601-6

Supporting Information

S1 Table. Trait dataset and cluster grouping of individual M_1 cowpea plants across EMS treatments and control

S2 Table. PCA loadings and variance explained for traits in M_1 cowpea

Effect Of of Ethyl Methane Sulfonate Mutagenesis on Phenological, Yield-Related and Yield Traits In in Cowpea *(Vigna unguiculata* (L.) Walp)

Henry .K. Mensah[1¶*], Ralieva A.K. Norety[1¶], Isaac .K. Asante[1&], Felicia Oppong[1&]

[1]Department of Plant and Environmental Biology, University of Ghana, Accra, Ghana

*Corresponding author

henryquofi@gmail.com (HKM)

.

[¶] These authors contributed equally to this work.

[&]These authors also contributed equally to this work.

Abstract

This study investigated the impact of ethyl methane sulfonate (EMS) on the M_1 generation of cowpea (*Vigna unguiculata* (L.) Walp). Seventy-five seeds of '*Wang Kae*' cowpea variety were soaked in water for six hours and later subjected to three different doses of ethyl methane sulfonate (20 mM, 40 mM and 80 mM)the 'Wang Kae' cowpea variety were soaked in water for six hours and later subjected to three different doses of ethyl methane sulfonate (20 mM, 40 mM, and 80 mM), with fifty serving as a control. Phenological, yield-related, and yield-specific data were collected in accordance with the International Board for Plant Genetic Resources cowpea descriptor. Data analysis was performed using Jamovi 2.7.15 and Jasp 0.95.4.0 to compute one-way ANOVA and post hoc contrast, principal component biplot, and cluster analyses. No optimal EMS concentration (LD50) for inducing mutations was identified. The maximum seed germination rate of 89.33% and seedling survival rate of 74.67% were recorded at 80 mM, whereas the control group exhibited the lowest rates, with 70.00% germination and 62.00% survival. The trend observed for both seed germination and seedling survival rates was: 0 mM < 20 mM < 40 mM < 80 mM. Significant differences ($P<0.05$) in all phenological traits, pod length, locule number, seed traits, and yield per plant were found between the control and treatments. Yield was significantly higher ($P = 0.047$) at 20 mM, with a mean of 61.19 ± 3.34 g, compared to the control. Contrast analysis identified genotypes B33 and D56 as the most productive mutants, yielding 125.44 g and 111.85 g, respectively. Principal component analysis produced eighteen components, with the first four accounting for 50.60% of total variation. Biplot analysis revealed that PC1 and PC2 encompassed all phenological traits, key seed traits, and yield attributes, especially for genotypes B33 and D56. Cluster analysis divided the 190 genotypes into six groups, with B33 and D56 constituting distinct clusters 4 and 1, respectively. In conclusion, the EMS mutagenesis was effective in inducing the unique variations that will be useful for breeding and development of new farmer

unique variations that will be useful for breeding and the development of new farmer-preferred varieties.

Introduction

Cowpea (Vigna unguiculata [L.] Walp.) is a widely cultivated and nutritionally important legume, predominantly grown in tropical and subtropical regions, particularly in Sub-Saharan Africa, Asia, and Central and South America, while also being adapted to certain temperate zones such as the Mediterranean basin and the southern regions of the United States [1-3].Cowpea (*Vigna unguiculata)* is an essential legume that is commonly grown in the developing countries of the tropics and sub-tropics especially in Sub Saharan Africa, Asia, Central, and South America, and in some temperate areas like the Mediterranean region and the Southern states of the USA.

Due to a drop in the market value of cowpeas due to pests, pathogens, and a lack of genetic variability, many scientists around the world have attempted to develop cowpea varieties to fulfilecline in cowpea market value caused by pests, pathogens, and a lack of genetic variability, many scientists worldwide have sought to develop cowpea varieties that meet the taste and demand of a growing population [14-37]. Previous studies have demonstrated substantial genetic variability in cowpea for economically important traits, including protein and mineral composition, underscoring the need to broaden the genetic base through breeding or mutagenesis [11,12]. The most noteworthy studies have used mutagens to try to boostincrease genetic variability [14-710]. Ethyl Methane Sulfonate (EMS), one of the most effective mutagens, has been successfully used by several researchers in West Africa to improve genetic variability in cowpea [36, 138].

Formatted: Default Paragraph Font, Font:

MA mutation is a heritable alteration in the structure or nucleotide sequence of a gene that arises suddenly and can be transmitted to subsequent generations. It results from changes in DNA such as substitutions, insertions, deletions, or rearrangements, and may occur naturally through replication or repair errors or be intentionally induced by physical and chemical mutagens. In plants, mutations can be fixed in both seed-propagated and vegetatively propagated species, allowing stable transmission of new traits. Consequently, mutations constitute a fundamental source of genetic variation and play a critical role in plant breeding and crop improvement programs utation is a heritable alteration in the structure of a gene that occurs suddenly in an organism. It is caused by a change in the base sequence of genes, and it can happen naturally or intentionally in both seed and vegetatively propagated plants [914, 1015].

Formatted: English (United States)

Formatted: Turkish

To create usefufunctional mutants in various crop species, a variety of chemical mutagens have been used. The artificial induction of mutation by colchicine (Col), ethyl methane sulphonates (EMS), and sodium azide (SA) provides a tool to overcome the limitations of variability in plants, particularly Carnation (*Dianthus caryophyllus*)., that induces specific improvement without disturbing their better attributes, according to the various researchers [614]. The present study was undertaken to test the efficacy of EMS in inducing variability in phenological, yield-related and yield characters of cowpea for, and yield characters of cowpea, thereby enabling the selection of superior genotypes for these importantessential traits.

Materials & Methods

Experimental site, material and design

The field study was carried out at the Department of Plant and Environmental Biology, University of Ghana, from early April to July 2024. Seventy-five seeds of the ‘*Wang Kae*’ cowpea variety (M_0 generation) were soaked in water for six hours and later subjected to three different doses of ethyl methane sulfonate (20 mM, 40 mM, and 80 mM), with 50 serving as a

control. Genotypes were labelled according to the EMS treatment dose. Genotypes exposed to 0 mM EMS were designated A1 A31 , those treated with 20 mM EMS were labelled B1 B48, genotypes subjected to 40 mM EMS were designated C1 C55 , and those treated with 80 mM EMS were labelled D1 D56. The treated seeds were thoroughly washed in running water for 30 minutes to reduce the residual effect of the mutagen sticking to the seed coat. The treated seeds together with the control were planted to raise the M_1 generation, along with the control, were planted to raise the M1 generation, and standard cultural practices were carried out thereafter. The experiment was designed in pots with dimensionmeasuring 3.29 m^2. Spacing used was 0.8m between lines and 0.5 m between rows. One cowpea seed were planted per hole in each pot at a depthas planted per hole in each pot 3 cm to , at a depth of 3-4 cm.

Parameters studied in the M_1 generation

The growth, yield, and yield-related parameters studied in the M1 generation were grouped as phenological: days to germination (*dtg*), days to first flower (*dff*), days to 50% flowering (d50f), days to first harvest (*dfh*), days to 50% mature pod (*d50mp*); and yield-related traits: peduncle length at first harvest (*pel*), number of pods per peduncle (*npp*), number of pods per plant at maturity (*nptm*), number of seeds per pod (*nsp*), number of seeds per plant (*nspt*), pod length (*pdl*), pod width (*pdw*), number of locules (*ln*), percent seed abortion (*psa*), seed length (*sdl*), seed width (*sdw*), seed thickness (*sdt*), seed weight (*swg*), and yield per plant (*yld*).

Percentage seed abortion was calculated as;

$$\frac{[(number\ of\ locules - number\ of\ seed\ per\ pod)]}{number\ of\ locules}\ x\ 100\% \ldots\ldots\ldots 1$$

Yield (g) was calculated

as; : $$\frac{100\ seed\ weight\ (g)\ x\ number\ of\ seeds\ per\ plant}{100} \ldots\ldots\ldots 2$$

Statistical analysis

Germination and survival rate

Germination of seedsSeed germination was recorded 7 days after planting. Survival plants were recorded at 50% flowering, and germination and survival rate were recorded using the formula by [1116]

$$Germination\ rate\ (\%) = \frac{(number\ of\ germinated\ seeds)}{(number\ of\ (un)treated\ seeds\ planted)}\ x\ 100 \ldots\ldots\ldots\ldots 3$$

$$Survival\ rate\ (\%) = \frac{(number\ of\ survived\ seedlings)}{(number\ of\ germinated\ seeds)}\ x\ 100 \ldots\ldots\ldots\ldots 4$$

[1116]

XLSTAT was also used to generate the linear regression equation of seedling germination and survival rate.

Distribution and post hoc contrast analysis for the selection of putative traits and mutants'

Descriptive statistics, one-way ANOVA and post hoc contrast analysis were analyzed for each trait to compare differences between treatments using Jamovi 2.7.15.0 software, and post hoc contrast analysis were used to compare treatment effects for each trait in Jamovi 2.7.15.0. Plants within treatments whose values fell outside the control range for each character and were statistically significant from the control were selected as putative mutants.

Multivariate analysis for the verification of the selected putative mutant traits and genotypes

Principal Component Analysis (PCA) was carried out accompanied by biplot analysis usingperformed, accompanied by biplot analysis in Jamovi 2.7.15.0. Principal Component Analysis for quantitative traits was employed to determine the percentage contribution of each trait to total genetic variation. The principal components (PCs) with eigenvalues >1 wasere selected, and traits that had coefficients >0.3 were considered to have significantly contributed

towith coefficients >0.3 were considered to have significantly contributed to the variability. The biplot plotted computed similarity values to show the relationship among the traits for each treatment and genotypesdisplayed computed similarity values to show the relationships among traits for each treatment and genotype. Cluster analyses were performed using Jamovi 2.7.15.0 was to group genotypes within treatments using the method ofto group genotypes within treatments using the Ward. D2 hierarchical clustering optimization criterion. The circular dendrogram plotted from the computed similarity values showed the relationship among the genotypes and traits that showed significant difference, plotted from the computed similarity values, showed relationships among genotypes and traits that differed significantly among treatments.

Results and discussion

Percentage germination and survival of M_1 plants

The results for seed germination and survival rates (%) and linear regression fits for M_1 cowpea genotypes subjected to three EMS doses for 6 h are presented in Fig 1. The highest seed germination rate (89.33%) and seedling survival rate (74.67%) were recorded at 80 mM, whereas the lowest germination rate (70.00%) and seedling survival rate (62.00%) were observed in the 0 mM (control) treatment. The percentage seed germination and seedlings survival were in this order;s of seed germination and seedling survival were in this order: 0 mM < 20 mM < 40 mM < 80 mM.

Linear regression analysis revealed a strong positive relationship between EMS concentration and both seed germination and survival rate (Fig 1). For seed germination rate, the regression equation was $y = 6.4667x + 65.333$ with an R^2 value of 0.9395. Seedling survival showed a positive linear relationship with EMS concentration as follows:

$y = 4.7333x + 56.667$ with an R^2 value of 0.9042.

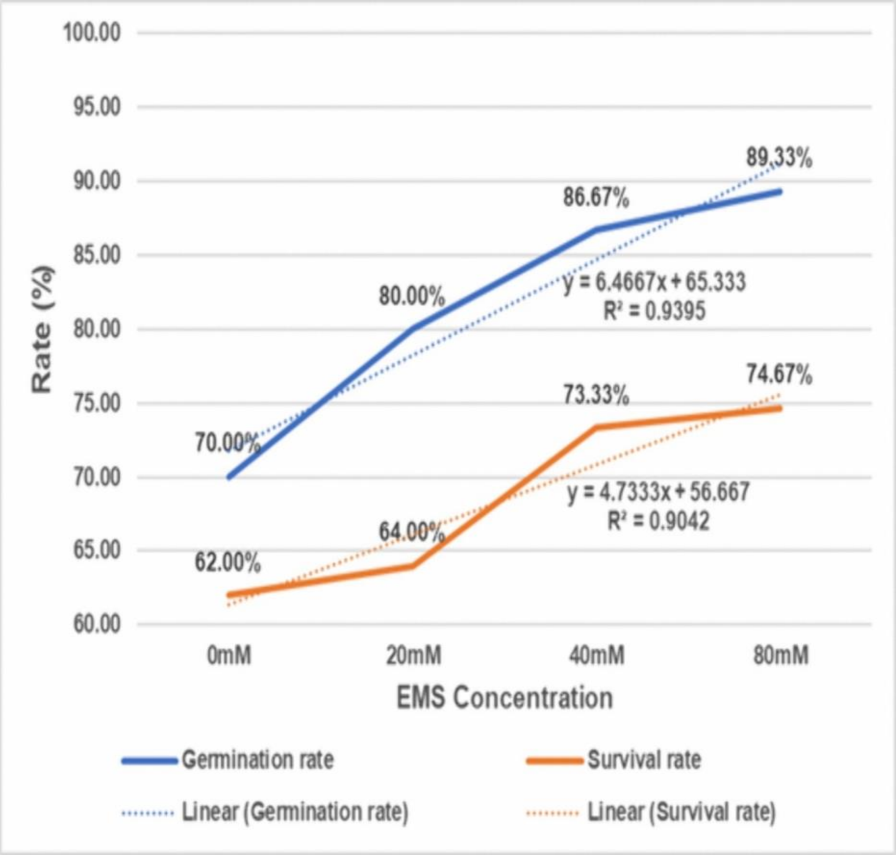

Fig 1: Seed germination and survival rates (%) and linear regression fits for M_1 cowpea genotypes subjected to three ethyl methanesulfonate (EMS) doses for 6 h.

In the present study, LD_{50} was not achieved, as seed germination and seedling survival increased with increasing EMS concentration. This response differs from the findings of [914, 1217–1419]. [1417], who reported a clearn apparent dose-dependent reduction in germination and survival in the M_1 generation of cowpea varieties *Pusa Komal* and *V-240 (Rambha)*

following 0.1–0.3% EMS treatmentsEMS treatments at 0.1–0.3%, with control germination exceeding 95% and higher doses approaching the LD_{50} threshold. Similarly, [813] observed a strong negative linear relationship between EMS concentration and germination in cowpea and identified an optimal LD_{50} dose at 0.4% EMS. The contrast observed in this current study where germination and survival were lowest in the control and increased with EMS concentration indicate was that germination and survival were lowest in the control. It increased with EMS concentration, indicating that LD_{50} expression is highly dependent on genotype, EMS dose range, and exposure duration. Unlike previous studies that employed percentage-based EMS concentrations and different treatment protocols, the present study used millimolar EMS concentrations with a fixed 6-h exposure, which may have altered the physiological response. In addition, the relatively low germination and survival recorded in the control suggest baseline establishment constraints that may have obscured EMS-induced lethality. These findings indicate that the absence of an LD_{50} endpoint in the present study does not imply insufficient mutagenic effect. RatherInstead, it underscores the importance of genotype × dose × exposure-time interactions in EMS mutagenesis.

Contrast analysis of traits in the M_1 generation

Phenological traits

Days to germination (*dtg*) differed significantly (P < 0.001) among treatmentsamong treatments (P < 0.001). The control (0 mM) germinated earliest, with a mean of 3.45 ± 0.13 days and a range of 2–5 days. All EMS treatments delayed germination, with the strongestmost substantial effect at 20 mM, while 40 and 80 mM showed delayed but comparable responses. The frequency of putative mutants was 50.0% at 20 mM, 29.10% at 40 mM, and 30.36% at 80 mM (Table 1). Selected putative mutants can be found in S1 Table.

Days to first flower (*dff*) were significantly (P < 0.001) affected by EMS among treatmentsaffected by EMS across treatments (P < 0.001). *Dff* was*FF was the* earliest in the

control, with a mean of 37.87 ± 0.77 days and values ranged froma range of 30 to 45 days, and was progressively delayed with increasing EMS concentration, reaching a maximum mean of 45.04 ± 0.86 days at 40 mM. Mutant frequencies declined from 51.17% at 20 mM to 41.07% at 80 mM (Table 1). Selected putative mutants can be found in S1 Table.

Table 1: Mean, variability, mutant frequency, and pairwise significance of phenological traits in the M_1 generation of cowpea under different EMS concentrations

Trait	Trt	Mean±se	%CV	Min	Max	Freq	P	Significant pairwise comparisons			
								0mM	20mM	40mM	80mM
dtg	0mM	3.45±0.13$_a$	20.90	2	5		<0.001		<0.001	<0.001	<0.001
	20mM	5.38±0.20$_b$	25.30	3	7	50.00		<0.001			
	40mM	4.78±0.19$_b$	29.20	3	7	29.10		<0.001			
	80mM	4.77±0.19$_b$	29.70	3	7	30.36		<0.001			
dff	0mM	37.87±0.77$_a$	11.30	30	45	-	<0.001	-	<0.001	<0.001	<0.001
	20mM	44.69±0.91$_b$	14.10	33	54	51.17		<0.001			
	40mM	45.04±0.86$_b$	14.10	34	55	47.27		<0.001			
	80mM	44.46±0.79$_b$	13.30	33	55	41.07		<0.001			
d50f	0mM	57.84±0.92$_a$	8.90	48	68	-	<0.001	-	<0.001	<0.001	<0.001
	20mM	64.67±0.99$_b$	10.70	50	75	37.50		<0.001			
	40mM	65.18±0.97$_b$	11.00	49	80	40.00		<0.001			
	80mM	64.79±0.85$_b$	9.80	53	80	28.57		<0.001			
dfh	0mM	49.35±1.15$_a$	13.00	40	67	-	<0.001	-	-	-	-
	20mM	60.17±0.94$_b$	10.80	48	72	18.75		<0.001			
	40mM	58.93±0.97$_b$	12.10	38	72	9.100		<0.001			
	80mM	58.50±0.91$_b$	11.70	43	72	12.50		<0.001			
d50mp	0mM	79.48±1.41$_a$	9.90	66	94	-	<0.001	-	<0.001	<0.001	<0.001
	20mM	90.17±1.37$_b$	10.50	68	107	39.58		<0.001			
	40mM	88.65±1.09$_b$	9.10	68	106	25.45		<0.001			
	80mM	88.32±1.16$_b$	9.80	69	110	23.21		<0.001			

Note. Means sharing the same superscript letter within a trait are not significantly different according to Tukey's HSD test ($P \leq 0.05$). Trt= Treatment, Mean ± SE = Mean plus or minus standard error, %CV= Percentage coefficient of variation, Min= minimum, Max, = maximum, Freq= Percentage frequency of putative mutants; P=Probability value. Traits: dtg = days to germination, dff = days to first flower, d50f = days to 50% flowering, dfh = days to first harvest, d50mp = days to 50% mature pods

Days to 50% flowering (*d50f*) were significantly ($P < 0.001$) affected by EMS among treatmentsaffected by EMS across treatments ($P < 0.001$). ***D50f*** increased from a control mean of 57.84 ± 0.92 days, with values ranging from 48 to 68 days, to mean values of approximately 65 days across EMS treatments, with broader ranges of 49–80 days. Corresponding mutant frequencies were 37.50%, 40.00%, and 28.57% at 20, 40, and 80 mM, respectively (Table 1). Selected putative mutants can be found in S1 Table.

Days to first harvest *(dfh)* was earliest in the control, with a mean of 49.35 ± 1.15 days and a range of 40–67 days and wasere the earliest in the control, with a mean of 49.35 ± 1.15 days and a range of 40–67 days, and were significantly ($P < 0.001$) delayed in all EMS treatments. Mean dfh increased to 60.17 ± 0.94 days at 20 mM, 58.93 ± 0.97 days at 40 mM,

and 58.50 ± 0.91 days at 80 mM. Mutant frequencies were 18.75% at 20 mM, 9.10% at 40 mM, and 12.50% at 80 mM (Table 1). Selected putative mutants can be found in S1 Table.

A similar trend was observed for **days to 50% maturity of pods (*d50mp*)**, where the control recorded the earliest maturity of 79.48 ± 1.41 days, with valuesith the control recording the earliest maturity at 79.48 ± 1.41 days, and values ranging from 66 to 94 days. EMS treatments significantly (P<0.001) delayed in pod maturity frompod maturity compared with the control, with mean ***d50mp*** values of 90.17 ± 1.37 days at 20 mM, 88.65 ± 1.09 days at 40 mM, and 88.32 ± 1.16 days at 80 mM. The frequency of selected mutants for d50mp was 39.58%, 25.45%, and 23.21% at 20, 40, and 80 mM, respectively (Table 1). Selected putative mutants can be found in S1 Table.

In our study, EMS treatments affected the timing of developmental events of flowering and maturity in cowpealtered the timing of developmental events in cowpea, including flowering and maturity. The expansion of phenotypic variability observed in the present EMS-treated population is particularly relevant for cowpea improvement, as earlier studies have demonstrated that key agronomic and nutritional traits are under strong genetic control and respond effectively to selection when sufficient genetic variability is available, [3, 11-12]. Days to first flowering and days to 50% flowering tended to be delayed in many high-dose EMS mutants, although some early flowering outliers were observed at lower doses. The delay in flowering under stronger mutagenesis is a common phenomenon, often attributed to physiological stress or disruption of genes controlling flowering time [15–18to disruption of genes controlling flowering time [20-23]. [1420] did not directly measure flowering time, but their M_1 mutants showed overall physiological delays, and other researchers have explicitly noted flowering extensions. For example, [1924] working in mung bean reported that higher EMS levels prolonged days to flowering and maturity, likely due to mutational interference

Formatted: English (United States)

inwith mung bean reported that higher EMS levels prolonged the time to flowering and maturity, likely due to mutational interference with developmental pathways. It is also recorded by [2025] in black gram, [2126] in *Gloriosa superben*, and [58, 2227] in *Vigna radiata*. In cowpea, [2328] interestingly observed an *earlier* onset of flowering,observed an earlier onset of flowering and a decrease in days to 50% flowering in an EMS-treated yardlong bean (*Vigna sesquipedalis*) population, accompanied by reduced maturity duration.

Such wide variation in flowering time due to EMS has also been reported by [813], who found that their M_2 cowpea population showed continuous distribution for days to flowering far beyond the range of the wild type. This implies that EMS created both later- and earlier-flowering individuals. Notably, [813] identified dozens of mutants that flowered significantly ($P<0.05$) earlier than the parent variety, reflectingindicating the emergence of new earliness traits. These results suggest that certainspecific mutations can accelerate flowering, potentially by knocking out repressors of flowering even as overall vigor is reduced. In our mutants, while the predominant trend at high EMS dosages was delayed flowering and maturity, a few early blooming mutants from lower EMS treatments were record blooming mutants edwere observed at lower EMS treatments, indicating increased phenological variation.

Our results confirm that EMS mutagenesis increased flowering time variancthe variance in flowering time. While early flowering is advantageous for drought avoidance and multi-cropping systems, our evaluation of the EMS population did not yield any early-flowering mutants; ratherinstead, such occurrences were observed in the control group. This outcome aligns with results previously reported in mung bean and other pulse crops.

Yield-related and yield traits

The **pod length (*pdl*)** was highest in the control (0 mM), with a mean of 14.65 ± 0.31 cm and a range of 11.55–17.90 cm whereas 80mM recorded the lowest mean ***pdl*** of 13.52±0.28 cm which was the significantly ($P=0.008$) different, whereas 80mM recorded the lowest mean

pdl of 13.52±0.28 cm, which was significantly different (P=0.008) from the control. Based on deviations from the control range, the frequency of selected mutants for ***pdl*** was 10.42, 9.10 and 21.43% in 20 mM, 40 mM and 80 mM%, 9.10%, and 21.43% in 20 mM, 40 mM, and 80 mM, respectively (Table 1). Selected putative mutants can be found in S1 Table.

The ***number of locules per pod (ln)*** differed significantly among EMS treatments (P < 0.001). The control recorded a mean of 17.00 ± 0.60, with values ranging froma range of 12 to 24 locules. Higher EMS concentrations resulted in a significant reduction in locule number for 40 mM at 14.93 ± 0.48 locules and further to: 14.93 ± 0.48 locules at 40 mM and 11.41 ± 0.39 locules at 80 mM. Pairwise comparisons showed that only the 80 mM treatment differed significantly (P < 0.001). from both the control and all other treatments, Phenotypic variability increased with EMS dose, as reflected by the rise in coefficient of variation from 17.5% in From both the control and all other treatments, Phenotypic variability increased with EMS dose, as reflected by a rise in the coefficient of variation from 17.5% at 20 mM to 25.8% at 80 mM. Based on deviations

Table 2: Mean, variability, mutant frequency, and pairwise significance of yield-related and yield traits in the M_1 generation of cowpea under different EMS concentrations

Trait	Trt	Mean±se	%CV	Min	Max	Freq	P	Significant pairwise comparisons			
-	-	-	-	-	-	-	-	0mM	20mM	40mM	80mM
npp	0mM	3.84±0.17	25.30	2	5	-	0.447	-	-	-	-
	20mM	4.02±0.13	21.50	2	5						
	40mM	3.82±0.13	25.70	2	5						
	80mM	3.70±0.16	31.80	2	5						
nptm	0mM	24.68±1.31	29.50	14	44	-	0.412	-	-	-	-
	20mM	25.46±1.07	29.10	10	45						
	40mM	23.25±0.78	25.00	11	44						
	80mM	23.87±0.97	30.40	10	46						
nsp	0mM	9.84±0.63	35.60	5	15	-	0.730	-	-	-	-
	20mM	9.92±0.46	32.30	5	15						
	40mM	10.44±0.39	27.80	5	15						
	80mM	9.80±0.46	35.10	5	15						
nspt	0mM	233.10±14.38	34.30	100	406	-	0.689	-	-	-	-
	20mM	246.29±13.29	37.40	100	450						
	40mM	238.20±10.34	32.20	100	435						
	80mM	226.36±12.17	40.20	100	450						
pdl	0mM	14.65±0.31b	11.80	11.55	17.86	-	0.030	-	-	-	0.008

	20mM	14.34±0.25ab	12.30	10.50	19.88	10.42					
	40mM	13.83±0.26ab	14.10	6.95	16.75	9.10					
	80mM	13.52±0.28a	15.20	8.45	17.50	21.43		0.008			
pdw	0mM	0.85±0.01	9.70	0.70	1.03	-	0.144	-	-	-	-
	20mM	0.89±0.02	16.50	0.61	1.23						
	40mM	0.90±0.03	25.60	0.52	1.28						
	80mM	0.83±0.02	18.00	0.60	1.13						
ln	0mM	17.00±0.60c	19.70	12.00	24.00	-	<0.001	-	-	0.023	<0.001
	20mM	17.06±0.43c	17.50	12.00	23.00					0.005	<0.001
	40mM	14.93±0.48b	23.90	8.00	21.00	20.00		0.023	0.005		<0.001
	80mM	11.41±0.39a	25.80	7.00	23.00	50.00		<0.001	<0.001	<0.001	
psa	0mM	14.27±1.93	75.30	0.00	35.71	-	-	-	-	-	-
	20mM	18.60±1.68	62.70	0.00	35.71						
	40mM	19.35±1.66	63.80	0.00	35.71						
	80mM	15.43±1.53	74.20	0.00	35.71						
sdl	0mM	7.44±0.24a	18.10	5.68	11.59	-	<0.001	-	<0.001	<0.001	<0.001
	20mM	8.77±0.25b	19.60	6.10	11.65	0.42		<0.001		<0.001	<0.001
	40mM	9.79±0.07c	4.90	8.10	11.05			<0.001	<0.001		
	80mM	9.95±0.09c	6.60	7.95	11.60	1.79		<0.001	<0.001		
sdw	0mM	4.78±0.10a	11.90	3.94	6.08	-	<0.001	-	<0.001	<0.001	<0.001
	20mM	5.14±0.09b	11.80	4.05	6.44	0.42		<0.001			
	40mM	5.14±0.04b	5.90	4.58	5.69			<0.001			
	80mM	5.25±0.05b	7.00	4.28	6.11	1.79		<0.001			
sdt	0mM	4.09±0.07a	9.80	3.29	4.92	-	<0.001	-	0.027	-	-
	20mM	4.35±0.08b	12.20	3.31	5.19	12.50		0.027		<0.001	0.005
	40mM	3.95±0.04a	8.50	3.43	5.59	1.82			<0.001		
	80mM	4.08±0.05a	8.90	3.41	4.94	1.79			0.005		

Note. Means sharing the same superscript letter within a trait are not significantly different according to Tukey's HSD test (P ≤ 0.05). Trt= Treatment, Mean ± SE = Mean plus or minus standard error, %CV= Percentage coefficient of variation, Min= minimum, Max=, maximum, Freq= Percentage frequency of putative mutants; P=Probability value. Traits: npp = number of pods per peduncle, nptm = number of pods per plant at maturity, nspt = number of seeds per plant, pdl = pod length, pdw = pod width, ln = number of locules, nsp = number of seeds per pod, psa = percent seed abortion, sdl = seed length, sdw = seed width, sdt = seed thickness, swgt = seed weight, yld = yield per plant.

Table 2: Mean, variability, mutant frequency, and pairwise significance of yield-related and yield traits in the M_1 generation of cowpea under different EMS concentration (*cont'd*)

Trait	Trt	Mean±se	%CV	Min	Max	Freq	P	Significant pairwise comparisons			
								0mM	20mM	40mM	80mM
swg	0mM	21.03±0.34a	9.10	17.82	24.87	-	<0.001	-	<0.001	<0.001	0.020
	20mM	24.86±0.34c	9.40	17.81	29.99	54.17		<0.001			<0.001
	40mM	24.37±0.35c	10.70	17.70	28.39	49.10		<0.001			<0.001
	80mM	22.60±0.33b	11.00	17.35	28.68	16.07		0.020	<0.001	<0.001	
yld	0mM	48.79±3.01a	34.40	20.61	85.68	-	0.018	-	0.047	-	-
	20mM	61.19±3.34b	37.80	18.70	125.44	16.67		0.047			
	40mM	58.02±2.59ab	33.10	19.97	105.43	12.72					
	80mM	51.16±2.87ab	42.00	18.45	111.85	10.71	-	-	-	-	-

Note. Means sharing the same superscript letter within a trait are not significantly different according to Tukey's HSD test (P ≤ 0.05). Trt= Treatment, Mean ± SE = Mean plus or minus standard error, %CV= Percentage coefficient of variation, Min= minimum, Max=, maximum, Freq= Percentage frequency of putative mutants; P=Probability value. Traits: npp = number of pods per peduncle, nptm = number of pods per plant at maturity, nspt = number of seeds per plant, pdl = pod length, pdw = pod width, ln = number of locules, nsp = number of seeds per pod, psa = percent seed abortion, sdl = seed length, sdw = seed width, sdt = seed thickness, swgt = seed weight, yld = yield per plant.

from the control range, the frequency of putative mutants for ***ln*** reached 20.0% at 40 mM and 50.0% at 80 mM (Table 1). Selected putative mutants can be found in S1 Table.

Seed length (*sdl*) varied significantly across EMS treatments (P < 0.001). The control recorded the lowest mean seed length of 7.44 ± 0.24 mm(7.44 ± 0.24 mm), with values ranging froma range of 5.68 to 11.59 mm and a CV of 18.1%. EMS treatment groups resulted in a progressive increase in seed length, with means of 8.77 ± 0.25 mm at 20 mM, 9.79 ± 0.07 mm at 40 mM, and 9.95 ± 0.09 mm at 80 mM. Pairwise comparisons showed that all EMS treatments differed significantly from the control (P < 0.001), while the 40 mM and 80 mM treatments did not differ from each other. Based on deviations from the control range, mutant frequencies were low for seed length, recorded at 0.42% for 20 mM and 1.79% for 80 mM (Table 1). Selected putative mutants can be found in S1 Table.

Seed width (*sdw*) was also significantly (P < 0.001) affected by EMS treatment. The control population recorded a mean width of 4.78 ± 0.10 mm and ranged from 3.94–6.08 mmhad a mean width of 4.78 ± 0.10 mm, ranging from 3.94 to 6.08 mm, with a CV of 11.9%. All EMS treatments differed significantly from the control (P < 0.001). Variability declined at higher EMS doses, with CV values as low as 5.9% at 40 mM. Frequencies of putative mutants increased with EMS concentration, reaching 0.42% at 20 mM and 1.79% at 80 mM (Table 1). Selected putative mutants can be found in S1 Table.

Seed thickness (*sdt*) differed significantly among treatments (P < 0.001). The control recorded a mean thickness of 4.09 ± 0.07 mm with values ranging from 3.29 to 4.92 mm. Thickness increased significantly (P=0.027) at 20 mM with a mean of 4.36 ± 0.08 mm. Pairwise comparisons indicated significant differences between 20 mM and both 40 mM (P < 0.001) and 80 mM (P = 0.005). Mutant frequencies for seed thickness were highest at 20 mM (12.5%)

but remained low at 40 mM (1.82%) and 80 mM (1.79%) (Table 1). Selected putative mutants can be found in S1 Table.

100-Seed weight (*swg*) showed highly significant variation among EMS treatments (, P < 0.001). The control recorded a mean of 21.03 ± 0.34 g and values ranged, with froma range of 17.82 to 24.87 g. EMS treatment increased seed weight, with the highest means observed at 20 mM with a value of 24.86 ± 0.34 g and 40 mM weighed 24.37 ± 0.35 g(24.86 ± 0.34 g) and 40 mM (24.37 ± 0.35 g), both of which differed significantly from the control (P < 0.001). The 80 mM treatment weighed 22.60 ± 0.33 g and showed an intermediate response and differed significantly from both the control (P = 0.02) and lower EMS doses (P<, differing considerably from both the control (P = 0.02) and lower EMS doses (P < 0.001). Frequencies of putative mutants were highest at 20 mM (54.17%), followed by 40 mM (49.1%) and 80 mM (16.07%) (Table 1). Selected putative mutants can be found in S1 Table.

Yield per plant (*yld*) differed significantly among treatments (P = 0.018). The control population recorded the lowest mean yield of 48.79 ± 3.01 g, with values ranging froma range of 20.61 to 85.68 g. Yield increased significantly (P=0.047) at 20 mM = 0.047) at 20 mM, with a mean of 61.19 ± 3.34 g, which differed from the control (P = 0.047). The means of 40 mM, 58.02 ± 2.59 g and 80 mM, 51.16 ± 2.87 g treatments exhibited intermediate yields and40 mM (58.02 ± 2.59 g) and 80 mM (51.16 ± 2.87 g) treatments, which exhibited intermediate yields, did not differ significantly from either the control or 20 mMthe 20 mM treatment. Phenotypic variability was high across treatments with a CV between 33.10 , with a CV of 33.10–42%, and mutant frequencies declined with increasing EMS concentration, from 16.67% at 20 mM to 10.71% at 80 mM (Table 1). Selected putative mutants can be found in S1 Table.

The lack of notable mutants for number of pods per peduncle (*npp*), number of pods per plant at maturity (*nptm*), number of seeds per pod (*nsp*) and number of seeds per plant

(*nspt*) in the M_1 generation is likely due to these traits being highly polygenic and developmentally buffered, which restricts observable changes [2429- 2631]. Yield components may mask mutational effects through compensatory relationships [2732, 2833]. However, significant mutants were identified for these traits in M_2 populations, where recessive mutations are revealed and chimerism is resolved [8, 29,- 31 and chimerism is resolved [13, 34-36]. Moreover, higher EMS doses often reduce pod and seed numbers due to partial sterility or lower pollen viability, as seen in legumes [1419, 2025, 3237].

In the present study, several putative mutants from the 20 mM, 40 mM, and 80 mM EMS treatments exhibited significantly increased pod length, seed dimensions, seed weight, and yield per plant relative to the control. Comparable yield-enhancing mutations have been reported in earlier cowpea studies. For example, [813] identified M_2 cowpea mutants with significantly higher pods per plant, seeds per plant, seeds per pod, and pod length than the wild type. Those authors further noted that increased pod number, seed weight, and plant stature have frequently been achieved through EMS mutagenesis in cowpea.

Seed size traits also varied widely, with some mutants significantly affected withhowing significantly longer and wider seeds in the present study. Variations in seed size and structure are particularly important in cowpea, as seed morphological traits have been associated with differences in protein content and mineral composition among genotypes, highlighting the potential nutritional relevance of EMS-induced mutants [3, 11-12]. Increased seed size due to mutagenesis has been reported previously; [33] [38]; isolated bold-seeded mutants in *Vigna mungo* with enhanced yield. In our studyresearch, high-yielding mutants, for example B09 typically combined larger seeds with and highsuch as B09, typically produce larger seeds with higher seed weight, a favorable combination for yield improvement.

Similarly, [3338] and [3439] reported cowpea mutants with enlarged seeds and improved yield performance, consistent with the superior lines identified in the present study.

Yield stimulation following mutagenic seed treatments has also been documented in other legumes. Increased yield or yield components after mutagen exposure have been reported in pigeonpea [3540], mung bean [3641], and mung bean treated with gamma rays, EMS, and epichlorohydrin [4237]. These studies collectively support the notion that induced mutagenesis can unlock favorable allelic variation controlling yield traits.

In contrast, many mutants particularly from the highest EMS dose (80 mM) exhibited reductions in yield and its components, including pod length, number of locules, seed size, particularly from the highest EMS dose (80 mM), exhibited reductions in yield and its components, including pod length, number of locules, seed size, and seed weight. Such negativadverse effects of high mutagen doses are well documented. [3843] in okra and [39] in petuniaIn okra and [44] in petunia, it was reported that mutagenesis frequently produces lines with reduced reproductive output. In cowpea, [4045] observed that EMS doses beyond the optimal range led to sharp declines in pollen fertility and seed yield, whil. At the same time, [4146] reported positive yield shifts at lower mutagen doses and negative shifts at higher doses. Our results closely mirror this dose-dependent pattern. Yield depression at elevated doses of irradiation or chemical mutagens has been widely reported. Similar declines were observed by [2025] in black gram, [2126] in *Gloriosa* Khan *superba* under EMS and DES treatments, [4247] in *Vigna mungo*, and [4348] in *Vigna radiata*.

These findings reflect the accumulation of deleterious mutations at higher mutagen doses, which can impair physiological and reproductive processes. Conversely, [3237] reported stimulation of seed weight in pigeon pea under combined mutagenic treatments, suggesting that moderate doses may enhance specific yield components. Similar observations were

reported by [4449], [4550], and Patil and [4651] across various pulse crops, reinforcing the dose- and trait-dependent nature of mutagenic responses.

In the present study, one elite mutant (B33) from the 20 mM EMS treatment produced approximately 25% higher seed yield than the control, accompanied by a modest increase in 100-seed weight. This indicates that EMS successfully generated yield-enhancing alleles. [4752] similarlySimilarly, reported EMS-derived cowpea lines with significantly increased pod number, confirming the potential of chemical mutagenesis to improve yield potentials confirm the potential of chemical mutagenesis to improve yield.

It is also noteworthy that the frequency of desirable mutants was low, consistent with previous reports. [813] estimated that only 0.1–1% of mutants exhibited significantly improved yield yield-related and yield traits. Similarly, although a few promising mutants were identified in the present study, the majority performed at or below the level of the control. This underscores the necessity of screeninged to screen large mutant populations to capture rare beneficial events.

Multivariate Analysis: PCA Biplot and Cluster Grouping

PCA Biplot of phenological and yield-related and yield traits

Principal component analysis (PCA) performed on the traits and genotypes plant observations across EMS treatments generated a total of 8 principal components with an eigenvalueof the traits and genotypes of plant observations across EMS treatments yielded 8 principal components with eigenvalues < 1 (S2 Table). The first four principal components accounted for a total variability greater than 50.60% in all treatments. The first two dimensions/principal components (Dim1 and Dim2) together explained 32.8% of the total phenotypic variation, with PC1 accounting for 17.7% and PC2 accounting for 15.1% of the variation (Fig 2,principal components (Dim1 and Dim2) together explained 32.8% of the total phenotypic variation, with

PC1 accounting for 17.7% and PC2 accounting for 15.1% (Fig 2, S2 Table). PC1 represented major seed and phenological traits, including seed length (*sdl*), seed width (*sdw*) days to first flower (*dff*), days to 50% flowering (*d50f*), days to first harvest (*dfh*), days to 50% pod maturity (*d50mp*) and *swg* loaded positively on PC1, indicating that higher PC1 scores were associated with delayed development. On the other hand, yield-related traits such as number of seeds per plant (*nspt*), number of seeds per pod (*nsp*), and yield (*yld*) were oriented in the opposite direction onnegatively correlated with PC2 reflecting an inverse relationship between prolonged phenology and yield-related and yiel, reflecting an inverse relationship between prolonged phenology and yield-related traits.

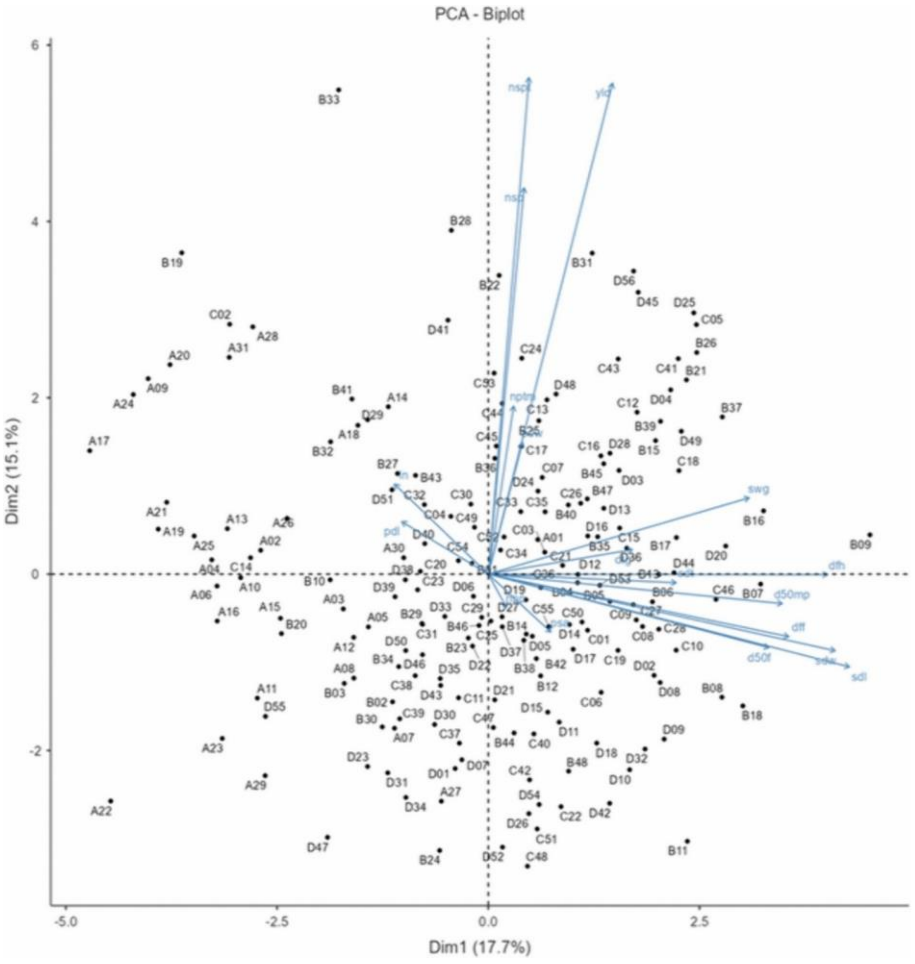

Fig 2: PCA biplot of cowpea genotypes and traits based on PC1 and PC2. Genotypes treated with 0-, 20-, 40- and 80-mM EMS are labelled A01–A31, B01–B48, C01–C55 and D01–D56, respectively. Vectors indicate trait loadings, and points represent genotype distribution. Percent variance explained by each PC is shown on the axes. Key: dtg days to germination, dff = days to first flower, d50f = days to 50% flowering, dfh = days to first harvest, d50mp = days to 50% mature pods, npp = number of pods per peduncle, nptm = number of pods per plant at maturity, nspt = number of seeds per plant, pdl = pod length, pdw = pod width, ln = number of locules, nsp = number of seeds per pod, psa = percent seed abortion, sdl = seed length, sdw = seed width, sdt = seed thickness, swgt = seed weight, yld = yield per plant

The distribution of genotypes plants on the PCA biplot revealed most control plants (0 mM) formed a compact cluster near the negative side of PC1, reflecting early phenology, uniform development, and moderate highly yield plants on the PCA biplot revealed that most control plants (0 mM) formed a compact cluster outputon the negative side of PC1, reflecting early phenology, uniform development, and moderate to high yield. Plants from the 20 mM EMS treatment showed partial overlap with the control group but. Still, they were moderately displaced toward positive PC1 values, indicating mild delays in phenological development and moderate reductions in yield components. Genotypes from the 40 mM and 80 mM EMS treatments were progressively displaced toward the positive side of PC1 and exhibited greater dispersion along

both PC1 and PC2.This shift reflects dose-dependent delays in flowering, harvest, and maturity, coupled with reductions in reproductive traits such as *nsp*, *nspt*, and *yld*.

Notably, vectors for seed length (*sdl*) and seed width (*sdw*) were oriented opposite to those for number of seed per pod and plants per pod, plant, and yield. Genotypes positioned along the *sdl*/*sdw* vectors from the 40 mM and 80 mM treatments were characterized byshowed larger seed size but reduced yield, suggesting altered resource allocation under EMS-induced mutational stress.

Selection focused on genotypes aligned with key trait vectors representing yield performance, seed size, and phenological timing. High-yielding putative mutants were identified among genotypes positioned in the upper region of the biplot with positive PC2 scores, in close alignment with the ***yld***, ***nsp***, ***nptm***, and ***swg*** vectors. These included B33, B28, B22, B31, D56, D45, D25, C5, C41, B26, and B21, representing EMS-derived plants with enhanced reproductive output relative to the population mean.

Large seed putative mutants were selected from genotypes located along the negative PC2 axis, in the direction of the sdl and sdw vectors. Representative genotypes included D23, D7, D34, D47, C37, C39, C40, C42, B18, B11, and B8, which were characterized byshowed increased seed size but reduced seed number and yield.

Early phenology putative mutants were identified from the extreme negative side of PC1, opposite to the late flowering and maturity vectors. These included B19 and C2, reflecting genotypes with accelerated development and earlier flowering and maturity.

Genotypes exhibiting extreme positive PC1 scores with weak alignment to yield vectors such as B9, B7, C46, and D20 were classified as severely delayed and low-performing and were not considered suitable for advancement but, such as B9, B7, C46, and D20, were classified as severely delayed and low-performing and were not considered suitable for advancement; however, they were retained as extreme phenotypes for genetic and physiological reference.

The PCA biplot in our study was especially informative, as it highlighted which traits drove mostparticularly informative, as it highlighted which traits accounted for the most variation among the EMS-treated lines. Notably, yield-related (*npp*, *nsp*, and *swg)* and yield traits loaded strongly on the first two principal components, indicating these had the most influence in differentiating mutants. Traits with minimal impact in PCA were also not highlighted in the clustering. By focusing on the PCA, we found a clear separation between high-yielding mutants and low-yielding ones along PC high-yield lines clustered and low-yielding mutants, with high-yield lines clustering in the quadrant associated with greater pod and seed numbers. In contrast, mutants with delayed phenology or poor fertility tended to lie on the opposite end of the PCA axes, separated from the productive genotypes. This reflects the underlying trade-offs induced by EMS: lines that suffered heavy damage for example dwarf

and sterile mutants, for example, dwarf and sterile mutants, clustered away from those that managed to improve or maintain yield traits. The utility of PCA in such mutation studies is well recognized; it helps distill many correlated traits into composite axes of variation.

[4853], for instance, used multi-trait analysis to identify superior M_2 individuals and reported that mutants with extreme high values in key traits such as seeds per plantFor instance, used multi-trait analysis to identify superior M_2 individuals and reported that mutants with extremely high values in key traits, such as seeds per plant, could be distinguished from the population distribution. Our PCA biplot offers similar insights by visually highlighting the direction of trait enhancement, such as higher yield, and demonstrating that the notable mutants distinctly deviated from the control group's path in multivariate analysis.

Cluster analysis of phenological and yield-related and yield traits

Hierarchical cluster analysis based on phenological, yield-related, and yield traits grouped the cowpea M_2 population into six distinct clusters (Fig 3, S1 Table). Cluster 1 comprised predominantly EMS-derived genotypes, representing 35.42%, 49.10%, and 33.93% of the 20 mM, 40 mM, and 80 mM treatment groups, respectively. Only a single control genotype (A01) clustered within this group, accounting for 3.22% of the 0 mM population. Genotypes in this cluster were characterized by prolonged phenological development coupled with superior yield performance. Days to 50% flowering (*d50f*) ranged from 53 to 80 days, with the most pronounced delays observed in C43 and C41, while days to 50% pod maturity (*d50mp*) ranged from 69 to 109 days, recorded in D43 and C08 respectivelywith the most extended delays recorded in D43 and C08.

Despite delayed development, individuals in this cluster exhibited markedly higher seed weight (*swg*), ranging from 18.82 g (D04) to 28.68 g (D56), and elevated yield per plant (*yld*), which ranged from 31.45 g (C37) to 111.85 g (D56), exceeding the control population. Selected putative mutants from Cluster 1 included D56, D45, D25, C41, C5, B31, B28, and

B22, all of which showed clearapparent positive deviation from the control range for majorsignificant yield and yield-related traits.

Cluster 2 was dominated by control genotypes, with 61.29% of the 0 mM population grouped within this clusteraccounting for 61.29% of the 0 mM population. Only 2.08%, 3.64%, and 3.57% of the 20 mM, 40 mM, and 80 mM treatment groups, respectively, were represented in this cluster. Genotypes in Cluster 2 exhibited early germination, flowering, and maturity, with yield levels comparable to the control mean. This cluster therefore represented the baseline phenotypic profile of the population and show, therefore, represented the population's baseline phenotypic profile and exhibited low within-cluster variability. A small number of EMS-treated genotypes within this cluster combined early days to first harvest with improved yieldshowed improved yield during the early days to first harvest, notably C02 (*38 days*) and C14 (*39 days*), which were selected as early-phenology putative mutants.

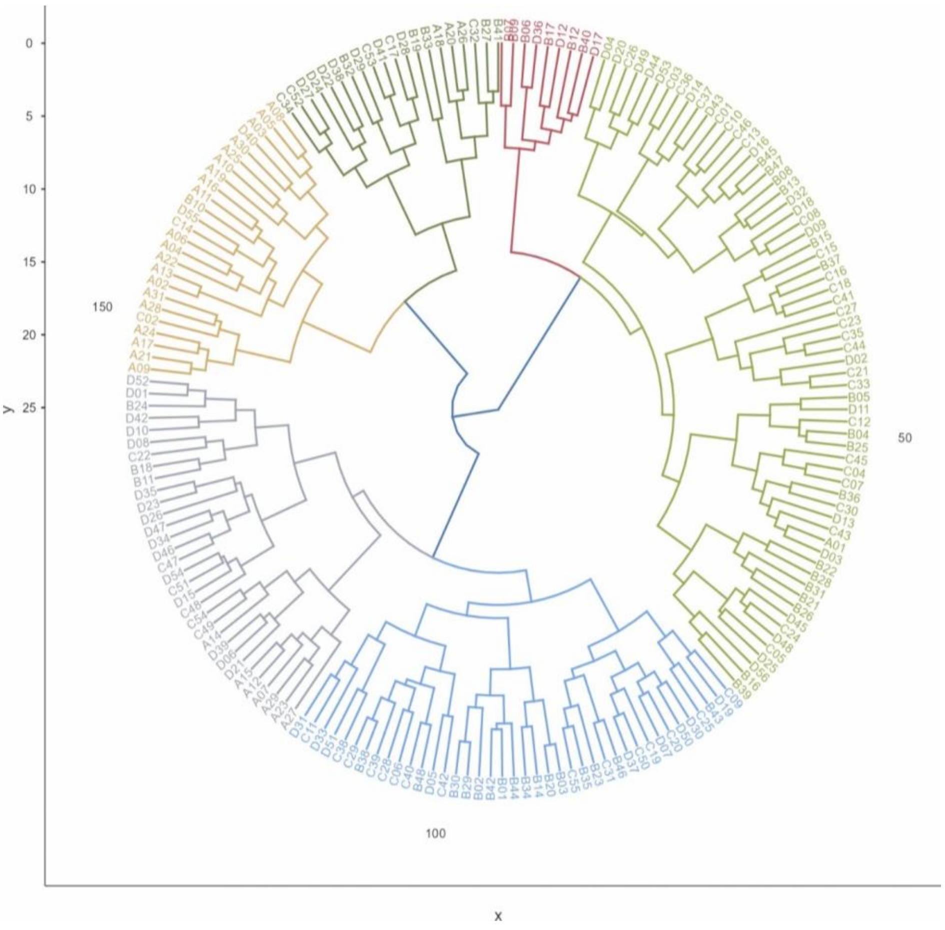

Fig 3: Dendrogram of hierarchical cluster analysis based on quantitative traits using Ward's linkage method, showing the genetic relationships among genotypes in the M_1 generation based on average distance. Key: A = 0mM, B = 20mM, C = 60mM and D = 80mM, Green = Cluster 1 [64 genotypes], Brown = Cluster 2 [24 genotypes], Grey = Cluster 3 [31 genotypes], Forest green = Cluster 4 [20 genotypes], Blue = Cluster 5 [40 genotypes], Red = Cluster 6 [9 genotypes]

Cluster 3 comprised mainly high-dose EMS derived genotypes, with 28.57% of the 80 mM population grouped within this cluster. It was defined by delayed phenological traits, reduced number of locules, and poor yield performanceDelayed phenological traits, fewer locules, and poor yield were characteristic of it. Representative genotypes such as B7, B9, C46, and D20 showed marked negative deviation from the control and were classified as low-value mutants,.

such as B7, B9, C46, and D20, showed a marked negative deviation from the control and were classified as low-value mutants and not considered for advancement.

Cluster 4 contained genotypes that combined early days to first flower (*dff*) and days to 50% flowering (*d50f*) with extended days to first harvest (*dfh*) and days to 50% mature pod (*d50mp*), resulting in high seed weight and elevated yield, particularly within the 20 mM EMS population. This cluster included 10.42%, 9.09%, and 12.50% of individuals from the 20 mM, 40 mM, and 80 mM treatments, respectively, indicating that favorable phenotypes arose across multiple EMS doses. Notable high-performing putative mutants in this cluster included B19 (*93.59 g*) and B33 (*125.44 g*), both of which substantially exceeded the control in yield while maintaining relatively early phenology.

Cluster 5 was composed exclusively of EMS-treated genotypes, representing 33.33%, 22.27%, and 16.07% of the 20 mM, 40 mM, and 80 mM treatment groups, respectively. These individuals exhibited moderately delayed phenological traits and high seed weight, but suffered from a reduced number ofhad fewer locules, resulting in inferior yield performance relative to the control.

Cluster 6 comprised a small but distinct group of EMS-derived genotypes, including 12.50% and 5.36% of the 20 mM and 80 mM populations, respectively. These genotypes were characterized by moderately delayed phenological traits, large seed size, and high seed weight. Despite a relatively low seed number per pod, total yield per plant was high, driven by seed weight. Selected putative mutants from this cluster included B07 and D34, which consistently outperformed the control forin terms of seed weight.

The cluster analysis further allowed us to group the EMS-derived cowpea lines into distinct categories based on overall performance. We identified six clusters, but importantly, not all clusters were equal in agronomic value. The clustering effectively segregated the elite putative mutants those combiningat combine multiple favorable traits from average performers

and poor performers. Cluster membership was closely associated with EMS treatment levels and trait profiles. For example, Cluster 2 in our results consisted predominantly of untreated control plants and a few low-dose mutants, characterized by moderate phenological and yield trait values (baseline yield and phenology). In contrast, another cluster (Cluster 5) contained high-yielding mutants mostly from the 20 mM and 40 mM EMS treatments, featuring significantly high, mainly from the 20 mM and 40 mM EMS treatments, with significantly higher seed weight than the control, underscoring that this group containincluded the desirable phenotypes created by the mutagenesis.

A small cluster (Cluster VI) captured a handful of outlier mutants these included some top-yielding lines with respect to weight as well as unusual genotypes. These included some top-yielding lines suggesting thatfor weight, as well as unusual genotypes, suggesting that, while most of the best mutants grouped together, a few unique lines had singular trait combinations. On the other hand, it was observed a cluster 3a cluster 3 was observed, composed largelymainly composed of 80 mM EMS mutants with severely reduced yields and late maturation. This stratification by clustering aligns with expectations from other studies employing grouping strategies othat employ grouping strategies in mutant populations.

Although formal cluster analysis is not always reported, researchers often categorize mutants by yield or other indices. For instance, [4954] and [5055] in mutation breeding of pulses have discussed grouping mutant lines by overall performance, noting that mutants from optimal dose treatments tend to form a cohort of superior types, whereas those from high doses populate thethe context of mutation breeding of pulses have discussed grouping mutant lines by overall performance, noting that mutants from optimal dose treatments tend to form a cohort of superior types. In contrast, those from high dose treatments tend to form inferior groups. Our results mirror this trend: moderate EMS doses produced the bulk of the high-performance

cluster, while. In contrast, the highest dose contributed disproportionately to the low-performance cluster, consistent with the dose performance relationship reported by [4146].

The clustering outcomes in our study also have practical breeding implications. The fact that the best-yielding mutants clustered together means they share a similar multi-trait profile, which could be harnessed as a selection index. We can infer that these elite lines possess a favorable combination of genes or alleles for yield, likely with minimal negativadverse pleiotropic effects on other traits. This is promising for developing mutant lines, as a high yield was achieved without majorsignificant negative consequences. [4853] selected the top 4% of their M_2 population as high-yield mutants for further breeding. Likewise, our cluster analysis identified 10.71 to 16.67% of lines that significantly exceeded 16.67% of lines that significantly exceeded the others in yield per plant.

By cross comparing our elite cluster with the control cluster, we found that the comparing our elite cluster with the control cluster, we found that improvements in yield traits were statistically significant, reinforcing that these differences are genetically based and not due to random chance. Previous mutation breeding successes in cowpea and related crops have come from recognizing and stabilizing such superior segregants. For example, [5156] developed a high-yielding, large-seeded cowpea mutant variety by isolating an outstanding M_2 plant from a 250 Gy treatment and advancing it through selection. This resonates with our strategy of using multivariate clustering to pinpoint candidates for advancement. Moreover, our cluster analysis revealed that mutants derived from the same EMS dose did not always fall into one cluster indeed, some 20 mM mutants ended up in a poor performance cluster anda single cluster; indeed, some 20 mM mutants ended up in a poor-performing cluster, while some 80 mM mutants grouped with better performers. This indicates that while dose influences overall trends, the phenotypic expression of each mutant line is unique and must be evaluated on its own merit. Therefore, the use of clustering and PCA helps ensure that selection is based

on actual trait patterns rather than preconceived notions of which treatment "should" yield better mutants.

In summary, the elite mutants isolated such as those in high yield related and yield clusters, including those in high-yielding related and yield clusters, will be further evaluated in advanced generations. If their performance is stable, they could serve as new breeding germplasm or even be released as improved cowpea varieties, analogous to the mutant lines released in other legumes such as mungbean and chickpeamutant lines released in other legumes, such as mungbean and chickpea, as noted by [4954].

Conclusion

This study demonstrates the effectiveness of ethyl methanesulfonatemethane sulfonate (EMS) in inducing substantial genetic variability in cowpea (*Vigna unguiculata* (L.) Walp), resulting in a broadenedthereby broadening the phenotypic spectrum for key agronomic, yield, and seed traits. Contrast analysis revealed significant treatment effects and highlighted superior mutant lines, while principal component analysis (PCA) identified yield and reproductive traits as the primary drivers of phenotypic variation. These findings were consistently supported by cluster analysis, which grouped mutants according to overall agronomic performance. The convergence of these analytical approaches strengthened the selection of putative mutants, including high-yielding (B33, B31, B28, B22, C41, C05, D25, D45, D56), early phenology (B19), and large-seeded or prolific podding lines (D34, C39, C40).

As the study was confined to the M_1 generation, further evaluation in the M_2 and M_3 generations is required to confirm trait stability, heritability, and breeding value. Complementary molecular analyses will be essential to explain the genetic basis of the observed variation and support marker-assisted selection. Overall, the selected mutants

constitute valuable genetic resources for cowpea improvement and have strong potential to contribute to enhancedenhance productivity and global food security.

Considering the documented genetic variability of cowpea for nutritional traits such as protein and mineral content, the EMS-induced mutants identified in this study may serve as valuable genetic resources for future breeding programs targeting both yield and nutritional quality improvement .

Acknowledgments

HMK, RANK, IKA, and FO conceived and designed the study. HMK and RANK performed the experiments. HMK analyzed the data and drafted the manuscript. RANK and IKA provided critical revisions. All authors read and approved the final manuscript. We gratefully acknowledge the Department of Plant and Environmental Biology, at the University of Ghana, Legon, for supporting this research.

References

1.

1. Oladosu Y, Rafii MY, Abdullah N, Hussin G, Ramli A, Rahim HA, et al. Principle and application of plant mutagenesis in crop improvement: A review. Biotechnology and Biotechnological Equipment. 2015;30(1):1–16.Oladosu Y, Rafii MY, Abdullah N, Hussin G, Ramli A, Rahim HA, et al. Principle and application of plant mutagenesis in crop improvement: A review. Biotechnology and Biotechnological Equipment. 2015;30(1):1–16.

2. Owusu EY, Karikari B, Kusi F, Haruna M, Amoah RA, Attamah P, et al. Genetic variability, heritability and correlation analysis among maturity and yield traits in Cowpea (Vigna unguiculata (L) Walp) in Northern Ghana. Heliyon. 2021;7(9).

3. Bhat TA, Khan AH, Parveen S. Comparative analysis of meiotic abnormalities induced by gamma rays, EMS and MMS in *Vicia faba* L. Journal of Indian Botanical Society. 2005;84:45–58.

4. Olasupo FO, Ilori CO, Forster BP, Bado S. Mutagenic effects of gamma radiation on eight accessions of cowpea (Vigna unguiculata [L.] Walp.). Am J Plant Sci. 2016;7(2):339–51.

Formatted: List Paragraph, Indent: Left: 0.89", No bullets or numbering, Don't adjust space between Latin and Asian text, Don't adjust space between Asian text and numbers

Formatted: Justified, Indent: Left: 0.44", Don't adjust space between Latin and Asian text, Don't adjust space between Asian text and numbers

Formatted: Font: Font color: Black

Formatted: Left, Indent: Left: 0.44", Adjust space between Latin and Asian text, Adjust space between Asian text and numbers

Formatted: Indent: Left: 0", First line: 0"

5. Hoque ME, Morshad MN. Somaclonal variation in potato (Solanum tuberosum L.) using chemical mutagens. The Agriculturists. 2014;12(1):15–25.

6. Opoku Gyamfi M, Eleblu JSY, Sarfoa LG, Asante IK, Opoku-Agyemang F, Danquah EY. Induced variations of ethyl methane sulfonate mutagenized cowpea (Vigna unguiculata L. Walp) plants. Front Plant Sci. 2022;13:952247.

7. Dhanavel D, Girija M. Effect of EMS, DES and SA on quantitative traits of cowpea (Vigna unguiculata (L.) Walp.) in M1 generation. Crop Research (Hisar). 2009;37(1–3):239–41.

8. Sert H, Ceyhan E. The effects of seed yield and some agricultural characters of different plant density on cowpea (*Vigna sinensis* (L.) Savi) in Hatay ecological conditions. Selcuk Journal of Agriculture and Food Sciences. 2012; *26*(1): 34–43.

Harmankaya M, Ceyhan E, Çelik AS, Sert H, Kahraman A, Özcan MM. Some chemical properties, mineral content and amino acid composition of cowpeas (*Vigna sinensis* (L.) Savi). Quality Assurance and Safety of Crops & Foods. 2016;8(1): 111–116. https://doi.org/10.3920/QAS2014.0487

Jou-Nteufa C, Ceyhan E. Determination of combining ability and heredity through diallel analysis method in F_2 populations of cowpea. Selcuk Journal of Agriculture and Food Sciences. 2022;36(3): 299–311. https://doi.org/10.15316/SJAFS.2022.039

Oladosu Y, Rafii MY, Abdullah N, Hussin G, Ramli A, Rahim HA, et al. Principle and application of plant mutagenesis in crop improvement: A review. Biotechnology and Biotechnological Equipment. 2015;30(1):1–16.

Owusu EY, Karikari B, Kusi F, Haruna M, Amoah RA, Attamah P, et al. Genetic variability, heritability, and correlation analysis of maturity and yield traits in Cowpea (Vigna unguiculata (L.) Walp.) in Northern Ghana. Heliyon. 2021;7(9).

Bhat TA, Khan AH, Parveen S. Comparative analysis of meiotic abnormalities induced by gamma rays, EMS, and MMS in *Vicia faba* L. Journal of Indian Botanical Society. 2005; 84:45–58.

Olasupo FO, Ilori CO, Forster BP, Bado S. Mutagenic effects of gamma radiation on eight accessions of cowpea (*Vigna unguiculata* [L.] Walp.). Am J Plant Sci. 2016;7(2):339–51.

Hoque ME, Morshad MN. Somaclonal variation in potato (Solanum tuberosum L.) using chemical mutagens. The Agriculturists. 2014;12(1):15–25.

Opoku Gyamfi M, Eleblu JSY, Sarfoa LG, Asante IK, Opoku-Agyemang F, Danquah EY. Induced variations of ethyl methane sulfonate mutagenized cowpea (*Vigna unguiculata* L. Walp) plants. Front Plant Sci. 2022; 13:952247.

Dhanavel D, Girija M. Effect of EMS, DES, and SA on quantitative traits of cowpea (*Vigna unguiculata* (L.) Walp.) in M1 generation. Crop Research (Hisar). 2009;37(1–3):239–41.

Jou-Nteufa C, Ceyhan E. Genetic analysis of seed yield and some traits in cowpea using diallel analysis. Turkish Journal of Agriculture and Forestry. 2024;48(3): 278–293. https://doi.org/10.55730/1300-011X.3180

Dalgıç H, Ali A, Karadaş S, Korkmaz A, Ayran Çolak İ, Harmankaya M, Karaköy T, Ceyhan E, Türkoğlu A. (2025). Diallel analysis of cowpea (*Vigna unguiculata* L. Walp) for protein and mineral composition: Genetic variability and heritability. Euphytica. 2025:221: 168. https://doi.org/10.1007/s10681-025-03618-0

Dhanavel D, Girija M. Effect of EMS on induced morphological mutants in cowpea (*Vigna unguiculata* (L.) Walp.). Crop Research. 2009;38:27–31.

Roychowdhury R, Tah J. Assessment of chemical mutagenic effects in mutation breeding programme for M1 generation of carnation (*Dianthus caryophyllus*). Res Plant Biol. 2011;1(4).

Gnankambary TK, Benoit JB, Sawadogo N, Mahamadou S, Tignegre JB, Yonli D, et al. Genetic variability induced by gamma radiation in cowpea (*Vigna unguiculata* L. Walp) in Burkina Faso. Eur Sci J. 2019;15(15):153–64.

Nawale SR, Apte UB, Jadhav BB. Effect of gamma rays and ethyl methane sulfonate on seed germination and survival of seedlings in cowpea (*Vigna unguiculata* (L.) Walp.). Journal of Arid Legumes. 2006;3(1):102–5.

Bind D, Dwivedi VK. Effect of Mutagenesis on Germination, Plant Survival, and Pollen Sterility in M1 Generation of in Cowpea [*Vigna unguiculata* (L.) Walp] [Internet]. Vol. 48, Indian J. Agric. Res. 2014. Available from: www.arccjournals.com

Crino P, Lai A, Bonito RD, Veronese P, Saccardo F. Genetic variability in tomato plants regenerated from irradiated cotyledons. In: Jain SM, Brar DS, Ahloowalia BS, editors. Somaclonal Variation and Induced Mutations in Crop Improvement. Dordrecht: Springer Science & Business Media; 2013.

Jain SM, Brar DS, Ahloowalia BS, editors. Somaclonal Variation and Induced Mutations in Crop Improvement. Dordrecht: Springer Science & Business Media; 2013.

Jain SM, Maluszynski M. In vitro applications in crop improvement: Recent progress. In: Mujib A, Cho M, Predieri S, Banerjee S, editors. Biotechnology for Crop Improvement. New Delhi, India: IBH–Oxford; 2004. p. 169–202.

Jain SM. Mutagenesis in crop improvement under the climate change. Rom Biotechnol Lett. 2010;15(2):88–106.

Kumar A, Rai PK. Induced mutagenesis in mungbean (*Vigna radiata* L.). In: Singh DP, editor. Mutation Breeding in Pulse Crops. Kalyani Publishers; 2006. p. 85–93.

Kundu SK, Singh DP. Gamma ray induced variability for quantitative characters in black gram. Madras Agricultural Journal. 1982;69(10):644–8.

Chandra N. Cytological and mutational studies on Gloriosa superba Linn. [Nagpur, India]: Nagpur University; 1988.

Khan S, Wani MR. Comparison of the effect of chemical mutagens on mung bean. Advances in Plant Sciences. 2005;18(2):533–5.

Khan S, Wani MR. Chemomutagenic effect of two alkylating agents on mung bean (*Vigna radiata*). Bionotes. 2004;6(3):82–3.

Nanda S, Rao GM, Nayak S. Mutagenic effects of EMS on yardlong bean (*Vigna sesquipedalis*). Indian J Agric Res. 1997;31:602–10.

Allard RW. Principles of Plant Breeding. 2nd ed. New York: John Wiley & Sons; 1999.

Falconer DS, Mackay TFC. Introduction to Quantitative Genetics. 4th ed. Harlow: Longman; 1996.

Principles SBDPB, ed. New Delhi: Kalyani Publishers Methods 7th. Singh BD. Plant Breeding: Principles and Methods. 7th ed. New Delhi: Kalyani Publishers; 2006. 2006.

Grafius JE. Components of yield in oats: A geometrical interpretation. Agron J. 1959;51:515–8.

Donald CM. The breeding of crop ideotypes. Euphytica. 1968;17:385–403.

Gaul H. Mutations in plant breeding. Radiation Botany. 1964;4:155–232.

Maluszynski M, Nichterlein K, Van Zanten L, Ahloowalia BS. Officially released mutant varieties The FAO/IAEA database. Mutation Breeding Reviews. 2000;12:1–84.

Goyal P, Khan S, Wani MR. EMS-induced genetic variability for yield and yield components in urdbean (*Vigna mungo* (L.) Hepper). Legume Research. 2021;44:1220–6.

Premsekhar S, Appadurai P. Effect of doses of gamma rays and EMS on induced mutation in pigeonpea. Indian Journal of Agricultural Sciences. 1982;51:381–4.

Singh BD. Plant Breeding: Principles and Methods. 7th ed. New Delhi: Kalyani Publishers; 2006.

Odeigah PGC, Osanyinpeju AO, Myers GO. Induced mutations in cowpea: Morphological and yield traits. Mutation Breeding Newsletter. 1998;44:10–3.

Rai A. Effect of Ethyl Methane Sulfonate Mutagen in M1 Generation in Pigeonpea (*Cajanas Cajan* L. Millasp). Rajmata Vijayaraje Scindia Krishi Vishwa Vidyalaya; 2011.

Khan IA. Mutation studies in mung bean (*Vigna radiata* L. Wilczek): IX. Estimates of genetic variability. Legume Research. 1988;11(2):89–93.

Singh M, Singh VP. Genetic analysis of certain mutant lines of urdbean for yield and quality traits in M4 generation. Indian Journal of Pulses Research. 2001;14(1):60–2.

Jagajanantham N, Dhanavel D, Pavadai P. EMS induced variability in okra (*Abelmoschus esculentus* L.). Int J Sci Res. 2013;2:148–51.

Berenschot AS, Zucchi MI, Tulmann Neto A, Quecini V. Mutagenesis in ornamentals: Application and perspective. Plant Cell Tissue Organ Cult. 2009;98:161–72.

Premsekar R, Appadurai R. EMS induced variability in pigeonpea (*Cajanus cajan* L.). Madras Agricultural Journal. 1982;69:458–66.

Mohanasundaram K, Kalamani A, Ramalingam RS. Induced mutagenesis in cowpea (*Vigna unguiculata* (L.) Walp.). Legume Research. 1999;22:642–8.

Anis M, Sharma Pk, Abbasi N. Gamma rays induced variability in urd bean (*Vigna mungo* (L.) hepper) I M1 generation. Journal of the Indian Botanical Society. 1999;78(1–2):111–3.

Khan SB, Siddiqui A, Nadeem M. Variation in quantitative characters of mung bean after seed treatment with DES. Advances in Plant Sciences. 1994;7(1):41–5.

Kansagra UM, Shukla PT. Variability studies in induced mutants of mungbean. Madras Agricultural Journal. 1993;80(4):180–4.

Iepziba SJ, Subramanian M. Variability in M3 and M4 mutants of urdbean. Indian Journal of Pulses Research. 1993;6(2):197.

Patil BN, Guha VY. Growth attributes in induced mutants of chickpea. PKV Research Journal. 1994;15(2):97–101.

Gnanamurthy S, Dhanavel D. Effect of EMS on induced morphological mutants and chromosomal variation in cowpea (*Vigna unguiculata* (L.) Walp.). International Letters of Natural Sciences. 2014;13:33–43.

Gyamfi MO. Inducing Genetic Variability In Cowpea (*Vigna Unguiculata*, L. Walp Var. Asontem) Using Ethyl Methane Sulfonate A Thesis Presented [Internet]. Available from: http://ugspace.ug.edu.gh

Wani AA. Induced mutagenesis for improvement of pulse crops. Plant Breed Rev. 2017;41:155–200.

- Goyal S, Wani MR, Laskar RA, Raina A, Amin R, Khan S. Induction of morphological mutations and mutant phenotyping in black gram [*Vigna mungo* (L.) Hepper] using gamma rays and EMS. Vegetos. 2019;32(4):464–72.
- Pandey B, Dhanasekar P. Development of high-yielding mutant varieties in cowpea. Indian Journal of Genetics. 2003;63:151–9.
- Kumar Y, Mishra VK. Effect of gamma rays and diethyl sulphate on germination,growth, fertility and yield in greengram (*Vigna radiata* L. Wilczek). 1999.

Dhanavel D, Girija M. Effect of EMS on induced morphological mutants in cowpea (Vigna unguiculata (L.) Walp.). Crop Research. 2009;38:27–31.

9. Roychowdhury R, Tah J. Assessment of chemical mutagenic effects in mutation breeding programme for M1 generation of carnation (Dianthus caryophyllus). Res Plant Biol. 2011;1(4).

10. Gnankambary TK, Benoit JB, Sawadogo N, Mahamadou S, Tignegre JB, Yonli D, et al. Genetic variability induced by gamma radiation in cowpea (Vigna unguiculata L. Walp) in Burkina Faso. Eur Sci J. 2019;15(15):153–64.

11. Nawale SR, Apte UB, Jadhav BB. Effect of gamma rays and ethyl methane sulfonate on seed germination and survival of seedlings in cowpea (Vigna unguiculata (L.) Walp.). Journal of Arid Legumes. 2006;3(1):102–5.

12. Bind D, Dwivedi VK. EFFECT OF MUTAGENESIS ON GERMINATION, PLANT SURVIVAL AND POLLEN STERILITY IN M 1 GENERATION OF IN COWPEA [VIGNA UNGUICULATA (L.) WALP] [Internet]. Vol. 48, Indian J. Agric. Res. 2014. Available from: www.arccjournals.com

13. Crino P, Lai A, Bonito RD, Veronese P, Saccardo F. Genetic variability in tomato plants regenerated from irradiated cotyledons. In: Jain SM, Brar DS, Ahloowalia BS, editors. Somaclonal Variation and Induced Mutations in Crop Improvement. Dordrecht: Springer Science & Business Media; 2013.

14. Jain SM, Brar DS, Ahloowalia BS, editors. Somaclonal Variation and Induced Mutations in Crop Improvement. Dordrecht: Springer Science & Business Media; 2013.

15. Jain SM, Maluszynski M. In vitro applications in crop improvement: Recent progress. In: Mujib A, Cho M, Predieri S, Banerjee S, editors. Biotechnology for Crop Improvement. New Delhi, India: IBH–Oxford; 2004. p. 169–202.

16. Jain SM. Mutagenesis in crop improvement under the climate change. Rom Biotechnol Lett. 2010;15(2):88–106.

17. Kumar A, Rai PK. Induced mutagenesis in mungbean (Vigna radiata L.). In: Singh DP, editor. Mutation Breeding in Pulse Crops. Kalyani Publishers; 2006. p. 85–93.

18. Kundu SK, Singh DP. Gamma-ray induced variability for quantitative characters in black gram. Madras Agricultural Journal. 1982;69(10):644–8.

19. Chandra N. Cytological and mutational studies on Gloriosa superba Linn. [Nagpur, India]: Nagpur University; 1988.

20. Khan S, Wani MR. Comparison of the effect of chemical mutagens on mung bean. Advances in Plant Sciences. 2005;18(2):533–5.

21. Khan S, Wani MR. Chemomutagenic effect of two alkylating agents on mung bean (Vigna radiata). Bionotes. 2004;6(3):82–3.

22. Nanda S, Rao GM, Nayak S. Mutagenic effects of EMS on yardlong bean (Vigna sesquipedalis). Indian J Agric Res. 1997;31:602–10.

23. Allard RW. Principles of Plant Breeding. 2nd ed. New York: John Wiley & Sons; 1999.

24. Falconer DS, Mackay TFC. Introduction to Quantitative Genetics. 4th ed. Harlow: Longman; 1996.

25. Principles SBDPB, ed. New Delhi: Kalyani Publishers Methods 7th. Singh BD. Plant Breeding: Principles and Methods. 7th ed. New Delhi: Kalyani Publishers; 2006. 2006.

26. Grafius JE. Components of yield in oats: A geometrical interpretation. Agron J. 1959;51:515–8.

27. Donald CM. The breeding of crop ideotypes. Euphytica. 1968;17:385–403.

28. Gaul H. Mutations in plant breeding. Radiation Botany. 1964;4:155–232.

29. Maluszynski M, Nichterlein K, Van Zanten L, Ahloowalia BS. Officially released mutant varieties – The FAO/IAEA database. Mutation Breeding Reviews. 2000;12:1–84.

30. Goyal P, Khan S, Wani MR. EMS-induced genetic variability for yield and yield components in urdbean (Vigna mungo (L.) Hepper). Legume Research. 2021;44:1220–6.

31. Premsekhar S, Appadurai P. Effect of doses of gamma rays and EMS on induced mutation in pigeonpea. Indian Journal of Agricultural Sciences. 1982;51:381–4.

32. Singh BD. Plant Breeding: Principles and Methods. 7th ed. New Delhi: Kalyani Publishers; 2006.

33. Odeigah PGC, Osanyinpeju AO, Myers GO. Induced mutations in cowpea: Morphological and yield traits. Mutation Breeding Newsletter. 1998;44:10–3.

34. Rai A. Effect of Ethyl Methane Sulfonate Mutagen in M1 Generation in Pigeonpea (Cajanas Cajan L. Millasp). Rajmata Vijayaraje Scindia Krishi Vishwa Vidyalaya; 2011.

35. Khan IA. Mutation studies in mung bean (Vigna radiata L. Wilczek): IX. Estimates of genetic variability. Legume Research. 1988;11(2):89–93.

36. Singh M, Singh VP. Genetic analysis of certain mutant lines of urdbean for yield and quality traits in M4 generation. Indian Journal of Pulses Research. 2001;14(1):60–2.

37. Jagajanantham N, Dhanavel D, Pavadai P. EMS-induced variability in okra (Abelmoschus esculentus L.). Int J Sci Res. 2013;2:148–51.

38. Berenschot AS, Zucchi MI, Tulmann Neto A, Quecini V. Mutagenesis in ornamentals: Application and perspective. Plant Cell Tissue Organ Cult. 2009;98:161–72.

39. Premsekar R, Appadurai R. EMS-induced variability in pigeonpea (Cajanus cajan L.). Madras Agricultural Journal. 1982;69:458–66.

40. Mohanasundaram K, Kalamani A, Ramalingam RS. Induced mutagenesis in cowpea (Vigna unguiculata (L.) Walp.). Legume Research. 1999;22:642–8.

41. Anis M, Sharma Pk, Abbasi N. Gamma rays induced variability in urd bean (Vigna mungo (L.) hepper) I M1 generation. Journal of the Indian Botanical Society. 1999;78(1–2):111–3.

42. Khan SB, Siddiqui A, Nadeem M. Variation in quantitative characters of mung bean after seed treatment with DES. Advances in Plant Sciences. 1994;7(1):41–5.

43. Kansagra UM, Shukla PT. Variability studies in induced mutants of mungbean. Madras Agricultural Journal. 1993;80(4):180–4.

44. Iepziba SJ, Subramanian M. Variability in M3 and M4 mutants of urdbean. Indian Journal of Pulses Research. 1993;6(2):197.

45. Patil BN, Guha VY. Growth attributes in induced mutants of chickpea. PKV Research Journal. 1994;15(2):97–101.

46. Gnanamurthy S, Dhanavel D. Effect of EMS on induced morphological mutants and chromosomal variation in cowpea (Vigna unguiculata (L.) Walp.). International Letters of Natural Sciences. 2014;13:33–43.

47. Gyamfi MO. Inducing Genetic Variability In Cowpea (Vigna Unguiculata, L. Walp Var. Asontem) Using Ethyl Methane Sulfonate A Thesis Presented [Internet]. Available from: http://ugspace.ug.edu.gh

48. Wani AA. Induced mutagenesis for improvement of pulse crops. Plant Breed Rev. 2017;41:155–200.

49. Goyal S, Wani MR, Laskar RA, Raina A, Amin R, Khan S. Induction of morphological mutations and mutant phenotyping in black gram [Vigna mungo (L.) Hepper] using gamma rays and EMS. Vegetos. 2019;32(4):464–72.

50. Pandey B, Dhanasekar P. Development of high-yielding mutant varieties in cowpea. Indian Journal of Genetics. 2003;63:151–9.

51. Kumar Y, Mishra VK. Effect of gamma rays and diethyl sulphate on germination, growth, fertility and yield in greengram (Vigna radiata L. Wilczek). 1999.

Formatted: Font: Font color: Black

Formatted: Indent: Left: 0", Hanging: 0.44"

Supporting Information

S1: Trait dataset and cluster grouping of individual M1 cowpea plants across EMS treatments

Trt	ID	*dtg*	*dff*	*d50f*	*dfh*	*d50mp*	*pdl*	*ln*	*sdl*	*sdw*	*sdt*	*swg*	*yld*	Cluster group
0mM	A01	4	40	60	67	89	12.94	13	8.94	5.6	4.3	23.2	55.68	1
	A02	4	39	60	53	88	17	18	6.62	4.53	3.86	20.34	42.71	2
	A03	3	40	56	49	87	13.24	21	8.98	5.4	4.13	17.94	43.05	2
	A04	3	35	52	50	81	13.2	13	7.03	4.35	3.29	23.96	52.71	2
	A05	3	43	66	58	93	14.24	18	7.22	4.7	4.02	20.08	43.38	2
	A06	3	30	60	60	78	13.58	21	6.72	4.42	3.7	20.97	41.53	2
	A07	4	40	55	47	75	13.8	12	9.26	5.71	4.19	24.87	34.82	3
	A08	4	41	62	53	83	11.55	19	7.91	5.11	4.17	19.93	36.27	2
	A09	4	37	52	41	67	15.4	14	6.16	4.44	4.41	20.92	72.81	2
	A10	4	37	54	58	89	13.09	16	6.23	4.15	3.64	22.13	44.92	2
	A11	4	40	62	45	74	15.29	18	7.37	4.84	4.29	21.1	31.66	2
	A12	4	44	59	46	76	15.95	17	8.98	5.4	4.13	21.01	43.71	3
	A13	3	43	60	49	87	17.58	16	6.99	4.46	3.81	17.82	42.78	2
	A14	5	30	52	53	80	16.64	18	9.65	5.67	4.28	23.13	73.78	3
	A15	4	39	54	47	80	15.57	17	8.24	5.25	4.13	19.75	40.09	3
	A16	3	45	66	40	70	14.08	24	6.77	4.57	4.36	20.79	39.49	2
	A17	4	31	54	43	66	17.21	12	6.74	4.34	3.42	20.36	61.08	2
	A18	3	32	49	56	94	14.25	20	7.57	5.03	4.92	22.64	67.93	4
	A19	2	35	59	42	72	15.3	13	6.21	4.46	4.43	20.61	54.41	2
	A20	3	33	48	47	84	16.62	20	6.38	4.3	4.1	22.57	72.66	4
	A21	4	40	57	44	71	13.33	17	6.43	4.47	4.19	18.36	50.5	2
	A24	4	39	58	49	72	15.42	17	5.68	3.94	3.63	19.63	61.83	2
	A25	3	39	59	50	70	12.83	21	6.24	4.19	3.71	24.67	51.81	2
	A26	3	37	59	54	86	15.3	23	6.76	4.54	4.53	22.04	52.89	4
	A27	4	32	53	53	84	16	16	11.59	6.08	4.82	19.21	22.86	3
	A28	4	40	65	43	79	11.88	12	6.07	4.03	3.85	21.97	85.68	2
	A29	2	39	57	44	81	14.92	17	8.49	5.13	3.92	23.24	23.24	3
	A30	4	43	68	51	88	13.53	18	8.35	5.15	4.48	18.63	50.31	2
	A31	3	40	64	40	69	14.9	13	6.61	4.53	4.52	18.39	74.66	2
20mM	B01	5	50	70	68	92	12.13	18	6.33	4.24	4.06	22.28	55.69	5
	B02	4	46	66	59	98	13.23	21	6.65	4.38	3.66	26.08	28.69	5
	B03	5	42	60	53	79	14.98	13	6.94	4.58	4.23	25.41	37.36	5
	B04	6	43	60	60	100	16.28	18	9.26	5.71	4.19	25.92	54.44	1
	B05	5	47	66	62	82	13.8	21	10.1	5.78	4.92	25.17	49.83	1
	B06	5	51	75	54	76	15.75	12	10.35	5.76	4.91	25.9	62.15	6
	B07	7	38	60	63	89	11.45	19	11.65	6.44	5.19	29.99	67.18	6
	B08	5	49	74	68	102	15.25	14	9.85	5.67	5.12	22.68	41.27	1
	B09	7	52	74	71	98	13	21	11.59	6.08	4.82	27.48	74.2	6
	B10	6	47	63	56	81	14.31	23	7.03	4.48	3.79	20.06	46.14	2
	B11	7	52	70	60	95	13.3	16	9.75	5.66	4.99	26.1	26.1	3
	B12	3	41	59	49	87	17.7	13	10.4	5.9	5.16	26.32	48.7	6
	B13	4	47	62	66	100	14.25	18	9.9	5.72	4.87	23.49	59.2	1
	B14	7	53	69	58	87	14.5	21	7.18	4.88	4.76	24.86	45.75	5
	B15	3	54	71	55	76	15.25	16	9.75	5.88	4.83	27.77	79.15	1
	B16	5	53	75	64	102	14.7	18	9.75	5.68	4.85	26.24	75.56	1
	B17	3	41	65	71	93	13.7	17	10.4	5.82	4.84	27.34	71.37	6
	B18	6	54	71	62	98	15.5	12	10	5.62	4.89	28.33	45.32	3
	B19	5	36	51	48	68	19.88	19	6.23	4.17	4	24.82	93.59	4
	B20	5	34	57	52	72	14.7	14	6.77	4.57	4.36	26.63	42.6	5

Values highlighted red indicate trait means significantly (P<0.05) below the control, while green indicate values significantly (P<0.05) above the control.Key: Trt= Treatment ID=Genotype Identifier, dtg days to germination, dff = days to first flower, d50f = days to 50% flowering, dfh = days to first harvest, d50mp = days to 50% mature pods, pdl = pod length, pdw = pod width, ln = number of locules, nsp = number of seeds per pod, psa = percent seed abortion, sdl = seed length, sdw = seed width, sdt = seed thickness, swgt = seed weight, yld = yield per plant

S1 Table: Trait dataset and cluster grouping of individual M_1 cowpea plants across EMS treatments (*cont'd*)

Trt	ID	*dtg*	*dff*	*d50f*	*dfh*	*d50mp*	*pdl*	*ln*	*sdl*	*sdw*	*sdt*	*swg*	*yld*	Cluster group
20mM	B21	5	47	63	70	91	13.6	13	9.85	5.64	5.09	22.6	81.37	1
	B22	6	36	56	70	97	12.1	20	7.04	4.98	4.85	21.91	88.96	1
	B23	7	48	73	55	79	13.04	17	8.26	5.04	3.93	22.04	46.28	5
	B24	6	50	74	52	75	13.35	13	8.7	5.13	4.91	17.81	18.7	3
	B25	7	39	61	58	89	17.75	18	9.65	5.31	4.52	25.92	76.99	1
	B26	4	47	72	64	100	14	21	10.1	5.54	4.87	24.26	91.45	1
	B27	7	39	55	56	85	16.1	12	7.04	4.68	4.28	25.59	67.56	4
	B28	4	39	60	67	87	12.13	19	7.3	4.75	4.04	23	103.5	1
	B29	6	43	66	61	94	13.27	14	6.62	4.53	3.86	24.2	45.73	5
	B30	7	40	61	63	95	14.1	13	6.46	4.29	4.1	23.82	28.58	5
	B31	3	39	60	58	98	14.85	20	9.9	5.46	5	24.65	107.22	1
	B32	6	47	71	49	85	14.22	17	6.14	4.06	3.87	22.04	66.11	4
	B33	6	37	61	56	85	16.33	19	6.1	4.05	3.31	27.88	125.44	4
	B34	7	41	57	53	85	12.44	17	7.04	4.98	4.85	22.2	38.86	5
	B35	7	51	68	56	91	14.75	13	10.05	5.24	3.56	26.06	63.85	5
	B36	4	33	54	64	100	14.1	19	10.15	5.18	3.91	23.62	64.48	1
	B37	7	53	73	62	82	14.35	17	11.25	5.77	3.71	26.15	82.38	1
	B38	5	43	61	61	81	14	13	10.3	5.01	3.84	27.88	42.94	5
	B39	6	54	71	49	85	13.25	19	10.4	5.67	4.46	26.71	86.53	1
	B40	7	37	58	61	90	16.6	15	10.2	5.5	4.4	26.06	68.8	6
	B41	7	33	50	60	99	17.52	18	6.43	4.47	4.19	25.04	66.61	4
	B42	5	49	74	69	107	12.7	20	6.74	4.34	3.42	26.21	46.13	5
	B43	3	37	56	58	96	12.25	18	9.3	4.73	3.72	23.01	68.33	5
	B44	6	48	70	69	107	14.43	19	6.65	4.66	4.27	21.38	29.94	5
	B45	3	48	67	62	96	13.75	20	10.1	5.57	4.08	23.17	76.45	1
	B46	6	46	65	55	80	10.5	18	9.5	4.7	3.72	27.56	46.86	5
	B47	4	52	70	59	89	14.5	19	10.15	5.15	3.97	24.42	72.54	1
	B48	4	39	59	72	105	14.5	14	9.65	5.36	3.86	25.18	30.22	5
40mM	C01	4	49	68	67	103	13.95	21	10	4.95	3.8	23.44	50.63	1
	C02	3	43	59	38	68	14.05	21	9.25	4.69	3.71	17.7	77.01	2
	C03	4	52	67	61	85	11.75	12	9.6	4.67	3.43	25.95	61.76	1
	C04	4	40	55	50	76	11.5	17	10	5.5	4.18	25.96	68.52	1
	C05	7	47	67	62	91	14.2	11	9.55	5.61	4.29	26.03	105.43	1
	C06	7	45	70	66	92	11.95	12	9.85	4.84	3.68	25.34	44.6	5
	C07	3	35	60	60	89	11.75	17	9.95	5.49	4.2	28.39	78.35	1
	C08	4	46	62	67	106	14	11	9.95	5.48	4.09	23.9	55.22	1
	C09	3	46	71	61	98	13.15	11	9.85	5.27	3.95	27.53	60.57	5
	C10	4	55	76	68	97	16.75	12	9.75	5.09	4	25.64	57.43	1
	C11	5	38	54	58	96	11	14	9.2	5.13	4.05	23.3	39.61	5
	C12	6	44	59	67	98	13.6	18	9.85	5.56	3.97	26.7	82.24	1
	C13	6	45	67	65	93	13.6	18	8.1	4.72	3.82	26.98	87.43	1
	C14	3	43	68	39	74	14.95	17	9.4	4.63	3.49	17.77	52.79	2
	C15	3	50	71	66	88	15.6	19	10	5.18	4.29	27.88	57.98	1
	C16	7	52	69	55	80	12.5	17	9.6	5.14	3.98	27.98	76.93	1
	C17	7	45	64	55	95	16.65	13	10.45	5.01	3.51	20.9	68.96	4
	C18	5	53	78	66	91	12.6	19	9.65	4.87	3.99	26.66	72.78	1
	C19	4	52	72	65	88	14.8	17	9.85	5.25	3.83	26.46	47.62	5
	C20	4	46	71	47	83	13.7	13	9.3	4.79	3.51	21.5	54.19	5
	C21	3	53	73	58	90	10.5	19	9	5.02	4.06	25.8	59.33	1

Values highlighted red indicate trait means significantly ($P<0.05$) below the control, while green indicate values significantly ($P<0.05$) above the control.Key: Trt= Treatment ID=Genotype Identifier, dtg days to germination, dff = days to first flower, d50f = days to 50% flowering, dfh = days to first harvest, d50mp = days to 50% mature pods, pdl = pod length, pdw = pod width, ln = number of locules, nsp = number of seeds per pod, psa = percent seed abortion, sdl = seed length, sdw = seed width, sdt = seed thickness, swgt = seed weight, yld = yield per plant

S2 Table: Trait dataset and cluster grouping of individual M_1 cowpea plants across EMS treatments *(cont'd)*

Trt	ID	*dtg*	*dff*	*d50f*	*dfh*	*d50mp*	*pdl*	*ln*	*sdl*	*sdw*	*sdt*	*swg*	*yld*	Cluster group
40mM	C22	5	55	73	51	78	14.9	15	10	5.5	4.32	25.76	27.04	3
	C23	5	43	64	55	81	12.05	18	9.6	4.79	3.84	21.7	42.96	1
	C24	4	43	65	56	76	13.5	11	9.8	5.31	3.85	26.02	94.7	1
	C25	4	40	59	62	102	12.5	12	9.4	4.81	3.63	23.51	55.96	5
	C26	7	51	70	54	88	11.95	14	9.85	5.25	3.77	23.6	69.4	1
	C27	5	48	69	67	95	6.95	19	9	4.58	5.59	23.01	48.33	1
	C28	7	49	72	67	91	15.95	17	10	5.33	3.86	26.46	47.62	5
	C29	5	39	62	56	92	15.4	13	10	4.98	3.81	25.45	42.76	5
	C30	3	37	57	62	93	14.9	20	10	5.58	3.98	21.34	59.76	1
	C31	5	42	59	50	83	12.75	18	10	4.88	3.57	27.56	48.24	5
	C32	7	34	49	59	84	14.95	19	9.85	4.91	3.87	27.16	62.74	4
	C33	3	52	77	50	76	12.25	19	9.35	5.33	4.21	24.58	63.91	1
	C34	3	44	68	57	93	15.75	15	8.65	5.03	4.35	24.24	56.71	4
	C35	5	48	70	58	94	15.45	18	9.85	4.88	4.01	23.82	60.04	1
	C36	3	54	74	54	83	13.95	12	10.05	4.78	3.63	24.73	61.83	1
	C37	4	49	67	51	76	13.9	17	9.8	5.28	4.08	23.83	31.45	1
	C38	6	34	59	62	89	8.77	11	8.95	5.06	3.81	19.95	39.5	5
	C39	6	37	57	52	92	15.5	19	10.1	4.98	3.91	22.71	27.02	5
	C40	6	35	58	72	98	13.5	17	10	5.4	3.68	24.03	28.83	5
	C41	5	55	80	64	89	14.5	13	10	4.83	3.71	26.36	90.96	1
	C42	7	36	59	58	95	13.35	19	10	5.59	4.49	25.19	25.19	5
	C43	5	35	53	71	95	14	15	10	5.55	4.45	27.28	90.01	1
	C44	5	41	57	55	95	15.5	18	10	4.97	4.14	25.65	76.17	1
	C45	3	40	59	54	84	15.75	12	11.05	5.07	3.8	27.77	83.32	1
	C46	5	52	71	64	103	16.15	17	10.9	5.69	4.25	24.74	59.87	1
	C47	4	40	57	63	87	13.95	11	10	5.58	4.09	23.3	37.51	3
	C48	4	50	73	62	92	16.75	13	10	5.19	3.99	19.97	19.97	3
	C49	7	39	55	59	80	14.75	8	10.4	5.29	3.92	22.82	60.26	3
	C50	3	51	69	62	84	14.5	9	10.1	5.39	3.93	23.53	54.59	5
	C51	4	53	70	62	87	12.5	13	9.9	5.3	3.96	19.66	21.63	3
	C52	4	39	60	64	86	15.9	8	9.9	5.17	4.02	23.13	60.13	4
	C53	6	39	63	62	85	14.75	9	9.75	5.22	3.9	19.28	75.19	4
	C54	5	41	59	54	80	16.55	12	10.15	5.58	3.55	25.23	55.51	3
	C55	7	53	70	51	89	14.5	11	9.85	4.76	3.69	25.06	52.62	5
80mM	D01	7	49	69	49	80	13.5	17	10	4.94	3.95	22.58	31.17	3
	D02	6	50	72	54	92	10.75	10	10.7	5.71	4.28	24.18	42.55	1
	D03	3	40	58	70	93	13.5	9	9.85	5.61	4.56	24.13	74.33	1
	D04	5	55	73	72	100	12.5	13	9.35	5.34	3.81	18.82	76.2	1
	D05	7	35	53	59	96	11.75	10	9.95	5.69	4.18	22.05	45.64	5
	D06	4	34	59	55	93	15	9	10.3	5.68	4.16	20.79	50.32	3
	D07	4	46	70	49	83	12.5	13	9.7	5.44	3.91	20.34	31.72	5
	D08	4	48	69	61	81	15.25	13	10.55	6.11	4.94	26.24	43.3	3
	D09	5	47	65	66	100	15	9	10.3	5.21	4.28	27.62	39.78	1
	D10	4	53	78	58	97	13.75	10	9.85	5.04	4.38	24.53	35.57	3
	D11	7	46	62	61	81	14	12	9.95	5.55	4.91	22.22	34	1
	D12	3	43	68	59	95	15.75	9	10.05	5.58	4.7	22.36	60.82	6

Values highlighted red indicate trait means significantly (P<0.05) below the control, while green indicate values significantly (P<0.05) above the control.Key: Trt= Treatment ID=Genotype Identifier, dtg days to germination, dff = days to first flower, d50f = days to 50% flowering, dfh = days to first harvest, d50mp = days to 50% mature pods, pel = peduncle length at first harvest, npp = number of pods per peduncle, nptm = number of pods per plant at maturity, nspt = number of seeds per plant, pdl = pod length, pdw = pod width, ln = number of locules, nsp = number of seeds per pod, psa = percent seed abortion, sdl = seed length, sdw = seed width, sdt = seed thickness, swgt = seed weight, yld = yield per plant

S1 Table: Trait dataset and cluster grouping of individual M_1 cowpea plants across EMS treatments *(cont'd)*

Trt	ID	*dtg*	*dff*	*d50f*	*dfh*	*d50mp*	*pdl*	*ln*	*sdl*	*sdw*	*sdt*	*swg*	*yld*	Cluster group
80mM	D13	6	42	59	69	91	16.5	13	10.05	5.2	4.67	27	64.8	1
80mM	D14	6	52	74	45	81	12.75	12	9.4	5.37	4.46	24.66	54.24	1
80mM	D15	4	43	68	61	92	14.5	9	10.5	5.47	4	21.45	36.03	3
80mM	D16	5	48	68	65	92	13	13	9.9	5.02	3.95	22.74	68.23	1
80mM	D17	6	40	56	64	86	12.75	11	9.4	5.91	4.89	23.07	49.84	6
80mM	D18	3	44	68	64	101	11.45	7	9.95	4.94	3.97	25.55	35.77	1
80mM	D19	5	43	58	59	91	14.5	12	11	5.24	3.41	23.94	55.07	5
80mM	D20	4	55	75	64	103	11.25	13	10.75	5.41	4.21	22.04	65.9	1
80mM	D21	3	37	59	59	97	17.5	8	11	5.35	3.74	24.48	42.83	3
80mM	D22	7	47	62	63	92	14.5	14	9.35	4.73	3.74	20.59	40.77	4
80mM	D23	7	37	55	57	78	16	13	9.35	5.21	3.94	22.7	25.88	3
80mM	D24	4	40	62	72	109	14.25	8	9.15	4.95	4.07	17.35	58.65	4
80mM	D25	3	45	68	68	90	15	9	11	5.62	4.15	24.44	102.66	1
80mM	D26	4	46	63	71	110	17.1	13	8.95	5.02	3.86	22.7	22.7	3
80mM	D27	4	44	62	61	86	16.25	8	10	5.31	4.04	21.91	42.73	4
80mM	D28	6	43	66	60	92	17.1	9	10.45	5.48	4.28	22.32	75	4
80mM	D29	7	39	62	58	82	12.75	12	7.95	4.28	3.45	21.58	69.93	4
80mM	D30	4	35	60	56	84	12.75	11	10.25	5.02	3.79	25.06	36.08	5
80mM	D31	4	35	55	54	87	14.9	17	10.75	5.17	4.02	21.98	24.17	5
80mM	D32	4	44	62	68	96	14.15	11	10.2	6.05	4.82	22.16	37.23	1
80mM	D33	4	37	60	48	85	11.25	7	10.3	4.81	3.92	25.86	46.54	5
80mM	D34	4	45	66	52	83	12.25	12	10.25	4.97	3.46	21.21	23.75	3
80mM	D35	7	41	66	58	81	16.75	13	9.3	5.09	4.06	21.79	35.96	3
80mM	D36	3	53	71	49	73	14	8	11	5.64	4.37	28.29	74.69	6
80mM	D37	4	43	61	59	80	15.25	9	10	5.38	4.08	25.05	50.86	5
80mM	D38	5	42	57	62	88	15.5	10	9.3	5.14	3.5	17.49	44.24	4
80mM	D39	4	40	56	52	90	15.75	9	9.4	5.3	4.06	19.27	48.57	3
80mM	D40	4	44	69	54	77	9.95	13	8.55	5.18	3.96	19.42	55.55	2
80mM	D41	7	45	63	59	81	15	12	9	4.79	3.75	19.76	80.03	4
80mM	D42	3	55	80	66	96	9.95	11	9.9	4.72	3.72	22.02	29.06	3
80mM	D43	3	54	73	48	69	11.7	17	10.5	4.9	3.67	21.98	42.19	1
80mM	D44	6	48	65	52	86	9.5	12	11.6	6.03	4.46	24.35	64.29	1
80mM	D45	4	47	68	60	92	11.5	9	9.8	5.4	4.22	23.58	106.09	1
80mM	D46	3	44	62	57	78	12.5	13	9.8	5.05	4.15	21.53	45.21	3
80mM	D47	3	38	59	58	78	13	13	9.85	5.08	3.99	18.45	18.45	3
80mM	D48	5	47	65	52	84	13.65	8	10	5.3	4.24	22.52	85.13	1
80mM	D49	7	52	72	62	83	11.2	9	10.45	5.48	4.03	22.93	79.1	1
80mM	D50	3	42	60	52	92	13.1	12	9.65	4.82	3.7	22.5	43.19	5
80mM	D51	7	33	56	50	83	8.45	11	9.55	4.76	3.84	22.62	64.7	5
80mM	D52	5	55	77	56	84	14.2	17	10.4	4.87	3.87	19.54	22.48	3
80mM	D53	7	43	62	59	99	11.4	12	9.8	5.51	4.38	22.83	55.25	1
80mM	D54	5	47	71	60	85	11.5	9	9.8	5.44	4.1	20.24	28.33	3
80mM	D55	4	37	62	43	74	14.25	13	9.05	4.85	3.85	20.31	35.34	2
80mM	D56	5	53	69	57	94	13.5	23	10.15	5.03	3.78	28.68	111.85	1

Values highlighted red indicate trait means significantly ($P<0.05$) below the control, while green indicate values significantly ($P<0.05$) above the control.Key: Trt= Treatment ID=Genotype Identifier, dtg = days to germination, dff = days to first flower, d50f = days to 50% flowering, dfh = days to first harvest, d50mp = days to 50% mature pods, pdl = pod length, pdw = pod width, ln = number of locules, nsp = number of seeds per pod, psa = percent seed abortion, sdl = seed length, sdw = seed width, sdt = seed thickness, swgt = seed weight, yld = yield per plant

S2 Table: PCA loadings and variance explained for traits in M_1 cowpea

Traits	PC1	PC2	PC3	PC4	PC5	PC6	PC7	PC8	Uniqueness
sdl	0.73								0.18
sdw	0.70		0.50						0.11
dfh	0.68			0.50				-0.32	0.14
dff	0.61		-0.64						0.10
d50mp	0.59			0.51				-0.40	0.13
d50f	0.57		-0.68						0.09
swg	0.53				0.35			0.43	0.36
sdt	0.38		0.42			0.52	-0.38		0.24
nspt		0.96							0.02
yld		0.95							0.02
nsp		0.75		0.36	-0.38		-0.32		0.03
nptm		0.32		-0.66			0.53		0.04
pdl			0.42						0.77
pdw					0.54	-0.35		0.32	0.40
ln					0.47	0.63			0.22
dtg					0.46	-0.33			0.51
psa					-0.42			0.32	0.52
npp						0.32	0.41	0.43	0.43
**Eigenvalue**	**3.18**	**2.71**	**1.70**	**1.51**	**1.34**	**1.17**	**1.07**	**1.03**	-
**Proportion %**	**17.70**	**15.10**	**9.40**	**8.40**	**7.50**	**6.50**	**6.00**	**5.70**	
**Cumulative %**	**17.70**	**32.70**	**42.20**	**50.60**	**58.00**	**64.50**	**70.50**	**76.20**	-

PC= principal component, dtg = days to germination, dff = days to first flower, d50f = days to 50% flowering, dfh = days to first harvest, d50mp = days to 50% mature pods, npp = number of pods per peduncle, nptm = number of pods per plant at maturity, nspt = number of seeds per plant, pdl = pod length, pdw = pod width, ln = number of locules, nsp = number of seeds per pod, psa = percent seed abortion, sdl = seed length, sdw = seed width, sdt = seed thickness, swgt = seed weight, yld = yield per plant

HENRY KOFI MENSAH
Department of Plant and Environmental Biology
University of Ghana
P. O. Box LG 55, Legon–Accra, Ghana
Email: henryquofi@gmail.com
Tel: +1 437 383 2410

09th April 2026

The Academic Editor and Reviewers
PLOS ONE

Dear Academic Editor and Reviewers,

Response to Reviewers – Manuscript ID: PONE-D-26-01099

We sincerely thank the academic editor and reviewers for the careful and constructive evaluation of our manuscript. We have carefully considered all comments raised and have addressed each one fully in the revised manuscript. The specific revisions made in response to each comment are detailed in the accompanying point-by-point response letter. We are confident that these changes have substantially improved the quality, clarity and depth of the manuscript, and we hope the revised submission meets the standards of PLOS ONE.

Sincerely yours,

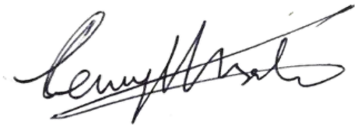

Henry Kofi Mensah (Corresponding author)
On behalf of all co-authors

RESPONSE TO EDITOR AND REVIEWERS

Reviewer #1 Comment 1:
The rationale for selecting the EMS concentrations and the fixed exposure duration should be clarified in relation to previous studies, and the unexpectedly lower germination and survival rates observed in the control compared with EMS treatments should be discussed more explicitly to avoid potential misinterpretation.

Response:
We thank the reviewer for raising both points.
Regarding EMS concentrations and exposure duration: We have now clarified in the revised manuscript that 20-, 40-, and 80-mM EMS concentrations with a fixed 6-hour exposure were selected based on prior mutagenesis studies in cowpea and closely related legumes that reported effective mutation induction at similar concentration ranges and exposure durations. This selection ensured biological comparability with published literature while covering a dose range sufficient to produce measurable variation in phenological, yield-related and yield traits.

Regarding the lower germination and survival in the control: We agree that this result required explicit discussion to prevent misinterpretation. We now attribute the lower germination and survival in the 0 mM control group primarily to variability in seed physiological status specifically, pre-existing dormancy or uneven seed viability within the control lot, which received no pre-treatment. It is additionally plausible that low-dose EMS exposure elicited a mild hormetic or priming-like response, stimulating metabolic activation in treated seeds at sub-lethal concentrations, a phenomenon that has been reported in leguminous crops subjected to sub-lethal chemical mutagen doses. Both explanations have been incorporated into the revised discussion section to prevent misinterpretation.

Reviewer #1 Comment 2:
Sample sizes should be reported consistently across all traits.

Response:
We agree with this observation. The revised manuscript now consistently reports sample sizes for each treatment group across all traits. Treatment-wise sample sizes, N = 31 (0 mM), 48 (20 mM), 55 (40 mM), and 56 (80 mM) are now stated in all relevant tables and sections.

Reviewer #1 Comment 3:
The presentation of statistical significance in tables should be standardized for clarity.

Response:
We thank the reviewer for this observation. All tables in the revised manuscript have been reviewed and standardized to present statistical significance consistently. Significance codes (‘***’ for $p \leq 0.001$, ‘**’ for $p \leq 0.01$, ‘*’ for $p \leq 0.05$, and ‘ns’ for non-significant) are now uniformly applied and clearly defined in all table footnotes. Pairwise t-values and overall P-values are presented in a consistent format across all trait tables.
Reviewer #1 Comment 4:
Since the study is confined to the M_1 generation, interpretations related to breeding value and selection should be slightly moderated, with clearer emphasis on the need for confirmation in M_2 and subsequent generations due to chimerism and the limited expression of recessive mutations in M_1.

Response:

We fully agree with this important observation. Throughout the revised manuscript particularly in the Discussion and Conclusion sections we have moderated all statements relating to breeding value and selection. We now clearly emphasize that observations in the M_1 generation are preliminary in nature and may be influenced by chimerism and by the limited phenotypic expression of recessive mutations, which are detectable only upon homozygosity in M_2 and later generations. Specifically, we have added explicit statements to the effect that the putative mutants identified through biplot and cluster analysis must be confirmed through M_2 progeny evaluation before any breeding value can be attributed, and that trait stabilization requires evaluation across multiple selfing generations. This framing is now consistent with established principles of mutation breeding and aligned with the interpretive conventions applied in comparable M_1 studies in cowpea and related legumes.

Reviewer #1 Comment 5:
Although the manuscript is generally well written, minor grammatical issues, long sentences, and occasional repetition should be corrected during revision.

Response:
We thank the reviewer for this feedback. The entire manuscript has been carefully proofread. Long sentences have been broken into shorter, clearer statements; repeated phrases and redundant expressions have been removed or consolidated; and minor grammatical issues throughout the text have been corrected. We trust that these revisions have meaningfully improved the readability and overall presentation of the manuscript.

Reviewer #2 Comment 1:
Statement from manuscript:
"This study investigated the impact of ethyl methane sulfonate (EMS) on the M1 generation of cowpea (Vigna unguiculata (L.) Walp). Seventy-five seeds of 'Wang Kae' cowpea variety were soaked in water for six hours and later subjected to three different doses of ethyl methane sulfonate (20 mM, 40 mM and 80 mM) with fifty serving as a control."
Reviewer Comment:
What do you mean by this statement?

Response:
We thank the reviewer for flagging this ambiguity. The original statement was poorly worded and could be misinterpreted to suggest that seeds were first soaked in water for six hours and then additionally soaked in EMS, which would be incorrect. The revised manuscript now makes the protocol explicit and unambiguous. Seventy-five seeds per EMS treatment group were pre-soaked in distilled water for six hours to facilitate imbibition and improve mutagen uptake, after which they were immediately transferred to and soaked in the respective EMS solutions (20 mM, 40 mM, or 80 mM) for a further six hours. A separate group of fifty seeds was soaked in distilled water only for six hours and served as the untreated control (0 mM). Following treatment, all seeds treated, and control were thoroughly washed in running water for 30 minutes to remove residual EMS, then planted to raise the M_1 generation. This clarified protocol has been rewritten in the Materials and Methods section to remove any ambiguity regarding the treatment sequence.

Reviewer #2 Comment 2:
1. The introduction is very shallow. No clear picture of previous studies and outcome. No evidence of justification of study. Problem statement not convincing or not there at all. The crop not well explained and described.
2. Your paragraphs are too short to be termed as paragraphs. Kindly structure the introduction well to give a clear picture of the study review.
3. Needs to be referenced.

Response:
We fully accept this criticism and have substantially revised and expanded the Introduction in the revised manuscript. The original Introduction was underdeveloped, consisting of short, underdeveloped paragraphs that did not adequately describe the crop, review the existing literature, or justify the study. The following specific changes have been made:

First, the description of cowpea has been expanded to include its economic and nutritional importance, its role in food and income security for smallholder farmers in Sub-Saharan Africa, its protein content and drought tolerance, and its significance as a climate-resilient legume in the context of food security in the tropics. Appropriate citations have been added to all factual statements about the crop.

Second, a structured review of previous EMS mutagenesis studies in cowpea and related legumes has been added. This now includes a summary of outcomes from key studies, including Opoku Gyamfi et al. (2022) in cowpea, Gnanamurthy and Dhanavel (2014) in cowpea, and Odeigah et al. (1998), along with comparable EMS studies in mung bean, black gram, and pigeonpea. The review highlights both the successes of mutation breeding in pulses and the gaps that remain, particularly the limited work in West African cowpea germplasm.

Third, a clear and convincing problem statement has been added, noting that: (i) the genetic base of cowpea in West Africa is narrow; (ii) conventional breeding has made limited progress in improving yield-related traits due to this limited variability; (iii) EMS mutagenesis offers a cost-effective and well-established route to broadening genetic diversity; and (iv) no previous study has characterized the M_1 EMS mutagenesis response of the '*Wang Kae*' cultivar across the full spectrum of phenological, yield-related, and yield traits using multivariate analysis.

Fourth, the short, disjointed paragraphs have been consolidated into three well-developed paragraphs covering: (a) the importance and constraints of cowpea production; (b) the rationale for and evidence base of mutation breeding as a strategy; and (c) the specific objectives of the present study. All statements in the revised Introduction are fully referenced.

Reviewer #2 Comment 3:
Statement from manuscript:
"Germination and survival rate. Germination of seeds was recorded 7 days after planting. Survival plants were recorded at 50% flowering and germination and survival rate were recorded using the formula by [11] ..."
Reviewer Comment:
This should be part of parameters recorded, not Statistical analysis.

Response:
The reviewer is entirely correct. The formulas for germination rate and seedling survival rate are measurement and data collection procedures, not statistical analyses, and should therefore

appear under the 'Parameters Studied' sub-section rather than the 'Statistical Analysis' sub-section. In the revised manuscript, this content has been moved to the 'Parameters Studied in M_1 Generation' section, where it now appears as the first sub-section under data collection and measurement, immediately after the list of phenological and yield-related traits.

Reviewer #2 Comment 4:
To ensure a proper review of this study, I humbly suggest authors separate results from the discussion. As it stands now some parts are well discussed and others are not discussed at all. This makes the manuscript very shallow and not presentable. Conventionally, a separate discussion will give a clear picture of the study since it will capture the review done in the introduction and your findings together with previous studies to help in making the manuscript solid.

Response:
We thank the reviewer for this important and well-founded observation. We agree entirely that the combined Results and Discussion format led to uneven treatment of the data: some findings were well discussed while others lacked contextualization, and the overall narrative failed to build a coherent argument connecting the results to the broader literature.

In the revised manuscript, the Results and Discussion sections have been fully separated. The Results section now presents all findings in a logical, structured sequence beginning with percentage germination and seedling survival, followed by the contrast analysis of phenological traits, yield-related traits, and yield per plant (with Tables 1 and 2), and concluding with the multivariate analysis results (PCA biplot and cluster analysis). Each sub-section reports the data clearly and objectively without interpretation.

The Discussion section has been substantially expanded and now constitutes a standalone section that: (i) interprets the key findings in the context of the hypotheses and study objectives; (ii) compares and contrasts the results with findings from previous EMS and mutagen studies in cowpea and related legumes; (iii) explains the biological mechanisms underlying the observed patterns, including the dose–response relationships, phenological delay, seed trait changes, and yield responses; (iv) discusses the significance of the PCA and cluster analysis results for putative mutant selection; and (v) acknowledges the limitations of M_1 generation data and the need for M_2 confirmation. The revised Discussion is directly informed by and refers to the expanded Introduction, creating a coherent narrative throughout the manuscript.
