# Supplementary material for: Effect of ethyl methane sulfonate mutagenesis on phenological, yield-related and yield traits in cowpea (Vigna unguiculata (L.) Walp)
Source: PLoS One. 2026 Jul 20;21(7):e0354037. doi: 10.1371/journal.pone.0354037 (PMC13384270; doi:10.1371/journal.pone.0354037)
Supplement: Attachment — Submitted filename: Response_to_Reviewers_auresp_2.pdf [file pone.0354037.s008.pdf]

HENRY KOFI MENSAH
Department of Plant and Environmental Biology
University of Ghana
P. O. Box LG 55, Legon–Accra, Ghana
Email: henryquofi@gmail.com
Tel: +1 437 383 2410

12th June 2026
The Academic Editor and Reviewers
PLOS ONE

Dear Academic Editor and Reviewers,

<u>Response to Reviewers – Manuscript ID: PONE-D-26-01099R1</u>

We sincerely thank you for your careful review of our manuscript and for the constructive comments provided. We appreciate the time and effort invested in evaluating our work. We have carefully addressed all comments raised by the reviewers and made the necessary corrections and improvements in the revised manuscript.

Below, we provide a point-by-point response to each comment.

Reviewer #1

Comment 1: All comments have been addressed.

Response:
We sincerely thank Reviewer #1 for the positive evaluation of our revised manuscript. We are pleased that the reviewer finds that all previous concerns have been adequately addressed. We appreciate the recognition of the improvements made in clarity, methodology, data presentation, and overall scientific quality.

Reviewer #2

Comment 1: Authors should please check the referencing in text very well. I have made examples to follow in the attached manuscript review.

Response:
We thank the reviewer for this important observation. We have carefully reviewed the entire manuscript and corrected all in-text citations and the reference list to ensure consistency, accuracy, and adherence to the journal's formatting style. All cited works have been verified and aligned with the required referencing format. These corrections have been incorporated throughout the revised manuscript.

Sincerely yours,

Henry Kofi Mensah
(Corresponding author)
On behalf of all co-authors
