# Supplementary material for: Effect of ethyl methane sulfonate mutagenesis on phenological, yield-related and yield traits in cowpea (Vigna unguiculata (L.) Walp)
Source: PLoS One. 2026 Jul 20;21(7):e0354037. doi: 10.1371/journal.pone.0354037 (PMC13384270; doi:10.1371/journal.pone.0354037)
Supplement: Attachment — Submitted filename: EFFECT OF EMS ON TRAITS IN COWPEA.docx [file pone.0354037.s003.docx]

**Effect of Ethyl Methane Sulfonate Mutagenesis on Phenological, Yield-Related and Yield Traits in Cowpea *(Vigna unguiculata* (L.) Walp)**

Henry .K. Mensah^1¶*^, Ralieva A.K. Norety^1¶^ , Isaac .K. Asante^1&^, Felicia Oppong^1&^

^1^Department of Plant and Environmental Biology, University of Ghana, Accra, Ghana

*Corresponding author

[henryquofi@gmail.com](mailto:henryquofi@gmail.com) (HKM)

.

^¶^ These authors contributed equally to this work.

^&^These authors also contributed equally to this work.

# Abstract

This study investigated the impact of ethyl methane sulfonate (EMS) on the M_1_ generation of cowpea (*Vigna unguiculata* (L.) Walp). Seventy-five seeds of the ‘Wang Kae’ cowpea variety were soaked in water for six hours and later subjected to three different doses of ethyl methane sulfonate (20 mM, 40 mM, and 80 mM), with fifty serving as a control. Phenological, yield-related, and yield-specific data were collected in accordance with the International Board for Plant Genetic Resources cowpea descriptor. Data analysis was performed using Jamovi 2.7.15 and Jasp 0.95.4.0 to compute one-way ANOVA and post hoc contrast, principal component biplot, and cluster analyses. No optimal EMS concentration (LD50) for inducing mutations was identified. The maximum seed germination rate of 89.33% and seedling survival rate of 74.67% were recorded at 80 mM, whereas the control group exhibited the lowest rates, with 70.00% germination and 62.00% survival. The trend observed for both seed germination and seedling survival rates was: 0 mM < 20 mM < 40 mM < 80 mM. Significant differences (P<0.05) in all phenological traits, pod length, locule number, seed traits, and yield per plant were found between the control and treatments. Yield was significantly higher (P = 0.047) at 20 mM, with a mean of 61.19 ± 3.34 g, compared to the control. Contrast analysis identified genotypes B33 and D56 as the most productive mutants, yielding 125.44 g and 111.85 g, respectively. Principal component analysis produced eighteen components, with the first four accounting for 50.60% of total variation. Biplot analysis revealed that PC1 and PC2 encompassed all phenological traits, key seed traits, and yield attributes, especially for genotypes B33 and D56. Cluster analysis divided the 190 genotypes into six groups, with B33 and D56 constituting distinct clusters 4 and 1, respectively. In conclusion, the EMS mutagenesis was effective in inducing unique variations that will be useful for breeding and the development of new farmer-preferred varieties.

# Introduction

Cowpea (Vigna unguiculata [L.] Walp.) is a widely cultivated and nutritionally important legume, predominantly grown in tropical and subtropical regions, particularly in Sub-Saharan Africa, Asia, and Central and South America, while also being adapted to certain temperate zones such as the Mediterranean basin and the southern regions of the United States [1-3].

Due to a decline in cowpea market value caused by pests, pathogens, and a lack of genetic variability, many scientists worldwide have sought to develop cowpea varieties that meet the taste and demand of a growing population [4-7]. **Previous studies have demonstrated substantial genetic variability in cowpea for economically important traits, including protein and mineral composition, underscoring the need to broaden the genetic base through breeding or mutagenesis** [11,12]. The most noteworthy studies have used mutagens to increase genetic variability [4-10]. Ethyl Methane Sulfonate (EMS), one of the most effective mutagens, has been successfully used by several researchers in West Africa to improve genetic variability in cowpea [6, 13].

A mutation is a heritable alteration in the structure or nucleotide sequence of a gene that arises suddenly and can be transmitted to subsequent generations. It results from changes in DNA such as substitutions, insertions, deletions, or rearrangements, and may occur naturally through replication or repair errors or be intentionally induced by physical and chemical mutagens. In plants, mutations can be fixed in both seed-propagated and vegetatively propagated species, allowing stable transmission of new traits. Consequently, mutations constitute a fundamental source of genetic variation and play a critical role in plant breeding and crop improvement programs [14,15].

To create functional mutants in various crop species, a variety of chemical mutagens have been used. The artificial induction of mutation by colchicine (Col), ethyl methane sulphonates (EMS), and sodium azide (SA) provides a tool to overcome the limitations of variability in plants, particularly Carnation (*Dianthus caryophyllus*), that induces specific improvement without disturbing their better attributes, according to various researchers [14]. The present study was undertaken to test the efficacy of EMS in inducing variability in phenological, yield-related, and yield characters of cowpea, thereby enabling the selection of superior genotypes for these essential traits.

# Materials & Methods

## Experimental site, material and design

The field study was carried out at the Department of Plant and Environmental Biology, University of Ghana, from early April to July 2024. Seventy-five seeds of the ‘*Wang Kae*’ cowpea variety (M_0_ generation) were soaked in water for six hours and later subjected to three different doses of ethyl methane sulfonate (20 mM, 40 mM, and 80 mM), with 50 serving as a control. Genotypes were labelled according to the EMS treatment dose. Genotypes exposed to 0 mM EMS were designated A1–A31, those treated with 20 mM EMS were labelled B1–B48, genotypes subjected to 40 mM EMS were designated C1–C55, and those treated with 80 mM EMS were labelled D1–D56. The treated seeds were thoroughly washed in running water for 30 minutes to reduce the residual effect of the mutagen sticking to the seed coat. The treated seeds, along with the control, were planted to raise the M1 generation, and standard cultural practices were carried out thereafter. The experiment was designed in pots measuring 3.29 m^2^. Spacing used was 0.8m between lines and 0.5 m between rows. One cowpea seed was planted per hole in each pot, at a depth of 3-4 cm.

## Parameters studied in the M_1_ generation

The growth, yield, and yield-related parameters studied in the M1 generation were grouped as phenological: days to germination (dtg), days to first flower (dff), days to 50% flowering (d50f), days to first harvest (dfh), days to 50% mature pod (d50mp); and yield-related traits: peduncle length at first harvest (pel), number of pods per peduncle (npp), number of pods per plant at maturity (nptm), number of seeds per pod (nsp), number of seeds per plant (nspt), pod length (pdl), pod width (pdw), number of locules (ln), percent seed abortion (psa), seed length (sdl), seed width (sdw), seed thickness (sdt), seed weight (swg), and yield per plant (yld).

Percentage seed abortion was calculated as; $\frac{\left[ \left( number of locules-number of seed per pod \right) \right]}{number of locules} x 100$%...................................................................1

Yield (g) was calculated as:$\frac{100 seed weight (g) x number of seeds per plant}{100}$……………..………..2

## Statistical analysis

### Germination and survival rate

Seed germination was recorded 7 days after planting. Survival plants were recorded at 50% flowering, and germination and survival rate were recorded using the formula by [16]

$Germination rate \left( \% \right)=\frac{\left( number of germinated seeds \right)}{\left( number of \left( un \right)treated seeds planted \right)}x 100$….…………………..….…3

$Survival rate (\%) =\frac{(number of survived seedlings)}{(number of germinated seeds )}$ x 100…………………………..…..…..4

[16]

XLSTAT was also used to generate the linear regression equation of seedling germination and survival rate.

### Distribution and post hoc contrast analysis for the selection of putative traits and mutants’

Descriptive statistics, one-way ANOVA, and post hoc contrast analysis were used to compare treatment effects for each trait in Jamovi 2.7.15.0. Plants within treatments whose values fell outside the control range for each character and were statistically significant from the control were selected as putative mutants**.**

### Multivariate analysis for the verification of the selected putative mutant traits and genotypes

Principal Component Analysis (PCA) was performed, accompanied by biplot analysis in Jamovi 2.7.15.0. Principal Component Analysis for quantitative traits was employed to determine the percentage contribution of each trait to total genetic variation. The principal components (PCs) with eigenvalues >1 were selected, and traits with coefficients >0.3 were considered to have significantly contributed to the variability. The biplot displayed computed similarity values to show the relationships among traits for each treatment and genotype. Cluster analyses were performed using Jamovi 2.7.15.0 to group genotypes within treatments using the Ward D2 hierarchical clustering optimization criterion. The circular dendrogram, plotted from the computed similarity values, showed relationships among genotypes and traits that differed significantly among treatments.

# Results and discussion

## Percentage germination and survival of M_1_ plants

The results for seed germination and survival rates (%) and linear regression fits for M₁ cowpea genotypes subjected to three EMS doses for 6 h are presented in Fig 1. The highest seed germination rate (89.33%) and seedling survival rate (74.67%) were recorded at 80 mM, whereas the lowest germination rate (70.00%) and seedling survival rate (62.00%) were observed in the 0 mM (control) treatment. The percentages of seed germination and seedling survival were in this order: 0 mM < 20 mM < 40 mM < 80 mM.

Linear regression analysis revealed a strong positive relationship between EMS concentration and both seed germination and survival rate (Fig 1). For seed germination rate, the regression equation was y = 6.4667x + 65.333 with an R^2^ value of 0.9395. Seedling survival showed a positive linear relationship with EMS concentration as follows:

y = 4.7333x + 56.667 with an R^2^ value of 0.9042.

Fig 1: Seed germination and survival rates (%) and linear regression fits for M₁ cowpea genotypes subjected to three ethyl methanesulfonate (EMS) doses for 6 h.

In the present study, LD₅₀ was not achieved, as seed germination and seedling survival increased with increasing EMS concentration. This response differs from the findings of [14, 17- 19]. [17], who reported an apparent dose-dependent reduction in germination and survival in the M₁ generation of cowpea varieties *Pusa Komal* and *V-240 (Rambha)* following EMS treatments at 0.1–0.3%, with control germination exceeding 95% and higher doses approaching the LD₅₀ threshold. Similarly, [13] observed a strong negative linear relationship between EMS concentration and germination in cowpea and identified an optimal LD₅₀ dose at 0.4% EMS. The contrast observed in this current study was that germination and survival were lowest in the control. It increased with EMS concentration, indicating that LD₅₀ expression is highly dependent on genotype, EMS dose range, and exposure duration. Unlike previous studies that employed percentage-based EMS concentrations and different treatment protocols, the present study used millimolar EMS concentrations with a fixed 6-h exposure, which may have altered the physiological response. In addition, the relatively low germination and survival recorded in the control suggest baseline establishment constraints that may have obscured EMS-induced lethality. These findings indicate that the absence of an LD₅₀ endpoint in the present study does not imply insufficient mutagenic effect. Instead, it underscores the importance of genotype × dose × exposure-time interactions in EMS mutagenesis.

## Contrast analysis of traits in the M_1_ generation

### Phenological traits

**Days to germination (*dtg*)** differed significantly among treatments (P < 0.001). The control (0 mM) germinated earliest, with a mean of 3.45 ± 0.13 days and a range of 2–5 days. All EMS treatments delayed germination, with the most substantial effect at 20 mM, while 40 and 80 mM showed delayed but comparable responses. The frequency of putative mutants was 50.0% at 20 mM, 29.10% at 40 mM, and 30.36% at 80 mM (Table 1). Selected putative mutants can be found in S1 Table.

**Days to first flower (*dff*)** were significantly affected by EMS across treatments (P < 0.001). *DFF was the* earliest in the control, with a mean of 37.87 ± 0.77 days and a range of 30 to 45 days, and was progressively delayed with increasing EMS concentration, reaching a maximum mean of 45.04 ± 0.86 days at 40 mM. Mutant frequencies declined from 51.17% at 20 mM to 41.07% at 80 mM (Table 1). Selected putative mutants can be found in S1 Table.

**Table 1: Mean, variability, mutant frequency, and pairwise significance of phenological traits in the M₁ generation of cowpea under different EMS concentrations**

| **Trait** | **Trt** | **Mean±se** | **%CV** | **Min** | **Max** | **Freq** | **P** | **Significant pairwise comparisons** | | | |
| --- | --- | --- | --- | --- | --- | --- | --- | --- | --- | --- | --- |
|  |  |  |  |  |  |  |  | **0mM** | **20mM** | **40mM** | **80mM** |
| dtg | 0mM | 3.45±0.13_a_ | 20.90 | 2 | 5 |  | <0.001 |  | <0.001 | <0.001 | <0.001 |
|  | 20mM | 5.38±0.20_b_ | 25.30 | 3 | 7 | 50.00 |  | <0.001 |  |  |  |
|  | 40mM | 4.78±0.19_b_ | 29.20 | 3 | 7 | 29.10 |  | <0.001 |  |  |  |
|  | 80mM | 4.77±0.19_b_ | 29.70 | 3 | 7 | 30.36 |  | <0.001 |  |  |  |
| dff | 0mM | 37.87±0.77_a_ | 11.30 | 30 | 45 |  | <0.001 |  | <0.001 | <0.001 | <0.001 |
|  | 20mM | 44.69±0.91_b_ | 14.10 | 33 | 54 | 51.17 |  | <0.001 |  |  |  |
|  | 40mM | 45.04±0.86_b_ | 14.10 | 34 | 55 | 47.27 |  | <0.001 |  |  |  |
|  | 80mM | 44.46±0.79_b_ | 13.30 | 33 | 55 | 41.07 |  | <0.001 |  |  |  |
| d50f | 0mM | 57.84±0.92_a_ | 8.90 | 48 | 68 |  | <0.001 |  | <0.001 | <0.001 | <0.001 |
|  | 20mM | 64.67±0.99_b_ | 10.70 | 50 | 75 | 37.50 |  | <0.001 |  |  |  |
|  | 40mM | 65.18±0.97_b_ | 11.00 | 49 | 80 | 40.00 |  | <0.001 |  |  |  |
|  | 80mM | 64.79±0.85_b_ | 9.80 | 53 | 80 | 28.57 |  | <0.001 |  |  |  |
| dfh | 0mM | 49.35±1.15_a_ | 13.00 | 40 | 67 |  | <0.001 |  |  |  |  |
|  | 20mM | 60.17±0.94_b_ | 10.80 | 48 | 72 | 18.75 |  | <0.001 |  |  |  |
|  | 40mM | 58.93±0.97_b_ | 12.10 | 38 | 72 | 9.100 |  | <0.001 |  |  |  |
|  | 80mM | 58.50±0.91_b_ | 11.70 | 43 | 72 | 12.50 |  | <0.001 |  |  |  |
| d50mp | 0mM | 79.48±1.41_a_ | 9.90 | 66 | 94 |  | <0.001 |  | <0.001 | <0.001 | <0.001 |
|  | 20mM | 90.17±1.37_b_ | 10.50 | 68 | 107 | 39.58 |  | <0.001 |  |  |  |
|  | 40mM | 88.65±1.09_b_ | 9.10 | 68 | 106 | 25.45 |  | <0.001 |  |  |  |
|  | 80mM | 88.32±1.16_b_ | 9.80 | 69 | 110 | 23.21 |  | <0.001 |  |  |  |

Note. Means sharing the same superscript letter within a trait are not significantly different according to Tukey’s HSD test (P ≤ 0.05). Trt= Treatment, Mean ± SE = Mean plus or minus standard error, %CV= Percentage coefficient of variation, Min= minimum, Max= maximum, Freq= Percentage frequency of putative mutants; P=Probability value. Traits: dtg = days to germination, dff = days to first flower, d50f = days to 50% flowering, dfh = days to first harvest, d50mp = days to 50% mature pods

**Days to 50% flowering** (***d50f***) were significantly affected by EMS across treatments (P < 0.001). ***D50f*** increased from a control mean of 57.84 ± 0.92 days, with values ranging from 48 to 68 days, to mean values of approximately 65 days across EMS treatments, with broader ranges of 49–80 days. Corresponding mutant frequencies were 37.50%, 40.00%, and 28.57% at 20, 40, and 80 mM, respectively (Table 1). Selected putative mutants can be found in S1 Table.

**Days to first harvest *(dfh)*** were the earliest in the control, with a mean of 49.35 ± 1.15 days and a range of 40–67 days, and were significantly (P < 0.001) delayed in all EMS treatments. Mean dfh increased to 60.17 ± 0.94 days at 20 mM, 58.93 ± 0.97 days at 40 mM, and 58.50 ± 0.91 days at 80 mM. Mutant frequencies were 18.75% at 20 mM, 9.10% at 40 mM, and 12.50% at 80 mM (Table 1). Selected putative mutants can be found in S1 Table.

A similar trend was observed for **days to 50% maturity of pods (*d50mp*)**, with the control recording the earliest maturity at 79.48 ± 1.41 days, and values ranging from 66 to 94 days. EMS treatments significantly (P<0.001) delayed pod maturity compared with the control, with mean ***d50mp*** values of 90.17 ± 1.37 days at 20 mM, 88.65 ± 1.09 days at 40 mM, and 88.32 ± 1.16 days at 80 mM. The frequency of selected mutants for d50mp was 39.58%, 25.45%, and 23.21% at 20, 40, and 80 mM, respectively (Table 1). Selected putative mutants can be found in S1 Table.

In our study, EMS treatments altered the timing of developmental events in cowpea, including flowering and maturity. The expansion of phenotypic variability observed in the present EMS-treated population is particularly relevant for cowpea improvement, as earlier studies have demonstrated that key agronomic and nutritional traits are under strong genetic control and respond effectively to selection when sufficient genetic variability is available [3, 11-12]. Days to first flowering and days to 50% flowering tended to be delayed in many high-dose EMS mutants, although some early-flowering outliers were observed at lower doses. The delay in flowering under stronger mutagenesis is a common phenomenon, often attributed to physiological stress or to disruption of genes controlling flowering time [20-23]. [20] did not directly measure flowering time, but their M₁ mutants showed overall physiological delays, and other researchers have explicitly noted flowering extensions. For example, [24] working with mung bean reported that higher EMS levels prolonged the time to flowering and maturity, likely due to mutational interference with developmental pathways. It is also recorded by [25] in black gram, [26] in *Gloriosa superben,* and [8, 27] in *Vigna radiata*. In cowpea, [28] observed an earlier onset of flowering and a decrease in days to 50% flowering in an EMS-treated yardlong bean (*Vigna sesquipedalis*) population, accompanied by reduced maturity duration.

Such wide variation in flowering time due to EMS has also been reported by [13], who found that their M₂ cowpea population showed continuous distribution for days to flowering far beyond the range of the wild type. This implies that EMS created both later- and earlier-flowering individuals. Notably, [13] identified dozens of mutants that flowered significantly (P<0.05) earlier than the parent variety, indicating the emergence of new earliness traits. These results suggest that specific mutations can accelerate flowering, potentially by knocking out repressors of flowering even as overall vigor is reduced. In our mutants, while the predominant trend at high EMS dosages was delayed flowering and maturity, a few early-blooming mutants were observed at lower EMS treatments, indicating increased phenological variation.

Our results confirm that EMS mutagenesis increased the variance in flowering time. While early flowering is advantageous for drought avoidance and multi-cropping systems, our evaluation of the EMS population did not yield any early-flowering mutants; instead, such occurrences were observed in the control group. This outcome aligns with results previously reported in mung bean and other pulse crops.

### Yield-related and yield traits

The **pod length (*pdl*)** was highest in the control (0 mM), with a mean of 14.65 ± 0.31 cm and a range of 11.55–17.90 cm, whereas 80mM recorded the lowest mean pdl of 13.52±0.28 cm, which was significantly different (P=0.008) from the control. Based on deviations from the control range, the frequency of selected mutants for ***pdl*** was 10.42%, 9.10%, and 21.43% in 20 mM, 40 mM, and 80 mM, respectively (Table 1). Selected putative mutants can be found in S1 Table.

The ***number of locules per pod (ln)*** differed significantly among EMS treatments (P < 0.001). The control recorded a mean of 17.00 ± 0.60, with a range of 12 to 24 locules. Higher EMS concentrations resulted in a significant reduction in locule number: 14.93 ± 0.48 locules at 40 mM and 11.41 ± 0.39 locules at 80 mM. Pairwise comparisons showed that only the 80 mM treatment differed significantly (P < 0.001). From both the control and all other treatments, Phenotypic variability increased with EMS dose, as reflected by a rise in the coefficient of variation from 17.5% at 20 mM to 25.8% at 80 mM. Based on deviations

**Table 2: Mean, variability, mutant frequency, and pairwise significance of yield-related and yield traits in the M₁ generation of cowpea under different EMS concentrations**

| **Trait** | **Trt** | **Mean±se** | **%CV** | **Min** | **Max** | **Freq** | **P** | **Significant pairwise comparisons** | | | |
| --- | --- | --- | --- | --- | --- | --- | --- | --- | --- | --- | --- |
|  |  |  |  |  |  |  |  | **0mM** | **20mM** | **40mM** | **80mM** |
| npp | 0mM | 3.84±0.17 | 25.30 | 2 | 5 |  | 0.447 |  |  |  |  |
|  | 20mM | 4.02±0.13 | 21.50 | 2 | 5 |  |  |  |  |  |  |
|  | 40mM | 3.82±0.13 | 25.70 | 2 | 5 |  |  |  |  |  |  |
|  | 80mM | 3.70±0.16 | 31.80 | 2 | 5 |  |  |  |  |  |  |
| nptm | 0mM | 24.68±1.31 | 29.50 | 14 | 44 |  | 0.412 |  |  |  |  |
|  | 20mM | 25.46±1.07 | 29.10 | 10 | 45 |  |  |  |  |  |  |
|  | 40mM | 23.25±0.78 | 25.00 | 11 | 44 |  |  |  |  |  |  |
|  | 80mM | 23.87±0.97 | 30.40 | 10 | 46 |  |  |  |  |  |  |
| nsp | 0mM | 9.84±0.63 | 35.60 | 5 | 15 |  | 0.730 |  |  |  |  |
|  | 20mM | 9.92±0.46 | 32.30 | 5 | 15 |  |  |  |  |  |  |
|  | 40mM | 10.44±0.39 | 27.80 | 5 | 15 |  |  |  |  |  |  |
|  | 80mM | 9.80±0.46 | 35.10 | 5 | 15 |  |  |  |  |  |  |
| nspt | 0mM | 233.10±14.38 | 34.30 | 100 | 406 |  | 0.689 |  |  |  |  |
|  | 20mM | 246.29±13.29 | 37.40 | 100 | 450 |  |  |  |  |  |  |
|  | 40mM | 238.20±10.34 | 32.20 | 100 | 435 |  |  |  |  |  |  |
|  | 80mM | 226.36±12.17 | 40.20 | 100 | 450 |  |  |  |  |  |  |
| pdl | 0mM | 14.65±0.31b | 11.80 | 11.55 | 17.86 |  | 0.030 |  |  |  | 0.008 |
|  | 20mM | 14.34±0.25ab | 12.30 | 10.50 | 19.88 | 10.42 |  |  |  |  |  |
|  | 40mM | 13.83±0.26ab | 14.10 | 6.95 | 16.75 | 9.10 |  |  |  |  |  |
|  | 80mM | 13.52±0.28a | 15.20 | 8.45 | 17.50 | 21.43 |  | 0.008 |  |  |  |
| pdw | 0mM | 0.85±0.01 | 9.70 | 0.70 | 1.03 |  | 0.144 |  |  |  |  |
|  | 20mM | 0.89±0.02 | 16.50 | 0.61 | 1.23 |  |  |  |  |  |  |
|  | 40mM | 0.90±0.03 | 25.60 | 0.52 | 1.28 |  |  |  |  |  |  |
|  | 80mM | 0.83±0.02 | 18.00 | 0.60 | 1.13 |  |  |  |  |  |  |
| ln | 0mM | 17.00±0.60c | 19.70 | 12.00 | 24.00 |  | <0.001 |  |  | 0.023 | <0.001 |
|  | 20mM | 17.06±0.43c | 17.50 | 12.00 | 23.00 |  |  |  |  | 0.005 | <0.001 |
|  | 40mM | 14.93±0.48b | 23.90 | 8.00 | 21.00 | 20.00 |  | 0.023 | 0.005 |  | <0.001 |
|  | 80mM | 11.41±0.39a | 25.80 | 7.00 | 23.00 | 50.00 |  | <0.001 | <0.001 | <0.001 |  |
| psa | 0mM | 14.27±1.93 | 75.30 | 0.00 | 35.71 |  |  |  |  |  |  |
|  | 20mM | 18.60±1.68 | 62.70 | 0.00 | 35.71 |  |  |  |  |  |  |
|  | 40mM | 19.35±1.66 | 63.80 | 0.00 | 35.71 |  |  |  |  |  |  |
|  | 80mM | 15.43±1.53 | 74.20 | 0.00 | 35.71 |  |  |  |  |  |  |
| sdl | 0mM | 7.44±0.24a | 18.10 | 5.68 | 11.59 |  | <0.001 |  | <0.001 | <0.001 | <0.001 |
|  | 20mM | 8.77±0.25b | 19.60 | 6.10 | 11.65 | 0.42 |  | <0.001 |  | <0.001 | <0.001 |
|  | 40mM | 9.79±0.07c | 4.90 | 8.10 | 11.05 |  |  | <0.001 | <0.001 |  |  |
|  | 80mM | 9.95±0.09c | 6.60 | 7.95 | 11.60 | 1.79 |  | <0.001 | <0.001 |  |  |
| sdw | 0mM | 4.78±0.10a | 11.90 | 3.94 | 6.08 |  | <0.001 |  | <0.001 | <0.001 | <0.001 |
|  | 20mM | 5.14±0.09b | 11.80 | 4.05 | 6.44 | 0.42 |  | <0.001 |  |  |  |
|  | 40mM | 5.14±0.04b | 5.90 | 4.58 | 5.69 |  |  | <0.001 |  |  |  |
|  | 80mM | 5.25±0.05b | 7.00 | 4.28 | 6.11 | 1.79 |  | <0.001 |  |  |  |
| sdt | 0mM | 4.09±0.07a | 9.80 | 3.29 | 4.92 |  | <0.001 |  | 0.027 |  |  |
|  | 20mM | 4.35±0.08b | 12.20 | 3.31 | 5.19 | 12.50 |  | 0.027 |  | <0.001 | 0.005 |
|  | 40mM | 3.95±0.04a | 8.50 | 3.43 | 5.59 | 1.82 |  |  | <0.001 |  |  |
|  | 80mM | 4.08±0.05a | 8.90 | 3.41 | 4.94 | 1.79 |  |  | 0.005 |  |  |

Note. Means sharing the same superscript letter within a trait are not significantly different according to Tukey’s HSD test (P ≤ 0.05). Trt= Treatment, Mean ± SE = Mean plus or minus standard error, %CV= Percentage coefficient of variation, Min= minimum, Max= maximum, Freq= Percentage frequency of putative mutants; P=Probability value. Traits: npp = number of pods per peduncle, nptm = number of pods per plant at maturity, nspt = number of seeds per plant, pdl = pod length, pdw = pod width, ln = number of locules, nsp = number of seeds per pod, psa = percent seed abortion, sdl = seed length, sdw = seed width, sdt = seed thickness, swgt = seed weight, yld = yield per plant.

**Table 2: Mean, variability, mutant frequency, and pairwise significance of yield-related and yield traits in the M₁ generation of cowpea under different EMS concentration (cont’d)**

| **Trait** | **Trt** | **Mean±se** | **%CV** | **Min** | **Max** | **Freq** | **P** | **Significant pairwise comparisons** | | | |
| --- | --- | --- | --- | --- | --- | --- | --- | --- | --- | --- | --- |
|  |  |  |  |  |  |  |  | **0mM** | **20mM** | **40mM** | **80mM** |
| swg | 0mM | 21.03±0.34a | 9.10 | 17.82 | 24.87 |  | <0.001 |  | <0.001 | <0.001 | 0.020 |
|  | 20mM | 24.86±0.34c | 9.40 | 17.81 | 29.99 | 54.17 |  | <0.001 |  |  | <0.001 |
|  | 40mM | 24.37±0.35c | 10.70 | 17.70 | 28.39 | 49.10 |  | <0.001 |  |  | <0.001 |
|  | 80mM | 22.60±0.33b | 11.00 | 17.35 | 28.68 | 16.07 |  | 0.020 | <0.001 | <0.001 |  |
| yld | 0mM | 48.79±3.01a | 34.40 | 20.61 | 85.68 |  | 0.018 |  | 0.047 |  |  |
|  | 20mM | 61.19±3.34b | 37.80 | 18.70 | 125.44 | 16.67 |  | 0.047 |  |  |  |
|  | 40mM | 58.02±2.59ab | 33.10 | 19.97 | 105.43 | 12.72 |  |  |  |  |  |
|  | 80mM | 51.16±2.87ab | 42.00 | 18.45 | 111.85 | 10.71 |  |  |  |  |  |

Note. Means sharing the same superscript letter within a trait are not significantly different according to Tukey’s HSD test (P ≤ 0.05). Trt= Treatment, Mean ± SE = Mean plus or minus standard error, %CV= Percentage coefficient of variation, Min= minimum, Max= maximum, Freq= Percentage frequency of putative mutants; P=Probability value. Traits: npp = number of pods per peduncle, nptm = number of pods per plant at maturity, nspt = number of seeds per plant, pdl = pod length, pdw = pod width, ln = number of locules, nsp = number of seeds per pod, psa = percent seed abortion, sdl = seed length, sdw = seed width, sdt = seed thickness, swgt = seed weight, yld = yield per plant.

from the control range, the frequency of putative mutants for ***ln*** reached 20.0% at 40 mM and 50.0% at 80 mM (Table 1). Selected putative mutants can be found in S1 Table.

**Seed length (*sdl*)** varied significantly across EMS treatments (P < 0.001). The control recorded the lowest mean seed length (7.44 ± 0.24 mm), with a range of 5.68 to 11.59 mm and a CV of 18.1%. EMS treatment groups resulted in a progressive increase in seed length, with means of 8.77 ± 0.25 mm at 20 mM, 9.79 ± 0.07 mm at 40 mM, and 9.95 ± 0.09 mm at 80 mM. Pairwise comparisons showed that all EMS treatments differed significantly from the control (P < 0.001), while the 40 mM and 80 mM treatments did not differ from each other. Based on deviations from the control range, mutant frequencies were low for seed length, recorded at 0.42% for 20 mM and 1.79% for 80 mM (Table 1). Selected putative mutants can be found in S1 Table.

**Seed width (*sdw*)** was also significantly (P < 0.001) affected by EMS treatment. The control population had a mean width of 4.78 ± 0.10 mm, ranging from 3.94 to 6.08 mm, with a CV of 11.9%. All EMS treatments differed significantly from the control (P < 0.001). Variability declined at higher EMS doses, with CV values as low as 5.9% at 40 mM. Frequencies of putative mutants increased with EMS concentration, reaching 0.42% at 20 mM and 1.79% at 80 mM (Table 1). Selected putative mutants can be found in S1 Table.

**Seed thickness (*sdt*)** differed significantly among treatments (P < 0.001). The control recorded a mean thickness of 4.09 ± 0.07 mm with values ranging from 3.29 to 4.92 mm. Thickness increased significantly (P=0.027) at 20 mM with a mean of 4.36 ± 0.08 mm. Pairwise comparisons indicated significant differences between 20 mM and both 40 mM (P < 0.001) and 80 mM (P = 0.005). Mutant frequencies for seed thickness were highest at 20 mM (12.5%) but remained low at 40 mM (1.82%) and 80 mM (1.79%) (Table 1). Selected putative mutants can be found in S1 Table.

**100-Seed weight (*swg*)** showed highly significant variation among EMS treatments (P < 0.001). The control recorded a mean of 21.03 ± 0.34 g, with a range of 17.82 to 24.87 g. EMS treatment increased seed weight, with the highest means observed at 20 mM (24.86 ± 0.34 g) and 40 mM (24.37 ± 0.35 g), both of which differed significantly from the control (P < 0.001). The 80 mM treatment weighed 22.60 ± 0.33 g and showed an intermediate response, differing considerably from both the control (P = 0.02) and lower EMS doses (P < 0.001). Frequencies of putative mutants were highest at 20 mM (54.17%), followed by 40 mM (49.1%) and 80 mM (16.07%) (Table 1). Selected putative mutants can be found in S1 Table.

**Yield per plant (*yld*)** differed significantly among treatments (P = 0.018). The control population recorded the lowest mean yield of 48.79 ± 3.01 g, with a range of 20.61 to 85.68 g. Yield increased significantly (P = 0.047) at 20 mM, with a mean of 61.19 ± 3.34 g, which differed from the control (P = 0.047). The 40 mM (58.02 ± 2.59 g) and 80 mM (51.16 ± 2.87 g) treatments, which exhibited intermediate yields, did not differ significantly from either the control or the 20 mM treatment. Phenotypic variability was high across treatments, with a CV of 33.10–42%, and mutant frequencies declined with increasing EMS concentration, from 16.67% at 20 mM to 10.71% at 80 mM (Table 1). Selected putative mutants can be found in S1 Table.

The lack of notable mutants for number of pods per peduncle (*npp*), number of pods per plant at maturity (*nptm*), number of seeds per pod (*nsp*) and number of seeds per plant (*nspt*) in the M₁ generation is likely due to these traits being highly polygenic and developmentally buffered, which restricts observable changes [29- 31]. Yield components may mask mutational effects through compensatory relationships [32, 33]. However, significant mutants were identified for these traits in M₂ populations, where recessive mutations are revealed, and chimerism is resolved [13, 34-36]. Moreover, higher EMS doses often reduce pod and seed numbers due to partial sterility or lower pollen viability, as seen in legumes [19, 25, 37].

In the present study, several putative mutants from the 20 mM, 40 mM, and 80 mM EMS treatments exhibited significantly increased pod length, seed dimensions, seed weight, and yield per plant relative to the control. Comparable yield-enhancing mutations have been reported in earlier cowpea studies. For example, [13] identified M₂ cowpea mutants with significantly higher pods per plant, seeds per plant, seeds per pod, and pod length than the wild type. Those authors further noted that increased pod number, seed weight, and plant stature have frequently been achieved through EMS mutagenesis in cowpea.

Seed size traits also varied widely, with some mutants showing significantly longer and wider seeds in the present study. Variations in seed size and structure are particularly important in cowpea, as seed morphological traits have been associated with differences in protein content and mineral composition among genotypes, highlighting the potential nutritional relevance of EMS-induced mutants [3, 11-12]. Increased seed size due to mutagenesis has been reported previously [38]; isolated bold-seeded mutants in *Vigna mungo* with enhanced yield. In our research, high-yielding mutants, such as B09, typically produce larger seeds with higher seed weight, a favorable combination for yield improvement. Similarly, [38] and [39] reported cowpea mutants with enlarged seeds and improved yield performance, consistent with the superior lines identified in the present study.

Yield stimulation following mutagenic seed treatments has also been documented in other legumes. Increased yield or yield components after mutagen exposure have been reported in pigeonpea [40], mung bean [41], and mung bean treated with gamma rays, EMS, and epichlorohydrin [42]. These studies collectively support the notion that induced mutagenesis can unlock favorable allelic variation controlling yield traits.

In contrast, many mutants, particularly from the highest EMS dose (80 mM), exhibited reductions in yield and its components, including pod length, number of locules, seed size, and seed weight. Such adverse effects of high mutagen doses are well documented. [43] In okra and [44] in petunia, it was reported that mutagenesis frequently produces lines with reduced reproductive output. In cowpea, [45] observed that EMS doses beyond the optimal range led to sharp declines in pollen fertility and seed yield. At the same time, [46] reported positive yield shifts at lower mutagen doses and negative shifts at higher doses. Our results closely mirror this dose-dependent pattern. Yield depression at elevated doses of irradiation or chemical mutagens has been widely reported. Similar declines were observed by [25] in black gram, [26] in *Gloriosa* Khan *superba* under EMS and DES treatments, [47] in *Vigna mungo*, and [48] in *Vigna radiata*.

These findings reflect the accumulation of deleterious mutations at higher mutagen doses, which can impair physiological and reproductive processes. Conversely, [37] reported stimulation of seed weight in pigeon pea under combined mutagenic treatments, suggesting that moderate doses may enhance specific yield components. Similar observations were reported by [49], [50], and Patil and [51] across various pulse crops, reinforcing the dose- and trait-dependent nature of mutagenic responses.

In the present study, one elite mutant (B33) from the 20 mM EMS treatment produced approximately 25% higher seed yield than the control, accompanied by a modest increase in 100-seed weight. This indicates that EMS successfully generated yield-enhancing alleles. [52] Similarly, reported EMS-derived cowpea lines with significantly increased pod numbers confirm the potential of chemical mutagenesis to improve yield.

It is also noteworthy that the frequency of desirable mutants was low, consistent with previous reports. [13] estimated that only 0.1–1% of mutants exhibited significantly improved yield-related and yield traits. Similarly, although a few promising mutants were identified in the present study, the majority performed at or below the level of the control. This underscores the need to screen large mutant populations to capture rare beneficial events.

## Multivariate Analysis: PCA Biplot and Cluster Grouping

### PCA Biplot of phenological and yield-related traits

Principal component analysis (PCA) of the traits and genotypes of plant observations across EMS treatments yielded 8 principal components with eigenvalues < 1 (S2 Table). The first four principal components accounted for a total variability greater than 50.60% in all treatments. The first two principal components (Dim1 and Dim2) together explained 32.8% of the total phenotypic variation, with PC1 accounting for 17.7% and PC2 accounting for 15.1% (Fig 2, S2 Table). PC1 represented major seed and phenological traits, including seed length (*sdl*), seed width (*sdw*) days to first flower (*dff*), days to 50% flowering (*d50f*), days to first harvest (*dfh*), days to 50% pod maturity (*d50mp*) and *swg* loaded positively on PC1, indicating that higher PC1 scores were associated with delayed development. On the other hand, yield-related traits such as number of seeds per plant (*nspt*), number of seeds per pod (*nsp*), and yield (*yld*) were negatively correlated with PC2, reflecting an inverse relationship between prolonged phenology and yield-related traits.

Fig 2: PCA biplot of cowpea genotypes and traits based on PC1 and PC2. Genotypes treated with 0-, 20-, 40- and 80-mM EMS are labelled A01–A31, B01–B48, C01–C55 and D01–D56, respectively. Vectors indicate trait loadings, and points represent genotype distribution. Percent variance explained by each PC is shown on the axes. Key: dtg days to germination, dff = days to first flower, d50f = days to 50% flowering, dfh = days to first harvest, d50mp = days to 50% mature pods, npp = number of pods per peduncle, nptm = number of pods per plant at maturity, nspt = number of seeds per plant, pdl = pod length, pdw = pod width, ln = number of locules, nsp = number of seeds per pod, psa = percent seed abortion, sdl = seed length, sdw = seed width, sdt = seed thickness, swgt = seed weight, yld = yield per plant

The distribution of genotype plants on the PCA biplot revealed that most control plants (0 mM) formed a compact cluster on the negative side of PC1, reflecting early phenology, uniform development, and moderate to high yield. Plants from the 20 mM EMS treatment showed partial overlap with the control group. Still, they were moderately displaced toward positive PC1 values, indicating mild delays in phenological development and moderate reductions in yield components. Genotypes from the 40 mM and 80 mM EMS treatments were progressively displaced toward the positive side of PC1 and exhibited greater dispersion along

both PC1 and PC2.This shift reflects dose-dependent delays in flowering, harvest, and maturity, coupled with reductions in reproductive traits such as *nsp*, *nspt,* and *yld*.

Notably, vectors for seed length (*sdl*) and seed width (*sdw*) were oriented opposite to those for number of seeds per pod, plant, and yield. Genotypes positioned along the *sdl*/*sdw* vectors from the 40 mM and 80 mM treatments showed larger seed size but reduced yield, suggesting altered resource allocation under EMS-induced mutational stress.

Selection focused on genotypes aligned with key trait vectors representing yield performance, seed size, and phenological timing. High-yielding putative mutants were identified among genotypes positioned in the upper region of the biplot with positive PC2 scores, in close alignment with the ***yld***, ***nsp***, ***nptm***, and ***swg*** vectors. These included B33, B28, B22, B31, D56, D45, D25, C5, C41, B26, and B21, representing EMS-derived plants with enhanced reproductive output relative to the population mean.

Large-seed putative mutants were selected from genotypes located along the negative PC2 axis, in the direction of the sdl and sdw vectors. Representative genotypes included D23, D7, D34, D47, C37, C39, C40, C42, B18, B11, and B8, which showed increased seed size but reduced seed number and yield.

Early-phenology putative mutants were identified from the extreme negative side of PC1, opposite to the late-flowering and maturity vectors. These included B19 and C2, reflecting genotypes with accelerated development and earlier flowering and maturity.

Genotypes exhibiting extreme positive PC1 scores with weak alignment to yield vectors, such as B9, B7, C46, and D20, were classified as severely delayed and low-performing and were not considered suitable for advancement; however, they were retained as extreme phenotypes for genetic and physiological reference.

The PCA biplot in our study was particularly informative, as it highlighted which traits accounted for the most variation among the EMS-treated lines. Notably, yield-related (*npp*, *nsp,* and *swg)* and yield traits loaded strongly on the first two principal components, indicating these had the most influence in differentiating mutants. Traits with minimal impact in PCA were also not highlighted in the clustering. By focusing on the PCA, we found a clear separation between high- and low-yielding mutants, with high-yield lines clustering in the quadrant associated with greater pod and seed numbers. In contrast, mutants with delayed phenology or poor fertility tended to lie on the opposite end of the PCA axes, separated from the productive genotypes. This reflects the underlying trade-offs induced by EMS: lines that suffered heavy damage, for example, dwarf and sterile mutants, clustered away from those that managed to improve or maintain yield traits. The utility of PCA in such mutation studies is well recognized; it helps distill many correlated traits into composite axes of variation.

[53], For instance, used multi-trait analysis to identify superior M₂ individuals and reported that mutants with extremely high values in key traits, such as seeds per plant, could be distinguished from the population distribution. Our PCA biplot offers similar insights by visually highlighting the direction of trait enhancement, such as higher yield, and demonstrating that the notable mutants distinctly deviated from the control group's path in multivariate analysis.

### Cluster analysis of phenological and yield-related traits

Hierarchical cluster analysis based on phenological, yield-related, and yield traits grouped the cowpea M₂ population into six distinct clusters (Fig 3, S1 Table). Cluster 1 comprised predominantly EMS-derived genotypes, representing 35.42%, 49.10%, and 33.93% of the 20 mM, 40 mM, and 80 mM treatment groups, respectively. Only a single control genotype (A01) clustered within this group, accounting for 3.22% of the 0 mM population. Genotypes in this cluster were characterized by prolonged phenological development coupled with superior yield performance. Days to 50% flowering (*d50f*) ranged from 53 to 80 days, with the most pronounced delays observed in C43 and C41, while days to 50% pod maturity (*d50mp*) ranged from 69 to 109 days, with the most extended delays recorded in D43 and C08.

Despite delayed development, individuals in this cluster exhibited markedly higher seed weight (*swg*), ranging from 18.82 g (D04) to 28.68 g (D56), and elevated yield per plant (*yld)*, which ranged from 31.45 g (C37) to 111.85 g (D56), exceeding the control population. Selected putative mutants from Cluster 1 included D56, D45, D25, C41, C5, B31, B28, and B22, all of which showed apparent positive deviation from the control range for significant yield and yield-related traits.

Cluster 2 was dominated by control genotypes, accounting for 61.29% of the 0 mM population. Only 2.08%, 3.64%, and 3.57% of the 20 mM, 40 mM, and 80 mM treatment groups, respectively, were represented in this cluster. Genotypes in Cluster 2 exhibited early germination, flowering, and maturity, with yield levels comparable to the control mean. This cluster, therefore, represented the population's baseline phenotypic profile and exhibited low within-cluster variability. A small number of EMS-treated genotypes within this cluster showed improved yield during the early days to first harvest, notably C02 (*38 days*) and C14 (*39 days*), which were selected as early-phenology putative mutants.

Fig 3: Dendrogram of hierarchical cluster analysis based on quantitative traits using Ward’s linkage method, showing the genetic relationships among genotypes in the M_1_ generation based on average distance. Key: A = 0mM, B = 20mM, C = 60mM and D = 80mM, Green = Cluster 1 [64 genotypes], Brown = Cluster 2 [24 genotypes], Grey = Cluster 3 [31 genotypes], Forest green = Cluster 4 [20 genotypes], Blue = Cluster 5 [40 genotypes], Red = Cluster 6 [9 genotypes]

Cluster 3 comprised mainly high-dose EMS-derived genotypes, with 28.57% of the 80 mM population grouped within this cluster. Delayed phenological traits, fewer locules, and poor yield were characteristic of it. Representative genotypes, such as B7, B9, C46, and D20, showed a marked negative deviation from the control and were classified as low-value mutants and not considered for advancement.

Cluster 4 contained genotypes that combined early days to first flower (*dff*) and days to 50% flowering (*d50f*) with extended days to first harvest (*dfh*) and days to 50% mature pod (*d50mp*), resulting in high seed weight and elevated yield, particularly within the 20 mM EMS population. This cluster included 10.42%, 9.09%, and 12.50% of individuals from the 20 mM, 40 mM, and 80 mM treatments, respectively, indicating that favorable phenotypes arose across multiple EMS doses. Notable high-performing putative mutants in this cluster included B19 (*93.59 g*) and B33 (*125.44 g*), both of which substantially exceeded the control in yield while maintaining relatively early phenology.

Cluster 5 was composed exclusively of EMS-treated genotypes, representing 33.33%, 22.27%, and 16.07% of the 20 mM, 40 mM, and 80 mM treatment groups, respectively. These individuals exhibited moderately delayed phenological traits and high seed weight, but had fewer locules, resulting in inferior yield performance relative to the control.

Cluster 6 comprised a small but distinct group of EMS-derived genotypes, including 12.50% and 5.36% of the 20 mM and 80 mM populations, respectively. These genotypes were characterized by moderately delayed phenological traits, large seed size, and high seed weight. Despite a relatively low seed number per pod, total yield per plant was high, driven by seed weight. Selected putative mutants from this cluster included B07 and D34, which consistently outperformed the control in terms of seed weight.

The cluster analysis further allowed us to group the EMS-derived cowpea lines into distinct categories based on overall performance. We identified six clusters, but importantly, not all clusters were equal in agronomic value. The clustering effectively segregated the elite putative mutants that combine multiple favorable traits from average performers and poor performers. Cluster membership was closely associated with EMS treatment levels and trait profiles. For example, Cluster 2 in our results consisted predominantly of untreated control plants and a few low-dose mutants, characterized by moderate phenological and yield trait values (baseline yield and phenology). In contrast, another cluster (Cluster 5) contained high-yielding mutants, mainly from the 20 mM and 40 mM EMS treatments, with significantly higher seed weight than the control, underscoring that this group included the desirable phenotypes created by the mutagenesis.

A small cluster (Cluster VI) captured a handful of outlier mutants. These included some top-yielding lines for weight, as well as unusual genotypes, suggesting that, while most of the best mutants grouped, a few unique lines had singular trait combinations. On the other hand, a cluster 3 was observed, mainly composed of 80 mM EMS mutants with severely reduced yields and late maturation. This stratification by clustering aligns with expectations from other studies that employ grouping strategies in mutant populations.

Although formal cluster analysis is not always reported, researchers often categorize mutants by yield or other indices. For instance, [54] and [55] in the context of mutation breeding of pulses have discussed grouping mutant lines by overall performance, noting that mutants from optimal dose treatments tend to form a cohort of superior types. In contrast, those from high-dose treatments tend to form inferior groups. Our results mirror this trend: moderate EMS doses produced the bulk of the high-performance cluster. In contrast, the highest dose contributed disproportionately to the low-performance cluster, consistent with the dose–performance relationship reported by [46].

The clustering outcomes in our study also have practical breeding implications. The fact that the best-yielding mutants clustered together means they share a similar multi-trait profile, which could be harnessed as a selection index. We can infer that these elite lines possess a favorable combination of genes or alleles for yield, likely with minimal adverse pleiotropic effects on other traits. This is promising for developing mutant lines, as a high yield was achieved without significant negative consequences. [53] selected the top 4% of their M₂ population as high-yield mutants for further breeding. Likewise, our cluster analysis identified 10.71-16.67% of lines that significantly exceeded the others in yield per plant.

By cross-comparing our elite cluster with the control cluster, we found that improvements in yield traits were statistically significant, reinforcing that these differences are genetically based and not due to random chance. Previous mutation breeding successes in cowpea and related crops have come from recognizing and stabilizing such superior segregants. For example, [56] developed a high-yielding, large-seeded cowpea mutant variety by isolating an outstanding M₂ plant from a 250 Gy treatment and advancing it through selection. This resonates with our strategy of using multivariate clustering to pinpoint candidates for advancement. Moreover, our cluster analysis revealed that mutants derived from the same EMS dose did not always fall into a single cluster; indeed, some 20 mM mutants ended up in a poor-performing cluster, while some 80 mM mutants grouped with better performers. This indicates that while dose influences overall trends, the phenotypic expression of each mutant line is unique and must be evaluated on its own merit. Therefore, the use of clustering and PCA helps ensure that selection is based on actual trait patterns rather than preconceived notions of which treatment “should” yield better mutants.

In summary, the elite mutants isolated, including those in high-yielding-related and yield clusters, will be further evaluated in advanced generations. If their performance is stable, they could serve as new breeding germplasm or even be released as improved cowpea varieties, analogous to mutant lines released in other legumes, such as mungbean and chickpea, as noted by [54].

# Conclusion

This study demonstrates the effectiveness of ethyl methane sulfonate (EMS) in inducing substantial genetic variability in cowpea (*Vigna unguiculata* (L.) Walp), thereby broadening the phenotypic spectrum for key agronomic, yield, and seed traits. Contrast analysis revealed significant treatment effects and highlighted superior mutant lines, while principal component analysis (PCA) identified yield and reproductive traits as the primary drivers of phenotypic variation. These findings were consistently supported by cluster analysis, which grouped mutants according to overall agronomic performance. The convergence of these analytical approaches strengthened the selection of putative mutants, including high-yielding (B33, B31, B28, B22, C41, C05, D25, D45, D56), early-phenology (B19), and large-seeded or prolific podding lines (D34, C39, C40).

As the study was confined to the M₁ generation, further evaluation in the M₂ and M₃ generations is required to confirm trait stability, heritability, and breeding value. Complementary molecular analyses will be essential to explain the genetic basis of the observed variation and support marker-assisted selection. Overall, the selected mutants constitute valuable genetic resources for cowpea improvement and have strong potential to enhance productivity and global food security.

Considering the documented genetic variability of cowpea for nutritional traits such as protein and mineral content, the EMS-induced mutants identified in this study may serve as valuable genetic resources for future breeding programs targeting both yield and nutritional quality improvement .

# Acknowledgments

HMK, RANK, IKA, and FO conceived and designed the study. HMK and RANK performed the experiments. HMK analyzed the data and drafted the manuscript. RANK and IKA provided critical revisions. All authors read and approved the final manuscript. We gratefully acknowledge the Department of Plant and Environmental Biology at the University of Ghana, Legon, for supporting this research.

# References

1. Sert H, Ceyhan E. The effects of seed yield and some agricultural characters of different plant density on cowpea (Vigna sinensis (L.) Savi) in Hatay ecological conditions. Selcuk Journal of Agriculture and Food Sciences. 2012; 26(1): 34–43.
2. Harmankaya M, Ceyhan E, Çelik AS, Sert H, Kahraman A, Özcan MM. Some chemical properties, mineral content and amino acid composition of cowpeas (Vigna sinensis (L.) Savi). Quality Assurance and Safety of Crops & Foods. 2016;8(1): 111–116. <https://doi.org/10.3920/QAS2014.0487>
3. Jou-Nteufa C, Ceyhan E. Determination of combining ability and heredity through diallel analysis method in F₂ populations of cowpea. Selcuk Journal of Agriculture and Food Sciences. 2022;36(3): 299–311. <https://doi.org/10.15316/SJAFS.2022.039>
4. Oladosu Y, Rafii MY, Abdullah N, Hussin G, Ramli A, Rahim HA, et al. Principle and application of plant mutagenesis in crop improvement: A review. Biotechnology and Biotechnological Equipment. 2015;30(1):1–16.
5. Owusu EY, Karikari B, Kusi F, Haruna M, Amoah RA, Attamah P, et al. Genetic variability, heritability, and correlation analysis of maturity and yield traits in Cowpea (Vigna unguiculata (L.) Walp.) in Northern Ghana. Heliyon. 2021;7(9).
6. Bhat TA, Khan AH, Parveen S. Comparative analysis of meiotic abnormalities induced by gamma rays, EMS, and MMS in *Vicia faba* L. Journal of Indian Botanical Society. 2005; 84:45–58.
7. Olasupo FO, Ilori CO, Forster BP, Bado S. Mutagenic effects of gamma radiation on eight accessions of cowpea (*Vigna unguiculata* [L.] Walp.). Am J Plant Sci. 2016;7(2):339–51.
8. Hoque ME, Morshad MN. Somaclonal variation in potato (Solanum tuberosum L.) using chemical mutagens. The Agriculturists. 2014;12(1):15–25.
9. Opoku Gyamfi M, Eleblu JSY, Sarfoa LG, Asante IK, Opoku-Agyemang F, Danquah EY. Induced variations of ethyl methane sulfonate mutagenized cowpea (*Vigna unguiculata* L. Walp) plants. Front Plant Sci. 2022; 13:952247.
10. Dhanavel D, Girija M. Effect of EMS, DES, and SA on quantitative traits of cowpea (*Vigna unguiculata* (L.) Walp.) in M1 generation. Crop Research (Hisar). 2009;37(1–3):239–41.
11. Jou-Nteufa C, Ceyhan E. Genetic analysis of seed yield and some traits in cowpea using diallel analysis. Turkish Journal of Agriculture and Forestry. 2024;48(3): 278–293. <https://doi.org/10.55730/1300-011X.3180>
12. Dalgıç H, Ali A, Karadaş S, Korkmaz A, Ayran Çolak İ, Harmankaya M, Karaköy T, Ceyhan E, Türkoğlu A. (2025). Diallel analysis of cowpea (Vigna unguiculata L. Walp) for protein and mineral composition: Genetic variability and heritability. Euphytica. 2025:221: 168. [https://doi.org/10.1007/s10681-025-03618-0](https://doi.org/10.1007/s10681-025-03618-0)
13. Dhanavel D, Girija M. Effect of EMS on induced morphological mutants in cowpea (*Vigna unguiculata* (L.) Walp.). Crop Research. 2009;38:27–31.
14. Roychowdhury R, Tah J. Assessment of chemical mutagenic effects in mutation breeding programme for M1 generation of carnation (*Dianthus caryophyllus*). Res Plant Biol. 2011;1(4).
15. Gnankambary TK, Benoit JB, Sawadogo N, Mahamadou S, Tignegre JB, Yonli D, et al. Genetic variability induced by gamma radiation in cowpea (*Vigna unguiculata* L. Walp) in Burkina Faso. Eur Sci J. 2019;15(15):153–64.
16. Nawale SR, Apte UB, Jadhav BB. Effect of gamma rays and ethyl methane sulfonate on seed germination and survival of seedlings in cowpea (*Vigna unguiculata* (L.) Walp.). Journal of Arid Legumes. 2006;3(1):102–5.
17. Bind D, Dwivedi VK. Effect of Mutagenesis on Germination, Plant Survival, and Pollen Sterility in M1 Generation of in Cowpea [*Vigna unguiculata* (L.) Walp] [Internet]. Vol. 48, Indian J. Agric. Res. 2014. Available from: [www.arccjournals.com](http://www.arccjournals.com)
18. Crino P, Lai A, Bonito RD, Veronese P, Saccardo F. Genetic variability in tomato plants regenerated from irradiated cotyledons. In: Jain SM, Brar DS, Ahloowalia BS, editors. Somaclonal Variation and Induced Mutations in Crop Improvement. Dordrecht: Springer Science & Business Media; 2013.
19. Jain SM, Brar DS, Ahloowalia BS, editors. Somaclonal Variation and Induced Mutations in Crop Improvement. Dordrecht: Springer Science & Business Media; 2013.
20. Jain SM, Maluszynski M. In vitro applications in crop improvement: Recent progress. In: Mujib A, Cho M, Predieri S, Banerjee S, editors. Biotechnology for Crop Improvement. New Delhi, India: IBH–Oxford; 2004. p. 169–202.
21. Jain SM. Mutagenesis in crop improvement under the climate change. Rom Biotechnol Lett. 2010;15(2):88–106.
22. Kumar A, Rai PK. Induced mutagenesis in mungbean (*Vigna radiata* L.). In: Singh DP, editor. Mutation Breeding in Pulse Crops. Kalyani Publishers; 2006. p. 85–93.
23. Kundu SK, Singh DP. Gamma-ray induced variability for quantitative characters in black gram. Madras Agricultural Journal. 1982;69(10):644–8.
24. Chandra N. Cytological and mutational studies on Gloriosa superba Linn. [Nagpur, India]: Nagpur University; 1988.
25. Khan S, Wani MR. Comparison of the effect of chemical mutagens on mung bean. Advances in Plant Sciences. 2005;18(2):533–5.
26. Khan S, Wani MR. Chemomutagenic effect of two alkylating agents on mung bean (*Vigna radiata*). Bionotes. 2004;6(3):82–3.
27. Nanda S, Rao GM, Nayak S. Mutagenic effects of EMS on yardlong bean (*Vigna sesquipedalis*). Indian J Agric Res. 1997;31:602–10.
28. Allard RW. Principles of Plant Breeding. 2nd ed. New York: John Wiley & Sons; 1999.
29. Falconer DS, Mackay TFC. Introduction to Quantitative Genetics. 4th ed. Harlow: Longman; 1996.
30. Principles SBDPB, ed. New Delhi: Kalyani Publishers Methods 7th. Singh BD. Plant Breeding: Principles and Methods. 7th ed. New Delhi: Kalyani Publishers; 2006. 2006.
31. Grafius JE. Components of yield in oats: A geometrical interpretation. Agron J. 1959;51:515–8.
32. Donald CM. The breeding of crop ideotypes. Euphytica. 1968;17:385–403.
33. Gaul H. Mutations in plant breeding. Radiation Botany. 1964;4:155–232.
34. Maluszynski M, Nichterlein K, Van Zanten L, Ahloowalia BS. Officially released mutant varieties—The FAO/IAEA database. Mutation Breeding Reviews. 2000;12:1–84.
35. Goyal P, Khan S, Wani MR. EMS-induced genetic variability for yield and yield components in urdbean (*Vigna mungo* (L.) Hepper). Legume Research. 2021;44:1220–6.
36. Premsekhar S, Appadurai P. Effect of doses of gamma rays and EMS on induced mutation in pigeonpea. Indian Journal of Agricultural Sciences. 1982;51:381–4.
37. Singh BD. Plant Breeding: Principles and Methods. 7th ed. New Delhi: Kalyani Publishers; 2006.
38. Odeigah PGC, Osanyinpeju AO, Myers GO. Induced mutations in cowpea: Morphological and yield traits. Mutation Breeding Newsletter. 1998;44:10–3.
39. Rai A. Effect of Ethyl Methane Sulfonate Mutagen in M1 Generation in Pigeonpea (*Cajanas Cajan* L. Millasp). Rajmata Vijayaraje Scindia Krishi Vishwa Vidyalaya; 2011.
40. Khan IA. Mutation studies in mung bean (*Vigna radiata* L. Wilczek): IX. Estimates of genetic variability. Legume Research. 1988;11(2):89–93.
41. Singh M, Singh VP. Genetic analysis of certain mutant lines of urdbean for yield and quality traits in M4 generation. Indian Journal of Pulses Research. 2001;14(1):60–2.
42. Jagajanantham N, Dhanavel D, Pavadai P. EMS-induced variability in okra (*Abelmoschus esculentus* L.). Int J Sci Res. 2013;2:148–51.
43. Berenschot AS, Zucchi MI, Tulmann Neto A, Quecini V. Mutagenesis in ornamentals: Application and perspective. Plant Cell Tissue Organ Cult. 2009;98:161–72.
44. Premsekar R, Appadurai R. EMS-induced variability in pigeonpea (*Cajanus cajan* L.). Madras Agricultural Journal. 1982;69:458–66.
45. Mohanasundaram K, Kalamani A, Ramalingam RS. Induced mutagenesis in cowpea (*Vigna unguiculata* (L.) Walp.). Legume Research. 1999;22:642–8.
46. Anis M, Sharma Pk, Abbasi N. Gamma rays induced variability in urd bean (*Vigna mungo* (L.) hepper) I M1 generation. Journal of the Indian Botanical Society. 1999;78(1–2):111–3.
47. Khan SB, Siddiqui A, Nadeem M. Variation in quantitative characters of mung bean after seed treatment with DES. Advances in Plant Sciences. 1994;7(1):41–5.
48. Kansagra UM, Shukla PT. Variability studies in induced mutants of mungbean. Madras Agricultural Journal. 1993;80(4):180–4.
49. Iepziba SJ, Subramanian M. Variability in M3 and M4 mutants of urdbean. Indian Journal of Pulses Research. 1993;6(2):197.
50. Patil BN, Guha VY. Growth attributes in induced mutants of chickpea. PKV Research Journal. 1994;15(2):97–101.
51. Gnanamurthy S, Dhanavel D. Effect of EMS on induced morphological mutants and chromosomal variation in cowpea (*Vigna unguiculata* (L.) Walp.). International Letters of Natural Sciences. 2014;13:33–43.
52. Gyamfi MO. Inducing Genetic Variability In Cowpea (*Vigna Unguiculata*, L. Walp Var. Asontem) Using Ethyl Methane Sulfonate A Thesis Presented [Internet]. Available from: <http://ugspace.ug.edu.gh>
53. Wani AA. Induced mutagenesis for improvement of pulse crops. Plant Breed Rev. 2017;41:155–200.
54. Goyal S, Wani MR, Laskar RA, Raina A, Amin R, Khan S. Induction of morphological mutations and mutant phenotyping in black gram [*Vigna mungo* (L.) Hepper] using gamma rays and EMS. Vegetos. 2019;32(4):464–72.
55. Pandey B, Dhanasekar P. Development of high-yielding mutant varieties in cowpea. Indian Journal of Genetics. 2003;63:151–9.
56. Kumar Y, Mishra VK. Effect of gamma rays and diethyl sulphate on germination,growth, fertility and yield in greengram (*Vigna radiata* L. Wilczek). 1999.

# Supporting Information

**S1: Trait dataset and cluster grouping of individual M₁ cowpea plants across EMS treatments**

| **Trt** | **ID** | ***dtg*** | ***dff*** | ***d50f*** | ***dfh*** | ***d50mp*** | ***pdl*** | ***ln*** | ***sdl*** | ***sdw*** | ***sdt*** | ***swg*** | **yld** | **Cluster group** |
| --- | --- | --- | --- | --- | --- | --- | --- | --- | --- | --- | --- | --- | --- | --- |
| 0mM | A01 | 4 | 40 | 60 | 67 | 89 | 12.94 | 13 | 8.94 | 5.6 | 4.3 | 23.2 | 55.68 | 1 |
|  | A02 | 4 | 39 | 60 | 53 | 88 | 17 | 18 | 6.62 | 4.53 | 3.86 | 20.34 | 42.71 | 2 |
|  | A03 | 3 | 40 | 56 | 49 | 87 | 13.24 | 21 | 8.98 | 5.4 | 4.13 | 17.94 | 43.05 | 2 |
|  | A04 | 3 | 35 | 52 | 50 | 81 | 13.2 | 13 | 7.03 | 4.35 | 3.29 | 23.96 | 52.71 | 2 |
|  | A05 | 3 | 43 | 66 | 58 | 93 | 14.24 | 18 | 7.22 | 4.7 | 4.02 | 20.08 | 43.38 | 2 |
|  | A06 | 3 | 30 | 60 | 60 | 78 | 13.58 | 21 | 6.72 | 4.42 | 3.7 | 20.97 | 41.53 | 2 |
|  | A07 | 4 | 40 | 55 | 47 | 75 | 13.8 | 12 | 9.26 | 5.71 | 4.19 | 24.87 | 34.82 | 3 |
|  | A08 | 4 | 41 | 62 | 53 | 83 | 11.55 | 19 | 7.91 | 5.11 | 4.17 | 19.93 | 36.27 | 2 |
|  | A09 | 4 | 37 | 52 | 41 | 67 | 15.4 | 14 | 6.16 | 4.44 | 4.41 | 20.92 | 72.81 | 2 |
|  | A10 | 4 | 37 | 54 | 58 | 89 | 13.09 | 16 | 6.23 | 4.15 | 3.64 | 22.13 | 44.92 | 2 |
|  | A11 | 4 | 40 | 62 | 45 | 74 | 15.29 | 18 | 7.37 | 4.84 | 4.29 | 21.1 | 31.66 | 2 |
|  | A12 | 4 | 44 | 59 | 46 | 76 | 15.95 | 17 | 8.98 | 5.4 | 4.13 | 21.01 | 43.71 | 3 |
|  | A13 | 3 | 43 | 60 | 49 | 87 | 17.58 | 16 | 6.99 | 4.46 | 3.81 | 17.82 | 42.78 | 2 |
|  | A14 | 5 | 30 | 52 | 53 | 80 | 16.64 | 18 | 9.65 | 5.67 | 4.28 | 23.13 | 73.78 | 3 |
|  | A15 | 4 | 39 | 54 | 47 | 80 | 15.57 | 17 | 8.24 | 5.25 | 4.13 | 19.75 | 40.09 | 3 |
|  | A16 | 3 | 45 | 66 | 40 | 70 | 14.08 | 24 | 6.77 | 4.57 | 4.36 | 20.79 | 39.49 | 2 |
|  | A17 | 4 | 31 | 54 | 43 | 66 | 17.21 | 12 | 6.74 | 4.34 | 3.42 | 20.36 | 61.08 | 2 |
|  | A18 | 3 | 32 | 49 | 56 | 94 | 14.25 | 20 | 7.57 | 5.03 | 4.92 | 22.64 | 67.93 | 4 |
|  | A19 | 2 | 35 | 59 | 42 | 72 | 15.3 | 13 | 6.21 | 4.46 | 4.43 | 20.61 | 54.41 | 2 |
|  | A20 | 3 | 33 | 48 | 47 | 84 | 16.62 | 20 | 6.38 | 4.3 | 4.1 | 22.57 | 72.66 | 4 |
|  | A21 | 4 | 40 | 57 | 44 | 71 | 13.33 | 17 | 6.43 | 4.47 | 4.19 | 18.36 | 50.5 | 2 |
|  | A24 | 4 | 39 | 58 | 49 | 72 | 15.42 | 17 | 5.68 | 3.94 | 3.63 | 19.63 | 61.83 | 2 |
|  | A25 | 3 | 39 | 59 | 50 | 70 | 12.83 | 21 | 6.24 | 4.19 | 3.71 | 24.67 | 51.81 | 2 |
|  | A26 | 3 | 37 | 59 | 54 | 86 | 15.3 | 23 | 6.76 | 4.54 | 4.53 | 22.04 | 52.89 | 4 |
|  | A27 | 4 | 32 | 53 | 53 | 84 | 16 | 16 | 11.59 | 6.08 | 4.82 | 19.21 | 22.86 | 3 |
|  | A28 | 4 | 40 | 65 | 43 | 79 | 11.88 | 12 | 6.07 | 4.03 | 3.85 | 21.97 | 85.68 | 2 |
|  | A29 | 2 | 39 | 57 | 44 | 81 | 14.92 | 17 | 8.49 | 5.13 | 3.92 | 23.24 | 23.24 | 3 |
|  | A30 | 4 | 43 | 68 | 51 | 88 | 13.53 | 18 | 8.35 | 5.15 | 4.48 | 18.63 | 50.31 | 2 |
|  | A31 | 3 | 40 | 64 | 40 | 69 | 14.9 | 13 | 6.61 | 4.53 | 4.52 | 18.39 | 74.66 | 2 |
| 20mM | B01 | 5 | 50 | 70 | 68 | 92 | 12.13 | 18 | 6.33 | 4.24 | 4.06 | 22.28 | 55.69 | 5 |
|  | B02 | 4 | 46 | 66 | 59 | 98 | 13.23 | 21 | 6.65 | 4.38 | 3.66 | 26.08 | 28.69 | 5 |
|  | B03 | 5 | 42 | 60 | 53 | 79 | 14.98 | 13 | 6.94 | 4.58 | 4.23 | 25.41 | 37.36 | 5 |
|  | B04 | 6 | 43 | 60 | 60 | 100 | 16.28 | 18 | 9.26 | 5.71 | 4.19 | 25.92 | 54.44 | 1 |
|  | B05 | 5 | 47 | 66 | 62 | 82 | 13.8 | 21 | 10.1 | 5.78 | 4.92 | 25.17 | 49.83 | 1 |
|  | B06 | 5 | 51 | 75 | 54 | 76 | 15.75 | 12 | 10.35 | 5.76 | 4.91 | 25.9 | 62.15 | 6 |
|  | B07 | 7 | 38 | 60 | 63 | 89 | 11.45 | 19 | 11.65 | 6.44 | 5.19 | 29.99 | 67.18 | 6 |
|  | B08 | 5 | 49 | 74 | 68 | 102 | 15.25 | 14 | 9.85 | 5.67 | 5.12 | 22.68 | 41.27 | 1 |
|  | B09 | 7 | 52 | 74 | 71 | 98 | 13 | 21 | 11.59 | 6.08 | 4.82 | 27.48 | 74.2 | 6 |
|  | B10 | 6 | 47 | 63 | 56 | 81 | 14.31 | 23 | 7.03 | 4.48 | 3.79 | 20.06 | 46.14 | 2 |
|  | B11 | 7 | 52 | 70 | 60 | 95 | 13.3 | 16 | 9.75 | 5.66 | 4.99 | 26.1 | 26.1 | 3 |
|  | B12 | 3 | 41 | 59 | 49 | 87 | 17.7 | 13 | 10.4 | 5.9 | 5.16 | 26.32 | 48.7 | 6 |
|  | B13 | 4 | 47 | 62 | 66 | 100 | 14.25 | 18 | 9.9 | 5.72 | 4.87 | 23.49 | 59.2 | 1 |
|  | B14 | 7 | 53 | 69 | 58 | 87 | 14.5 | 21 | 7.18 | 4.88 | 4.76 | 24.86 | 45.75 | 5 |
|  | B15 | 3 | 54 | 71 | 55 | 76 | 15.25 | 16 | 9.75 | 5.88 | 4.83 | 27.77 | 79.15 | 1 |
|  | B16 | 5 | 53 | 75 | 64 | 102 | 14.7 | 18 | 9.75 | 5.68 | 4.85 | 26.24 | 75.56 | 1 |
|  | B17 | 3 | 41 | 65 | 71 | 93 | 13.7 | 17 | 10.4 | 5.82 | 4.84 | 27.34 | 71.37 | 6 |
|  | B18 | 6 | 54 | 71 | 62 | 98 | 15.5 | 12 | 10 | 5.62 | 4.89 | 28.33 | 45.32 | 3 |
|  | B19 | 5 | 36 | 51 | 48 | 68 | 19.88 | 19 | 6.23 | 4.17 | 4 | 24.82 | 93.59 | 4 |
|  | B20 | 5 | 34 | 57 | 52 | 72 | 14.7 | 14 | 6.77 | 4.57 | 4.36 | 26.63 | 42.6 | 5 |

Values highlighted red indicate trait means significantly (P<0.05) below the control, while green indicate values significantly (P<0.05) above the control.Key: Trt= Treatment ID=Genotype Identifier, dtg days to germination, dff = days to first flower, d50f = days to 50% flowering, dfh = days to first harvest, d50mp = days to 50% mature pods, pdl = pod length, pdw = pod width, ln = number of locules, nsp = number of seeds per pod, psa = percent seed abortion, sdl = seed length, sdw = seed width, sdt = seed thickness, swgt = seed weight, yld = yield per plant

**S1 Table: Trait dataset and cluster grouping of individual M₁ cowpea plants across EMS treatments (cont’d)**

| **Trt** | **ID** | ***dtg*** | ***dff*** | ***d50f*** | ***dfh*** | ***d50mp*** | ***pdl*** | ***ln*** | ***sdl*** | ***sdw*** | ***sdt*** | ***swg*** | **yld** | **Cluster group** |
| --- | --- | --- | --- | --- | --- | --- | --- | --- | --- | --- | --- | --- | --- | --- |
| 20mM | B21 | 5 | 47 | 63 | 70 | 91 | 13.6 | 13 | 9.85 | 5.64 | 5.09 | 22.6 | 81.37 | 1 |
|  | B22 | 6 | 36 | 56 | 70 | 97 | 12.1 | 20 | 7.04 | 4.98 | 4.85 | 21.91 | 88.96 | 1 |
|  | B23 | 7 | 48 | 73 | 55 | 79 | 13.04 | 17 | 8.26 | 5.04 | 3.93 | 22.04 | 46.28 | 5 |
|  | B24 | 6 | 50 | 74 | 52 | 75 | 13.35 | 13 | 8.7 | 5.13 | 4.91 | 17.81 | 18.7 | 3 |
|  | B25 | 7 | 39 | 61 | 58 | 89 | 17.75 | 18 | 9.65 | 5.31 | 4.52 | 25.92 | 76.99 | 1 |
|  | B26 | 4 | 47 | 72 | 64 | 100 | 14 | 21 | 10.1 | 5.54 | 4.87 | 24.26 | 91.45 | 1 |
|  | B27 | 7 | 39 | 55 | 56 | 85 | 16.1 | 12 | 7.04 | 4.68 | 4.28 | 25.59 | 67.56 | 4 |
|  | B28 | 4 | 39 | 60 | 67 | 87 | 12.13 | 19 | 7.3 | 4.75 | 4.04 | 23 | 103.5 | 1 |
|  | B29 | 6 | 43 | 66 | 61 | 94 | 13.27 | 14 | 6.62 | 4.53 | 3.86 | 24.2 | 45.73 | 5 |
|  | B30 | 7 | 40 | 61 | 63 | 95 | 14.1 | 13 | 6.46 | 4.29 | 4.1 | 23.82 | 28.58 | 5 |
|  | B31 | 3 | 39 | 60 | 58 | 98 | 14.85 | 20 | 9.9 | 5.46 | 5 | 24.65 | 107.22 | 1 |
|  | B32 | 6 | 47 | 71 | 49 | 85 | 14.22 | 17 | 6.14 | 4.06 | 3.87 | 22.04 | 66.11 | 4 |
|  | B33 | 6 | 37 | 61 | 56 | 85 | 16.33 | 19 | 6.1 | 4.05 | 3.31 | 27.88 | 125.44 | 4 |
|  | B34 | 7 | 41 | 57 | 53 | 85 | 12.44 | 17 | 7.04 | 4.98 | 4.85 | 22.2 | 38.86 | 5 |
|  | B35 | 7 | 51 | 68 | 56 | 91 | 14.75 | 13 | 10.05 | 5.24 | 3.56 | 26.06 | 63.85 | 5 |
|  | B36 | 4 | 33 | 54 | 64 | 100 | 14.1 | 19 | 10.15 | 5.18 | 3.91 | 23.62 | 64.48 | 1 |
|  | B37 | 7 | 53 | 73 | 62 | 82 | 14.35 | 17 | 11.25 | 5.77 | 3.71 | 26.15 | 82.38 | 1 |
|  | B38 | 5 | 43 | 61 | 61 | 81 | 14 | 13 | 10.3 | 5.01 | 3.84 | 27.88 | 42.94 | 5 |
|  | B39 | 6 | 54 | 71 | 49 | 85 | 13.25 | 19 | 10.4 | 5.67 | 4.46 | 26.71 | 86.53 | 1 |
|  | B40 | 7 | 37 | 58 | 61 | 90 | 16.6 | 15 | 10.2 | 5.5 | 4.4 | 26.06 | 68.8 | 6 |
|  | B41 | 7 | 33 | 50 | 60 | 99 | 17.52 | 18 | 6.43 | 4.47 | 4.19 | 25.04 | 66.61 | 4 |
|  | B42 | 5 | 49 | 74 | 69 | 107 | 12.7 | 20 | 6.74 | 4.34 | 3.42 | 26.21 | 46.13 | 5 |
|  | B43 | 3 | 37 | 56 | 58 | 96 | 12.25 | 18 | 9.3 | 4.73 | 3.72 | 23.01 | 68.33 | 5 |
|  | B44 | 6 | 48 | 70 | 69 | 107 | 14.43 | 19 | 6.65 | 4.66 | 4.27 | 21.38 | 29.94 | 5 |
|  | B45 | 3 | 48 | 67 | 62 | 96 | 13.75 | 20 | 10.1 | 5.57 | 4.08 | 23.17 | 76.45 | 1 |
|  | B46 | 6 | 46 | 65 | 55 | 80 | 10.5 | 18 | 9.5 | 4.7 | 3.72 | 27.56 | 46.86 | 5 |
|  | B47 | 4 | 52 | 70 | 59 | 89 | 14.5 | 19 | 10.15 | 5.15 | 3.97 | 24.42 | 72.54 | 1 |
|  | B48 | 4 | 39 | 59 | 72 | 105 | 14.5 | 14 | 9.65 | 5.36 | 3.86 | 25.18 | 30.22 | 5 |
| 40mM | C01 | 4 | 49 | 68 | 67 | 103 | 13.95 | 21 | 10 | 4.95 | 3.8 | 23.44 | 50.63 | 1 |
|  | C02 | 3 | 43 | 59 | 38 | 68 | 14.05 | 21 | 9.25 | 4.69 | 3.71 | 17.7 | 77.01 | 2 |
|  | C03 | 4 | 52 | 67 | 61 | 85 | 11.75 | 12 | 9.6 | 4.67 | 3.43 | 25.95 | 61.76 | 1 |
|  | C04 | 4 | 40 | 55 | 50 | 76 | 11.5 | 17 | 10 | 5.5 | 4.18 | 25.96 | 68.52 | 1 |
|  | C05 | 7 | 47 | 67 | 62 | 91 | 14.2 | 11 | 9.55 | 5.61 | 4.29 | 26.03 | 105.43 | 1 |
|  | C06 | 7 | 45 | 70 | 66 | 92 | 11.95 | 12 | 9.85 | 4.84 | 3.68 | 25.34 | 44.6 | 5 |
|  | C07 | 3 | 35 | 60 | 60 | 89 | 11.75 | 17 | 9.95 | 5.49 | 4.2 | 28.39 | 78.35 | 1 |
|  | C08 | 4 | 46 | 62 | 67 | 106 | 14 | 11 | 9.95 | 5.48 | 4.09 | 23.9 | 55.22 | 1 |
|  | C09 | 3 | 46 | 71 | 61 | 98 | 13.15 | 11 | 9.85 | 5.27 | 3.95 | 27.53 | 60.57 | 5 |
|  | C10 | 4 | 55 | 76 | 68 | 97 | 16.75 | 12 | 9.75 | 5.09 | 4 | 25.64 | 57.43 | 1 |
|  | C11 | 5 | 38 | 54 | 58 | 96 | 11 | 14 | 9.2 | 5.13 | 4.05 | 23.3 | 39.61 | 5 |
|  | C12 | 6 | 44 | 59 | 67 | 98 | 13.6 | 18 | 9.85 | 5.56 | 3.97 | 26.7 | 82.24 | 1 |
|  | C13 | 6 | 45 | 67 | 65 | 93 | 13.6 | 18 | 8.1 | 4.72 | 3.82 | 26.98 | 87.43 | 1 |
|  | C14 | 3 | 43 | 68 | 39 | 74 | 14.95 | 17 | 9.4 | 4.63 | 3.49 | 17.77 | 52.79 | 2 |
|  | C15 | 3 | 50 | 71 | 66 | 88 | 15.6 | 19 | 10 | 5.18 | 4.29 | 27.88 | 57.98 | 1 |
|  | C16 | 7 | 52 | 69 | 55 | 80 | 12.5 | 17 | 9.6 | 5.14 | 3.98 | 27.98 | 76.93 | 1 |
|  | C17 | 7 | 45 | 64 | 55 | 95 | 16.65 | 13 | 10.45 | 5.01 | 3.51 | 20.9 | 68.96 | 4 |
|  | C18 | 5 | 53 | 78 | 66 | 91 | 12.6 | 19 | 9.65 | 4.87 | 3.99 | 26.66 | 72.78 | 1 |
|  | C19 | 4 | 52 | 72 | 65 | 88 | 14.8 | 17 | 9.85 | 5.25 | 3.83 | 26.46 | 47.62 | 5 |
|  | C20 | 4 | 46 | 71 | 47 | 83 | 13.7 | 13 | 9.3 | 4.79 | 3.51 | 21.5 | 54.19 | 5 |
|  | C21 | 3 | 53 | 73 | 58 | 90 | 10.5 | 19 | 9 | 5.02 | 4.06 | 25.8 | 59.33 | 1 |

Values highlighted red indicate trait means significantly (P<0.05) below the control, while green indicate values significantly (P<0.05) above the control.Key: Trt= Treatment ID=Genotype Identifier, dtg days to germination, dff = days to first flower, d50f = days to 50% flowering, dfh = days to first harvest, d50mp = days to 50% mature pods, pdl = pod length, pdw = pod width, ln = number of locules, nsp = number of seeds per pod, psa = percent seed abortion, sdl = seed length, sdw = seed width, sdt = seed thickness, swgt = seed weight, yld = yield per plant

**S2 Table: Trait dataset and cluster grouping of individual M₁ cowpea plants across EMS treatments *(cont’d)***

| **Trt** | **ID** | ***dtg*** | ***dff*** | ***d50f*** | ***dfh*** | ***d50mp*** | ***pdl*** | ***ln*** | ***sdl*** | ***sdw*** | ***sdt*** | ***swg*** | **yld** | **Cluster group** |
| --- | --- | --- | --- | --- | --- | --- | --- | --- | --- | --- | --- | --- | --- | --- |
| 40mM | C22 | 5 | 55 | 73 | 51 | 78 | 14.9 | 15 | 10 | 5.5 | 4.32 | 25.76 | 27.04 | 3 |
|  | C23 | 5 | 43 | 64 | 55 | 81 | 12.05 | 18 | 9.6 | 4.79 | 3.84 | 21.7 | 42.96 | 1 |
|  | C24 | 4 | 43 | 65 | 56 | 76 | 13.5 | 11 | 9.8 | 5.31 | 3.85 | 26.02 | 94.7 | 1 |
|  | C25 | 4 | 40 | 59 | 62 | 102 | 12.5 | 12 | 9.4 | 4.81 | 3.63 | 23.51 | 55.96 | 5 |
|  | C26 | 7 | 51 | 70 | 54 | 88 | 11.95 | 14 | 9.85 | 5.25 | 3.77 | 23.6 | 69.4 | 1 |
|  | C27 | 5 | 48 | 69 | 67 | 95 | 6.95 | 19 | 9 | 4.58 | 5.59 | 23.01 | 48.33 | 1 |
|  | C28 | 7 | 49 | 72 | 67 | 91 | 15.95 | 17 | 10 | 5.33 | 3.86 | 26.46 | 47.62 | 5 |
|  | C29 | 5 | 39 | 62 | 56 | 92 | 15.4 | 13 | 10 | 4.98 | 3.81 | 25.45 | 42.76 | 5 |
|  | C30 | 3 | 37 | 57 | 62 | 93 | 14.9 | 20 | 10 | 5.58 | 3.98 | 21.34 | 59.76 | 1 |
|  | C31 | 5 | 42 | 59 | 50 | 83 | 12.75 | 18 | 10 | 4.88 | 3.57 | 27.56 | 48.24 | 5 |
|  | C32 | 7 | 34 | 49 | 59 | 84 | 14.95 | 19 | 9.85 | 4.91 | 3.87 | 27.16 | 62.74 | 4 |
|  | C33 | 3 | 52 | 77 | 50 | 76 | 12.25 | 19 | 9.35 | 5.33 | 4.21 | 24.58 | 63.91 | 1 |
|  | C34 | 3 | 44 | 68 | 57 | 93 | 15.75 | 15 | 8.65 | 5.03 | 4.35 | 24.24 | 56.71 | 4 |
|  | C35 | 5 | 48 | 70 | 58 | 94 | 15.45 | 18 | 9.85 | 4.88 | 4.01 | 23.82 | 60.04 | 1 |
|  | C36 | 3 | 54 | 74 | 54 | 83 | 13.95 | 12 | 10.05 | 4.78 | 3.63 | 24.73 | 61.83 | 1 |
|  | C37 | 4 | 49 | 67 | 51 | 76 | 13.9 | 17 | 9.8 | 5.28 | 4.08 | 23.83 | 31.45 | 1 |
|  | C38 | 6 | 34 | 59 | 62 | 89 | 8.77 | 11 | 8.95 | 5.06 | 3.81 | 19.95 | 39.5 | 5 |
|  | C39 | 6 | 37 | 57 | 52 | 92 | 15.5 | 19 | 10.1 | 4.98 | 3.91 | 22.71 | 27.02 | 5 |
|  | C40 | 6 | 35 | 58 | 72 | 98 | 13.5 | 17 | 10 | 5.4 | 3.68 | 24.03 | 28.83 | 5 |
|  | C41 | 5 | 55 | 80 | 64 | 89 | 14.5 | 13 | 10 | 4.83 | 3.71 | 26.36 | 90.96 | 1 |
|  | C42 | 7 | 36 | 59 | 58 | 95 | 13.35 | 19 | 10 | 5.59 | 4.49 | 25.19 | 25.19 | 5 |
|  | C43 | 5 | 35 | 53 | 71 | 95 | 14 | 15 | 10 | 5.55 | 4.45 | 27.28 | 90.01 | 1 |
|  | C44 | 5 | 41 | 57 | 55 | 95 | 15.5 | 18 | 10 | 4.97 | 4.14 | 25.65 | 76.17 | 1 |
|  | C45 | 3 | 40 | 59 | 54 | 84 | 15.75 | 12 | 11.05 | 5.07 | 3.8 | 27.77 | 83.32 | 1 |
|  | C46 | 5 | 52 | 71 | 64 | 103 | 16.15 | 17 | 10.9 | 5.69 | 4.25 | 24.74 | 59.87 | 1 |
|  | C47 | 4 | 40 | 57 | 63 | 87 | 13.95 | 11 | 10 | 5.58 | 4.09 | 23.3 | 37.51 | 3 |
|  | C48 | 4 | 50 | 73 | 62 | 92 | 16.75 | 13 | 10 | 5.19 | 3.99 | 19.97 | 19.97 | 3 |
|  | C49 | 7 | 39 | 55 | 59 | 80 | 14.75 | 8 | 10.4 | 5.29 | 3.92 | 22.82 | 60.26 | 3 |
|  | C50 | 3 | 51 | 69 | 62 | 84 | 14.5 | 9 | 10.1 | 5.39 | 3.93 | 23.53 | 54.59 | 5 |
|  | C51 | 4 | 53 | 70 | 62 | 87 | 12.5 | 13 | 9.9 | 5.3 | 3.96 | 19.66 | 21.63 | 3 |
|  | C52 | 4 | 39 | 60 | 64 | 86 | 15.9 | 8 | 9.9 | 5.17 | 4.02 | 23.13 | 60.13 | 4 |
|  | C53 | 6 | 39 | 63 | 62 | 85 | 14.75 | 9 | 9.75 | 5.22 | 3.9 | 19.28 | 75.19 | 4 |
|  | C54 | 5 | 41 | 59 | 54 | 80 | 16.55 | 12 | 10.15 | 5.58 | 3.55 | 25.23 | 55.51 | 3 |
|  | C55 | 7 | 53 | 70 | 51 | 89 | 14.5 | 11 | 9.85 | 4.76 | 3.69 | 25.06 | 52.62 | 5 |
| 80mM | D01 | 7 | 49 | 69 | 49 | 80 | 13.5 | 17 | 10 | 4.94 | 3.95 | 22.58 | 31.17 | 3 |
|  | D02 | 6 | 50 | 72 | 54 | 92 | 10.75 | 10 | 10.7 | 5.71 | 4.28 | 24.18 | 42.55 | 1 |
|  | D03 | 3 | 40 | 58 | 70 | 93 | 13.5 | 9 | 9.85 | 5.61 | 4.56 | 24.13 | 74.33 | 1 |
|  | D04 | 5 | 55 | 73 | 72 | 100 | 12.5 | 13 | 9.35 | 5.34 | 3.81 | 18.82 | 76.2 | 1 |
|  | D05 | 7 | 35 | 53 | 59 | 96 | 11.75 | 10 | 9.95 | 5.69 | 4.18 | 22.05 | 45.64 | 5 |
|  | D06 | 4 | 34 | 59 | 55 | 93 | 15 | 9 | 10.3 | 5.68 | 4.16 | 20.79 | 50.32 | 3 |
|  | D07 | 4 | 46 | 70 | 49 | 83 | 12.5 | 13 | 9.7 | 5.44 | 3.91 | 20.34 | 31.72 | 5 |
|  | D08 | 4 | 48 | 69 | 61 | 81 | 15.25 | 13 | 10.55 | 6.11 | 4.94 | 26.24 | 43.3 | 3 |
|  | D09 | 5 | 47 | 65 | 66 | 100 | 15 | 9 | 10.3 | 5.21 | 4.28 | 27.62 | 39.78 | 1 |
|  | D10 | 4 | 53 | 78 | 58 | 97 | 13.75 | 10 | 9.85 | 5.04 | 4.38 | 24.53 | 35.57 | 3 |
|  | D11 | 7 | 46 | 62 | 61 | 81 | 14 | 12 | 9.95 | 5.55 | 4.91 | 22.22 | 34 | 1 |
|  | D12 | 3 | 43 | 68 | 59 | 95 | 15.75 | 9 | 10.05 | 5.58 | 4.7 | 22.36 | 60.82 | 6 |

Values highlighted red indicate trait means significantly (P<0.05) below the control, while green indicate values significantly (P<0.05) above the control.Key: Trt= Treatment ID=Genotype Identifier, dtg days to germination, dff = days to first flower, d50f = days to 50% flowering, dfh = days to first harvest, d50mp = days to 50% mature pods, pel = peduncle length at first harvest, npp = number of pods per peduncle, nptm = number of pods per plant at maturity, nspt = number of seeds per plant, pdl = pod length, pdw = pod width, ln = number of locules, nsp = number of seeds per pod, psa = percent seed abortion, sdl = seed length, sdw = seed width, sdt = seed thickness, swgt = seed weight, yld = yield per plant

**S1 Table: Trait dataset and cluster grouping of individual M₁ cowpea plants across EMS treatments *(cont’d)***

| **Trt** | **ID** | ***dtg*** | ***dff*** | ***d50f*** | ***dfh*** | ***d50mp*** | ***pdl*** | ***ln*** | ***sdl*** | ***sdw*** | ***sdt*** | ***swg*** | **yld** | **Cluster group** |
| --- | --- | --- | --- | --- | --- | --- | --- | --- | --- | --- | --- | --- | --- | --- |
| 80mM | D13 | 6 | 42 | 59 | 69 | 91 | 16.5 | 13 | 10.05 | 5.2 | 4.67 | 27 | 64.8 | 1 |
| 80mM | D14 | 6 | 52 | 74 | 45 | 81 | 12.75 | 12 | 9.4 | 5.37 | 4.46 | 24.66 | 54.24 | 1 |
| 80mM | D15 | 4 | 43 | 68 | 61 | 92 | 14.5 | 9 | 10.5 | 5.47 | 4 | 21.45 | 36.03 | 3 |
| 80mM | D16 | 5 | 48 | 68 | 65 | 92 | 13 | 13 | 9.9 | 5.02 | 3.95 | 22.74 | 68.23 | 1 |
| 80mM | D17 | 6 | 40 | 56 | 64 | 86 | 12.75 | 11 | 9.4 | 5.91 | 4.89 | 23.07 | 49.84 | 6 |
| 80mM | D18 | 3 | 44 | 68 | 64 | 101 | 11.45 | 7 | 9.95 | 4.94 | 3.97 | 25.55 | 35.77 | 1 |
| 80mM | D19 | 5 | 43 | 58 | 59 | 91 | 14.5 | 12 | 11 | 5.24 | 3.41 | 23.94 | 55.07 | 5 |
| 80mM | D20 | 4 | 55 | 75 | 64 | 103 | 11.25 | 13 | 10.75 | 5.41 | 4.21 | 22.04 | 65.9 | 1 |
| 80mM | D21 | 3 | 37 | 59 | 59 | 97 | 17.5 | 8 | 11 | 5.35 | 3.74 | 24.48 | 42.83 | 3 |
| 80mM | D22 | 7 | 47 | 62 | 63 | 92 | 14.5 | 14 | 9.35 | 4.73 | 3.74 | 20.59 | 40.77 | 4 |
| 80mM | D23 | 7 | 37 | 55 | 57 | 78 | 16 | 13 | 9.35 | 5.21 | 3.94 | 22.7 | 25.88 | 3 |
| 80mM | D24 | 4 | 40 | 62 | 72 | 109 | 14.25 | 8 | 9.15 | 4.95 | 4.07 | 17.35 | 58.65 | 4 |
| 80mM | D25 | 3 | 45 | 68 | 68 | 90 | 15 | 9 | 11 | 5.62 | 4.15 | 24.44 | 102.66 | 1 |
| 80mM | D26 | 4 | 46 | 63 | 71 | 110 | 17.1 | 13 | 8.95 | 5.02 | 3.86 | 22.7 | 22.7 | 3 |
| 80mM | D27 | 4 | 44 | 62 | 61 | 86 | 16.25 | 8 | 10 | 5.31 | 4.04 | 21.91 | 42.73 | 4 |
| 80mM | D28 | 6 | 43 | 66 | 60 | 92 | 17.1 | 9 | 10.45 | 5.48 | 4.28 | 22.32 | 75 | 4 |
| 80mM | D29 | 7 | 39 | 62 | 58 | 82 | 12.75 | 12 | 7.95 | 4.28 | 3.45 | 21.58 | 69.93 | 4 |
| 80mM | D30 | 4 | 35 | 60 | 56 | 84 | 12.75 | 11 | 10.25 | 5.02 | 3.79 | 25.06 | 36.08 | 5 |
| 80mM | D31 | 4 | 35 | 55 | 54 | 87 | 14.9 | 17 | 10.75 | 5.17 | 4.02 | 21.98 | 24.17 | 5 |
| 80mM | D32 | 4 | 44 | 62 | 68 | 96 | 14.15 | 11 | 10.2 | 6.05 | 4.82 | 22.16 | 37.23 | 1 |
| 80mM | D33 | 4 | 37 | 60 | 48 | 85 | 11.25 | 7 | 10.3 | 4.81 | 3.92 | 25.86 | 46.54 | 5 |
| 80mM | D34 | 4 | 45 | 66 | 52 | 83 | 12.25 | 12 | 10.25 | 4.97 | 3.46 | 21.21 | 23.75 | 3 |
| 80mM | D35 | 7 | 41 | 66 | 58 | 81 | 16.75 | 13 | 9.3 | 5.09 | 4.06 | 21.79 | 35.96 | 3 |
| 80mM | D36 | 3 | 53 | 71 | 49 | 73 | 14 | 8 | 11 | 5.64 | 4.37 | 28.29 | 74.69 | 6 |
| 80mM | D37 | 4 | 43 | 61 | 59 | 80 | 15.25 | 9 | 10 | 5.38 | 4.08 | 25.05 | 50.86 | 5 |
| 80mM | D38 | 5 | 42 | 57 | 62 | 88 | 15.5 | 10 | 9.3 | 5.14 | 3.5 | 17.49 | 44.24 | 4 |
| 80mM | D39 | 4 | 40 | 56 | 52 | 90 | 15.75 | 9 | 9.4 | 5.3 | 4.06 | 19.27 | 48.57 | 3 |
| 80mM | D40 | 4 | 44 | 69 | 54 | 77 | 9.95 | 13 | 8.55 | 5.18 | 3.96 | 19.42 | 55.55 | 2 |
| 80mM | D41 | 7 | 45 | 63 | 59 | 81 | 15 | 12 | 9 | 4.79 | 3.75 | 19.76 | 80.03 | 4 |
| 80mM | D42 | 3 | 55 | 80 | 66 | 96 | 9.95 | 11 | 9.9 | 4.72 | 3.72 | 22.02 | 29.06 | 3 |
| 80mM | D43 | 3 | 54 | 73 | 48 | 69 | 11.7 | 17 | 10.5 | 4.9 | 3.67 | 21.98 | 42.19 | 1 |
| 80mM | D44 | 6 | 48 | 65 | 52 | 86 | 9.5 | 12 | 11.6 | 6.03 | 4.46 | 24.35 | 64.29 | 1 |
| 80mM | D45 | 4 | 47 | 68 | 60 | 92 | 11.5 | 9 | 9.8 | 5.4 | 4.22 | 23.58 | 106.09 | 1 |
| 80mM | D46 | 3 | 44 | 62 | 57 | 78 | 12.5 | 13 | 9.8 | 5.05 | 4.15 | 21.53 | 45.21 | 3 |
| 80mM | D47 | 3 | 38 | 59 | 58 | 78 | 13 | 13 | 9.85 | 5.08 | 3.99 | 18.45 | 18.45 | 3 |
| 80mM | D48 | 5 | 47 | 65 | 52 | 84 | 13.65 | 8 | 10 | 5.3 | 4.24 | 22.52 | 85.13 | 1 |
| 80mM | D49 | 7 | 52 | 72 | 62 | 83 | 11.2 | 9 | 10.45 | 5.48 | 4.03 | 22.93 | 79.1 | 1 |
| 80mM | D50 | 3 | 42 | 60 | 52 | 92 | 13.1 | 12 | 9.65 | 4.82 | 3.7 | 22.5 | 43.19 | 5 |
| 80mM | D51 | 7 | 33 | 56 | 50 | 83 | 8.45 | 11 | 9.55 | 4.76 | 3.84 | 22.62 | 64.7 | 5 |
| 80mM | D52 | 5 | 55 | 77 | 56 | 84 | 14.2 | 17 | 10.4 | 4.87 | 3.87 | 19.54 | 22.48 | 3 |
| 80mM | D53 | 7 | 43 | 62 | 59 | 99 | 11.4 | 12 | 9.8 | 5.51 | 4.38 | 22.83 | 55.25 | 1 |
| 80mM | D54 | 5 | 47 | 71 | 60 | 85 | 11.5 | 9 | 9.8 | 5.44 | 4.1 | 20.24 | 28.33 | 3 |
| 80mM | D55 | 4 | 37 | 62 | 43 | 74 | 14.25 | 13 | 9.05 | 4.85 | 3.85 | 20.31 | 35.34 | 2 |
| 80mM | D56 | 5 | 53 | 69 | 57 | 94 | 13.5 | 23 | 10.15 | 5.03 | 3.78 | 28.68 | 111.85 | 1 |

Values highlighted red indicate trait means significantly (P<0.05) below the control, while green indicate values significantly (P<0.05) above the control.Key: Trt= Treatment ID=Genotype Identifier, dtg = days to germination, dff = days to first flower, d50f = days to 50% flowering, dfh = days to first harvest, d50mp = days to 50% mature pods, pdl = pod length, pdw = pod width, ln = number of locules, nsp = number of seeds per pod, psa = percent seed abortion, sdl = seed length, sdw = seed width, sdt = seed thickness, swgt = seed weight, yld = yield per plant

**S2 Table:** PCA loadings and variance explained for traits in M₁ cowpea

| **Traits** | **PC1** | **PC2** | **PC3** | **PC4** | **PC5** | **PC6** | **PC7** | **PC8** | **Uniqueness** |
| --- | --- | --- | --- | --- | --- | --- | --- | --- | --- |
| sdl | 0.73 |  |  |  |  |  |  |  | 0.18 |
| sdw | 0.70 |  | 0.50 |  |  |  |  |  | 0.11 |
| dfh | 0.68 |  |  | 0.50 |  |  |  | -0.32 | 0.14 |
| dff | 0.61 |  | -0.64 |  |  |  |  |  | 0.10 |
| d50mp | 0.59 |  |  | 0.51 |  |  |  | -0.40 | 0.13 |
| d50f | 0.57 |  | -0.68 |  |  |  |  |  | 0.09 |
| swg | 0.53 |  |  |  | 0.35 |  |  | 0.43 | 0.36 |
| sdt | 0.38 |  | 0.42 |  |  | 0.52 | -0.38 |  | 0.24 |
| nspt |  | 0.96 |  |  |  |  |  |  | 0.02 |
| yld |  | 0.95 |  |  |  |  |  |  | 0.02 |
| nsp |  | 0.75 |  | 0.36 | -0.38 |  | -0.32 |  | 0.03 |
| nptm |  | 0.32 |  | -0.66 |  |  | 0.53 |  | 0.04 |
| pdl |  |  | 0.42 |  |  |  |  |  | 0.77 |
| pdw |  |  |  |  | 0.54 | -0.35 |  | 0.32 | 0.40 |
| ln |  |  |  |  | 0.47 | 0.63 |  |  | 0.22 |
| dtg |  |  |  |  | 0.46 | -0.33 |  |  | 0.51 |
| psa |  |  |  |  | -0.42 |  |  | 0.32 | 0.52 |
| npp |  |  |  |  |  | 0.32 | 0.41 | 0.43 | 0.43 |
| **Eigenvalue** | **3.18** | **2.71** | **1.70** | **1.51** | **1.34** | **1.17** | **1.07** | **1.03** |  |
| **Proportion %** | **17.70** | **15.10** | **9.40** | **8.40** | **7.50** | **6.50** | **6.00** | **5.70** |  |
| **Cumulative %** | **17.70** | **32.70** | **42.20** | **50.60** | **58.00** | **64.50** | **70.50** | **76.20** |  |

PC= principal component, dtg = days to germination, dff = days to first flower, d50f = days to 50% flowering, dfh = days to first harvest, d50mp = days to 50% mature pods, npp = number of pods per peduncle, nptm = number of pods per plant at maturity, nspt = number of seeds per plant, pdl = pod length, pdw = pod width, ln = number of locules, nsp = number of seeds per pod, psa = percent seed abortion, sdl = seed length, sdw = seed width, sdt = seed thickness, swgt = seed weight, yld = yield per plant
